# A Diazo-free Equivalent of the Unsubstituted Carbyne Cation: Straightforward Synthesis of Naphthalenes and Pyridines via [^12/13^CH]^+^ Insertion

**DOI:** 10.1021/jacs.5c21901

**Published:** 2026-01-19

**Authors:** Nicola S. Wenzel, Philipp C. Brehm, Maike Mücke, Monish A. Ansari, Brigitte Worbs, Martin Simon, Christopher Golz, Ricardo A. Mata, Manuel Alcarazo

**Affiliations:** † Institut für Organische und Biomolekulare Chemie, 9375 Georg-August-Universität Göttingen, Tammannstr. 2, Göttingen 37077, Germany; ‡ Institut für Physikalische Chemie, 9375 Georg-August-Universität Göttingen, Tammannstr. 6, Göttingen 37077, Germany

## Abstract

Stable isotope labeling is a crucial technique in pharmaceutical research to understand the mode of action and metabolism of new drug candidates; however, its utility is often jeopardized by the synthetic challenges associated with the installation of the isotopic label into the core of the structures under study. Herein, we address this problem for the case of ^13^C-labeled naphthalene and pyridine building blocks. Our synthetic protocol utilizes the sulfonium sulfaneylidene salt **1**, a benchtop stable reagent that does not incorporate diazo functionalities in its structure; yet, under Rh-catalysis, it efficiently acts as a synthetic equivalent of the simplest conceivable carbynyl cation, the [CH]^+^ fragment. Mechanistic experiments supported by DFT calculations suggest the initial formation of a sulfonio-substituted Rh­(II)-carbene that reacts with indenes or pyrroles to initially form sulfonio-substituted cyclopropanes; the diastereoselectivity of this step being inconsequential because both isomers interconvert under the reaction conditions. Subsequent electrocyclic ring expansion with the concomitant elimination of dibenzothiophene delivers the desired naphthalenes or pyridines.

## Graphical abstract

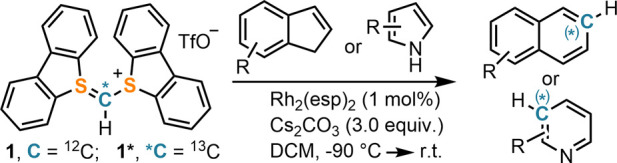

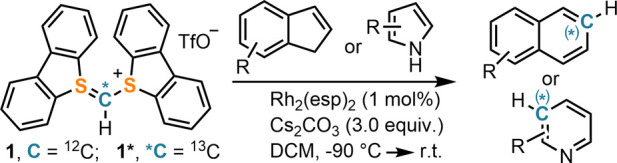

## Introduction

Stable isotope labeled drug candidates are intensively used by drug metabolism scientists and toxicologists to investigate the so-called ADMET profile, absorption, distribution, metabolism, excretion, and toxicology, of new active agents.^1^ This practice avoids the legal restrictions and safety issues associated with the handling and disposal of radioactively labeled substances and strongly profits from the advances of modern NMR spectroscopy and LC/MS technologies, which allow the detection, structural determination, and accurate quantification of metabolites even in biological matrices.^2^ Areas such as crop protection,^3^ quality control,^4^ or human food safety evaluation also benefit from the easy tracking of stable isotopomers.^5^ The heavy stable isotopes more likely to be used for the labeling of biomolecules are ^2^H, ^13^C, and ^15^N, with natural abundances of 0.015, 1.10, and 0.366%, respectively;^6^ yet, ^13^C-labeled tracers are often preferred due to the ubiquity of carbon in the skeleton of biomolecules, the stability of the label since deuterium is easily exchangeable with normal hydrogen in protic solution,^7^ and the lower cost of ^13^C-labeled building blocks when compared with ^15^N-containing ones.^8^ Even though it often represents a synthetic challenge, ^13^C-labels are preferably installed in a late step of the synthetic route in order to minimize the amount of the labeling reagent used and reduce costs. Moreover, the label should ideally be embedded in the core skeleton of the drug candidate to prevent its possible loss at an early stage of the metabolic process.

This set of demands predestines modern skeletal editing techniques as the tool of choice to deal with the incorporation of ^13^C-labels into drug-type structures;^9^ however, for the specific case of carbon atom insertion reactions, two main limitations hamper the general practicability of this synthetic approach. The available carbynyl cation equivalents do not insert the ubiquitous carbyne unit (=CH−), but a derivative (=C*R–*), whose structure is dictated by the stability requirements of the carbyne transfer reagent employed, and not by the final topology of the target molecule.^10^ For example, diazo-based reagents such as **A**,^11^ **B,** ^12^ **C,** ^13^ and analogues^14^ have been used for the transformation of indenes and indoles into naphthalenes^15^ and isoquinolines, respectively;^16^ yet, these species require for stability reasons a hanging electron withdrawing substituent attached to the azomethine carbon, which is ultimately transferred to the final products as well. Similarly, the 3-chlorodiazirines **D** that have been used for skeletal editing bear, with no exception, an aryl moiety attached at position 3.^17^ This situation forces the implementation of additional steps to either eliminate or interconvert the superfluous substituent installed^18^ or to design families of reagents where each member is characterized by a different hanging function, as in the case of sulfenyl carbene precursors of general formula **E**.^19^ No less important is the fact that no ^13^C-versions of the carbynyl cation equivalents **A**–**E** have been described, arguably because their syntheses from the available ^13^C-labeled building blocks are long and/or costly.

Being aware of the reactivity similarities between diazo compounds and sulfur ylides,^20^ and more specifically, about the suitability of the latter to serve as carbene precursors,^21^ we hypothesized that the replacement of the diazo group by a S-ylide in α-diazo sulfonium salts might offer the possibility to design reagents able to depict carbynyl cation reactivity, but based on a thermally more stable (sulfaneylidene)­sulfonium platform (Figure 1c).^22^ Once the desired stability is achieved, the presence of electron withdrawing groups attached to the central carbon atom is not relevant, and they can be eliminated. In addition, (sulfaneylidene)­sulfonium salts offer an advantage that qualifies them for ^13^C-labeling; their synthesis is straightforward from the corresponding organic sulfide and MeOTf, which is one of the few reagents whose ^13^C-derivative is still affordable in terms of price. Herein, we bring this unprecedented design of a carbynyl cation equivalent into practice and report the preparation of reagent **1** and its implementation into protocols to access naphthalenes and pyridines via CH-insertion processes.

**Figure 1:**
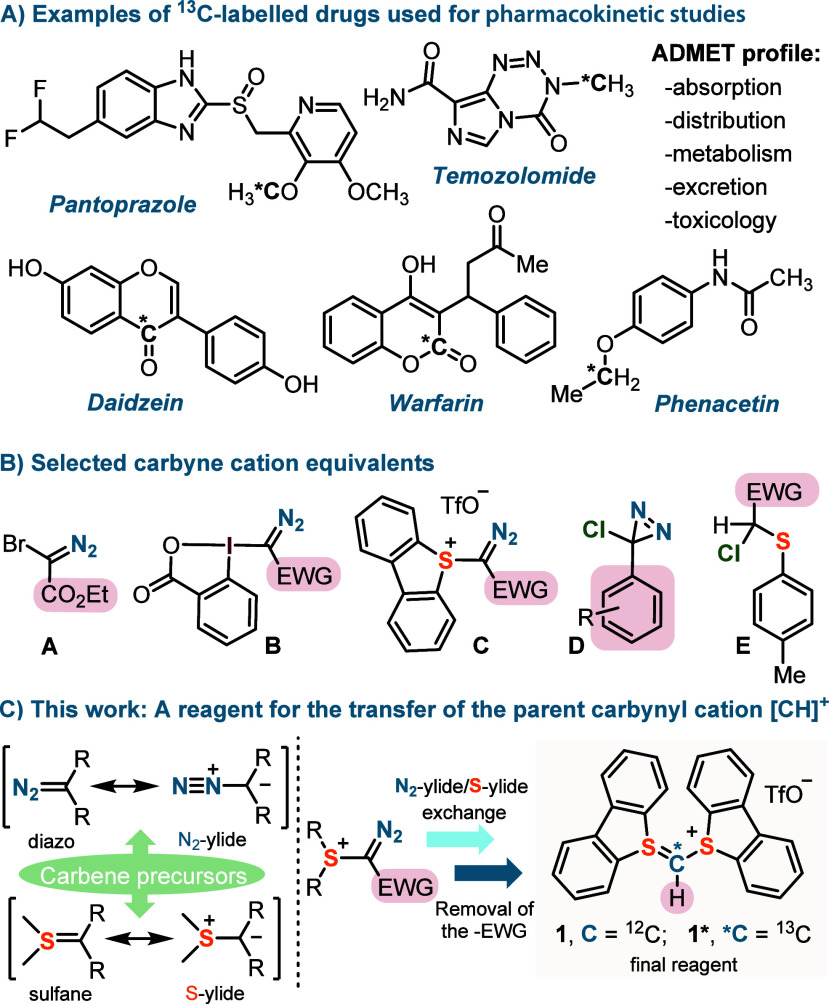
^13^C-labeling in clinical pharmacology. (A) Representative ^13^C-labeled drugs used for ADMET profile determination. (B) Available carbynyl cation equivalents that have been used for skeletal editing. (C) Our approach: a diazo-free reagent for the transfer of the parent [CH]^+^ moiety.

## Results and Discussion

A two-step synthetic route was developed for the preparation of reagent **1**. Treatment of dibenzothiophene **2** with methyl triflate at 60 °C delivers 5-methyldibenzothiophenium triflate **3** as an air stable white microcrystalline solid.^23^ Following a similar method, dibenzothiophene *S*-oxide **4** was methylated to deliver the methoxy substituted sulfonium salt **5**.^24^ This salt can be handled in air for short periods of time, but storage under N_2_ is required. For the last step of the synthesis, **3** is deprotonated using LDA at −90 °C, generating the corresponding S-ylide *in situ*, which is made to react with **5** to deliver **1**. Several multigram batches of salt **1** have been obtained through this procedure in a reproducible 60–65% yield (Figure 2A and the Supporting Information). The protocol is equally successful for the preparation of the ^13^C-labeled version of the reagent (1*).

**Figure 2:**
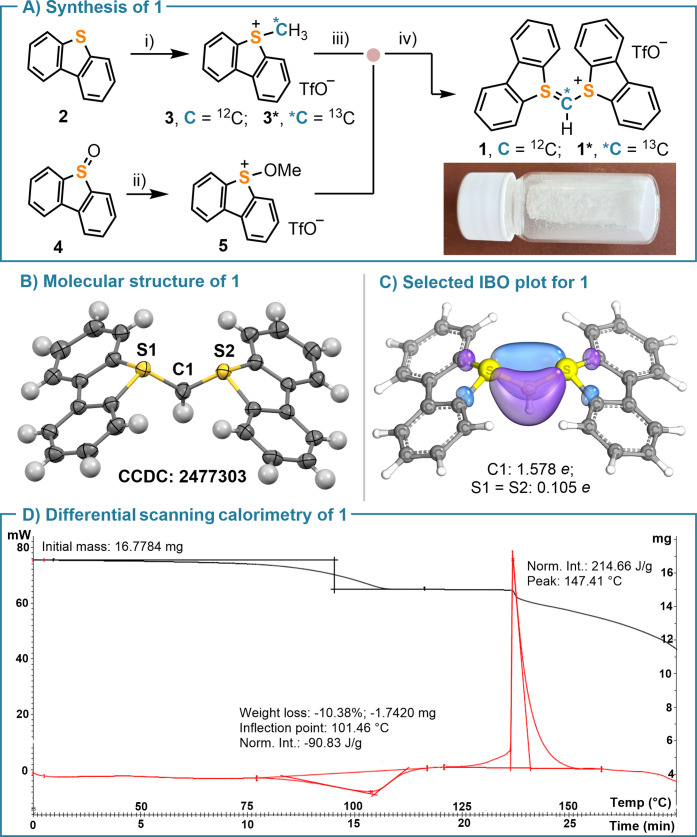
(A) Synthesis of salt **1**; reagents and conditions, yields are in parentheses: (i) TfOMe (1.2 equiv) or ^13^C-TfOMe (1.05 equiv), CH_2_Cl_2_, 60 °C, 16 h, **3** (91%); 3* (85%); (ii) TfOMe (1.5 equiv), CH_2_Cl_2_, 0 °C → r.t., 16 h, **5** (98%); (iii) LDA (1.05 equiv), THF, −90 °C and then **5** (1.0 equiv), −90 °C → r.t., 20 h, **1** (62%), 1* (58%). (B) Molecular structure of **1** in the solid state. Anisotropic displacement shown at the 50% probability level; solvent molecules and triflate anions removed for clarity. Selected metrical parameters: S1–C1 = 1.674(3) Å; S2–C1 = 1.682 (3) Å; S1–C1–S2 = 113.8(1)°. (C) Selected IBO plots for **1**, threshold value for printing: 80. (D) DSC analysis for **1** in air.

X-ray diffraction analysis of monocrystals of **1** confirmed the expected connectivity (Figure 2B). The cationic part of this salt possesses a nearly perfect *C*_*2v*_-symmetry, with both sulfur atoms adopting the expected trigonal–pyramidal coordination environment; the S1–C1–S2 bond angle (113.8°(1)) denotes sp^2^ hybridization of the central C atom. The analysis of the S1–C1 and S2–C1 bond lengths (1.674(3) and 1.682(3) Å, respectively) is quite indicative. These distances are closer to the ones typically observed for S–C double bonds (1.60 Å) than to those characteristics of S–C single bonds (1.80–1.84 Å);^25^ hence, they indicate a non-negligible π-interaction along the S1–C1–S2 fragment (Figure 2B).

In an attempt to better understand the bonding situation in **1**, its geometry was optimized at the B3LYP-D3­(BJ)/def2-TZVP level of theory and an IBO analysis was carried out at the default level (PBE/def2-TZVP/univ-JFIT) (see Figure 2C for selected IBO and Figure S3 for more details).^26^ In line with our interpretation of the crystallographic data, a π electron pair is located mainly at the central carbon atom, with some delocalization at the two flanking sulfur atoms. Natural population analysis (B3LYP/6-31G*) indicates that in **1** each sulfur atom bears a nearly entire positive charge (+0.96*e* for S1 and S2), while the central carbon is negatively charged (−0.95*e*). The Wiberg and Mayer bond indices for the identical S1–C1 and S2–C1 interactions are 1.61 and 1.30, respectively.

A differential scanning calorimetry (DSC) analysis was carried out for **1**. It shows an initial endothermic peak centered at 101.5 °C, which corresponds to the loss of cocrystallized THF, followed by the exothermal decomposition of the reagent that starts at 125 °C, and leads to a moderate energy release (214.7 J/g). This value is significantly lower than those reported for typical diazo-based cationic carbyne transfer reagents (**B**, EWG = CO_2_Et, 690 J/g @ 119 °C;^13^ **C**, EWG = CO_2_Et, 400 J/g @ 152 °C);^14^ the Yoshida correlation predicts that **1** is neither explosive nor impact sensitive (Figure 2D).^27^ We also conducted an isothermal aging experiment at 60 °C for 24 h, which revealed no loss of mass. Altogether, these results suggest that the use of **1** for multigram laboratory-scale syntheses is safe.

With multigram amounts of reagent **1** in hand, we examined its potential for ring expansion reactions initially using 4,7-dimethylindene **6a** as a model substrate. Several catalysts were screened, but as in the case of the mechanistically related N atom insertion reactions,^28^ Rh_2_(esp)_2_ (esp = α,α,α′,α′-tetramethyl-1,3-benzenedipropionate) afforded the best results in terms of yields (Figure 3A,C).^29^ Optimization of the base revealed that Cs_2_CO_3_ was crucial for the efficient formation of the desired naphthalene **7a**; when no base was added, or alternatively Na_2_CO_3_ or K_3_PO_4_ was used, the yields of **7a** decreased. In these cases, NMR analysis of the crudes indicated the formation of substantial amounts of *exo*-cyclopropyl sulfonium salt *exo-* **8a** alongside **7a** (Figure 3A, entries 2–4). This intermediate was isolated, and its structure was unambiguously confirmed by X-ray crystallography (Figure 3B).^30^ Prolonged heating (110 °C, DCE, 4 days) is necessary to reach the conversion of *exo-* **8a** into **7a** in the presence of K_3_PO_4_ (entry 5); however, no trace of *exo-* **8a** is observed when Cs_2_CO_3_ is employed as the base, even if the reaction is carried out at room temperature (entry 1). Actually, when a CD_2_Cl_2_ solution of isolated *exo-* **8a** is treated at room temperature with solid Cs_2_CO_3_ (1.0 equiv), it cleanly evolves into **7a** in the course of 16 h. We infer from that experiment that Cs_2_CO_3_ efficiently equilibrates the *exo-* and *endo-*isomers of **8a** via a deprotonation–protonation process that involves the corresponding S-ylide; once *endo-* **8a** is formed, it easily opens to the naphthalene. The experimental p*K*_a_ values available for sulfonium salts of analogous structures (p*K*_a_ = 16–18),^31^ and the well-established use of Cs_2_CO_3_ as a base of choice for the mild deprotonation of organic substrates of similar acidity,^32^ including tosylamides and tosylhydrazones,^33^ phosphonates,^34^ or even structurally related sulfonium salts,^35^ support this scenario.

**Figure 3:**
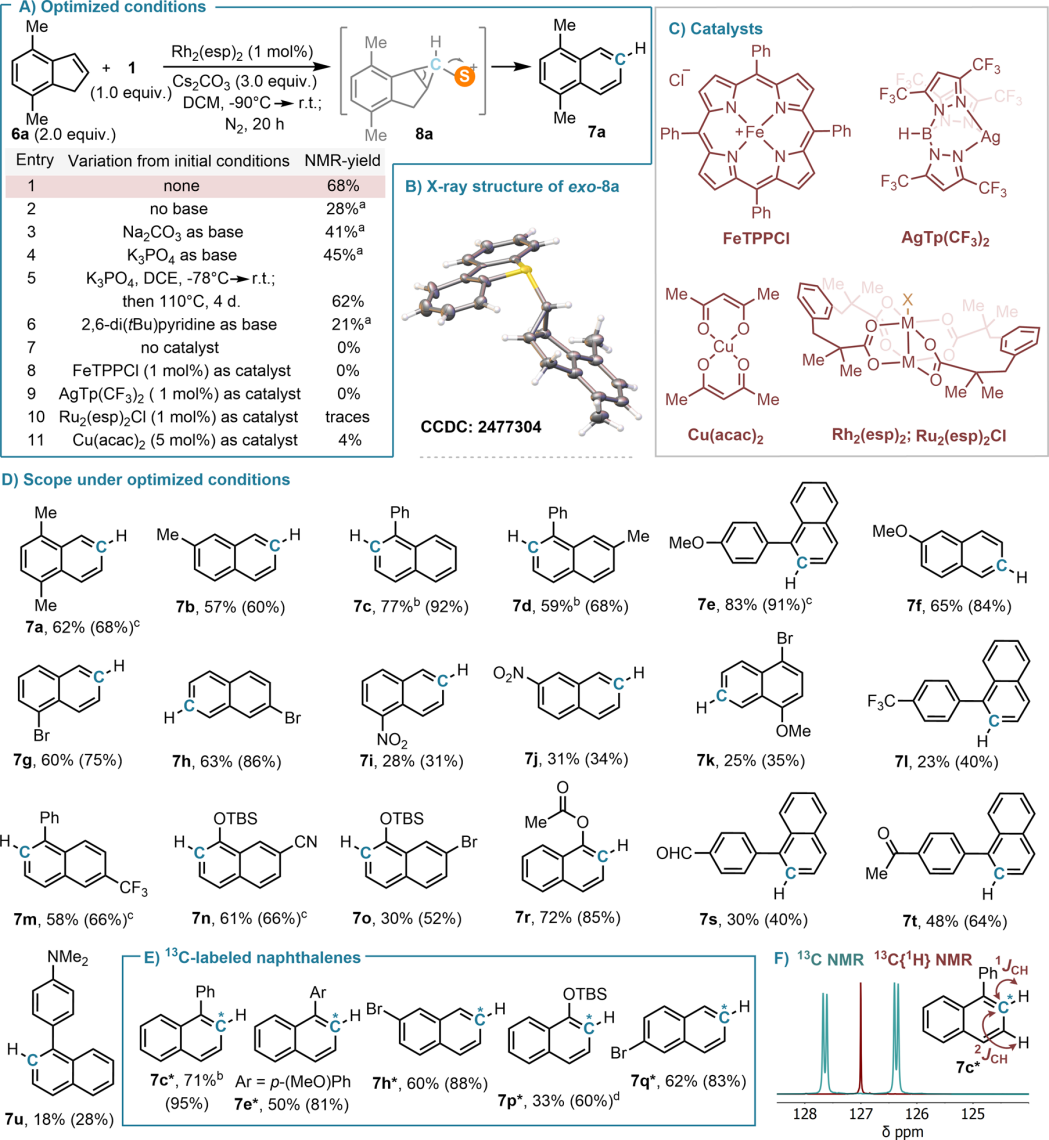
Synthesis of naphthalenes from indenes. (A) Reaction optimization table. (B) Structure of *exo* **-8a** in the solid state determined by X-ray analysis. Anisotropic displacement shown at the 50% probability level; solvent molecules and triflate anions removed for clarity. (C–E) Substrate scope for the synthesis of naphthalenes and ^13^C-labeled naphthalenes via C atom insertion. Yields are of the isolated products. ^1^H NMR conversions are in parentheses; determined using CH_2_Br_2_ as an internal standard. (F) ^13^C NMR and ^13^C­{^1^H} NMR of 7c*. ^a^Compound *exo* **-8a** was detected in these experiments. ^b^Pyridine (0.5 equiv) was additionally added to these experiments. ^c^Naphthalene products were isolated using a AgNO_3_-doped thin layer preparative silica gel plate. ^d^Naphthol 12* is isolated in 45% yields from **6p** if TBS-deprotection is carried out before column chromatography.

Making use of the optimized conditions, we subsequently investigated the scope and functional group compatibility of this insertion reaction. Alkyl, aryl, and (silyl) ethers, esters, ketones, and halogen substituents are tolerated, and the corresponding naphthalenes are obtained in good, isolated yields (Figure 3D). The reaction also proceeds in the presence of nitro groups, aldehydes, and amines, but the conversions to naphthalenes **7i**, **7j**, **7s**, and **7u** were lower. No product formation was observed when the indene substrates contained a free alcohol. Due to the near identical polarity of naphthalenes and their parent indenes, the isolation of analytically pure naphthalenes often required HPLC separation; products **7a**, **7e**, **7m**, and **7n** were successfully isolated by chromatography using AgNO_3_ impregnated silica gel.^36^ We also observed for product 7p* that TBS-deprotection of the crude reaction mixture before chromatography is beneficial; in this way, naphthol 12* is isolated in 45% yields from **6p**.

Our method also offers a straightforward route for the incorporation of ^13^C-labels into naphthalene building blocks when using 1* as the carbon atom source; compounds 7c*, 7e*, 7h*, 7p*, and 7q* were prepared in synthetically useful yields through this pathway (Figure 3E). The incorporation of a heavier carbon isotope in these naphthalenes was confirmed through high-resolution mass spectrometry and NMR analysis. Taking as an example the ^13^C NMR spectrum of 7c*, an intensive doublet of doublets centered at δ = 127.0 ppm is observed, which is caused by the coupling of the ^13^C-nucleus at the 2-position with its directly bonded hydrogen atom (^1^ *J*_CH_ = 159.6 Hz) and with the hydrogen at the 3-position (^2^ *J*_CH_ = 8.4 Hz) (Figure 3F). In the ^1^H NMR spectrum, the proton at the 2-position appears as a doublet of doublets at δ = 7.48 ppm, as a consequence of the coupling with its own carbon (^2^ *J*_CH_ = 159.6 Hz) and with the protons at the 3- and 4-positions (^3^ *J*_HH_ = 6.9 Hz; ^4^ *J*_HH_ = 1.2 Hz), respectively.

Subsequently, the potential use of our skeletal editing protocol for ring expansion of pyrroles into pyridines was evaluated. Soon we realized that the scope of this transformation is limited to 2,5-disubstituted pyrroles of moderate electron richness, which were converted to 2,6-disubstituted pyridines (**10a**–**h**) in modest yields. Thiophene and 3,5-dimethoxybenzene substituents are still tolerated, but the reaction mixtures were complex, and pyridines **10i** and **10j** were obtained in low yields. 2-Phenylpyrrole only delivered traces of the pyridine **10k**, while pyrroles decorated with electron withdrawing groups at the 2-position were *N*-alkylated under the reaction conditions employed and exclusively afforded 1,1-di­(pyrrol-1-yl)­methane products **11a**–**c** (Figure 4).^37^ As expected, ^13^C-labeled pyridines 10a*, 10f* were obtained when 1* was used as the carbon atom source; in the latter case, the C atom insertion occurred on both double bonds of the pyrrole skeleton, showing no regioselectivity.

**Figure 4:**
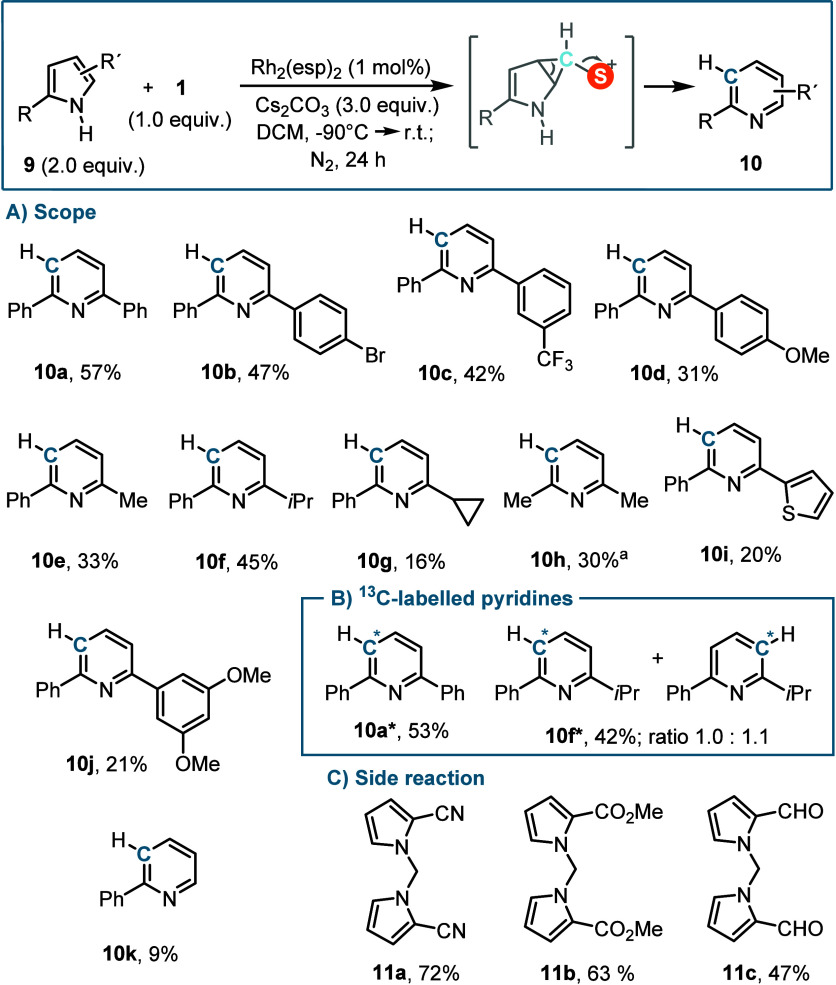
Synthesis of pyridines from pyrroles. (A) Reaction scope of the C-insertion in pyrroles. (B) Synthesis of ^13^C-labeled pyridines via C atom insertion. (C) Formation of 1,1-di­(pyrrol-1-yl)­methane products. Yields are those of the isolated products. ^a 1^H NMR conversions were determined using CH_2_Br_2_ as an internal standard.

The practical utility of reagent 1* for the preparation of ^13^C-labeled drugs has been illustrated through the synthesis of 2-(^13^C)-propranolol, a beta blocker widely used to treat high blood pressure and arrhythmia (Scheme 1).^38^ First, crude 7p* directly obtained from the CH-insertion onto **6p** was deprotected with LiOH to deliver 2-(^13^C)-1-naphthol 12* in 45% yields (two steps). Subsequent reaction with epichlorohydrin under basic conditions affords naphthyl ether 13*, which is finally submitted to epoxide ring opening with isopropylamine. Following this still non-optimized protocol, the desired ^13^C-labeled propranolol 14* was obtained with a global yield of 19% (four steps) from indene **6p** (Scheme 1).

**Scheme 1:**
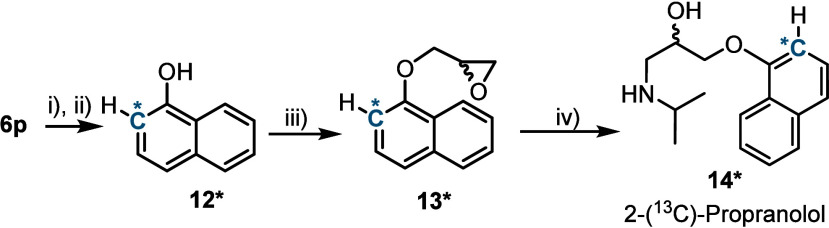
Synthesis of 2-(^13^C)-Propranolol^a^

a Reagents and conditions, yields are in parentheses: (i) 1* (1.0 equiv), Rh_2_(esp)_2_ (1.0 mol %), Cs_2_CO_3_ (3.0 equiv), DCM, −78 °C → r.t. 20 h; (ii) LiOH (4.0 equiv), THF:H_2_O (1:1), r.t. (45%, two steps); (iii) (±)-epichlorohydrin (2.8 equiv), K_2_CO_3_ (3.0 equiv), CH_3_CN, 95 °C, 16 h, (68%); (iv) isopropylamine (2.0 equiv), CaCl_2_ (1.0 equiv), CH_3_CN, 16 h, r.t., (62%).

Finally, considering the limited number of precedents for the formation of Rh-carbene intermediates from sulfur ylides,^39,40^ and the unique cationic character of **1**, leading to the generation of a highly electrophilic α-sulfonio Rh-carbene, we decided to computationally study the mechanism of formation of such intermediates and its subsequent evolution to the naphthalene and pyridine products. The carbene formation and cyclopropanation steps required the explicit modeling of the Rh-catalyst; therefore, the composite electronic structure method r2SCAN-3c was used for geometry optimizations,^41^ and the final energies were computed at the SMD/B3LYP-D3­(BJ)/def2-TZVP level of theory. For intermediates **A** and **B**, the geometry was also reoptimized at the B3LYP-D3­(BJ)/def2-SVP level in order to provide improved bond distances. The final ring expansion step was then fully modeled at the B3LYP-D3­(BJ)/def2-TZVP level (optimizations without solvent corrections, which are added to the final Gibbs energy).^42^ Further computational details are provided in the Supporting Information.

The reaction starts by the coordination of **1** to Rh_2_(esp)_2_ through the π-lone pair centered at carbon forming intermediate **A** (Figure 5A,B). The carbon–rhodium interaction in **A** is relatively weak (Wiberg bond index: 0.39; Mayer bond index: 0.38) and resembles that of protonated carbodiphosphoranes with coinage metals.^43^ At this stage, the Rh-carbene complex **B** is formed from **A** through the elimination of a dibenzothiophene unit, a process that requires 20.3 kcal/mol and clearly mimics the generation of Rh-carbenes from diazocompounds via N_2_-extrusion.^44^ The Rh_1_–C_1_ bond in intermediate **B** significantly shortens when compared with that in **A** (d_Rh–C_ = 2.174 Å in **A**; d_Rh–C_ = 1.912 Å in **B**) supporting the carbene character of this species; consequently, the Rh_1_–C_1_ Wiberg and Mayer bond indices in **B** increase to 1.06 and 1.20, respectively (Figure 5B,C). The LUMO of **B** is mainly localized at the carbene carbon inducing strong electrophilicity at that position (Figure 5C). Compound **B** reacts nearly barrierless with indene to deliver the two possible diasteromers of **C**, which subsequently evolve to the sulfonio-substituted cyclopropanes **D**_**endo**_ and **D**_**exo**_. Our computational investigations suggest that the intrinsic diastereoselectivity of the cyclopropanation is low due to the nearly barrierless progression from **B** to **C**. This would lead to similar amounts of **C**_**endo**_ and **C**_**exo**_ with a low forward barrier to **D**_**endo**_ and **D**_**exo**_, respectively. In these calculations, we have observed a strong impact of dispersion interactions in the overall energetics. The C–C bond formation steps from **B** to **D** are significantly aided by the π–π interaction between the indene, carbene, and dibenzothiophene moieties, with much larger barriers when the D3 correction is removed.

**Figure 5:**
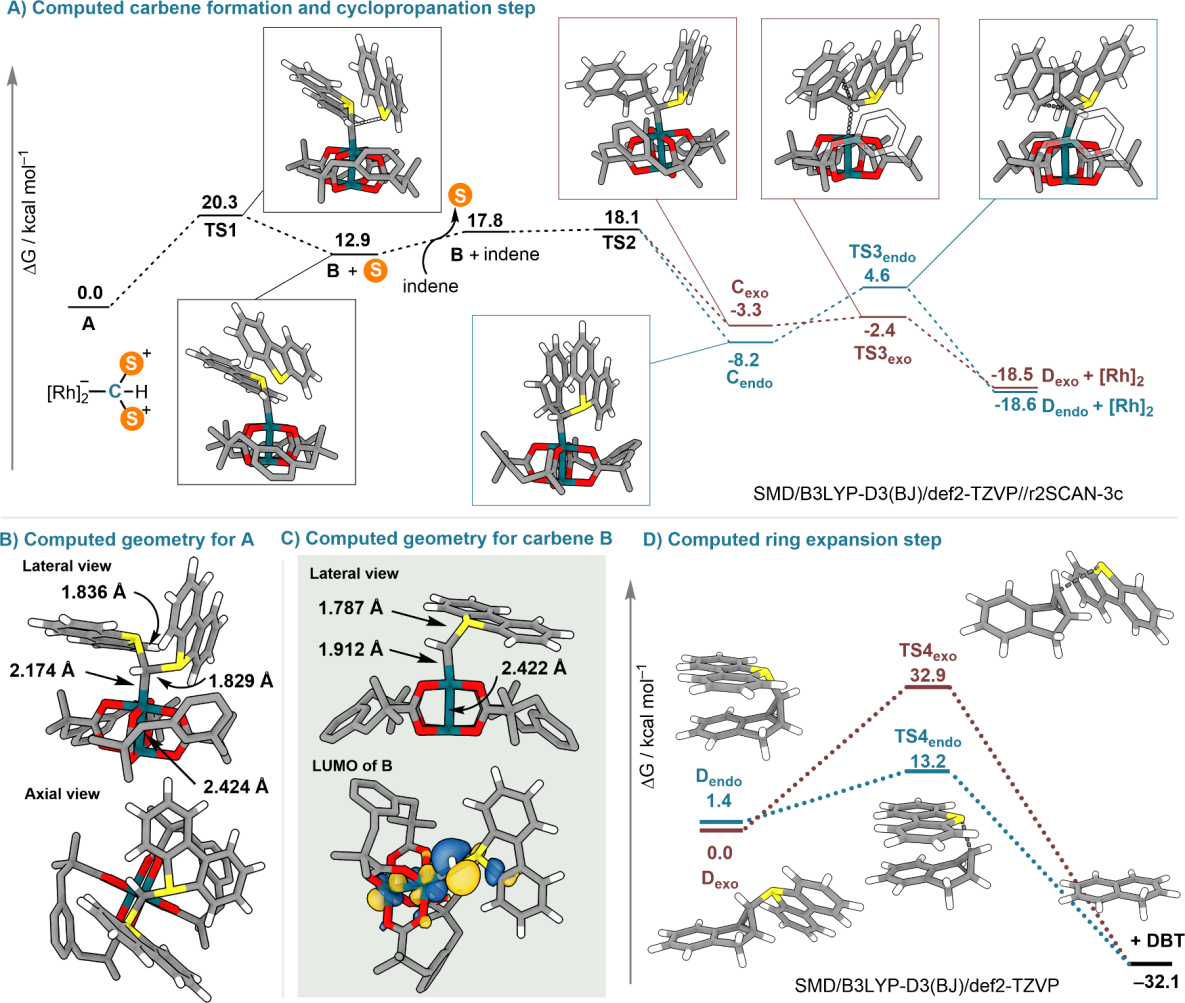
(A) Free energy profile for the Rh-catalyzed formation of cyclopropanes **D**. (B) Computed B3LYP-D3­(BJ)/def2-SVP geometry for intermediate **A**. (C) Computed B3LYP-D3­(BJ)/def2-SVP geometry for carbene intermediate **B**. (D) DFT calculations on the ring expansion of sulfonio-substituted cyclopropanes **D** to naphthalenes.

The computed barrier for ring opening of **D**_**endo**_ is quite low, only 11.8 kcal/mol, which explains why this intermediate is directly transformed to the corresponding naphthalene under the experimental conditions applied. This is not the case for **D**_**exo**_. The formation of naphthalenes from this species is predicted to be prohibitive (32.9 kcal/mol). It is for this reason that **D**_**exo**_ accumulates in the reaction mixture and can be isolated by column chromatography (Figure 5D). Naphthalenes can still be gained from **D**_**exo**_ but only under mild basic conditions that allow its conversion to **D**_**endo**_ via the corresponding S-ylide.

The calculated free energy profile for carbon atom insertion into pyrroles is depicted in Figure S5. Until the formation of cyclopropanes **D**_**endo**_ and **D**_**exo**_, the reaction follows a pathway basically identical to the one just described for indenes; yet the computationally predicted barriers for the final ring expansion step are unexpectedly low when compared with those calculated for indene substrates (Figures 5D and 6A). Even more surprising is that the barrier reduction is more pronounced for the geometrically *forbidden* **TS4**_**exo**_ (10.3 kcal/mol). Building on the analysis reported by Levin for the analogue 6-chloro-2-azabicyclo[3.1.0]­hex-3-ene intermediates,^17c^ this phenomenon can be attributed to the homoaromaticity of the pyrrolic moiety in **TS4**_**endo/exo**_, a stabilizing factor that is not operative for indene substrates. Moreover, the late transition state **TS4**_**exo**_, in which the C–S bond is still basically untouched, specially benefits from this stabilizing interaction, as indicated by the more negative *NICS*(1)_*zz*_ value for its pyrrole fragment (−30.9 ppm) when compared with that in **TS4**_**endo**_ (−25.3 ppm).^45^ It is for this reason that the reaction through the a priori *disallowed* pathway still occurs, and no **D**_**exo**_ cyclopropane intermediate is detected for pyrrole substrates (Figure 6B).

**Figure 6:**
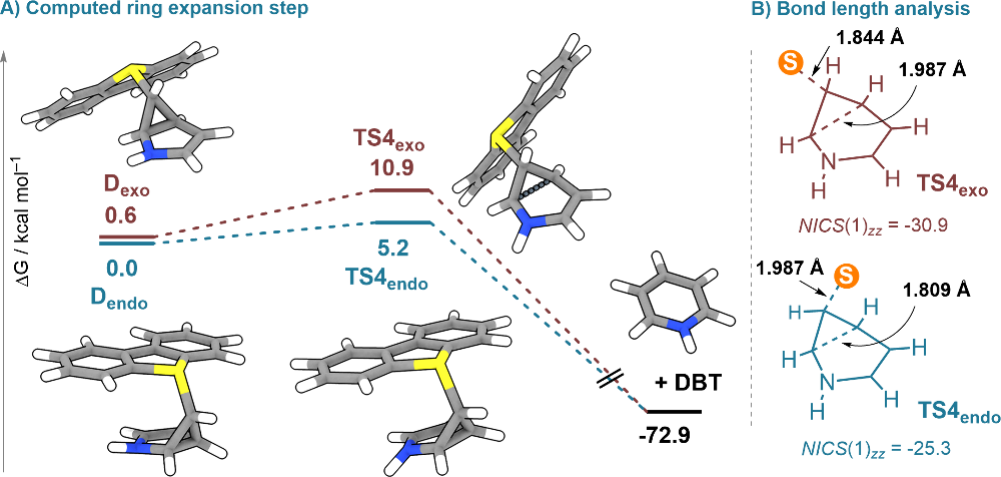
(A) Computed ring expansion of sulfonio-substituted cyclopropanes **D** to pyridines at the SMD/B3LYP-D3­(BJ)/def2-TZVP//r2SCAN-3c level of theory.

## Conclusions

In summary, a Rh-catalyzed protocol has been established, which enables the insertion of the simplest conceivable carbyne cation into indenes and pyrroles to deliver naphthalenes and pyridines. For the achievement of that transformation, the development of salt **1** has been crucial. This reagent is thermally robust because it utilizes an S-ylide as a surrogate of the more commonly used diazo function. For this reason, it does not require additional stabilizing groups attached to carbon to be transferred. The method is particularly suitable for the straightforward preparation of ^13^C-labeled building blocks, which predestines its use for the synthesis of isotopically labeled drug candidates.

## Supplementary Material



## Notes

Experimental procedures and NMR spectra data (PDF)
