# Supplementary material for: A Diazo-free Equivalent of the Unsubstituted Carbyne Cation: Straightforward Synthesis of Naphthalenes and Pyridines via [12/13CH]+ Insertion
Source: J Am Chem Soc. 2026 Jan 19;148(3):3852–61. doi: 10.1021/jacs.5c21901 (PMC12856908; doi:10.1021/jacs.5c21901)
Supplement: Supplementary file 1 [file ja5c21901_si_001.pdf]

SUPPORTING INFORMATION FOR:

A Diazo-free Equivalent of the Unsubstituted Carbyne Cation: Straightforward Synthesis of Naphthalenes and Pyridines via $[^{12/13}CH]^+$ Insertion

Nicola S. Wenzel,[a] Philipp C. Brehm,[a] Maike Mücke,[b] Monish A. Ansari,[a] Brigitte Worbs,[a] Martin Simon,[a] Christopher Golz,[a] Ricardo A. Mata,[b] and Manuel Alcarazo,[a]*

[a]Institut für Organische und Biomolekulare Chemie, Georg-August-Universität Göttingen,

Tammannstr 2, Göttingen 37077, Germany.

[b]Institut für Physikalische Chemie, Georg-August-Universität Göttingen,

Tammannstr 6, Göttingen 37077, Germany.

TABLE OF CONTENTS

TABLE OF CONTENTS 1

MATERIALS AND METHODS 2

PREPARATION OF SILVER NITRATE IMPREGNATED SILICA GEL 4

SYNTHESIS OF SULFONIUM SALTS 5

SYNTHESIS OF INDENE SUBSTRATES 10

General procedure A for the synthesis of substrates **6b, 6f-k, 6q**: 11

General procedure B for the synthesis of indenes **6c-e, 6m**: 15

OPTIMIZATION OF REACTION CONDITIONS: NAPHTHALENES 23

SYNTHESIS OF NAPHTHALENES 25

General procedure C for the synthesis of naphthalenes **7**: 25

SYNTHESIS OF ^{13}C LABELLED NAPHTHALENES 38

SYNTHESIS OF PYRROLE SUBSTRATES 44

OPTIMIZATION OF REACTION CONDITIONS: PYRIDINES 53

SYNTHESIS OF PYRIDINES 54

General procedure E for the synthesis of pyridines **10**: 54

Side reactions for the formation of 1,1-di(pyrrol-1-yl)methane products **11**: 61

SYNTHESIS OF ^{13}C LABELLED PYRIDINES 63

X-RAY CRYSTALLOGRAPHIC ANALYSIS 65

Compound **1**◦MeCN 65

Compound **8a(exo)** 66

SPECTROSCOPIC DATA 67

DFT SUPPLEMENT 137

REFERENCES 211

MATERIALS AND METHODS

Unless stated otherwise, all reactions were carried out using pre-dried glassware under an inert atmosphere (nitrogen or argon) using standard Schlenk techniques, or in a MBraun UNIlab plus glovebox. Dry and degassed solvents (THF, dichloromethane, toluene, diethyl ether, pentane, acetonitrile) were obtained from a MBraun Solvent Purification System (MB-SPS-800) or by distillation over the appropriate drying agent and stored under a protective gas atmosphere. Flash column chromatography was performed on Macherey Nagel 60 (40-63 µm) silica gel. Reactions were controlled by thin-layer chromatography (TLC) analysis, performed using polygram SIL G/UV254 from Macherey Nagel and visualized by UV irradiation (λ = 254 nm), phosphomolybdic acid or iodine stains. Purified compounds were further dried under high vacuum when necessary. Yields refer to spectroscopically pure compounds.

Starting materials: Commercially available reagents were purchased from *Acros Organics*, *ABCR*, *Alfa Aesar*, *BLD Pharmatech*, *Sigma Aldrich* and *TCI*, and used as received.

NMR: Spectra were recorded on Bruker Avance Neo 600, Avance Neo 400, Avance III HD 400, Avance III 400 or Avance III HD 300 spectrometers. ^{1}H and ^{13}C chemical shifts (δ) are reported in ppm relative to TMS, using the solvent signals as reference in $CDCl_3$ (^{1}H: 7.26 ppm, ^{13}C: 77.16 ppm). The chemical shift is depicted in parts per million (ppm), and the coupling constants (*J*) in Hertz (Hz). Data are reported as follows: s = singlet, d = doublet, t = triplet, q = quartet, m = multiplet, br = broad; coupling constants in Hz; integration.

HRMS: Spectra were recorded using *Bruker Daltonik maXis Q-TOF* (ESI), *Bruker Daltonik micrOTOF* (ESI), *Thermo Scientific LTQ Orbitrap XL* (ESI), *Thermo Scientific Exactive GC-Orbitrap-MS* (EI) or *Jeol AccuTOF* (EI) instruments. Dimensionless mass-to-charge ratios (*m*/*z*) are given.

IR: Infrared spectra were recorded on a Jasco FT/IR-4600 spectrometer at room temperature and reported in wavenumbers (cm^{-1}).

Melting point: Melting points were measured with a Büchi M-560 apparatus with a heating rate of 10°C/min.

Single crystal X-ray diffraction analysis: Data collection was done on two dual source equipped *Bruker D8 Venture* four-circle-diffractometer from *Bruker AXS GmbH*. The X-ray sources used were: microfocus *IµS 2.0* Cu/Mo and microfocus *IµS 3.0* Ag/Mo from *Incoatec GmbH* with mirror optics *HELIOS* and single-hole collimator from *Bruker AXS GmbH*. The 3 detectors used were: *Photon III CE14* (Cu/Mo) and *Photon III HE* (Ag/Mo) from *Bruker AXS GmbH*.

APEX6 Suite (v2024.9-1) was employed for data collection together with the therein integrated programs *SAINT* V8.41A (Integration) und *SADABS* 2016/2 (Absorption correction) from *Bruker AXS GmbH*. Structure solution was done with *SHELXT*, refinement with *SHELXL*-2018/3,[1] OLEX^2,[2] and FinalCif were used for data finalization (D. Kratzert, *FinalCif*, *V113*, https://dkratzert.de/finalcif.html).

Special Utilities: A *SMZ1270* stereomicroscope from *Nikon Metrology GmbH* was used for sample preparation; crystals were mounted on *MicroMounts* or *MicroLoops* from *MiTeGen* in NVH oil; crystals were cooled to given temperature with *Cryostream 800* from *Oxford Cryosystems*.

PREPARATION OF SILVER NITRATE IMPREGNATED SILICA GEL

TLC plates coated with silver nitrate impregnated silica gel were prepared following a modified literature procedure:[3],[4] A TLC plate was developed once with an aqueous solution of silver nitrate (1.1 g) in distilled water (20 mL). Then, the TLC plate was allowed to dry, and subsequently, it was activated with a heat gun (2 min) or placed in an oven (120 °C, 15 min). The TLC plates thus prepared could be used as usual but they should be stored under exclusion of light.

The preparation of silver nitrate impregnated silica to be used in typical column chromatography follows a slightly modified literature procedure:[3],[5] The desired weight of silica was measured and transferred into a flask. The desired amount of silver nitrate was calculated and dissolved in acetonitrile. This solution was subsequently added to a suspension of silica gel in acetonitrile. The flask was placed in an ultrasound bath for 5 min to distribute the silver nitrate more homogeneously and all volatile solvents were then removed under reduced pressure on a rotary evaporator until a free-flowing powder was obtained. At this stage, the flask was wrapped in aluminum foil, as the adsorbent becomes sensitive to light. Finally, the silver nitrate impregnated silica gel was placed under high vacuum overnight to complete the drying/activation process. The silver nitrate impregnated silica gel is bench stable, however must be stored under exclusion of light to prevent the silica from turning grey. When running a column with the prepared silica gel over an extended period of time, it is recommended to wrap the column with aluminum foil as well. We always used freshly prepared silver nitrate impregnated silica gel for our columns.

SYNTHESIS OF SULFONIUM SALTS

Synthesis of **4**:

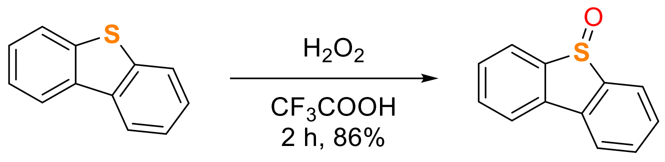

2 **4**

Dibenzo[*b,d*]thiophene-5-oxide **4** was synthesized according to literature procedure.[6] A 250 mL two-necked flask was charged with dibenzo[*b,d*]thiophene **2** (20.0 g, 109 mmol, 1.0 equiv.) and trifluoroacetic acid (80 mL), resulting in a white suspension. To this mixture, H_2O_2 (35% w/w, 9.3 mL, 109 mmol, 1.0 equiv.) was added dropwise by syringe pump over two hours, resulting in a clear solution. The solution was subsequently poured in ice water (700 mL) to form a white suspension. The white solid thus obtained was filtered through a glass frit, washed with cold water, and dried. Finally, the solid was recrystallized from toluene (800 mL), resulting in white needles (18.7 g, 93.3 mmol, 86%).

^{1}H NMR (400 MHz, $CDCl_3$): δ = 8.00 (dt, *J* = 7.7, 0.9 Hz, 2H), 7.82 (dt, *J* = 7.7, 0.9 Hz, 2H), 7.61 (td, *J* = 7.6, 1.2 Hz, 2H), 7.51 (td, *J* = 7.6, 1.1 Hz, 2H) ppm.

^{13}C{^{1}H} NMR (101 MHz, CD_3CN): δ = 145.3, 137.3, 132.7, 129.7, 127.7, 122.1 ppm.

The spectral data were in accordance with those reported in literature.[6]

Synthesis of **3**:

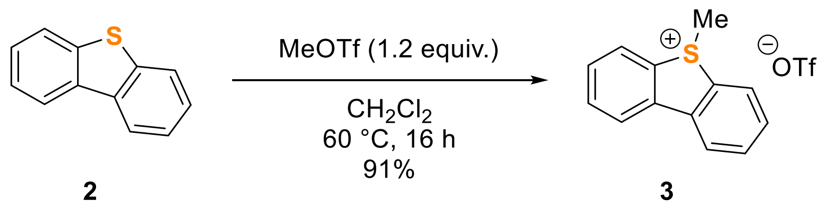

2 **3**

A pressure Schlenk flask equipped with a magnetic stirring bar was charged with dibenzo[*b,d*]thiophene **2** (5.00 g, 27.1 mmol, 1.0 equiv.) and CH_2Cl_2 (4 mL). To this solution, MeOTf (5.34 g, 3.7 mL, 32.6 mmol, 1.2 equiv.) was added dropwise, after which the reaction mixture was heated to 60°C and stirred overnight. Then, the reaction mixture was allowed to cool to room temperature, and the solvent was removed under reduced pressure. The product was thus obtained was washed with diethyl ether (3 × 20 mL) and pentane (1 × 20 mL) to afford **3** as a white solid (8.64 g, 24.8 mmol, 91%).

^{1}H NMR (300 MHz, $CDCl_3$): δ = 8.51 (d, *J* = 8.0 Hz, 2H), 8.11 (d, *J* = 7.6 Hz, 2H), 7.86 (td, *J* = 7.6, 1.1 Hz, 2H), 7.74 (td, *J* = 7.8, 1.3 Hz, 2H), 3.57 (s, 3H) ppm.

^{13}C{^{1}H} NMR (101 MHz, CD_3CN): δ = 140.2, 134.9, 132.0, 131.7, 128.7, 126.9 (CF_3), 125.2, 123.7 (CF_3), 120.5 (CF_3), 117.3 (CF_3), 35.1 ppm.

The spectral data were in accordance with those reported in literature.[7]

Synthesis of **3***:

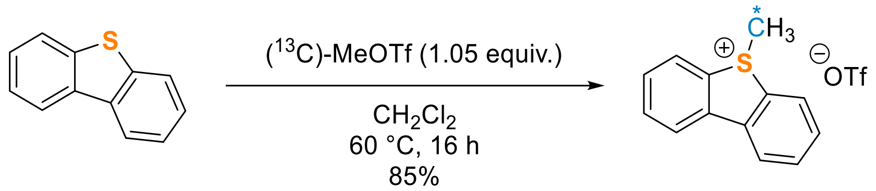

2 **3***

*C = ^{13}C

A pressure Schlenk flask equipped with a magnetic stirring bar was charged with dibenzo[*b*,*d*]thiophene **2** (1.06 g, 5.8 mmol, 1.0 equiv.) and CH_2Cl_2 (0.5 mL). To this solution, H_3**^{13}C**–OTf (1 g, 0.69 mL, 6.1 mmol, 1.05 equiv.) was added, and the reaction mixture was heated to 60°C overnight. Subsequently, the reaction mixture was allowed to cool to room temperature and the solvent was removed under reduced pressure. The solid residue thus obtained was washed with diethyl ether (3 × 10 mL) and pentane (2 × 10 mL) to afford a white solid (1.71 g, 4.9 mmol, 85%).

^{1}H NMR (300 MHz, CD_3CN): δ = 8.35 – 8.18 (m, 4H), 7.99 – 7.86 (m, 2H), 7.83 – 7.69 (m, 2H), 3.32 (d, $^1J_{C,H}$ = 149.3 Hz, 3H) ppm.

^{13}C{^{1}H} NMR (101 MHz, CD_3CN): δ = 35 ppm (observed as minor peaks: 140.3, 134.9, 132.0, 131.7, 128.6, 125.2).

HRMS-ESI(+) (m/z) calcd. for $C_{12}(^{13}C)_1H_{11}S$ [M−OTf]$^+$ 200.0610; found 200.0607.

Synthesis of **5**:

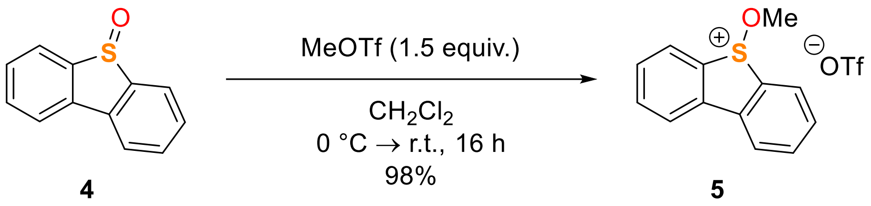

A Schlenk flask equipped with a magnetic stirring bar was charged with dibenzo[*b*,*d*]thiophene 5-oxide **4** (5.00 g, 25.0 mmol, 1.0 equiv.) and CH_2Cl_2 (50 mL). The solution was cooled using an ice bath, and MeOTf (5.15 g, 4.2 mL, 37.5 mmol, 1.5 equiv.) was added dropwise. The reaction mixture stirred overnight while it was allowed to warm to room temperature. Subsequently, the solvent was removed under reduced pressure and the resulting solid washed with dry diethyl ether (3 × 20 mL) to yield an off-white solid (8.90 g, 24.4 mmol, 98%).

^{1}H NMR (300 MHz, CD_3CN): δ = 8.28 (ddd, *J* = 7.9, 1.1, 0.5 Hz, 2H), 8.22 – 8.12 (m, 2H), 7.99 (td, *J* = 7.7, 1.1 Hz, 2H), 7.78 (td, *J* = 7.8, 1.2 Hz, 2H), 3.45 (s, 3H) ppm.

^{13}C{^{1}H} NMR (101 MHz, CD_3CN): δ = 141.9, 138.7, 132.8, 131.8, 129.2, 126.8 (CF_3), 125.3, 123.7 (CF_3), 120.5 (CF_3), 117.3 (CF_3), 58.8 ppm.

^{19}F NMR (377 MHz, $CDCl_3$): δ = −79.34 ppm.

IR (ATR, neat): $\tilde{\nu}$ = 3091, 3070, 3038, 3011, 2946, 2359, 2340, 1586, 1485, 1448, 1426, 1260, 1223, 1142, 1055, 1028, 949, 755, 727, 709, 687, 632, 564, 516, 477, 454, 423 cm^{-1}.

HRMS-ESI(+) (m/z) calcd. for $C_{13}H_{11}OS$ [M-OTf]$^+$: 215.0525; found: 215.0534.

Melting point: 119-120 °C.

Synthesis of **1**:

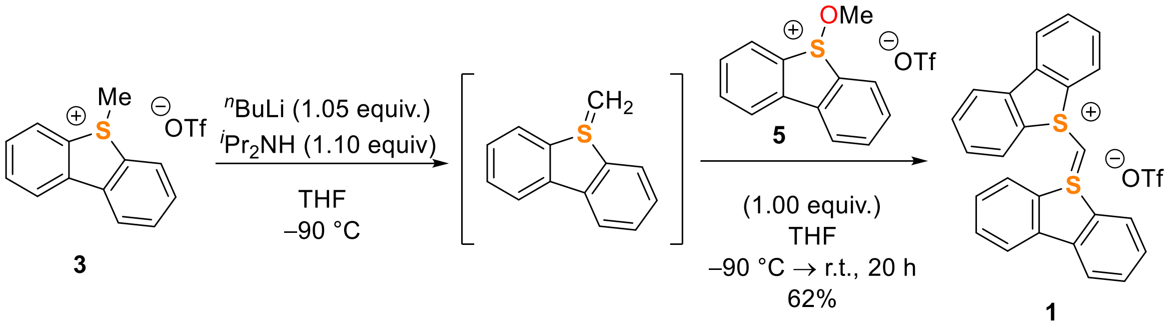

A Schlenk flask equipped with a magnetic stirring bar was charged with sulfonium salt **5** (3.14 g, 8.61 mmol, 1.0 equiv.) and dry THF (150 mL), and was cooled to −90 °C. In a second Schlenk flask, LDA was freshly prepared from diisopropylamine (958.6 mg, 1.35 mL, 9.47 mmol, 1.1 equiv.) and ^{n}BuLi (2.5 M solution in hexanes, 3.6 mL, 9.04 mmol, 1.05 equiv.) in dry THF (50 mL) also at −90 °C. Subsequently, salt **3** (3.00 g, 8.61 mmol, 1.0 equiv.) was added to the solution of LDA in one portion, forming a cloudy yellow suspension. When the yellow suspension became clear, it was cannulated to the flask containing sulfonium salt **5** in THF. During the addition, a white precipitate formed. The reaction mixture thus obtained was slowly allowed to reach room temperature under vigorous stirring during 20 h, after which the white precipitate was filtered and washed with THF (3 × 80 mL), diethyl ether (3 × 80 mL) and pentane (3 × 80 mL). Finally, the obtained residue was dried for 24 h under vacuum, resulting in an off-white solid (2.84 g, 5.35 mmol, 62%).

^{1}H NMR (400 MHz, $CDCl_3$, 70°C): δ = 7.86 (d, J = 7.7 Hz, 4H), 7.81 (d, J = 7.6 Hz, 4H), 7.61 (t, J = 7.5 Hz, 4H), 7.47 (t, J = 7.5 Hz, 4H), 3.30 (s, 1H) ppm.

^{13}C{^{1}H} NMR (101 MHz, $CDCl_3$, 70°C): δ = 137.9, 135.0, 134.8 (CF$_3$), 134.2, 132.2 (CF$_3$), 131.8, 128.0, 127.7 (CF$_3$), 124.9 (CF$_3$), 124.4 ppm.

^{19}F NMR (377 MHz, $CDCl_3$): δ = −80.12 ppm.

IR (ATR, neat): $\tilde{\nu}$ = 3084, 3051, 3011, 2357, 2330, 1707, 1595, 1576, 1480, 1467, 1446, 1426, 1360, 1262, 1219, 1146, 1090, 1065, 1052, 1026, 952, 884, 752, 708, 630, 571, 531, 515, 486, 456, 417 cm^{-1}.

HRMS-ESI(+) (m/z) calcd. for $C_{25}H_{17}S_2$ [M-OTf]$^+$: 381.0766; found: 381.0775.

Melting point: 130 °C decomp.

Synthesis of **1***:

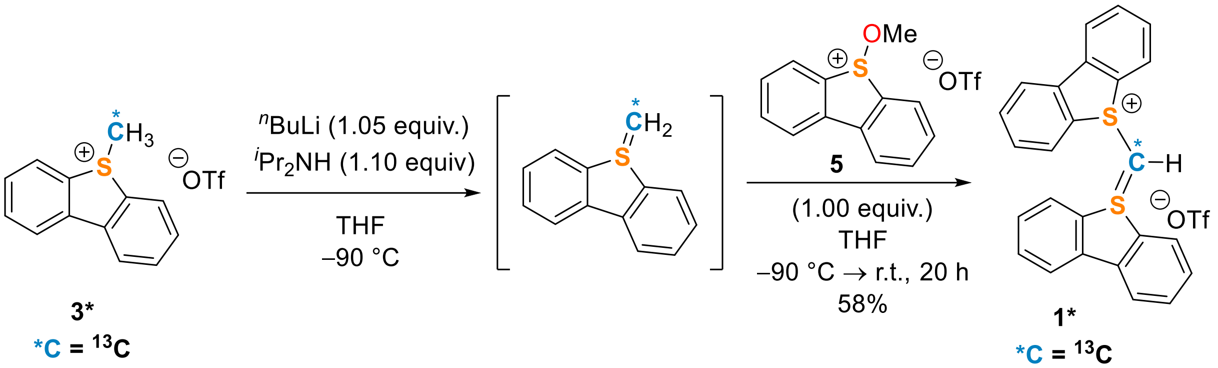

3*
*C = ^{13}C

1*
*C = ^{13}C

Prepared according to the procedure just described but using **3*** (500 mg, 1.4 mmol, 1 eq.) instead of **3**. The product was obtained as an off-white solid (443 mg, 0.83 mmol, 58%).

^{1}H NMR (500 MHz, CD_3CN, 70°C): δ = 7.88 (d_{br}, J = 7.6 Hz, 4H), 7.82 (d_{br}, J = 7.6 Hz, 4H), 7.62 (t_{br}, J = 7.6 Hz, 4H), 7.49 (t_{br}, J = 7.6 Hz, 4H), 3.30 (d, $^1J_{C,H}$ = 207.9 Hz, 1H) ppm.

^{13}C{^{1}H} NMR (126 MHz, CD_3CN, 70°C): δ = **15.2** broad singlet (observed as minor peaks: 137.8, 134.2, 131.7, 128.0, 124.3).

HRMS-ESI(+) (m/z) calcd. for $C_{24}(^{13}C)H_{17}S_2$ [M-OTf]$^+$: 382.0800; found: 382.0798.

SYNTHESIS OF INDENE SUBSTRATES

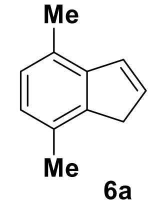
6a

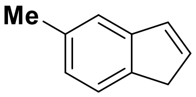
6b

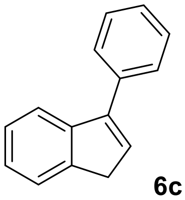
6c

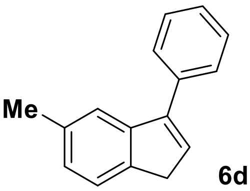
6d

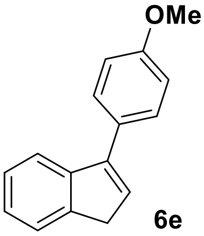
6e

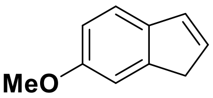
6f

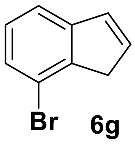
6g

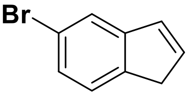
6h

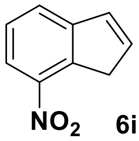
6i

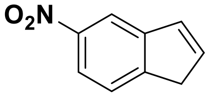
6j

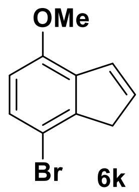
6k

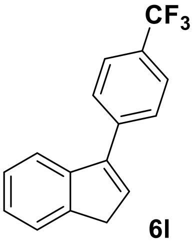
6l

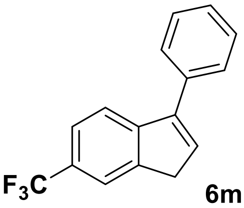
6m

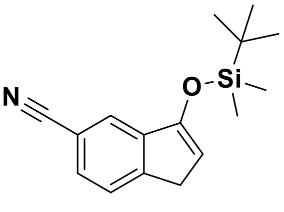
6n

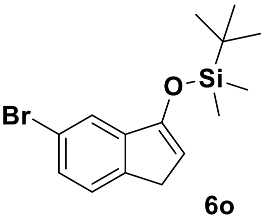
6o

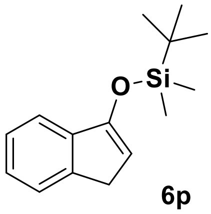
6p

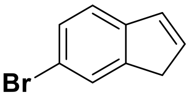
6q

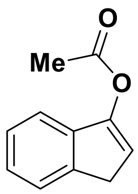
6r

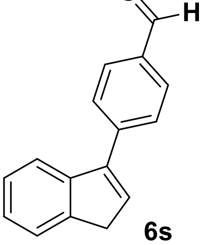
6s

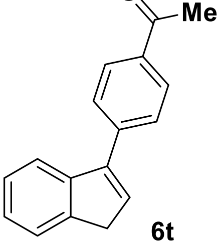
6t

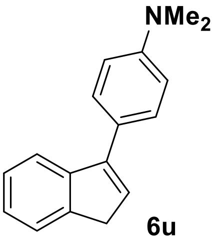
6u

Substrate **6a** was commercially available (BLD Pharm.) and was used as received.

General procedure A for the synthesis of substrates **6b, 6f-k, 6q**:

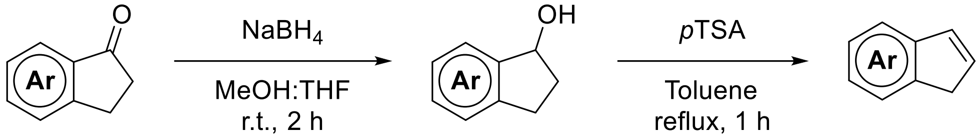

The substrates were synthesized following a modified literature procedure:[8]

A solution of 2,3-dihydro-1*H*-inden-1-one (5.0 mmol, 1.0 equiv.) in MeOH (5 mL) and THF (2.5 mL) was cooled with an ice bath to 0°C. To this solution, $NaBH_4$ (283.7 mg, 7.5 mmol, 1.5 equiv.) was added in one portion. The reaction was stirred at 0 °C for 30 min, after which the ice bath was removed and the reaction was allowed to warm to room temperature. The reaction mixture was stirred for an additional 90 min. Subsequently, the reaction was quenched with water (30 mL), extracted with diethyl ether (3 × 20 mL), and dried over $MgSO_4$. After concentration under reduced pressure, the crude alcohol was used directly without further purification.

In a second step, a solution of the crude alcohol product in toluene (35 mL) was treated with TsOH•H_2O (*p*TSA, 95.11 mg, 0.5 mmol, 0.1 equiv.) and the reaction heated to reflux for 90 min. After cooling to room temperature, an aqueous solution of K_2CO_3 was added (10% aq. K_2CO_3, 40 mL) and the organic phase was washed with brine, dried over $MgSO_4$ and concentrated under reduced pressure. The residue was purified by column chromatography on silica gel using as the eluent the solvent mixture indicated for each case.

Synthesis of **6b**:

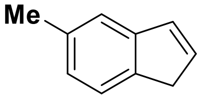

Prepared according to general procedure A from 6-methyl-1-indanone (731.0 mg, 5.00 mmol, 1.0 equiv.). Indene **6b** was purified by column chromatography on silica gel using hexane as the eluent. Colorless oil (466.1 mg, 3.58 mmol, 72%).

^{1}H NMR (400 MHz, $CDCl_3$): δ = 7.37 (d, *J* = 7.5 Hz, 1H), 7.24 (s, 1H), 7.02 (ddt, *J* = 7.6, 1.5, 0.7 Hz, 1H), 6.85 (dtd, *J* = 5.5, 2.0, 0.8 Hz, 1H), 6.55 (dt, *J* = 5.5, 2.0 Hz, 1H), 3.37 (tt, *J* = 2.0, 0.8 Hz, 2H), 2.41 (s, 3H) ppm.

^{13}C{^{1}H} NMR (101 MHz, $CDCl_3$): δ = 145.3, 140.9, 135.9, 134.6, 132.1, 125.5, 123.5, 121.8, 38.8, 21.6 ppm.

The spectral data were in accordance with those reported in literature.[9]

Synthesis of **6f**:

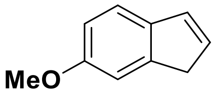

Prepared according to general procedure A from 5-methoxy-1-indanone (810.9 mg, 5.00 mmol, 1.0 equiv.). Indene **6f** was purified by column chromatography on silica gel using hexane as the eluent. Colorless oil (394.2 mg, 2.70 mmol, 54%).

^{1}H NMR (400 MHz, $CDCl_3$): δ = 7.29 (d, *J* = 8.2 Hz, 1H), 7.08 (dp, *J* = 2.3, 0.8 Hz, 1H), 6.88 – 6.78 (m, 2H), 6.42 (dt, *J* = 5.5, 2.0 Hz, 1H), 3.84 (s, 3H), 3.38 (tt, *J* = 2.0, 0.7 Hz, 2H) ppm.

^{13}C{^{1}H} NMR (101 MHz, $CDCl_3$): δ = 158.0, 145.7, 138.1, 132.1, 131.6, 121.3, 112.1, 110.4, 55.7, 39.3 ppm.

The spectral data were in accordance with those reported in literature.[10]

Synthesis of **6g**:

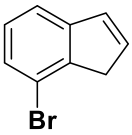

Prepared according to general procedure A from 4-bromo-1-indanone (1.06 g, 5.00 mmol, 1.0 equiv.). Indene **6g** was purified by column chromatography on silica gel using pentane as the eluent. Yellow oil (921.3 mg, 4.72 mmol, 94%).

^{1}H NMR (400 MHz, $CDCl_3$): δ = 7.40 (ddq, *J* = 7.3, 6.5, 0.8 Hz, 2H), 7.06 (t, *J* = 7.7 Hz, 1H), 6.98 (dtd, *J* = 5.6, 1.9, 0.8 Hz, 1H), 6.64 (dt, *J* = 5.6, 2.0 Hz, 1H), 3.50 (tt, *J* = 1.8, 0.8 Hz, 2H) ppm.

^{13}C{^{1}H} NMR (101 MHz, $CDCl_3$): δ = 145.4, 145.1, 135.4, 131.7, 129.6, 126.2, 122.7, 115.4, 40.5 ppm.

The spectral data were in accordance with those reported in literature.[10]

Synthesis of **6h**:

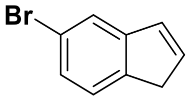

Prepared according to general procedure A from 6-bromo-1-indanone (1.06 g, 5.00 mmol, 1.0 equiv.). Indene **6h** was purified by column chromatography on silica gel using hexane as the eluent. White solid (820.4 mg, 4.21 mmol, 84%).

^{1}H NMR (400 MHz, $CDCl_3$): δ = 7.56 – 7.51 (m, 1H), 7.36 – 7.27 (m, 2H), 6.82 (dt, *J* = 5.7, 2.0 Hz, 1H), 6.60 (dt, *J* = 5.5, 2.0 Hz, 1H), 3.36 (t, *J* = 2.0 Hz, 2H) ppm.

^{13}C{^{1}H} NMR (101 MHz, $CDCl_3$): δ = 147.1, 142.5, 136.1, 131.4, 127.4, 125.1, 124.2, 120.4, 38.9 ppm.

The spectral data were in accordance with those reported in literature.[8]

Synthesis of **6i**:

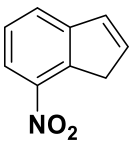

Prepared according to general procedure A from 4-nitro-1-indanone (885.8 mg, 5.00 mmol, 1.0 equiv.). Indene **6i** was purified by column chromatography on silica gel using hexane as the eluent. White solid (346.1 mg, 2.15 mmol, 43%).

^{1}H NMR (400 MHz, $CDCl_3$): δ = 8.06 (dd, *J* = 8.2, 1.0 Hz, 1H), 7.69 (dd, *J* = 7.5, 0.9 Hz, 1H), 7.50 – 7.41 (m, 1H), 6.94 (dt, *J* = 5.6, 1.8 Hz, 1H), 6.76 (dt, *J* = 5.6, 1.9 Hz, 1H), 3.97 – 3.91 (m, 2H) ppm.

^{13}C{^{1}H} NMR (101 MHz, $CDCl_3$): δ = 148.3, 145.3, 139.3, 136.9, 130.8, 127.9, 126.9, 120.2, 41.3 ppm.

IR (ATR, neat): $\tilde{\nu}$ = 3097, 3081, 2898, 2848, 1515, 1453, 1367, 1335, 1308, 1258, 1218, 1165, 1124, 1087, 1060, 948, 916, 841, 833, 810, 767, 744, 694, 619, 595, 555, 491, 462, 403 cm^{-1}.

HRMS-ESI(+) (m/z) calcd. for $C_9H_7NO_2$ $[M+Na]^+$: 184.0369; found: 184.0369.

Melting point: 87 °C.

Synthesis of **6j**:

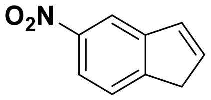

Prepared according to general procedure A from 6-nitro-1-indanone (885.8 mg, 5.00 mmol, 1.0 equiv.). Indene **6j** was purified by column chromatography on silica gel using hexane/EtOAc (100/0→5/1 (v/v)) as the eluent. Off-white solid (651.4 mg, 4.04 mmol, 81%).

^{1}H NMR (400 MHz, $CDCl_3$): δ = 8.23 (d, *J* = 2.1 Hz, 1H), 8.10 (dd, *J* = 8.2, 2.2 Hz, 1H), 7.58 (dt, *J* = 8.2, 0.8 Hz, 1H), 6.95 (dtd, *J* = 5.6, 2.0, 0.8 Hz, 1H), 6.76 (dt, *J* = 5.6, 2.0 Hz, 1H), 3.52 (td, *J* = 2.0, 0.7 Hz, 2H) ppm.

^{13}C{^{1}H} NMR (101 MHz, $CDCl_3$): δ = 150.7, 147.6, 146.1, 137.4, 131.5, 124.0, 120.3, 116.0, 39.4 ppm.

The spectral data were in accordance with those reported in literature.[8]

Synthesis of **6k**:

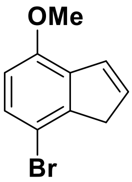

Prepared according to general procedure A from 4-bromo-7-methoxy-1*H*-inden-1-one (1.21 g, 5.00 mmol, 1.0 equiv.). Indene **6k** was purified by column chromatography on silica gel using hexane as the eluent. Colorless solid (306.4 mg, 1.26 mmol, 25%).

^{1}H NMR (400 MHz, $CDCl_3$): δ = 7.26 (d, *J* = 8.5 Hz, 1H), 7.05 (dt, *J* = 5.6, 1.8 Hz, 1H), 6.70 (d, *J* = 8.6 Hz, 1H), 6.51 (dt, *J* = 5.6, 2.0 Hz, 1H), 3.88 (s, 3H), 3.39 (td, *J* = 2.0, 0.6 Hz, 2H) ppm.

^{13}C{^{1}H} NMR (101 MHz, $CDCl_3$): δ = 152.7, 145.4, 134.9, 133.0, 128.6, 128.6, 110.7, 110.1, 55.9, 41.5 ppm.

IR (ATR, neat): $\tilde{\nu}$ = 3078, 3004, 2952, 2928, 2898, 2888, 2832, 1844, 1603, 1577, 1544, 1470, 1437, 1397, 1384, 1357, 1304, 1268, 1218, 1191, 1175, 1106, 1073, 1057, 935, 890, 797, 748, 686, 673, 537, 507, 408 cm^{-1}.

HRMS-EI (m/z) calcd. for $C_{10}H_9BrO$ $[M]^+$: 223.9831; found: 223.9830.

Melting point: 35 °C.

Synthesis of **6q**:

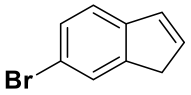

Prepared according to general procedure A from 5-bromo-1-indanone (1.06 g, 5.00 mmol, 1.0 equiv.). Indene **6q** was purified by column chromatography on silica gel using hexane as the eluent and obtained as a white solid (842.9 mg, 4.32 mmol, 86%).

^{1}H NMR (400 MHz, $CDCl_3$): δ = 7.60 (dt, *J* = 1.8, 0.8 Hz, 1H), 7.40 (dd, *J* = 8.0, 1.8 Hz, 1H), 7.26 (d, *J* = 8.0 Hz, 1H), 6.84 (dtd, *J* = 5.6, 2.0, 0.8 Hz, 1H), 6.55 (dt, *J* = 5.5, 2.0 Hz, 1H), 3.39 (td, *J* = 1.9, 0.9 Hz, 2H) ppm.

^{13}C{^{1}H} NMR (101 MHz, $CDCl_3$): δ = 145.9, 143.9, 134.8, 131.6, 129.4, 127.1, 122.2, 118.8, 39.2 ppm.

The spectral data were in accordance with those reported in literature.[8]

General procedure B for the synthesis of indenes **6c-e, 6m**:

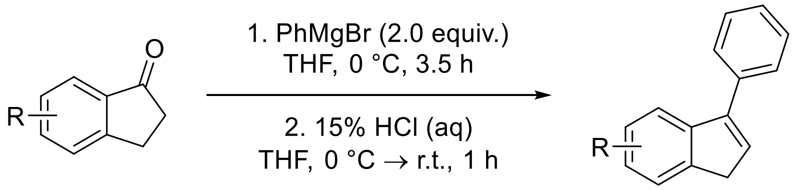

These indene substrates were synthesized following a slightly modified literature procedure:[9] To a Schlenk flask equipped with a magnetic stirring bar, a solution of PhMgBr (10 mmol, 2.0 equiv.) in THF was added, and subsequently diluted with more THF until the concentration was 0.4 M. The thus obtained solution was cooled to 0 °C using an ice-bath, and a solution of the desired 1-indanone (2.0 M) was added dropwise (5.0 mmol, 1.0 equiv.). The reaction mixture was stirred for 3.5 h and then, slowly allowed to warm to room temperature. Finally, the mixture was cooled again to 0 °C using an ice-bath, and quenched with a few drops of a 15% HCl solution. The mixture was subsequently transferred to a separatory funnel and extracted with Et_2O (3 × 50 mL). The combined organic layers were washed with sat. $NaHCO_3$, water and brine, followed by drying over Mg_2SO_4. The solvent was removed under reduced pressure and the crude product was purified by column chromatography on silica gel using the indicated solvent mixtures.

Synthesis of **6c**:

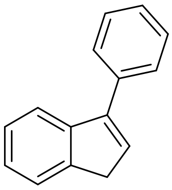

Prepared according to general procedure B from 1-indanone (660.8 mg, 5.0 mmol, 1.0 equiv.). Indene **6c** was purified by column chromatography on silica gel using hexane as the eluent. Colorless oil (809.6 mg, 4.21 mmol, 84%).

^{1}H NMR (400 MHz, $CDCl_3$): δ = 7.61 (ddt, *J* = 7.5, 4.3, 1.2 Hz, 3H), 7.55 (ddt, *J* = 7.3, 1.4, 0.8 Hz, 1H), 7.49 – 7.43 (m, 2H), 7.41 – 7.36 (m, 1H), 7.33 (tdd, *J* = 7.5, 1.3, 0.6 Hz, 1H), 7.27 (td, *J* = 7.4, 1.2 Hz, 1H), 6.60 (t, *J* = 2.2 Hz, 1H), 3.52 (d, *J* = 1.8 Hz, 1H) ppm.

^{13}C{^{1}H} NMR (101 MHz, $CDCl_3$): δ = 145.3, 144.9, 144.1, 136.3, 131.1, 128.7, 127.9, 127.7, 126.3, 125.0, 124.3, 120.5, 38.3 ppm.

The spectral data were in accordance with those reported in literature.[11]

Synthesis of **6d**:

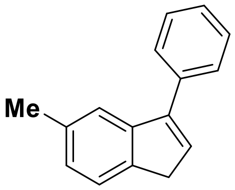

Prepared according to general procedure B from 5-methyl-3-phenyl-1*H*-inden-1-one (730.9 mg, 5.0 mmol, 1.0 equiv.). Indene **6d** was purified by column chromatography on silica gel using hexane/EtOAc (98/2 (v/v)) as the eluent. Colorless oil (656.1 mg, 3.18 mmol, 64%).

^{1}H NMR (400 MHz, $CDCl_3$): δ = 7.64 – 7.57 (m, 2H), 7.51 – 7.34 (m, 5H), 7.13 – 7.06 (m, 1H), 6.57 (t, *J* = 2.2 Hz, 1H), 3.48 (d, *J* = 2.2 Hz, 2H), 2.42 (s, 3H) ppm.

^{13}C{^{1}H} NMR (101 MHz, $CDCl_3$): δ = 145.3, 144.3, 141.9, 136.4, 135.9, 131.4, 128.7, 127.9, 127.7, 125.8, 123.9, 121.1, 38.0, 21.7 ppm.

The spectral data were in accordance with those reported in literature.[12]

Synthesis of **6e**:

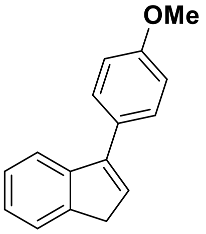

Prepared according to a literature procedure.[13] Obtained as a white solid (509 mg, 2.7 mmol, 61%).

^{1}H NMR (400 MHz, $CDCl_3$): δ = 7.60 – 7.57 (m, 1H), 7.56 – 7.51 (m, 3H), 7.35 – 7.30 (m, 1H), 7.27 – 7.23 (m, 1H), 7.04 – 6.95 (m, 2H), 6.52 (t, *J* = 2.2 Hz, 1H), 3.87 (s, 3H), 3.49 (d, *J* = 2.2 Hz, 2H).

The spectral data were in accordance with those reported in literature.[14]

Synthesis of **6m**:

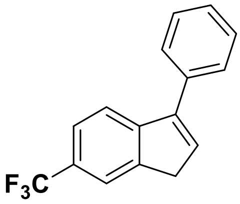

Prepared according to general procedure B from 5-methyl-3-phenyl-1*H*-inden-1-one (1.00 g, 5.0 mmol, 1.0 equiv.). Indene **6m** was purified by column chromatography on silica gel using pentane as the eluent. White solid (564.3 mg, 2.17 mmol, 43%).

^{1}H NMR (400 MHz, $CDCl_3$): δ = 7.78 (dt, *J* = 1.5, 0.8 Hz, 1H), 7.68 – 7.63 (m, 1H), 7.62 – 7.55 (m, 3H), 7.51 – 7.44 (m, 2H), 7.44 – 7.38 (m, 1H), 6.74 (t, *J* = 2.2 Hz, 1H), 3.58 (d, *J* = 2.1 Hz, 2H) ppm.

^{13}C{^{1}H} NMR (101 MHz, $CDCl_3$): δ = 147.4 (d, *J* = 1.4 Hz), 145.1, 145.0, 135.5, 133.8, 128.9, 128.1, 127.8, 127.0 (q, *J* = 30.3 Hz), 123.6, (q, *J* = 271.8 Hz), 124.7 (q, *J* = 3.9 Hz), 121.0 (q, *J* = 3.8 Hz), 120.4, 38.4 ppm.

^{19}F NMR (377 MHz, $CDCl_3$): δ = −61.5 ppm.

The spectral data were in accordance with those reported in literature.[12]

Synthesis of **6l**:

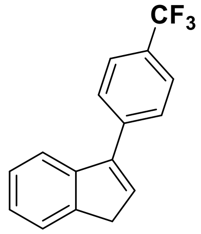

In a 10 mL Schlenk tube 1-iodo-4-(trifluoromethyl)benzene (1.05 g, 0.56 mL, 3.8 mmol, 1 equiv.) was added to a 1.3 M solution of ^{i}PrMgCl-LiCl in THF (2.9 mL, 3.8 mmol, 1 equiv.). After 1.5 hours, 1-indanone (502 mg, 3.8 mmol, 1 equiv.) was added. The reaction was quenched after 3 hours with 1 M aqueous HCl solution (10 mL) and extracted with DCM (3 × 10 mL). The organic phase was washed with water, subsequently dried over $MgSO_4$, and the volatiles were removed under reduced pressure at 45 °C. The crude product was redissolved in 20 mL MeOH, *para*-toluenesulfonic acid (*p*TsOH) monohydrate (75 mg, 0.4 mmol, 0.1 equiv.) was added, and the mixture was heated to 70°C for 3 hours. Finally, the reaction was allowed to cool to room temperature and the solvent was removed under reduced pressure (10 mbar) at 45 °C. Water (10 mL) was added to the residue, and the crude product was extracted with DCM (3 × 10 mL). The organic phase was dried with $MgSO_4$ and the solvent evaporated under reduced pressure to obtain crude **6l**. Column chromatography (SiO_2) using hexane as the eluent delivered **6l** as a white solid (587 mg, 2.3 mmol, 59%).

^{1}H NMR (300 MHz, $CDCl_3$): δ = 7.71 (s, 4H), 7.59 – 7.51 (m, 2H), 7.39 – 7.27 (m, 2H), 6.66 (t, *J* = 2.2 Hz, 1H), 3.55 (d, *J* = 2.2 Hz, 2H).

^{19}F NMR (377 MHz, $CDCl_3$): δ = −62.5 ppm

The spectral data were in accordance with those reported in literature.[10]

Synthesis of **6n**:

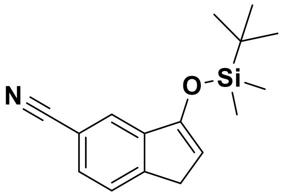

Prepared by following a slightly modified literature procedure.[15] 5-Cyano-1-indanone (443 mg, 2.82 mmol, 1 equiv.) were dissolved in DCM (5 mL) and cooled to 0 °C before NEt_3 (0.5 mL, 3.4 mmol, 1.2 equiv.) was added. The mixture was allowed to stir for 30 minutes, after which *tert*-butylchlorodimethylsilane (TBSCl) (0.45 g, 2.96 mmol, 1.05 equiv.) was added dropwise.

The reaction was stirred for 3 hours at 0 °C and additional 16 hours at room temperature. Then, an aqueous 1 M $NaHCO_3$ solution (9 mL) was added to quench the reaction and the aqueous phase extracted with DCM (3 × 10 mL). The combined organic phases were subsequently dried over Na_2SO_4 and concentrated to dryness. The crude product was purified by flash column chromatography using hexane/EtOAc (97:3) as the eluent. Indene **6n** was obtained as a colorless solid (657 mg, 2.4 mmol, 86%).

^{1}H NMR (400 MHz, $CDCl_3$): δ = 7.62 – 7.60 (m, 1H), 7.50 (dd, *J* = 7.6 Hz, *J* = 1.5 Hz, 1H), 7.45 (dd, *J* = 7.6 Hz, *J* = 0.8 Hz, 1H), 5.52 (t, *J* = 2.4 Hz,1H), 3.34 (d, *J* = 2.4 Hz, 2H), 1.03 (s, 9H), 0.26 (s, 6H) ppm.

^{13}C{^{1}H} NMR (101 MHz, $CDCl_3$): δ = 152.8, 147.7, 143.1, 129.3, 124.6, 121.8, 119.9, 110.1, 107.6, 34.6, 25.8, 18.3, −4.6.

IR (ATR, neat): $\tilde{\nu}$ = 2930, 2892, 2857, 2224, 1597, 1345, 1254, 1191, 1074, 913, 846, 824, 780, 757 cm^{-1}.

HRMS-APCI (m/z) calcd. for $[M+H]^+$: 272.1465; found: 272.1465.

Melting point: 87 °C.

Synthesis of **6o**:

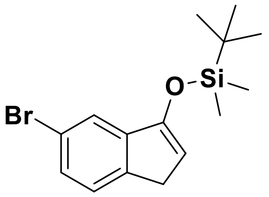

Prepared according to literature[16] and obtained as a colorless liquid (431 mg, 1.3 mmol, 68%).

^{1}H NMR (300 MHz, $CDCl_3$): δ = 7.47 (d, *J* = 1.8 Hz, 1H), 7.32 (dd, *J* = 7.9, 1.9 Hz, 1H), 7.24 (t, *J* = 7.9 Hz, 2H), 5.43 (t, *J* = 2.5 Hz, 1H), 3.22 (d, *J* = 2.4 Hz, 2H), 1.02 (s, 9H), 0.25 (s, 6H) ppm.

The spectral data were in accordance with those reported in literature.[16]

Synthesis of **6p**:

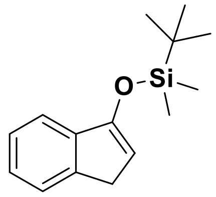

In a 100 mL round-bottom flask, 1-indanone (1.99 g, 15 mmol, 1 equiv.) and *tert*-butylchlorodimethylsilane (TBSCl) (2.67 g, 18 mmol, 1.2 equiv.) were combined and dissolved in toluene (15 mL). The solution was cooled to 0 °C and 1,8-Diazabicyclo[5.4.0]undec-7-ene (DBU) (3.25 mL, 22 mmol, 1.5 equiv.) was added dropwise. The reaction was allowed to stir for 16 hours before Et_2O (10 mL) was added. The organic phase was washed thoroughly with water (3 × 10 mL) and subsequently dried over $MgSO_4$ and concentrated to dryness. The crude orange yellow oil thus obtained was purified by flash column chromatography (SiO_2, conditioned with a 10% NEt_3 hexane solution before addition of the compound) using hexane as the mobile phase. Product **6p** was obtained as a colorless liquid (1.65 g, 6.7 mmol, 45%).

^{1}H NMR (300 MHz, $CDCl_3$): δ = 7.41 – 7.37 (m, 2H), 7.33 – 7.27 (m, 1H), 7.25 – 7.17 (m, 1H), 5.41 (t, *J* = 2.4 Hz, 1H), 3.27 (d, *J* = 2.4 Hz, 2H), 1.03 (s, 9H), 0.25 (s, 6H).

The spectral data were in accordance with those reported in literature.[10]

Synthesis of **6r**:

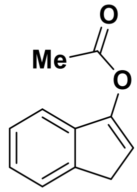

Prepared according to literature procedure.[17] Obtained as a yellow oily liquid (563 mg, 3.2 mmol, 54%).

^{1}H NMR (300 MHz, CDCl3): δ = 7.50 – 7.40 (m, 1H), 7.37 – 7.18 (m, 3H), 6.33 (t, *J* = 2.3 Hz, 1H), 3.42 (d, *J* = 2.3 Hz, 2H), 2.34 (s, 3H).

The spectral data were in accordance with those reported in literature.[18]

Synthesis of **6s**:

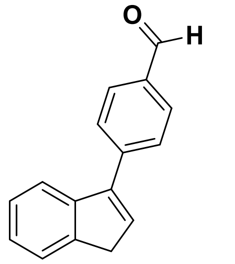

Prepared according to literature procedure.[19] Obtained as a colorless solid (346 mg, 1.6 mmol, 62%).

^{1}H NMR (300 MHz, CDCl3): δ = 10.07 (s, 1H), 7.98 (d, *J* = 8.2 Hz, 2H), 7.78 (d, *J* = 8.2 Hz, 2H), 7.64 – 7.52 (m, 2H), 7.39 – 7.28 (m, 2H), 6.72 (t, *J* = 2.2 Hz, 1H), 3.56 (d, *J* = 2.2 Hz, 2H).

The spectral data were in accordance with those reported in literature.[19]

Synthesis of **6t**:

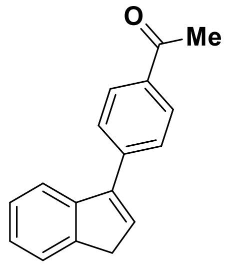

In a Schlenk tube 2-(4-bromophenyl)-2-methyl-1,3-dioxolane (400 mg, 1.6 mmol, 1 equiv.) (prepared according to the literature[20]) were dissolved in 20 mL THF. The solution was cooled to −80 °C before a hexane solution of ^{n}BuLi (2.5 M, 0.66 mL, 1.6 mmol, 1 equiv.) was added. The mixture was allowed to stir at that temperature for 20 minutes upon which 1-indanone (217 mg, 1.6 mmol, 1 equiv.) was added as a solid. The mixture was allowed to warm to room temperature before 0.5 mL concentrated hydrochloric acid was added slowly. The organic phase was washed with brine and the aqueous phase was extracted twice with 10 mL Et_2O. After removal of all volatiles under reduced pressure the crude solid mixture was purified by column chromatography (SiO_2) using hexane/ethylacetate 95:5 as the eluent. The product was obtained as a colourless solid (205 mg, 0.9 mmol, 52%).

^{1}H NMR (400 MHz, $CDCl_3$): δ = 8.13 – 8.07 (m, 2H), 7.92 (m, 2H), 7.88 – 7.83 (m, 1H), 7.64 – 7.59 (m, 2H), 7.58 – 7.49 (m, 2H), 7.49 – 7.42 (m, 2H), 2.69 (s, 2H).

^{13}C{^{1}H} NMR (101 MHz, $CDCl_3$): δ = 198.0, 145.9, 139.1, 136.1, 133.9, 131.3, 130.5, 128.6, 128.5, 128.5, 127.1, 126.5, 126.1, 125.7, 125.5, 26.9.

HRMS-EI (m/z) calcd. for $C_{17}H_{14}O$ $[M]^{\bullet+}$: 234.1039; found: 234.1043.

IR (ATR, neat): $\tilde{\nu}$ = 3042, 2878, 2761, 1676, 1603, 1273, 849, 768, 595 cm^{-1}.

Melting point: 98 °C.

Synthesis of **6u**:

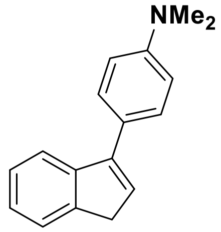

Prepared according to literature procedure.[21] Obtained as an off-white solid in (321 mg, 1.3 mmol, 42% yield).

^{1}H NMR (300 MHz, $CDCl_3$): δ = 7.70 – 7.62 (m, 1H), 7.60 – 7.51 (m, 3H), 7.37 – 7.30 (m, 1H), 7.30 – 7.22 (m, 1H), 6.88 – 6.81 (m, 2H), 6.49 (t, *J* = 2.3 Hz, 1H), 3.50 (d, *J* = 2.2 Hz, 2H), 3.02 (s, 6H).

Spectroscopic data is in accordance with previously reported values.[21]

OPTIMIZATION OF REACTION CONDITIONS: NAPHTHALENES

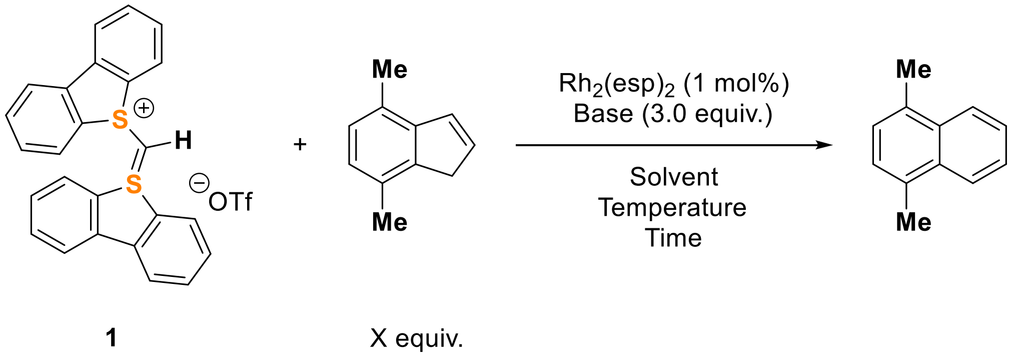

Entry	Equiv. Indene	Base	Solvent	Temperature	Time	Crude yield [%]
1	5	No base	CH_2Cl_2 (0.1 M)	−78 °C → r.t.	20 h	28
2	5	$NaHCO_3$	CH_2Cl_2 (0.1 M)	−78 °C → r.t.	20 h	42
3	5	$NaHCO_3$	CH_2Cl_2 (0.4 M)	−78 °C → r.t.	20 h	41
4	5	$NaHCO_3$	CH_2Cl_2 (0.1 M)	r.t.	20 h	40
5	5	$NaHCO_3$	CH_2Cl_2 (0.1 M)	50 °C	20 h	32
6	5	$NaHCO_3$	CH_2Cl_2 (0.1 M)	80 °C	20 h	traces
7	5	$NaHCO_3$	MeCN (0.1 M)	−40 °C → r.t.	20 h	30
8	5	$NaHCO_3$	CH_2Cl_2 (0.1 M), then MeCN (0.05 M)	−78 °C → r.t., then 110 °C	20 h → r.t., then 4 days	60
9	5	K_3PO_4	CH_2Cl_2 (0.1 M), then MeCN (0.05 M)	−78 °C → r.t., then 110 °C	20 h → r.t., then 4 days	62
10	5	K_3PO_4	$C_2H_4Cl_2$ (0.1 M)	−40 °C → r.t., then 110 °C	20 h → r.t., then 4 days	60 (isolated yield)

Entry	Equiv. Indene	Base	Solvent	Temperature	Time	Crude yield [%]
11	1	K_3PO_4	$C_2H_4Cl_2$ (0.1 M)	−40 °C → r.t., then 110 °C	20 h → r.t., then 4 days	25
12	2	K_3PO_4	$C_2H_4Cl_2$ (0.1 M)	−40 °C → r.t., then 110 °C	20 h → r.t., then 4 days	62 (isolated yield)
13	5	Na_2CO_3	CH_2Cl_2 (0.1 M)	−78 °C → r.t.	20 h	43
14	5	$KHCO_3$	CH_2Cl_2 (0.1 M)	−78 °C → r.t.	20 h	41
15	5	Cs_2CO_3	CH_2Cl_2 (0.1 M)	−78 °C → r.t.	20 h	65
**16**	**2**	**Cs_2CO_3**	**CH_2Cl_2 (0.1 M)**	**−78 °C → r.t.**	**20 h**	**68**
17	2	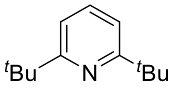	CH_2Cl_2 (0.1 M)	−78 °C → r.t.	20 h	21

Catalyst screening using the conditions of **Entry 16**:

Entry	Catalyst (X mol%)	Crude yield [%]
18	FeTPPCl (1 mol%)	0
19	$AgTp(CF_3)_2$ (1 mol%)	0
20	$Ru_2(esp)_2Cl$ (1 mol%)	traces
21	$Cu(acac)_2$ (5 mol%)	4

SYNTHESIS OF NAPHTHALENES

<u>General procedure C for the synthesis of naphthalenes **7**:</u>

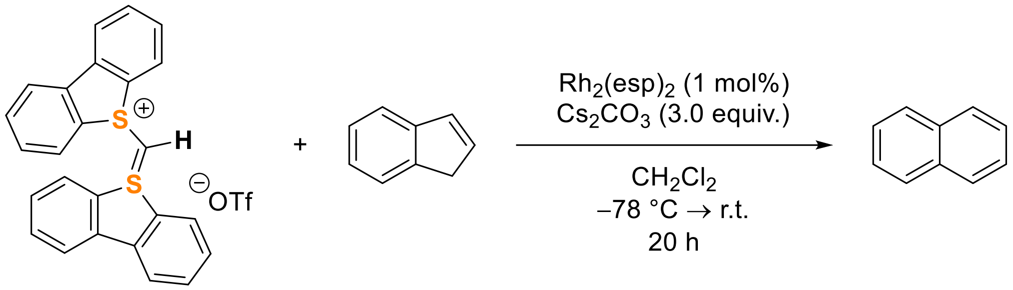

1

A Schlenk flask was charged with Cs_2CO_3 (391 mg, 1.2 mmol, 3.0 equiv.) and $Rh_2(esp)_2$ (3.0 mg, 4.0 µmol, 1 mol%), and was cooled to −78 °C. A solution of the desired indene (0.8 mmol, 2.0 equiv.) in CH_2Cl_2 (4 mL) was subsequently added and the mixture was stirred at this temperature for 10 min. Then, sulfonium salt **1** (212 mg, 0.4 mmol, 1.0 equiv.) was added in one portion and the mixture allowed to slowly reach room temperature under vigorous stirring over 20 h, after which the crude reaction mixture was absorbed onto silica. Depending on the product, the desired naphthalenes were purified by column chromatography using conventional silica gel, HPLC, or column chromatography using $AgNO_3$-doped SiO_2.

Synthesis of **7a**:

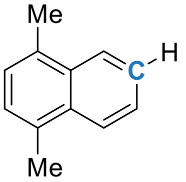

Prepared according to general procedure C from 4,7-dimethyl-1*H*-indene **6a** (115 mg, 0.8 mmol, 2.0 equiv.). The NMR yield of the crude reaction mixture was 68%, referenced against CH_2Br_2 as an internal standard. Naphthalene **7a** was purified by column chromatography using 10% $AgNO_3/SiO_2$ and pentane as the eluent. Colorless oil (38.7 mg, 248 µmol, 62%).

^{1}H NMR (400 MHz, $CDCl_3$): δ = 8.06 – 7.97 (m, 1H), 7.58 – 7.49 (m, 1H), 7.22 (s, 1H), 2.67 (s, 3H) ppm.

^{13}C{^{1}H} NMR (101 MHz, $CDCl_3$): δ = 132.8, 132.5, 126.4, 125.5, 124.8, 19.5 ppm.

HRMS-EI (m/z) calcd. for $C_{12}H_{12}$ $[M]^+$: 155.0855; found: 155.0856.

The spectral data were in accordance with those reported in literature.[22]

Synthesis of **7b**:

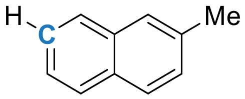

Prepared according to general procedure C from 5-methylindene **6b** (104 mg, 0.8 mmol, 2.0 equiv.). The NMR yield of the crude reaction mixture was 60%, referenced against CH_2Br_2 as an internal standard. Purified by preparative HPLC. Colorless oil (32.5 mg, 229 µmol, 57%).

^{1}H NMR (400 MHz, $CDCl_3$): δ = 7.86 – 7.72 (m, 3H), 7.63 (s, 1H), 7.44 (dddd, *J* = 14.7, 8.2, 6.9, 1.5 Hz, 2H), 7.34 (dd, *J* = 8.4, 1.8 Hz, 1H), 2.54 (s, 3H) ppm.

^{13}C{^{1}H} NMR (101 MHz, $CDCl_3$): δ = 135.6, 133.8, 131.8, 128.3, 127.8, 127.7, 127.4, 127.0, 126.0, 125.1, 21.9 ppm.

HRMS-EI (m/z) calcd. for $C_{11}H_{10}$ $[M]^+$: 142.0777; found: 142.0775.

The spectral data were in accordance with those reported in literature.[23]

Synthesis of **7c**:

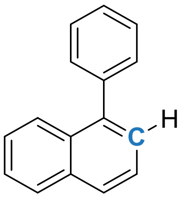

Prepared according to general procedure C from 3-phenylindene **6c** (154 mg, 0.8 mmol, 2.0 equiv.). In this case, pyridine (15.8 mg, 16 µL, 0.2 mmol, 0.5 equiv.) was found to accelerate the reaction. The NMR yield of the crude reaction mixture was 92%, referenced against CH_2Br_2 as an internal standard. Purified by column chromatography using hexane as the eluent. Further purification by preparative HPLC was needed to isolate **7c** analytically pure. Colorless oil (62.9 mg, 308 µmol, 77%).

^{1}H NMR (400 MHz, $CDCl_3$): δ = 7.94 (dd, *J* = 7.8, 1.7 Hz, 2H), 7.89 (dd, *J* = 8.1, 1.2 Hz, 1H), 7.60 – 7.40 (m, 9H) ppm.

^{13}C{^{1}H} NMR (101 MHz, $CDCl_3$): δ = 140.9, 140.4, 133.9, 131.8, 130.2, 130.2, 128.4, 127.8, 127.4, 127.1, 126.2, 126.2, 125.9, 125.5 ppm.

HRMS-EI (m/z) calcd. for $C_{16}H_{12}$ $[M]^+$: 204.0934; found: 204.0936.

The spectral data were in accordance with those reported in literature.[24]

Synthesis of **7d**:

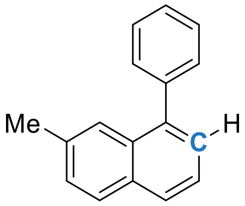

Prepared according to general procedure C from 5-methyl-3-phenyl-1*H*-indene **6d** (165 mg, 0.8 mmol, 2.0 equiv.). Pyridine (15.8 mg, 16 µL, 0.2 mmol, 0.5 equiv.) was found to accelerate the reaction. The NMR yield of the crude reaction mixture was 68%, referenced against CH_2Br_2 as an internal standard. Purified by column chromatography using hexane as the eluent. Further purification by preparative HPLC was needed to isolate **7d** analytically pure. Colorless oil (51.6 mg, 236 µmol, 59%).

^{1}H NMR (400 MHz, $CDCl_3$): δ = 7.86 – 7.78 (m, 2H), 7.66 (dq, *J* = 1.8, 1.0 Hz, 1H), 7.55 – 7.30 (m, 8H), 2.44 (s, 3H) ppm.

^{13}C{^{1}H} NMR (101 MHz, $CDCl_3$): δ = 141.1, 139.7, 135.9, 132.2, 131.9, 130.2, 128.4, 128.3, 128.2, 127.5, 127.3, 127.2, 125.0, 124.6, 22.1 ppm.

HRMS-EI (m/z) calcd. for $C_{17}H_{14}$ $[M]^+$: 218.1090; found: 218.1091.

The spectral data were in accordance with those reported in literature.[25]

Synthesis of **7e**:

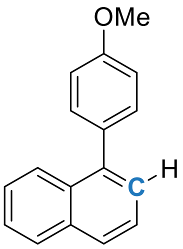

Prepared according to general procedure C from 3-(4-methoxyphenyl)-1*H*-indene **6e** (178 mg, 0.8 mmol, 2.0 equiv.). The NMR yield of the crude reaction mixture was 91%, referenced against CH_2Br_2 as an internal standard. Purified by column chromatography using10%

$AgNO_3/SiO_2$ as stationary phase and hexane as the eluent. Colorless solid (79.0 mg, 337 μmol, 82%).

^{1}H NMR (400 MHz, $CDCl_3$): δ = 7.97 – 7.94 (m, 1H), 7.94 – 7.90 (m, 1H), 7.88 – 7.83 (m, 1H), 7.56 – 7.48 (m, 2H), 7.48 – 7.41 (m, 4H), 7.08 – 7.03 (m, 2H), 3.91 (s, 3H) ppm.

^{13}C{^{1}H} NMR (101 MHz, $CDCl_3$): δ = 159.1, 140.0, 134.0, 133.3, 132.0, 131.25, 128.4, 127.5, 127.0, 126.2, 126.1, 125.8, 125.5, 113.9, 55.5 ppm.

HRMS-EI (m/z) calcd. for $C_{17}H_{14}O$ $[M]^+$: 234.1039; found: 234.1043.

The spectral data were in accordance with those reported in literature.[26]

Synthesis of **7f**:

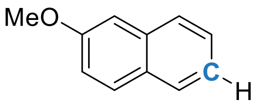

Prepared according to general procedure C from 6-methoxyindene **6f** (117 mg, 0.8 mmol, 2.0 equiv.). The NMR yield of the crude reaction mixture was 84%, referenced against CH_2Br_2 as an internal standard. Purified by column chromatography using hexane/EtOAc (100/0→95/5 (v/v)) as the eluent. Further purification by preparative HPLC was needed to isolate **7f** analytically pure. White solid (41.3 mg, 261 μmol, 65%).

^{1}H NMR (400 MHz, $CDCl_3$): δ 7.81 – 7.71 (m, 3H), 7.44 (ddd, J = 8.1, 6.8, 1.3 Hz, 1H), 7.34 (ddd, J = 8.1, 6.9, 1.2 Hz, 1H), 7.19 – 7.13 (m, 2H), 3.93 (s, 3H) ppm.

^{13}C{^{1}H} NMR (101 MHz, $CDCl_3$): δ = 157.7, 134.7, 129.5, 129.1, 127.8, 126.9, 126.5, 123.7, 118.9, 105.9, 55.4 ppm.

HRMS-EI (m/z) calcd. for $C_{11}H_{10}O$ $[M]^+$: 158.0726; found: 158.0728.

The spectral data were in accordance with those reported in literature.[27]

Synthesis of **7g**:

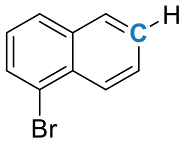

Prepared according to general procedure C from 4-bromoindene **6g** (156 mg, 0.8 mmol, 2.0 equiv.). The NMR yield of the crude reaction mixture was 75%, referenced against CH_2Br_2 as an internal standard. Purified by column chromatography using hexane as the eluent. Further purification by preparative HPLC was needed to isolate **7g** analytically pure. White solid (50.1 mg, 242 µmol, 60%).

^{1}H NMR (300 MHz, $CDCl_3$): δ = 8.25 (dq, *J* = 8.5, 0.9 Hz, 1H), 7.83 (ddt, *J* = 9.5, 8.4, 1.0 Hz, 2H), 7.79 (dd, *J* = 7.4, 1.1 Hz, 1H), 7.60 (ddd, *J* = 8.4, 6.9, 1.4 Hz, 1H), 7.54 (ddd, *J* = 8.1, 6.9, 1.3 Hz, 1H), 7.33 (dd, *J* = 8.2, 7.5 Hz, 1H) ppm.

^{13}C{^{1}H} NMR (101 MHz, $CDCl_3$): δ = 134.8, 132.2, 130.1, 128.5, 128.1, 127.5, 127.3, 126.9, 126.3, 123.0 ppm.

HRMS-EI (m/z) calcd. for $C_{10}H_7Br$ $[M]^+$: 205.9726; found: 205.9723.

The spectral data were in accordance with those reported in literature.[28]

Synthesis of **7h**:

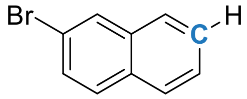

Prepared according to general procedure C from 5-bromoindene **6h** (156 mg, 0.8 mmol, 2.0 equiv.). The NMR yield of the crude reaction mixture was 86%, referenced against CH_2Br_2 as an internal standard. Purified by column chromatography using hexane as the eluent. Further purification by preparative HPLC was needed to isolate **7h** analytically pure. White solid (52.3 mg, 253 µmol, 63%).

^{1}H NMR (400 MHz, $CDCl_3$): δ = 8.01 (d, *J* = 2.0 Hz, 1H), 7.85 – 7.79 (m, 1H), 7.78 – 7.73 (m, 1H), 7.72 (d, *J* = 8.7 Hz, 1H), 7.55 (dd, *J* = 8.7, 2.0 Hz, 1H), 7.52 – 7.45 (m, 2H) ppm.

^{13}C{^{1}H} NMR (101 MHz, $CDCl_3$): δ = 134.6, 132.0, 130.1, 129.7, 129.4, 128.0, 127.1, 127.0, 126.4, 119.9 ppm.

HRMS-EI (m/z) calcd. for $C_{10}H_7Br$ $[M]^+$: 205.9726; found: 205.9723.

The spectral data were in accordance with those reported in literature.[29]

Synthesis of **7i**:

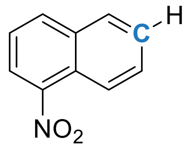

Prepared according to general procedure C from 7-nitroindene **6i** (129 mg, 0.8 mmol, 2.0 equiv.). The NMR yield of the crude reaction mixture was 31%, referenced against CH_2Br_2 as an internal standard. Purified by column chromatography using hexane/EtOAc (100/0→95/5 (v/v)) as the eluent. Yellow solid (19.6 mg, 113 µmol, 28%).

^{1}H NMR (400 MHz, $CDCl_3$): δ = 8.58 (dq, *J* = 8.8, 0.9 Hz, 1H), 8.25 (dd, *J* = 7.6, 1.2 Hz, 1H), 8.13 (dt, *J* = 8.0, 1.1 Hz, 1H), 7.97 (ddt, *J* = 8.3, 1.3, 0.6 Hz, 1H), 7.73 (ddd, *J* = 8.6, 6.9, 1.4 Hz, 1H), 7.63 (ddd, *J* = 8.1, 6.9, 1.2 Hz, 1H), 7.56 (dd, *J* = 8.2, 7.6 Hz, 1H) ppm.

^{13}C{^{1}H} NMR (101 MHz, $CDCl_3$): δ = 146.7, 134.8, 134.5, 129.6, 128.8, 127.5, 125.3, 124.3, 124.2, 123.3 ppm.

HRMS-EI (m/z) calcd. for $C_{10}H_7NO_2$ $[M]^+$: 173.0471; found: 173.0471.

The spectral data were in accordance with those reported in literature.[30]

Synthesis of **7j**:

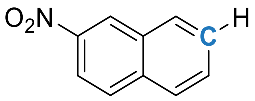

Prepared according to general procedure C from 5-nitroindene **6j** (129 mg, 0.8 mmol, 2.0 equiv.). The NMR yield of the crude reaction mixture was 34%, referenced against CH_2Br_2 as an internal standard. Purified by column chromatography using hexane/EtOAc (100/0→95/5 (v/v)) as the eluent. White solid (21.2 mg, 122 µmol, 31%).

^{1}H NMR (400 MHz, $CDCl_3$): δ = 8.81 (d, *J* = 2.4 Hz, 1H), 8.25 (dd, *J* = 9.1, 2.3 Hz, 1H), 8.14 – 8.01 (m, 1H), 8.00 – 7.92 (m, 2H), 7.68 (dddd, *J* = 21.3, 8.2, 6.9, 1.3 Hz, 2H) ppm.

^{13}C{^{1}H} NMR (101 MHz, $CDCl_3$): δ = 145.7, 136.0, 132.1, 130.2, 129.9, 129.7, 128.2, 128.1, 124.8, 119.4 ppm.

HRMS-ESI(+) (m/z) calcd. for $C_{10}H_7NO_2$ $[M+Na]^+$: 196.0369; found: 196.0368.

The spectral data were in accordance with those reported in literature.[31]

Synthesis of **7k**:

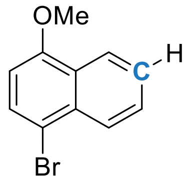

Prepared according to general procedure C from 7-bromo-4-methoxy-1*H*-indene **6k** (180 mg, 0.8 mmol, 2.0 equiv.). The NMR yield of the crude reaction mixture was 35%, referenced against CH_2Br_2 as an internal standard. Purified by column chromatography using hexane as the eluent. Further purification by preparative HPLC was needed to isolate **7k** analytically pure. Yellow oil (24.0 mg, 101 µmol, 25%).

^{1}H NMR (400 MHz, $CDCl_3$): δ = 8.28 (ddd, *J* = 8.3, 1.4, 0.7 Hz, 1H), 8.17 (ddd, *J* = 8.5, 1.3, 0.7 Hz, 1H), 7.66 (d, *J* = 8.2 Hz, 1H), 7.61 (ddd, *J* = 8.4, 6.9, 1.4 Hz, 1H), 7.53 (ddd, *J* = 8.2, 6.9, 1.3 Hz, 1H), 6.69 (d, *J* = 8.3 Hz, 1H), 4.00 (s, 3H) ppm.

^{13}C{^{1}H} NMR (101 MHz, $CDCl_3$): δ = 155.4, 132.6, 129.6, 127.9, 127.0, 126.9, 126.1, 122.6, 113.4, 104.7, 55.8 ppm.

HRMS-EI (m/z) calcd. for $C_{11}H_9BrO$ $[M]^+$: 235.9831; found: 235.9833.

The spectral data were in accordance with those reported in literature.[32]

Synthesis of **7l**:

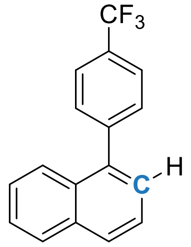

Prepared according to general procedure C from 3-(4-trifluoromethylphenyl)-1*H*-indene **6l** (208 mg, 0.8 mmol, 2.0 equiv.). Adding pyridine (15.8 mg, 16 µL, 0.2 mmol, 0.5 equiv.) was found to accelerate the reaction. The NMR yield of the crude reaction mixture was around 40%, according to ^{19}F-NMR. Purified by column chromatography using hexane as the eluent. Further purification by preparative HPLC was needed to isolate **7l** analytically pure. White solid (25 mg, 92 µmol, 23%).

^{1}H NMR (400 MHz, $CDCl_3$): δ = 7.96 – 7.89 (m, 2H), 7.83 – 7.80 (m, 1H), 7.79 – 7.74 (m, 2H), 7.65 – 7.60 (m, 2H), 7.58 – 7.40 (m, 4H) ppm.

$^{13}C\{^1H\}$ **NMR** (101 MHz, $CDCl_3$): δ = 144.6, 138.9, 133.92 131.4, 130.5, 129.6 (q, *J* =32.6 Hz), 128.6, 128.5, 127.2, 126.2, 125.6, 125.4 (q, *J* = 3.8 Hz), 124.5 (q, *J* = 272.0 Hz) ppm.

^{19}F **NMR** (377 MHz, $CDCl_3$): δ = −62.4 ppm.

HRMS-EI (m/z) calcd. for $C_{17}H_{11}F_3$ $[M]^+$: 272.0807; found: 272.0811.

The spectral data were in accordance with those reported in literature.[33]

Synthesis of **7m**:

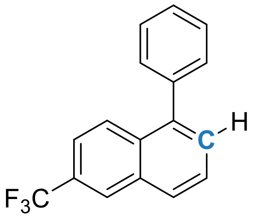

Prepared according to general procedure C from 3-phenyl-6-(trifluoromethyl)-1*H*-indene **6m** (208 mg, 0.8 mmol, 2.0 equiv.) with pyridine (15.8 mg, 16 µL, 0.2 mmol, 0.5 equiv.). The NMR yield of the crude reaction mixture was 66%, referenced against CH_2Br_2 as an internal standard. Purified by column chromatography using hexane as the eluent, followed by isolation using a $AgNO_3$-doped thin layer preparative silica gel plate. Colorless oil (63.5 mg, 233 µmol, 58%).

^1H **NMR** (400 MHz, $CDCl_3$): δ = 8.23 (s, 1H), 8.03 (dq, *J* = 9.0, 1.0 Hz, 1H), 7.96 (dt, *J* = 8.2, 1.2 Hz, 1H), 7.68 – 7.44 (m, 8H) ppm.

$^{13}C\{^1H\}$ **NMR** (101 MHz, $CDCl_3$): δ = 140.6, 140.1, 133.0, 132.8, 130.1, 129.1, 128.6, 127.8, 127.6 (q, *J* = 32.3 Hz), 127.4, 126.9, 126.1 (q, *J* = 4.6 Hz), 123.2 (q, *J* = 272.4 Hz), 121.7 (q, *J* = 3.1 Hz) ppm.

^{19}F **NMR** (377 MHz, $CDCl_3$): δ = −62.3 ppm.

IR (ATR, neat): $\tilde{\nu}$ = 3078, 3057, 3027, 1942, 1886, 1807, 1632, 1598, 1492, 1469, 1445, 1374, 1345, 1307, 1249, 1196, 1171, 1149, 1117, 1069, 1029, 1001, 965, 915, 893, 832, 79, 759, 737, 700, 670, 624, 609, 592, 581, 556, 537, 519, 496, 465, 448, 433, 405 cm^{-1}.

HRMS-EI (m/z) calcd. for $C_{17}H_{11}F_3$ $[M]^+$: 272.0807; found: 272.0809.

Synthesis of **7n**:

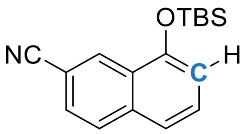

Prepared according to general procedure C from 3-((*tert*-butyldimethylsilyl)oxy)-1*H*-indene-5-carbonitrile **6n** (218 mg, 0.8 mmol, 2.0 equiv.). The NMR yield of the crude reaction mixture was 66%, referenced against CH_2Br_2 as an internal standard. Initially purified by column chromatography using hexane/EtOAc (99:1) as the eluent, followed by isolation using a $AgNO_3$-doped thin layer preparative silica gel plate (same eluent). Colorless oil (69 mg, 240 µmol, 61%).

^{1}**H NMR** (400 MHz, $CDCl_3$): δ = 8.58 – 8.52 (m, 1H), 7.85 (d, *J* = 8.5 Hz, 1H), 7.58 (dd, *J* = 8.5, *J* = 1.7 Hz, 1H), 7.53 – 7.44 (m, 2H), 6.95 (dd, *J* = 6.5, *J* = 2.1 Hz, 1H), 1.10 (s, 9H), 0.32 (s, 6H) ppm.

^{13}**C{^{1}H} NMR** (101 MHz, $CDCl_3$): δ = 152.2, 136.2, 129.7, 129.5, 129.0, 127.1, 126.8, 120.8, 119.9, 114.0, 108.5, 26.0, 18.6, −4.1 ppm.

IR (ATR, neat): $\tilde{\nu}$ = 2953, 2929, 2885, 2858, 2225, 1571, 1454, 1372, 1254, 927, 878, 827, 781, 577 cm^{-1}.

HRMS-ESI (m/z) calcd. for $[M+H]^+$: 284.1465; found: 284.1467.

Synthesis of **7o**:

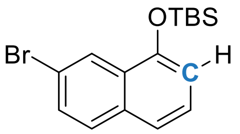

Prepared according to general procedure C from 5-bromo-3-[(1,1-dimethylethyl)dimethylsilyloxyl]-1*H*-indene **6o** (260 mg, 0.8 mmol, 2.0 equiv.). The NMR yield of the crude reaction mixture was 52%, referenced against CH_2Br_2 as an internal standard. Purified by column chromatography using hexane/EtOAc (95:5) as the eluent. Further purification by preparative HPLC was needed to isolate **7o** analytically pure. Yellow oil (41 mg, 104 µmol, 30%).

^{1}**H NMR** (400 MHz, $CDCl_3$): δ = 8.34 (d, *J* = 1.8 Hz, 1H), 7.67 (d, *J* = 8.7 Hz, 1H), 7.54 (dd, *J* = 8.8 Hz, *J* = 2.0 Hz, 1H), 7.42 (d, *J* = 8.3 Hz, 1H), 7.38 – 7.30 (m, 1H), 6.90 (dd, *J* = 7.5 Hz, *J* = 1.1 Hz, 1H), 1.11 (s, 9H), 0.31 (s, 6H) ppm.

$^{13}C\{^1H\}$ NMR (101 MHz, $CDCl_3$): δ = 151.0, 133.5, 129.6, 129.5, 129.2, 126.5, 125.3, 120.9, 119.48, 113.6, 26.0, 18.6, −4.1 ppm.

HRMS-ESI (m/z) calcd. for $C_{16}H_{21}BrOSi$ $[M+H]^+$: 337.0618; found: 337.0617.

The spectral data were in accordance with those reported in literature.[34]

Synthesis of **7r**:

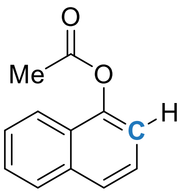

Prepared according to general procedure C from 1*H*-inden-3-yl acetate **6r** (139 mg, 0.8 mmol, 2.0 equiv.). The NMR yield of the crude reaction mixture was 85%, referenced against CH_2Br_2 as an internal standard. Purified by column chromatography using hexane/EtOAc (9:1) as the eluent. Colorless solid (53.6 mg, 288 μmol, 72%).

^1H NMR (400 MHz, $CDCl_3$): δ = 7.92 – 7.85 (m, 2H), 7.78 – 7.72 (m, 1H), 7.53 (ddd, J = 7.6, 3.4, 1.8 Hz, 2H), 7.47 (dd, J = 8.2, 7.5 Hz, 1H), 7.29 – 7.20 (m, 1H), 2.47 (s, 3H).

$^{13}C\{^1H\}$ NMR (101 MHz, $CDCl_3$): δ = 169.6, 146.7, 134.8, 128.2, 126.9, 126.6, 126.2, 125.6, 121.3, 118.2, 21.2.

HRMS-ESI (m/z) calcd. for $C_{12}H_{10}O_2Na$ $[M+Na]^+$: 209.0573; found: 209.0571.

The spectral data were in accordance with those reported in literature.[35]

Synthesis of **7s**:

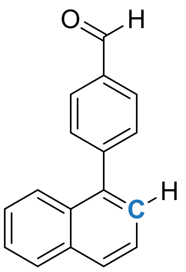

Prepared according to general procedure C from 4-(1*H*-inden-3-yl)benzaldehyde **6s** (176 mg, 0.8 mmol, 2.0 equiv.). The NMR yield of the crude reaction mixture was 40%, referenced against CH_2Br_2 as an internal standard. Purified by column chromatography using

hexane/EtOAc (9:1) as the eluent. Further purification by preparative HPLC was needed to isolate **7s** analytically pure. Colorless solid (27.9 mg, 120 µmol, 30%).

^{1}H NMR (400 MHz, $CDCl_3$): δ = 10.13 (s, 1H), 8.05 – 7.99 (m, 2H), 7.97 – 7.90 (m, 3H), 7.87 – 7.81 (m, 1H), 7.72 – 7.65 (m, 3H), 7.60 – 7.41 (m, 5H).

^{13}C{^{1}H} NMR (101 MHz, $CDCl_3$): δ = 192.2, 147.4, 139.0, 135.5, 133.9, 131.2, 130.9, 129.9, 128.7, 128.6, 127.1, 126.6, 126.2, 125.6, 125.5.

HRMS-EI (m/z) calcd. for $C_{17}H_{12}O$ $[M]^+$: 232.0883; found: 232.0883.

The spectral data were in accordance with those reported in literature.[36]

Synthesis of **7t**:

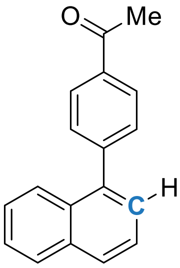

Prepared according to general procedure C from 1-(4-(1*H*-inden-3-yl)phenyl)ethan-1-one **6t** (187 mg, 0.8 mmol, 2.0 equiv.). The NMR yield of the crude reaction mixture was 64%, referenced against CH_2Br_2 as an internal standard. Purified by column chromatography using hexane/EtOAc (9:1) as the eluent. Further purification by preparative HPLC was needed to isolate **7t** analytically pure. Colorless solid (47.3 mg, 192 µmol, 48%).

^{1}H NMR (400 MHz, $CDCl_3$): δ = 8.13 – 8.08 (m, 2H), 7.95 – 7.89 (m, 2H), 7.87 – 7.82 (m, 1H), 7.63 – 7.59 (m, 2H), 7.58 – 7.50 (m, 2H), 7.49 – 7.39 (m, 2H), 2.69 (s, 3H).

^{13}C{^{1}H} NMR (101 MHz, $CDCl_3$): δ = 198.0, 145.9, 139.1, 136.1, 133.9, 131.3, 130.5, 128.6, 128.5, 128.5, 127.1, 126.5, 126.1, 125.7, 125.5, 26.9.

HRMS-EI (m/z) calcd. for $C_{18}H_{14}O_1$ $[M]^+$: 246.1039; found: 246.1041.

The spectral data were in accordance with those reported in literature.[37]

Synthesis of **7u**:

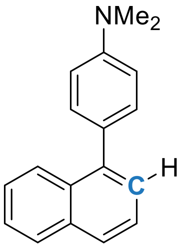

Prepared according to general procedure C from 4-(1*H*-inden-3-yl)-*N*,*N*-dimethylaniline **6u** (188 mg, 0.8 mmol, 2.0 equiv.). The NMR yield of the crude reaction mixture was 28%, referenced against CH_2Br_2 as an internal standard. Purified by column chromatography using hexane/EtOAc (95:5) as the eluent. Further purification by preparative HPLC was needed to isolate **7u** analytically pure. Colorless solid (17.8 mg, 72.0 μmol, 18%).

^{1}H NMR (400 MHz, $CDCl_3$): δ = 8.05 (d, *J* = 8.6 Hz, 1H), 7.95 – 7.89 (m, 1H), 7.83 (dt, *J* = 8.1, 1.2 Hz, 1H), 7.56 – 7.41 (m, 6H), 6.95 – 6.85 (m, 2H), 3.06 (s, 6H).

^{13}C{^{1}H} NMR (101 MHz, $CDCl_3$): δ = 149.9, 140.6, 134.1, 132.1, 131.0, 128.3, 127.0, 126.9, 126.5, 125.8, 125.7, 125.6, 112.5, 40.8.

HRMS-ESI (m/z) calcd. for $C_{18}H_{18}N$ $[M+H]^+$: 248.1434; found: 248.1431.

The spectral data were in accordance with those reported in literature.[38]

Synthesis of ***exo*-8a**:

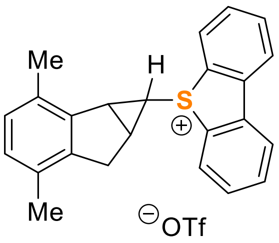

Obtained as a side product in the synthesis of **7a** (on 0.2 mmol scale), when using bases other than Cs_2CO_3. After elution of **7a**, the solvent of the column chromatography was changed to DCM/Acetone (9/1 (v/v)). Compound ***exo*-8a** was obtained as an off-white solid and could be transformed quantitatively into **7a** through stirring with Cs_2CO_3 (3.0 equiv.) at room temperature.

^{1}H NMR (400 MHz, $CDCl_3$): δ = 8.35 – 8.21 (m, 4H), 7.93 (tdd, *J* = 7.8, 1.8, 1.1 Hz, 2H), 7.80 (tt, *J* = 7.7, 1.5 Hz, 2H), 6.85 (s, 2H), 3.37 (ddd, *J* = 7.5, 2.2, 1.3 Hz, 1H), 3.22 (dd, *J* = 17.9, 6.8 Hz, 1H), 3.03 – 2.90 (m, 2H), 2.25 (t, *J* = 2.5 Hz, 1H), 2.20 (s, 3H), 2.05 (s, 3H) ppm.

$^{13}C\{^1H\}$ NMR (101 MHz, $CDCl_3$): δ = 141.1, 140.6, 140.3, 139.8, 135.4, 135.2, 133.3, 132.4, 132.3, 132.2, 131.1, 130.1, 129.8, 129.1, 129.0, 128.9, 125.5, 125.3, 123.8, 120.6, 40.8, 34.9, 31.2, 25.7, 18.8, 18.5 ppm.

^{19}F NMR (377 MHz, $CDCl_3$): δ = −79.3 ppm.

IR (ATR, neat): $\tilde{\nu}$ = 3105, 3081, 3064, 3043, 3041, 2915, 2864, 2359, 2337, 1496, 1482, 1447, 1429, 1381, 1266, 1223, 1165, 1144, 1028, 990, 964, 886, 865, 816, 761, 708, 629, 572, 515, 464, 425 cm^{-1}.

HRMS-ESI(+) (m/z) calcd. for $C_{24}H_{21}S_1$ $[M\text{-}OTf]^+$: 341.1358; found: 341.1370.

Melting point: 171-172 °C.

SYNTHESIS OF ^{13}C LABELLED NAPHTHALENES

Synthesis of **7c***:

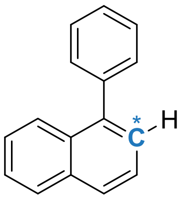

Prepared, as **7c**, from 3-phenylindene **6c** (154 mg, 0.8 mmol, 2.0 equiv.) but using **1*** instead of **1**. The NMR yield of the crude reaction mixture was 95%, referenced against CH_2Br_2 as an internal standard. Purified by column chromatography using hexane as the eluent. Further purification by preparative HPLC was needed to isolate **7c*** analytically pure. Colorless oil (58.3 mg, 284 µmol, 71%).

^{1}H NMR (500 MHz, $CDCl_3$): δ = 7.96 (dddd, *J* = 8.4, 4.5, 1.4, 0.7 Hz, 2H), 7.91 (tt, *J* = 8.4, 1.2 Hz, 1H), 7.64 (dd, *J* = 7.0, 1.3 Hz, 0.5H), 7.59 – 7.51 (m, 6H), 7.51 – 7.45 (m, 2H), 7.32 (dd, *J* = 7.0, 1.2 Hz, 0.5H) ppm.

^{13}C{^{1}H} NMR (101 MHz, $CDCl_3$): δ = 140.9 (d, *J* = 1.7 Hz), 140.4 (d, *J* = 61.5 Hz), 133.9 (d, *J* = 7.4 Hz), 131.8, 130.2 (d, *J* = 2.5 Hz), 128.4 (d, *J* = 1.6 Hz), 127.8 (d, *J* = 2.6 Hz), 127.4, **127.1** (labelled carbon), 126.8, 126.2, 126.2, 125.9 (d, *J* = 1.3 Hz), 125.7 ppm.

^{13}C NMR (126 MHz, $CDCl_3$): δ = **127.1** (d, *J* = 159.3 Hz) ppm.

HRMS-EI (m/z) calcd. for $C_{15}{}^{13}CH_{12}$ $[M]^+$: 205.0967; found: 205.0966.

Synthesis of **7e***:

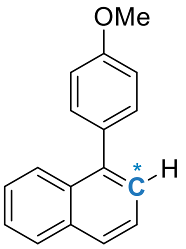

Prepared from 3-(4-methoxyphenyl)-1*H*-indene **6e** (178 mg, 0.8 mmol, 2.0 equiv.) according to general procedure C but using **1*** instead of **1**. The NMR yield of the crude reaction mixture was 81%, referenced against CH_2Br_2 as an internal standard. Purified by column chromatography using hexane/EtOAc (95:5) as the eluent. Further purification by preparative HPLC was needed to isolate **7e*** analytically pure. Colorless solid (47 mg, 200 µmol, 50%).

^{1}H NMR (500 MHz, $CDCl_3$): δ = 8.00 – 7.97 (m, 1H), 7.96 – 7.92 (m, 1H), 7.88 (tt, *J* = 8.3, 1.2 Hz, 1H), 7.61 (ddd, *J* = 159 Hz, 7.1, 1.3 Hz, 1H), 7.57 – 7.50 (m, 2H), 7.49 – 7.45 (m, 3H), 7.10 – 7.05 (m, 2H), 3.93 (s, 3H) ppm.

^{13}C{^{1}H} NMR (126 MHz, $CDCl_3$): δ = 159.1, 140.1 (d, *J* = 62 Hz), 134.0 (d, *J* = 7.5 Hz), 133.2 (d, *J* = 2.0 Hz), 132.0, 131.2 (d, *J* = 2.0 Hz), 128.4 (d, *J* = 2.0 Hz), 127.5 (d, *J* = 3.0 Hz), **127.0** (labelled C-atom) 126.2 (d, *J* = 4.0 Hz), 126.1, 125.8, 125.5 (d, *J* = 53.0 Hz), 113.8, 55.5 ppm.

^{13}C NMR (126 MHz, $CDCl_3$): δ = **127.0** (dd, *J* = 159.0 Hz, 8 Hz) ppm.

HRMS-EI (m/z) calcd. for $[M]^{+}$: 235.1073; found: 235.1073.

Synthesis of **7h***:

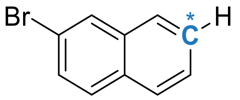

Prepared according to general procedure C from 5-bromoindene **6h** (156 mg, 0.8 mmol, 2.0 equiv.) using **1*** instead of **1** from. The NMR yield of the crude reaction mixture was 88%, referenced against CH_2Br_2 as an internal standard. Purified by column chromatography using hexane as the eluent. Further purification by preparative HPLC was needed to isolate **7h*** analytically pure. White solid (50.1 mg, 241 µmol, 60%).

^{1}H NMR (500 MHz, $CDCl_3$): δ = 8.02 (dt, *J* = 1.3, 0.6 Hz, 1H), 7.82 (tdt, *J* = 8.4, 1.1, 0.6 Hz, 1H), 7.76 (ddq, *J* = 8.2, 1.4, 0.7 Hz, 1H), 7.72 (dd, *J* = 8.8, 0.7 Hz, 1H), 7.67 (ddd, *J* = 8.2, 6.8, 1.2 Hz, 0.5H), 7.56 (dd, *J* = 8.7, 2.0 Hz, 1H), 7.50 (ddt, *J* = 8.2, 6.9, 1.2 Hz, 1H), 7.35 (ddd, *J* = 8.2, 6.9, 1.2 Hz, 0.5H) ppm.

^{13}C NMR (126 MHz, $CDCl_3$): δ = **127.0** (dd, *J* = 6.0, 169.0 Hz) ppm.

HRMS-EI (m/z) calcd. for $C_9{}^{13}CH_7Br$ $[M]^{+}$: 206.9759; found: 206.9759.

Synthesis of **7p***:

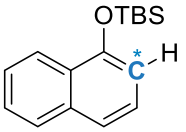

Prepared according to general procedure C from ((1*H*-inden-3-yl)oxy)(*tert*-butyl)dimethylsilane (197 mg, 0.8 mmol, 2.0 equiv.) but using **1*** instead of **1**. The NMR yield of the crude reaction mixture was 60%, referenced against CH_2Br_2 as an internal standard. Purified by column chromatography using hexane as the eluent. Further purification by preparative HPLC was needed to isolate **7p*** analytically pure. Colorless liquid (35 mg, 135 µmol, 33%).

^{1}H NMR (300 MHz, $CDCl_3$): δ = 8.26 – 8.19 (m, 1H), 7.86 – 7.78 (m, 1H), 7.54 – 7.43 (m, 3H), 7.39 – 7.31 (m, 1H), 6.89 (ddd, *J* = 157.3, 7.5, 1.1, 1H), 1.14 (s, 9H), 0.32 (s, 6H) ppm.

^{13}C{^{1}H} NMR (101 MHz, $CDCl_3$): δ = **112.8** ppm.

HRMS-APCI (m/z) calcd. for $[M+H]^+$: 260.1546; found: 260.1548.

Synthesis of **7q***:

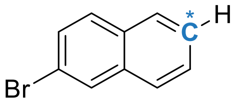

Prepared according to general procedure C using **1*** instead of **1** from 6-bromoindene **6q** (156 mg, 0.8 mmol, 2.0 equiv.). The NMR yield of the crude reaction mixture was 83%, referenced against CH_2Br_2 as an internal standard. Purified by column chromatography using hexane as the eluent. Further purification by preparative HPLC was needed to isolate **7q*** analytically pure. White solid (51 mg, 248 µmol, 62%).

^{1}H NMR (500 MHz, $CDCl_3$): δ = 8.01 (d, *J* = 1.9 Hz, 1H), 7.81 (d, *J* = 8.2 Hz, 1H), 7.76 (td, *J* = 8.4 Hz, 1.1 Hz, 1H), 7.72 (d, *J* = 8.7 Hz, 1H), 7.55 (dd, *J* = 8.7, 2.0 Hz, 1H), 7.51 (ddt, *J* = 8.1, 6.8, 1.2 Hz, 1H), 7.49 (dddd, *J* = 160.5 Hz, 8.1, 6.8, 1.2 Hz, 1H) ppm.

^{13}C{^{1}H} NMR (126 MHz, $CDCl_3$): δ = **126.4** ppm.

HRMS-EI (m/z) calcd. for $C_9{}^{13}CH_7Br$ $[M]^+$: 208.9739; found: 208.9740.

Synthesis of **12***:

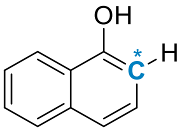

Compound **12*** was obtained from basic deprotection (aqueous solution (4 mL) of LiOH monohydrate (70 mg, 1.7 mmol)) of crude **7p*** (without HPLC purification). The reaction mixture was allowed to stir for 1 hour at room temperature and the reaction progress was monitored by TLC. After completion, crude **12*** was extracted with DCM (3 × 10 mL). The combined organic phases were dried over $MgSO_4$ and concentrated to dryness. The crystalline colorless residue was purified by flash column chromatography using hexane/EtOAc (95:5) as the eluent. Naphthol, **12*** was obtained as a colorless crystalline solid (33 mg, 227 µmol, 45%, two steps).

^{1}H NMR (400 MHz, $CDCl_3$): δ = 8.21 – 8.15 (m, 1H), 7.86 – 7.77 (m, 1H), 7.55 – 7.46 (m, 2H), 7.45 – 7.42 (m, 1H), 7.31 (ddd, *J* = 8.2, 7.4, 1.6 Hz, 2H), 6.82 (ddd, *J* = 156.6, 7.5, 1.0 Hz, 2H), 5.16 (s, 2H) ppm.

^{13}C{^{1}H} NMR (126 MHz, $CDCl_3$): δ = **108.7** ppm.

HRMS-ESI(−) (m/z) calcd. for $C_9{}^{13}CH_7OH$ $[M{-}H]^-$: 144.0536; found: 144.0535.

Synthesis of **13***:

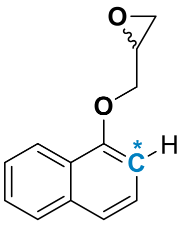

2-(^{13}C)-1-Naphthol **12*** (33 mg, 0.23 mmol, 1 equiv.) was added to a 10 mL round bottom flask and dissolved in 5 mL MeCN. Epichlorohydrine (50 µL, 0.64 mmol, 2.8 equiv.) and K_2CO_3 (0.94 mg, 0.68 mmol, 3 equiv.) were added and the reaction was refluxed at 95 °C for 16 hours. Subsequently, the reaction mixture was concentrated, water (5 mL) was added, and the product was extracted with DCM (3 × 5 mL). Crude **13*** was purified by flash column chromatography (SiO_2) using hexane/EtOAc (95:5 → 90:10) as the eluent. Compound **13*** was obtained as a colorless oil (31 mg, 154 µmol, 68%).

^{1}H NMR (400 MHz, $CDCl_3$): δ = 8.31 (d, *J* = 9.8 Hz, 1H), 7.86 – 7.76 (m, 1H), 7.55 – 7.41 (m, 3H), 7.37 (td, *J* = 8.0, 2.2 Hz, 1H), 6.82 (dd, *J* = 157.6, 7.5 Hz, 1H), 4.41 (dd, *J* = 11.0, 3.2 Hz, 1H), 4.16 (dd, *J* = 11.0, 5.5 Hz, 1H), 3.56 – 3.46 (m, 1H), 2.98 (dd, *J* = 5.0, 4.1 Hz, 1H), 2.86 (dd, *J* = 4.9, 2.6 Hz, 1H) ppm.

^{13}C{^{1}H} NMR (126 MHz, $CDCl_3$): δ = **105.1** (labelled carbon) ppm.

HRMS-APCI (m/z) calcd. for $C_{12}{}^{13}CH_{12}O_2$ $[M+H]^+$: 202.0944; found: 202.0943.

Synthesis of **14***:

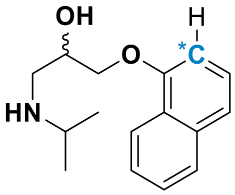

In a pear shaped 5 mL flask, **13*** (31 mg, 0.15 mmol, 1.0 equiv.) was dissolved in 0.15 mL anhydrous MeCN and dried $CaCl_2$ (16.5 mg, 0.15 mmol, 1.0 equiv.) was added. The mixture was stirred until the suspension had homogenized, and then freshly distilled isopropylamine (26 µL, 0.3 mmol, 2.0 equiv.) was added. The reaction mixture was allowed to stir for 16 hours at ambient temperature, before water (3 mL) was added. The product was extracted with DCM (3 × 5 mL) and the combined organic phases combined and dried over $MgSO_4$. Crude **14*** was purified by flash column chromatography (SiO_2) using DCM/MeOH (95:5 → 9:1). White crystalline solid (25 mg, 96 µmol, 62%).

^{1}H NMR (300 MHz, $CDCl_3$): δ = 8.29 – 8.20 (m, 1H), 7.85 – 7.77 (m, 1H), 7.53 – 7.41 (m, 3H), 7.37 (td, *J* = 8.0, 2.3 Hz, 1H), 6.84 (dd, *J*= 157.6, 7.2 Hz, 1H), 4.26 – 4.09 (m, 3H), 3.05 – 2.97 (m, 1H), 2.92 – 2.81 (m, 2H), 2.39 (s, 2H), 1.11 (d, *J* = 6.3 Hz, 6H) ppm.

^{13}C{^{1}H} NMR (126 MHz, $CDCl_3$): δ = 154.5 (d, *J* = 71.7 Hz), 134.6 (d, *J* = 5.5 Hz), 127.7 (d, *J* = 1.5 Hz), 126.6, 126.0 (d, *J* = 55.4 Hz), 125.7 (d, *J* = 3.4 Hz), 125.4, 122.0 (d, *J* = 3.0 Hz), 120.8 (d, *J*= 2.8 Hz), **105.1** (labelled C-atom), 70.8 (d, *J* = 4.5 Hz), 68.7, 49.6, 49.1, 23.4, 23.2 ppm.

^{13}C NMR (126 MHz, $CDCl_3$): δ = **105.1** (ddd, *J* = 158.0, 8.6, 2.0 Hz) ppm.

HRMS-ESI(+) (m/z) calcd. for $C_{15}{}^{13}CH_{21}NO_2$ $[M+H]^+$: 261.1679; found: 261.1676.

SYNTHESIS OF PYRROLE SUBSTRATES

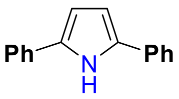

9a

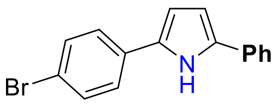

9b

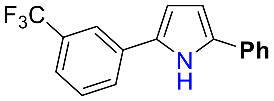

9c

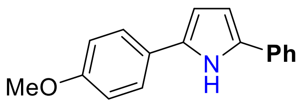

9d

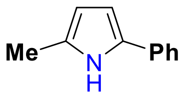

9e

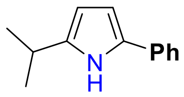

9f

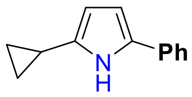

9g

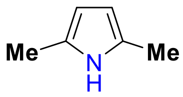

9h

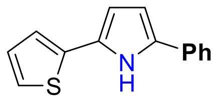

9i

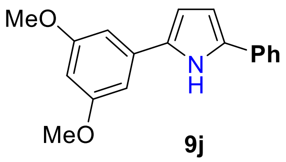

9j

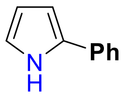

9k

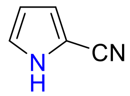

9l

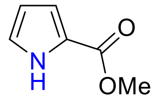

9m

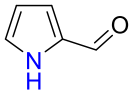

9n

Substrates **9h, 9k, 9l, 9m, 9n** were commercially available (BLD Pharm.) and were used as received. The pyrrole substrates **9a-9g, 9i, 9j** were synthesized following modified literature procedures.

General Procedure D for the synthesis of pyrroles **9a-9g, 9i, 9j**:

1) Elimination:[39]

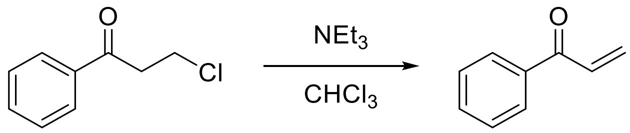

In a 250 mL round-bottom flask, a solution of 3-chloropropiophenone (9.00 g, 53.4 mmol, 1.0 equiv.) in chloroform (100 mL) was cooled to 0 °C. To this solution, triethylamine (13.0 g, 17.8 mL, 128 mmol, 2.4 equiv.) was added dropwise, after which the solution was stirred for 18 h at room temperature. Then, the mixture was washed with 0.1 M HCl (2 × 100 mL), demineralized water (2 × 100 mL), sat. $NaHCO_3$ (2 × 100 mL) and brine (1 × 100 mL). The organic phase was dried over $MgSO_4$ and the solvent was removed under reduced pressure. The resulting phenylvinylketone was a yellow oil (7.01 g, 53.0 mmol, 99%) and was used without further purification.

^{1}H NMR (300 MHz, $CDCl_3$): δ = 8.00 – 7.90 (m, 2H), 7.58 (tt, 1H, *J* = 7.3, 1.3 Hz), 7.55 – 7.42 (m, 2H), 7.16 (dd, *J* = 17.1, 10.6 Hz, 1H), 6.44 (dd, *J* = 17.1, 1.7 Hz, 1H), 5.94 (dd, *J* = 10.6, 1.7 Hz, 1H) ppm.

The spectral data were in accordance with those reported in literature.[39]

2) Stetter Reaction:[39]

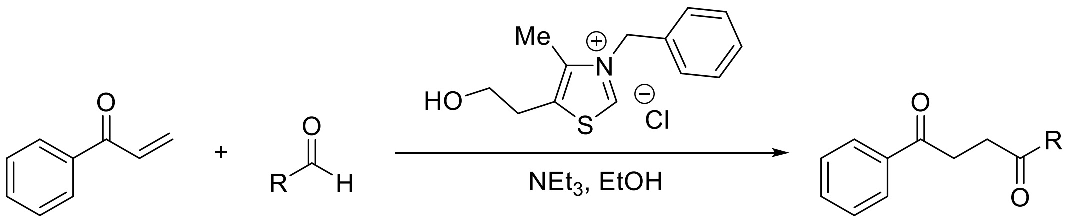

In the second step, a pressure Schlenk was charged with the thiazolium salt (216 mg, 0.8 mmol, 0.1 equiv.), ethanol (4 mL), phenylvinylketone (1.06 g, 8.0 mmol, 1.0 equiv.), triethylamine (486 mg, 0.67 mL, 4.8 mmol, 0.6 equiv.) and the desired aldehyde (8.8 mmol, 1.1 equiv.). The mixture was stirred vigorously at 80 °C for 16 h, after which it was allowed to cool to room temperature. The solvent was removed under reduced pressure. Chloroform was added to the residue, and the mixture was washed with dilute HCl (5%, 1 × 50 mL), sat. $NaHCO_3$ (2 × 50 mL) and demineralized water (1 × 50 mL). The organic phase was dried over $MgSO_4$, filtered and concentrated under reduced pressure. The residue was purified by column chromatography on silica gel using as the eluent the solvent mixture indicated for each case.

Synthesis of **1-(4-bromophenyl)-4-phenylbutane-1,4-dione**:

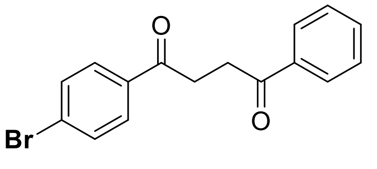

Prepared according to general procedure D from 4-bromobenzaldehyde (1.02 g, 8.8 mmol, 1.1 equiv.). The 1,4-diketone was purified by column chromatography on silica gel using hexane/EtOAc (95/5 (v/v)) as the eluent. White solid (630.8 mg, 2.0 mmol, 40%).

^{1}H NMR (300 MHz, $CDCl_3$): δ = 8.08 – 7.99 (m, 2H), 7.96 – 7.86 (m, 2H), 7.67 – 7.54 (m, 3H), 7.53 – 7.44 (m, 2H), 3.53 – 3.36 (m, 4H) ppm.

The spectral data were in accordance with those reported in literature.[40]

Synthesis of **1-phenyl-4-(3-(trifluoromethyl)phenyl)butane-1,4-dione**:

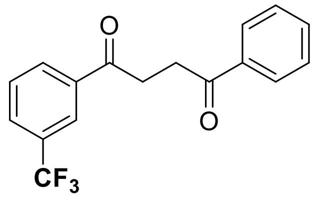

Prepared according to general procedure D from 3-(trifluoromethyl)benzaldehyde (1.53 g, 1.2 mL 8.8 mmol, 1.1 equiv.). The 1,4-diketone was purified by column chromatography on silica gel using hexane/EtOAc (95/5 (v/v)) as the eluent. Colorless solid (1.58 g, 5.2 mmol, 64%).

^{1}H NMR (300 MHz, $CDCl_3$): δ = 8.30 (s, 1H), 8.23 (d, *J* = 7.9 Hz, 1H), 8.10 – 8.00 (m, 2H), 7.84 (d, *J* = 7.8 Hz, 1H), 7.69 – 7.55 (m, 2H), 7.49 (t, *J* = 7.4 Hz, 2H), 3.57 – 3.41 (m, 4H) ppm.

^{19}F NMR (282 MHz, $CDCl_3$): δ = −62.8 ppm.

The spectral data were in accordance with those reported in literature.[41]

Synthesis of **1-(4-methoxyphenyl)-4-phenylbutane-1,4-dione**:

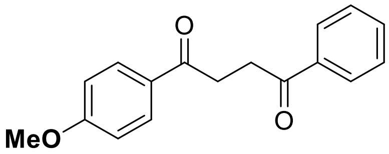

Prepared according to general procedure D from 4-methoxybenzaldehyde (1.20 g, 1.1 mL 8.8 mmol, 1.1 equiv.). The 1,4-diketone was purified by column chromatography on silica gel using hexane/EtOAc (95/5 (v/v)) as the eluent, and was subsequently recrystallized from toluene. Colorless crystals (645.8 mg, 2.4 mmol, 30%).

^{1}H NMR (300 MHz, $CDCl_3$): δ = 8.10 – 7.97 (m, 4H), 7.58 (t, *J* = 7.3 Hz, 1H), 7.48 (t, *J* = 7.4 Hz, 2H), 7.01 – 6.90 (m, 2H), 3.88 (s, 3H), 3.51 – 3.36 (m, 4H) ppm.

The spectral data were in accordance with those reported in literature.[42]

Synthesis of **5-methyl-1-phenylhexane-1,4-dione**:

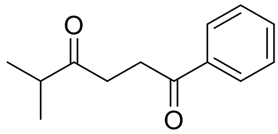

Prepared according to general procedure D from cyclohexanecarbaldehyde (634.5 mg, 8.8 mmol, 1.1 equiv.). The 1,4-diketone was purified by column chromatography on silica gel using hexane/EtOAc (95/5 (v/v)) as the eluent. Colorless oil (984.6 mg, 4.8 mmol, 60%).

^{1}H NMR (400 MHz, $CDCl_3$): δ = 8.02 – 7.93 (m, 2H), 7.55 (t, *J* = 7.3 Hz, 1H), 7.45 (t, *J* = 7.6 Hz, 2H), 3.26 (t, *J* = 6.2 Hz, 2H), 2.90 (t, *J* = 6.4 Hz, 2H), 2.73 (hept, *J* = 7.0 Hz, 1H), 1.17 (s, 3H), 1.16 (s, 3H) ppm.

The spectral data were in accordance with those reported in literature.[42]

Synthesis of **1-cyclopropyl-4-phenylbutane-1,4-dione**:

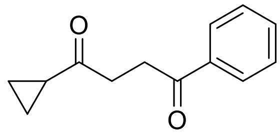

Prepared according to general procedure D from cyclopropanecarbaldehyde (616.8 mg, 8.8 mmol, 1.1 equiv.). The 1,4-diketone was purified by column chromatography on silica gel using hexane/EtOAc (95/5 (v/v)) as the eluent. Yellow oil (929.5 mg, 4.6 mmol, 57%).

^{1}H NMR (300 MHz, $CDCl_3$): δ = 8.04 – 7.94 (m, 2H), 7.56 (t, *J* = 7.3 Hz, 1H), 7.46 (t, *J* = 7.7 Hz, 2H), 3.28 (t, *J* = 6.3 Hz, 2H), 3.04 (t, *J* = 6.7 Hz, 2H), 2.08 – 1.97 (m, 1H), 1.10 – 1.02 (m, 2H), 0.95 – 0.87 (m, 2H) ppm.

The spectral data were in accordance with those reported in literature.[42]

Synthesis of **1-phenyl-4-(thiophen-2-yl)butane-1,4-dione**:

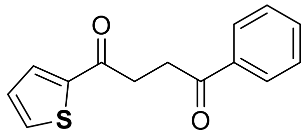

Prepared according to general procedure D from thiophene-2-carbaldehyde (986.9 mg, 8.8 mmol, 1.1 equiv.). The 1,4-diketone was purified by column chromatography on silica gel

using hexane/EtOAc (95/5 (v/v)) as the eluent, and was subsequently recrystallized from toluene. Off-white crystals (882.5 mg, 3.6 mmol, 45%).

^{1}H NMR (300 MHz, $CDCl_3$): δ = 8.07 – 7.99 (m, 2H), 7.84 (dd, *J* = 3.8, 1.2 Hz, 1H), 7.65 (dd, *J* = 5.0, 1.1 Hz, 1H), 7.58 (t, *J* = 7.3 Hz, 1H), 7.48 (t, *J* = 7.8 Hz, 2H), 7.16 (dd, *J* = 4.9, 3.8 Hz, 1H), 3.51 – 3.37 (m, 4H) ppm.

The spectral data were in accordance with those reported in literature.[42]

Synthesis of **1-(3,5-dimethoxyphenyl)-4-phenylbutane-1,4-dione**:

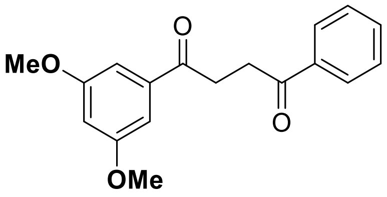

Prepared according to general procedure D from 3,5-dimethoxybenzaldehyde (914.0 mg, 8.8 mmol, 1.1 equiv.). The 1,4-diketone was purified by column chromatography on silica gel using hexane/EtOAc (95/5 (v/v)) as the eluent. Off-white solid (620.0 mg, 2.1 mmol, 42%).

^{1}H NMR (300 MHz, $CDCl_3$): δ = 8.09 – 8.00 (m, 2H), 7.58 (t, *J* = 7.2 Hz, 1H), 7.48 (t, *J* = 7.7 Hz, 2H), 7.18 (d, *J* = 2.3 Hz, 2H), 6.67 (t, *J* = 2.3 Hz, 1H), 3.84 (s, 6H), 3.44 (t, *J* = 2.3 Hz, 4H) ppm.

The spectral data were in accordance with those reported in literature.[43]

3) Paal Knorr Condensation:[33]

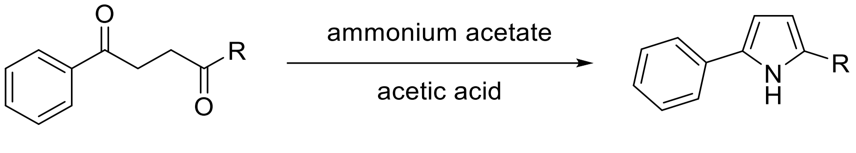

In the third step, a round-bottom flask was charged with the desired 1,4-diketone (1.0 equiv.) and ammonium acetate (6.0 equiv.) in acetic acid. The solution was refluxed for 18 h at 120 °C, after which it was allowed to cool to room temperature. The solution was washed with sat. $NaHCO_3$ (3 × 100 mL), after which the aqueous phases were combined and washed with EtOAc (3 × 50 mL). The combined organic phases were washed with brine (1 × 50 mL), dried over $MgSO_4$, filtered and concentrated under reduced pressure. The resulting pyrrole was used without further purification.

Synthesis of **9a**:

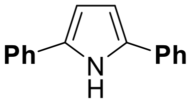

Prepared according to general procedure D from 1,4-diphenylbutane-1,4-dione (1.19 g, 5.0 mmol, 1.0 equiv.), which was commercially available (BLD Pharm.) and was used as received. Brown solid (1.09 g, 5.0 mmol, 99%).

^{1}**H NMR** (300 MHz, $CDCl_3$): δ = 8.58 (s_{br}, 1H), 7.63 – 7.47 (m, 4H), 7.47 – 7.32 (m, 4H), 7.32 – 7.15 (m, 2H), 6.59 (d, *J* = 2.6 Hz, 2H) ppm.

The spectral data were in accordance with those reported in literature.[44]

Synthesis of **9b**:

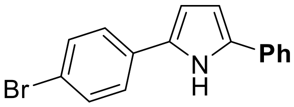

Prepared according to general procedure D from 1-(4-bromophenyl)-4-phenylbutane-1,4-dione (631 g, 2.0 mmol, 1.0 equiv.). Brown solid (493 mg, 1.7 mmol, 83%).

^{1}**H NMR** (400 MHz, DMSO-d_6): δ = 11.31 (s, 1H), 7.75 (ddd, *J* = 10.4, 7.5, 1.7 Hz, 4H), 7.55 (d, *J* = 8.5 Hz, 2H), 7.38 (t, *J* = 7.8 Hz, 2H), 7.20 (t, *J* = 7.3 Hz, 1H), 6.65 (dd, *J* = 3.7, 2.4 Hz, 1H), 6.61 (dd, *J* = 3.7, 2.4 Hz, 1H) ppm.

The spectral data were in accordance with those reported in literature.[45]

Synthesis of **9c**:

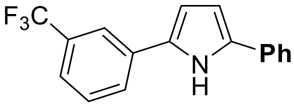

Prepared according to general procedure D from 1-phenyl-4-(3-(trifluoromethyl)phenyl)butane-1,4-dione (1.58 g, 5.2 mmol, 1.0 equiv.). Brown solid (1.43 g, 5.0 mmol, 96%).

^{1}**H NMR** (400 MHz, $CDCl_3$): δ = 8.61 (s_{br}, 1H), 7.74 (s, 1H), 7.69 (d, *J* = 7.3 Hz, 1H), 7.56 (d, *J* = 7.2 Hz, 2H), 7.53 – 7.45 (m, 2H), 7.42 (t, *J* = 7.8 Hz, 2H), 7.27 (t, *J* = 7.3 Hz, 1H), 6.66 (t, *J* = 3.3 Hz, 1H), 6.61 (t, *J* = 2.9 Hz, 1H) ppm.

$^{13}C\{^1H\}$ **NMR** (101 MHz, $CDCl_3$): δ = 134.3, 133.3, 132.3, 131.7, 131.4, 129.6, 129.2, 126.9, 125.6, 124.1, 123.1 – 122.7 (m), 120.4 (q, *J* = 3.4 Hz), 109.3, 108.4 ppm.

^{19}F **NMR** (377 MHz, $CDCl_3$): δ = −62.8 ppm.

IR (ATR, neat): $\tilde{\nu}$ = 3428, 1605, 1517, 1485, 1457, 1412, 1378, 1332, 1308, 1265, 1242, 1183, 1173, 1128, 1102, 1074, 1053, 995, 950, 897, 782, 755, 694, 650, 623, 538, 445, 425 cm^{-1}.

HRMS-ESI(+) (m/z) calcd. for $C_{17}H_{12}F_3N$ $[M+H]^+$: 287.0916; found: 287.0917.

Melting point: 116 °C.

Synthesis of **9d**:

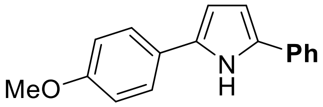

Prepared according to general procedure D from 1-(4-methoxyphenyl)-4-phenylbutane-1,4-dione (646 mg, 2.4 mmol, 1.0 equiv.). Brown solid (588 mg, 2.4 mmol, 98%).

^1H **NMR** (300 MHz, $CDCl_3$): δ = 8.48 (s_{br}, 1H), 7.56 – 7.33 (m, 6H), 7.21 (s_{br}, 1H), 6.94 (d, *J* = 8.8 Hz, 2H), 6.57 (s_{br}, 1H), 6.47 (s_{br}, 1H), 3.84 (s, 3H) ppm.

The spectral data were in accordance with those reported in literature.[46]

Synthesis of **9e**:

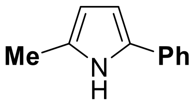

Prepared according to general procedure D from 1-phenyl-1,4-pentanedione (881.1 mg, 5.0 mmol, 1.0 equiv.), which was commercially available (Thermo Fisher Scientific) and was used as received. Brown solid (775 mg, 4.93 mmol, 94%).

^1H **NMR** (300 MHz, $CDCl_3$): δ = 8.10 (s_{br}, 1H), 7.48 – 7.39 (m, 2H), 7.39 – 7.29 (m, 2H), 7.22 – 7.10 (m, 1H), 6.40 (t, *J* = 3.0 Hz, 1H), 5.96 (tt, *J* = 2.6, 0.9 Hz, 1H), 2.34 (s, 3H) ppm.

The spectral data were in accordance with those reported in literature.[47]

Synthesis of **9f**:

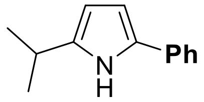

Prepared according to general procedure D from 5-methyl-1-phenylhexane-1,4-dione (1.02 g, 5.0 mmol, 1.0 equiv.). Brown oil (916.4 mg, 4.95 mmol, 99%).

^{1}H NMR (300 MHz, $CDCl_3$): δ = 8.12 (s_{br}, 1H), 7.48 – 7.41 (m, 2H), 7.34 (t, *J* = 7.7 Hz, 2H), 7.17 (t, *J* = 7.3 Hz, 1H), 6.42 (t, *J* = 8.1 Hz, 1H), 5.99 (ddd, *J* = 3.5, 2.6, 0.8 Hz, 1H), 2.98 (hept, *J* = 6.9 Hz, 1H), 1.32 (s, 3H), 1.30 (s, 3H) ppm.

The spectral data were in accordance with those reported in literature.[48]

Synthesis of **9g**:

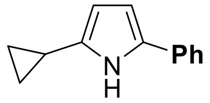

Prepared according to general procedure D from 1-cyclopropyl-4-phenylbutane-1,4-dione (930 mg, 4.6 mmol, 1.0 equiv.). Brown oil (838.2 mg, 4.6 mmol, 99%).

^{1}H NMR (300 MHz, $CDCl_3$): δ = 8.23 (s_{br}, 1H), 7.43 (d, *J* = 7.9 Hz, 2H), 7.33 (t, *J* = 7.5 Hz, 2H), 7.17 (t, *J* = 7.4 Hz, 1H), 6.38 (t, *J* = 3.1 Hz, 1H), 5.90 (t, *J* = 3.1 Hz, 1H), 1.94 – 1.79 (m, 1H), 0.91 – 0.83 (m, 2H), 0.72 – 0.64 (m, 2H) ppm.

The spectral data were in accordance with those reported in literature.[48]

Synthesis of **9i**:

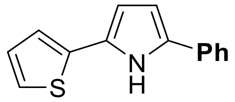

Prepared according to general procedure D from 1-phenyl-4-(thiophen-2-yl)butane-1,4-dione (882.5 mg, 3.6 mmol, 1.0 equiv.). Green solid (807 mg, 3.6 mmol, 99%).

^{1}H NMR (300 MHz, $CDCl_3$): δ = 8.45 (s_{br}, 1H), 7.60 – 7.47 (m, 2H), 7.39 (t, *J* = 7.3 Hz, 2H), 7.28 – 7.20 (m, 1H), 7.18 (dd, *J* = 5.0, 1.2 Hz, 1H), 7.11 (dd, *J* = 3.6, 1.2 Hz, 1H), 7.05 (dd, *J* = 5.0, 3.6 Hz, 1H), 6.55 (dd, *J* = 3.7, 2.6 Hz, 1H), 6.48 (dd, *J* = 3.7, 2.6 Hz, 1H) ppm.

The spectral data were in accordance with those reported in literature.[48]

Synthesis of **9j**:

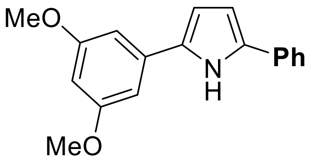

Prepared according to general procedure D from 1-(3,5-dimethoxyphenyl)-4-phenylbutane-1,4-dione (620 mg, 2.1 mmol, 1.0 equiv.). Brown solid (524 mg, 1.9 mmol, 90%).

^{1}H NMR (400 MHz, $CDCl_3$): δ = 8.56 (s_{br}, 1H), 7.57 – 7.49 (m, 2H), 7.39 (t, J = 7.6 Hz, 2H), 7.23 (t, J = 7.4 Hz, 1H), 6.68 (d, J = 2.2 Hz, 2H), 6.57 (d, J = 2.6 Hz, 2H), 6.37 (t, J = 2.2 Hz, 1H), 3.85 (s, 6H) ppm.

^{13}C{^{1}H} NMR (101 MHz, $CDCl_3$): δ = 161.4, 134.6, 133.3, 133.2, 132.6, 129.1, 126.6, 124.0, 108.5, 108.0, 102.5, 98.5, 55.6 ppm.

IR (ATR, neat): $\tilde{\nu}$ = 3425, 2935, 2834, 1721, 1590, 1515, 1472, 1454, 1431, 1373, 1353, 1301, 1249, 1226, 1203, 1152, 1062, 1042, 990, 925, 905, 828, 804, 784, 755, 692, 657, 6330, 584, 535, 461, 443 cm^{-1}.

HRMS-ESI(+) (m/z) calcd. for $C_{18}H_{18}NO_2$ $[M+H]^+$: 280.1332; found: 280.1330.

Melting point: 95 °C.

OPTIMIZATION OF REACTION CONDITIONS: PYRIDINES

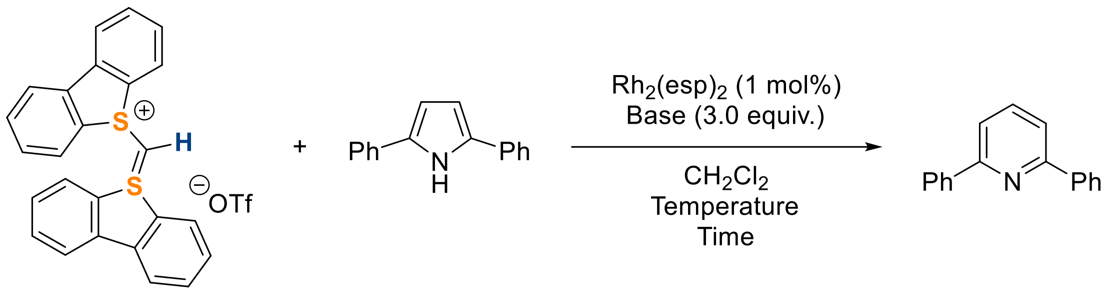

1 (2.0 equiv.)

Entry	Base	Temperature	Time	Crude yield [%]
**1**	**Cs_2CO_3**	**−90 °C → r.t.**	**24 h**	**62 (isolated 57)**
2	Cs_2CO_3	r.t.	2 h<br>24 h	After 2 h: 31%<br>After 24 h: 37%
3	Cs_2CO_3	−30 °C →<br>−10 °C	24 h<br>43 h	After 24 h: 25%<br>After 43 h: 30%
4[a]	Cs_2CO_3	−30 °C → r.t.	20 h	22
5	No base	−90 °C → r.t.	24 h	0
6	K_3PO_4	−90 °C → r.t.	24 h	60

[a] 1 equiv. pyrrole, 2 equiv. of **1**. **1** added portionwise every 30 min.

SYNTHESIS OF PYRIDINES

General procedure E for the synthesis of pyridines **10**:

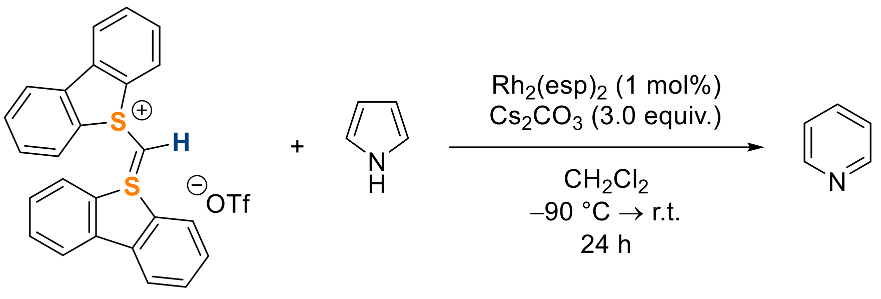

1

A Schlenk flask was charged with Cs_2CO_3 (391 mg, 1.2 mmol, 3.0 equiv.) and $Rh_2(esp)_2$ (3.0 mg, 4.0 µmol, 1 mol%), and was cooled to −90 °C. A solution of the desired pyrrole (0.8 mmol, 2.0 equiv.) in CH_2Cl_2 (4 mL) was subsequently added and the mixture was stirred at this temperature for 10 min. Then, sulfonium salt **1** (212 mg, 0.4 mmol, 1.0 equiv.) was added in one portion and the mixture allowed to slowly reach room temperature under vigorous stirring over 24 h. After reaction completion, the solvent was removed under reduced pressure and the residue was diluted with EtOAc. The residue was washed with brine (3 × 10 mL), and the combined aqueous phases were washed with EtOAc (3 × 10 mL). The combined organic phases were dried over $MgSO_4$ and filtered. The solvent was removed under reduced pressure and the crude product was purified by column chromatography on silica gel using the indicated solvent mixtures.

Synthesis of **10a**:

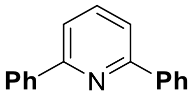

Prepared according to general procedure E from 2,5-diphenyl-1*H*-pyrrole **9a** (175 mg, 0.8 mmol, 2.0 equiv.). The NMR yield of the crude reaction mixture was 62%, referenced against CH_2Br_2 as an internal standard. Purified by column chromatography using hexane/EtOAc (100/0→95/5 (v/v)) as the eluent. White solid (52.4 mg, 227 µmol, 57%).

^{1}H NMR (400 MHz, $CDCl_3$): δ = 8.23 – 8.12 (m, 4H), 7.82 (dd, *J* = 8.4, 7.1 Hz, 1H), 7.74 – 7.67 (m, 2H), 7.58 – 7.47 (m, 4H), 7.47 – 7.40 (m, 2H) ppm.

^{13}C{^{1}H} NMR (101 MHz, $CDCl_3$): δ = 157.0, 139.6, 137.6, 129.1, 128.8, 127.1, 118.8 ppm.

HRMS-ESI(+) (m/z) calcd. for $C_{17}H_{14}N$ $[M+H]^+$: 232.1121; found: 232.1118.

The spectral data were in accordance with those reported in literature.[49]

Synthesis of **10b**:

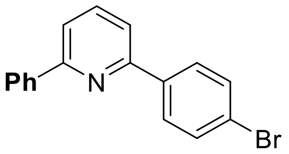

Prepared according to general procedure E from 2-(4-bromophenyl)-5-phenyl-1*H*-pyrrole **9b** (239 mg, 0.8 mmol, 2.0 equiv.). The NMR yield of the crude reaction mixture was 50%, referenced against CH_2Br_2 as an internal standard. Purified by column chromatography using hexane/EtOAc (100/0→95/5 (v/v)) as the eluent. Colorless solid (57.8 mg, 186 µmol, 47%).

^{1}H NMR (400 MHz, $CDCl_3$): δ = 8.13 (d, *J* = 7.0 Hz, 2H), 8.07 – 7.99 (m, 2H), 7.83 (t, *J* = 7.8 Hz, 1H), 7.72 (dd, *J* = 7.9, 0.9 Hz, 1H), 7.67 (dd, *J* = 7.8, 0.9 Hz, 1H), 7.65 – 7.60 (m, 2H), 7.51 (t, *J* = 7.3 Hz, 2H), 7.44 (t, *J* = 7.2 Hz, 1H) ppm.

^{13}C{^{1}H} NMR (101 MHz, $CDCl_3$): δ = 157.1, 155.8, 139.2, 138.3, 138.0, 132.0, 129.4, 128.9, 128.8, 127.2, 123.7, 119.3, 118.7 ppm.

HRMS-ESI(+) (m/z) calcd. for $C_{17}H_{13}BrN$ $[M+H]^+$: 310.0226; found: 310.0226.

The spectral data were in accordance with those reported in literature.[50]

Synthesis of **10c**:

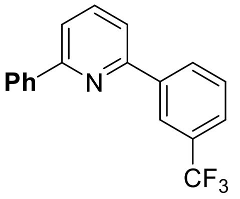

Prepared according to general procedure E from 2-phenyl-5-(3-(trifluoromethyl)phenyl)-1*H*-pyrrole **9c** (115 mg, 0.8 mmol, 2.0 equiv.). The NMR yield of the crude reaction mixture was 45%, referenced against CH_2Br_2 as an internal standard. Purified by column chromatography using hexane/EtOAc (100/0→95/5 (v/v)) as the eluent. Colorless solid (50.2 mg, 168 µmol, 42%).

^{1}**H NMR** (400 MHz, $CDCl_3$): δ = 8.41 (s, 1H), 8.34 (d, *J* = 7.8 Hz, 1H), 8.19 – 8.11 (m, 2H), 7.87 (t, *J* = 7.8 Hz, 1H), 7.74 (ddd, *J* = 10.7, 7.8, 0.9 Hz, 2H), 7.69 (d, *J* = 7.6 Hz, 1H), 7.62 (t, *J* = 7.8 Hz, 1H), 7.53 (t, *J* = 7.3 Hz, 2H), 7.46 (t, *J* = 7.3 Hz, 1H) ppm.

^{19}**F NMR** (282 MHz, $CDCl_3$): δ = −62.6 ppm.

^{13}**C{^{1}H} NMR** (101 MHz, $CDCl_3$): δ = 157.3, 155.4, 140.3, 139.2, 137.9, 131.3 (q, *J* = 32.3 Hz), 130.4, 129.4, 129.3, 128.9, 127.2, 125.7 (q, *J* = 3.6 Hz), 124.0 (q, *J* = 3.9 Hz), 124.4 (q, *J* = 272.6 Hz), 119.5, 118.9 ppm.

HRMS-ESI(+) (m/z) calcd. for $C_{18}H_{13}F_3N$ $[M+H]^+$: 300.0995; found: 300.0994.

The spectral data were in accordance with those reported in literature.[51]

Synthesis of **10d**:

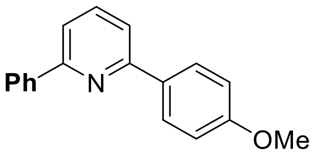

Prepared according to general procedure E from 2-(4-methoxyphenyl)-5-phenyl-1*H*-pyrrole **9d** (200 mg, 0.8 mmol, 2.0 equiv.). The NMR yield of the crude reaction mixture was 35%, referenced against CH_2Br_2 as an internal standard. Purified by column chromatography using hexane/EtOAc (100/0→95/5 (v/v)) as the eluent. Colorless solid (32.8 mg, 126 µmol, 31%).

^{1}**H NMR** (400 MHz, $CDCl_3$): δ = 8.20 – 8.06 (m, 4H), 7.79 (t, *J* = 7.8 Hz, 1H), 7.70 – 7.59 (m, 2H), 7.50 (t, *J* = 7.3 Hz, 2H), 7.43 (t, *J* = 7.3 Hz, 1H), 7.09 – 6.97 (m, 2H), 3.88 (s, 3H) ppm.

^{13}**C{^{1}H} NMR** (101 MHz, $CDCl_3$): δ = 160.7, 156.8, 156.6, 139.6, 137.6, 132.2, 129.1, 128.8, 128.4, 127.2, 118.2, 118.1, 114.2, 55.5 ppm.

HRMS-ESI(+) (m/z) calcd. for $C_{18}H_{16}NO$ $[M+H]^+$: 262.1226; found: 262.1226.

The spectral data were in accordance with those reported in literature.[50]

Synthesis of **10e**:

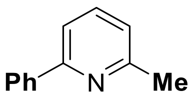

Prepared according to general procedure E from 2-methyl-5-phenyl-1*H*-pyrrole **9e** (126 mg, 0.8 mmol, 2.0 equiv.). The NMR yield of the crude reaction mixture was 37%, referenced

against CH_2Br_2 as an internal standard. Purified by column chromatography using hexane/EtOAc (100/0→95/5 (v/v)) as the eluent. Colorless oil (22.4 mg, 132 µmol, 33%).

^{1}H NMR (400 MHz, $CDCl_3$): δ = 8.02 – 7.94 (m, 2H), 7.63 (t, *J* = 7.7 Hz, 1H), 7.52 (d, *J* = 7.9 Hz, 1H), 7.47 (t, *J* = 7.3 Hz, 2H), 7.40 (t, *J* = 7.3 Hz, 1H), 7.10 (d, *J* = 7.6 Hz, 1H), 2.63 (s, 3H) ppm.

^{13}C{^{1}H} NMR (101 MHz, $CDCl_3$): δ = 158.5, 157.1, 139.9, 137.0, 128.8, 128.8, 127.1, 121.7, 117.8, 24.9 ppm.

HRMS-ESI(+) (m/z) calcd. for $C_{12}H_{12}N$ $[M+H]^+$: 170.0964; found: 170.0963.

The spectral data were in accordance with those reported in literature.[52]

Synthesis of **10f**:

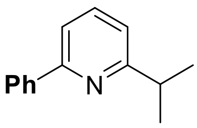

Prepared according to general procedure E from 2-isopropyl-5-phenyl-1*H*-pyrrole **9f** (148 mg, 0.8 mmol, 2.0 equiv.). The NMR yield of the crude reaction mixture was 52%, referenced against CH_2Br_2 as an internal standard. Purified by column chromatography using hexane/EtOAc (100/0→95/5 (v/v)) as the eluent. Colorless oil (35.7 mg, 181 µmol, 45%).

^{1}H NMR (400 MHz, $CDCl_3$): δ = 8.05 (dd, *J* = 8.4, 1.4 Hz, 2H), 7.67 (t, *J* = 7.7 Hz, 1H), 7.54 (dd, *J* = 7.8, 0.9 Hz, 1H), 7.47 (t, *J* = 7.3 Hz, 2H), 7.40 (t, *J* = 7.3 Hz, 1H), 7.12 (dd, *J* = 7.7, 0.9 Hz, 1H), 3.15 (hept, *J* = 6.9 Hz, 1H), 1.38 (s, 3H), 1.37 (s, 3H) ppm.

^{13}C{^{1}H} NMR (101 MHz, $CDCl_3$): δ = 167.3, 156.5, 140.0, 137.1, 128.8, 128.8, 127.1, 119.0, 117.8, 36.6, 22.8 ppm.

HRMS-ESI(+) (m/z) calcd. for $C_{14}H_{16}N$ $[M+H]^+$: 198.1277; found: 198.1276.

The spectral data were in accordance with those reported in literature.[53]

Synthesis of **10g**:

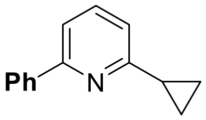

Prepared according to general procedure E from 2-cyclopropyl-5-phenyl-1*H*-pyrrole **9g** (147 mg, 0.8 mmol, 2.0 equiv.). The NMR yield of the crude reaction mixture was 28%,

referenced against CH_2Br_2 as an internal standard. Purified by column chromatography using hexane/EtOAc (100/0→95/5 (v/v)) as the eluent. Colorless oil (12.7 mg, 65.0 μmol, 16%).

^{1}H NMR (400 MHz, $CDCl_3$): δ = 8.04 – 7.97 (m, 2H), 7.59 (t, *J* = 7.7 Hz, 1H), 7.49 (dd, *J* = 7.8, 0.9 Hz, 1H), 7.45 (t, *J* = 7.3 Hz, 2H), 7.38 (t, *J* = 7.3 Hz, 1H), 7.07 (dd, *J* = 7.6, 1.0 Hz, 1H), 2.16 – 2.04 (m, 1H), 1.19 – 1.07 (m, 2H), 1.06 – 0.94 (m, 2H) ppm.

^{13}C{^{1}H} NMR (101 MHz, $CDCl_3$): δ = 162.6, 156.4, 136.7, 128.9, 128.7, 127.0, 119.8, 117.1, 29.9, 17.3, 10.1 ppm.

HRMS-ESI(+) (m/z) calcd. for $C_{14}H_{14}N$ $[M+H]^+$: 196.1121; found: 196.1120.

The spectral data were in accordance with those reported in literature.[54]

Synthesis of **10h**:

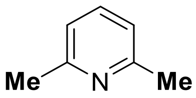

Prepared according to general procedure E from 2,5-dimethyl-1*H*-pyrrole **9h** (76.1 mg, 0.8 mmol, 2.0 equiv.). The NMR yield of the crude reaction mixture was 30%, referenced against CH_2Br_2 as an internal standard. The crude reaction mixture was concentrated under reduced pressure and Et_2O was added to the residue. The solution was extracted with 1 M HCl (10 mL). The organic and aqueous phases were separated, and the organic phase was washed with demineralized water (3 × 10 mL). The combined aqueous phases were washed with Et_2O (3 × 10 mL). The combined aqueous phases were neutralized using sat. $NaHCO_3$, and subsequently washed with Et_2O (3 × 10 mL). The organic phases were dried over $MgSO_4$ and the solvent was removed under reduced pressure. The desired pyridine is volatile.

^{1}H NMR (400 MHz, $CDCl_3$): δ = 7.45 (t, *J* = 7.6 Hz, 1H), 6.95 (dt, *J* = 7.6, 0.6 Hz, 2H), 2.52 (s, 6H) ppm.

^{13}C{^{1}H} NMR (101 MHz, $CDCl_3$): δ = 157.8, 136.7, 120.3, 24.6 ppm.

HRMS-ESI(+) (m/z) calcd. for $C_7H_{10}N$ $[M+H]^+$: 108.0808; found: 108.0808.

The spectral data were in accordance with those reported in literature.[55]

Synthesis of **10i**:

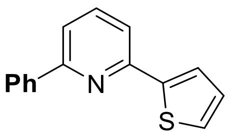

Prepared according to general procedure E from 2-phenyl-5-(thiophen-2-yl)-1*H*-pyrrole **9i** (180 mg, 0.8 mmol, 2.0 equiv.). The NMR yield of the crude reaction mixture was 26%, referenced against CH_2Br_2 as an internal standard. Purified by column chromatography using hexane/EtOAc (100/0→95/5 (v/v)) as the eluent. Colorless solid (18.9 mg, 79.6 µmol, 20%).

^{1}H NMR (400 MHz, $CDCl_3$): δ = 8.18 – 8.09 (m, 2H), 7.75 (t, *J* = 7.8 Hz, 1H), 7.66 (dd, *J* = 3.7, 1.1 Hz, 1H), 7.60 (ddd, *J* = 10.4, 7.9, 0.9 Hz, 2H), 7.50 (t, *J* = 7.3 Hz, 2H), 7.47 – 7.39 (m, 2H), 7.13 (dd, *J* = 5.0, 3.7 Hz, 1H) ppm.

^{13}C{^{1}H} NMR (101 MHz, $CDCl_3$): δ = 156.8, 152.4, 145.6, 139.0, 137.6, 129.3, 128.8, 128.1, 127.8, 127.1, 124.7, 118.4, 117.1 ppm.

HRMS-ESI(+) (m/z) calcd. for $C_{15}H_{12}NS$ $[M+H]^+$: 238.0685; found: 238.0684.

The spectral data were in accordance with those reported in literature.[56]

Synthesis of **10j**:

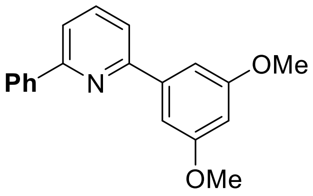

Prepared according to general procedure E from 2-(3,5-dimethoxyphenyl)-5-phenyl-1*H*-pyrrole **9j** (223 mg, 0.8 mmol, 2.0 equiv.). The NMR yield of the crude reaction mixture was 30%, referenced against CH_2Br_2 as an internal standard. Purified by column chromatography using hexane/EtOAc (100/0→95/5 (v/v)) as the eluent. Colorless solid (24.6 mg, 84.4 µmol, 21%).

^{1}H NMR (400 MHz, $CDCl_3$): δ = 8.19 – 8.11 (m, 2H), 7.81 (t, *J* = 7.8 Hz, 1H), 7.68 (dd, *J* = 16.9, 7.3 Hz, 2H), 7.50 (t, *J* = 6.9 Hz, 2H), 7.44 (t, *J* = 7.2 Hz, 1H), 7.33 (d, *J* = 2.3 Hz, 2H), 6.56 (t, *J* = 2.3 Hz, 1H), 3.90 (s, 6H) ppm.

^{13}C{^{1}H} NMR (101 MHz, $CDCl_3$): δ = 161.2, 156.8, 156.6, 141.8, 139.5, 137.6, 129.1, 128.8, 127.1, 119.1, 119.0, 105.4, 101.2, 55.6 ppm.

IR (ATR, neat): $\tilde{\nu}$ = 3075, 2960, 2919, 2835, 2359, 2340, 1587, 1566, 1452, 1426, 1385, 1352, 1310, 1284, 1201, 1153, 1099, 1062, 1038, 987, 927, 863, 838, 810, 761, 749, 738, 690, 668, 658, 625, 542, 480, 437, 421 cm^{-1}.

HRMS-ESI(+) (m/z) calcd. for $C_{19}H_{18}NO_2$ $[M+H]^+$: 292.1332; found: 292.1329.

Melting point: 70 °C.

Synthesis of **10k**:

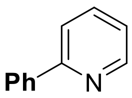

Prepared according to general procedure E from 2-phenyl-1*H*-pyrrole **9k** (115 mg, 0.8 mmol, 2.0 equiv.). The NMR yield of the crude reaction mixture was 12%, referenced against CH_2Br_2 as an internal standard. Purified by column chromatography using hexane/EtOAc (100/0→95/5 (v/v)) as the eluent. Colorless oil (5.60 mg, 36.1 µmol, 9%).

^{1}H NMR (400 MHz, $CDCl_3$): δ = 8.74 – 8.67 (m, 1H), 8.04 – 7.96 (m, 2H), 7.75 (dd, *J* = 6.1, 1.5 Hz, 2H), 7.48 (t, *J* = 7.3 Hz, 2H), 7.42 (t, *J* = 7.3 Hz, 1H), 7.23 (ddd, *J* = 6.8, 4.8, 2.3 Hz, 1H) ppm.

^{13}C{^{1}H} NMR (101 MHz, $CDCl_3$): δ = 157.6, 149.8, 139.5, 136.9, 129.1, 128.9, 127.1, 122.2, 120.7 ppm.

HRMS-ESI(+) (m/z) calcd. for $C_{11}H_{10}N$ $[M+H]^+$: 156.0808; found: 156.0807.

The spectral data were in accordance with those reported in literature.[57]

Side reactions for the formation of 1,1-di(pyrrol-1-yl)methane products **11**:

Synthesis of **11a**:

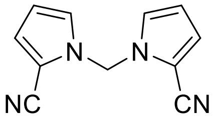

Prepared according to general procedure E from methyl 1*H*-pyrrole-2-carbonitrile **9l** (73.7 mg, 0.8 mmol, 2.0 equiv.). The NMR yield of the crude reaction mixture was 80%, referenced against CH_2Br_2 as an internal standard. Purified by column chromatography using hexane/EtOAc (100/0→95/5 (v/v)) as the eluent. Colorless solid (30.1 mg, 289 µmol, 72%).

^{1}H NMR (400 MHz, $CDCl_3$): δ = 7.27 (dd, *J* = 2.8, 1.7 Hz, 2H), 6.87 (dd, *J* = 4.0, 1.5 Hz, 2H), 6.27 (dd, *J* = 4.0, 2.8 Hz, 2H), 6.13 (s, 2H) ppm.

^{13}C{^{1}H} NMR (101 MHz, $CDCl_3$): δ = 127.5, 122.2, 113.5, 111.5, 103.6, 58.4 ppm.

IR (ATR, neat): $\tilde{\nu}$ = 3127, 2217, 1563, 1531, 1478, 1451, 1408, 1369, 1308, 1280, 1239, 1217, 1074, 1030, 985, 882, 830, 731, 634, 608, 449, 430, 413 cm^{-1}.

HRMS-ESI(+) (m/z) calcd. for $C_{11}H_8N_4Na$ $[M+Na]^+$: 219.0641; found: 219.0640.

Melting point: 188 °C.

Synthesis of **11b**:

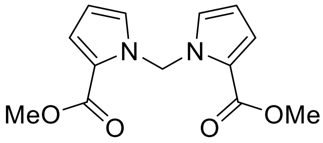

Prepared according to general procedure E from methyl 1*H*-pyrrole-2-carboxylate **9m** (100 mg, 0.8 mmol, 2.0 equiv.). The NMR yield of the crude reaction mixture was 85%, referenced against CH_2Br_2 as an internal standard. Purified by column chromatography using hexane/EtOAc (100/0→95/5 (v/v)) as the eluent. Colorless solid (34.5 mg, 252 µmol, 63%).

^{1}H NMR (400 MHz, $CDCl_3$): δ = 7.30 (dd, *J* = 2.8, 1.8 Hz, 2H), 6.97 (dd, *J* = 3.9, 1.8 Hz, 2H), 6.94 (s, 2H), 6.12 (dd, *J* = 3.9, 2.7 Hz, 2H), 3.85 (s, 6H) ppm.

^{13}C{^{1}H} NMR (101 MHz, $CDCl_3$): δ = 162.1, 129.4, 121.4, 119.5, 109.4, 56.8, 51.5 ppm.

IR (ATR, neat): $\tilde{\nu}$ = 3123, 2955, 2921, 2849, 1695, 1535, 1474, 1439, 1414, 1377, 1310, 1234, 1190, 1119, 1074, 1034, 980, 950, 881, 809, 759, 734, 716, 607, 506, 422, 405 cm^{-1}.

HRMS-ESI(+) (m/z) calcd. for $C_{13}H_{14}N_2O_4$ $[M+Na]^+$: 285.0846; found: 285.0845.

Melting point: 133 °C.

Synthesis of **11c**:

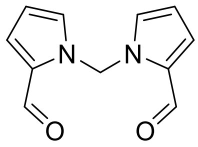

Prepared according to general procedure E from 1*H*-pyrrole-2-carbaldehyde **9n** (76.1 mg, 0.8 mmol, 2.0 equiv.). The NMR yield of the crude reaction mixture was 57%, referenced against CH_2Br_2 as an internal standard. Purified by column chromatography using hexane/EtOAc (100/0→95/5 (v/v)) as the eluent. Colorless solid (20.1 mg, 188 µmol, 47%).

^{1}H NMR (400 MHz, $CDCl_3$): δ = 9.57 (d, *J* = 1.1 Hz, 2H), 7.57 (ddd, *J* = 2.8, 1.7, 1.1 Hz, 2H), 6.96 (dd, *J* = 4.0, 1.7 Hz, 2H), 6.90 (s, 2H), 6.23 (dd, *J* = 4.0, 2.7 Hz, 2H) ppm.

^{13}C{^{1}H} NMR (101 MHz, $CDCl_3$): δ = 180.1, 132.8, 131.0, 126.8, 111.1, 56.6 ppm.

HRMS-ESI(+) (m/z) calcd. for $C_{11}H_{10}N_2O_2$ $[M+Na]^+$: 225.0634; found: 225.0634.

The spectral data were in accordance with those reported in literature.[58]

SYNTHESIS OF ^{13}C LABELLED PYRIDINES

Synthesis of **10a***:

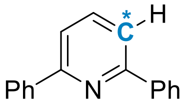

Prepared as **10a** according to general procedure E from 2,5-diphenyl-1*H*-pyrrole **9a** (175 mg, 0.8 mmol, 2.0 equiv.) but using **1*** instead of **1**. The NMR yield of the crude reaction mixture was 58%, referenced against CH_2Br_2 as an internal standard. Purified by column chromatography using hexane/EtOAc (100/0→95/5 (v/v)) as the eluent. White solid (45.7 mg, 197 μmol, 49%).

^{1}H NMR (500 MHz, $CDCl_3$): δ = 8.22 – 8.15 (m, 4H), 7.87 (dd, *J* = 7.8, 1.0 Hz, 0.5H), 7.82 (t, *J* = 7.8 Hz, 1H), 7.71 (ddd, *J* = 7.7, 6.5, 1.0 Hz, 1H), 7.57 – 7.49 (m, 4.5H), 7.46 (t, *J* = 7.3 Hz, 2H) ppm.

^{13}C{^{1}H} NMR (126 MHz, $CDCl_3$): δ = 156.9 (dd, *J* = 32.6, 21.2 Hz), 139.6 (t, *J* = 3.0 Hz), 137.6 (d, *J* = 55.5 Hz), 129.1, 128.8, 127.1, **118.8** (labelled carbon atom) ppm.

^{13}C NMR (126 MHz, $CDCl_3$): δ = **118.75** (dd, *J* = 162.6, 6.5 Hz) ppm.

HRMS-ESI(+) (m/z) calcd. for $C_{16}{}^{13}CH_{14}N$ $[M+H]^+$: 233.1154; found: 233.1154.

Synthesis of **10f***:

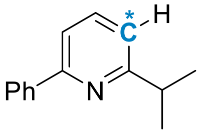

10f*^{I}

and

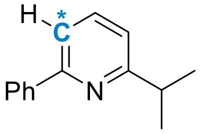

10f*^{II}

10f*^{I}:10f*^{II} = 1.1:1

Prepared as **10f** according to general procedure E from 2-isopropyl-5-phenyl-1*H*-pyrrole **9f** (148 mg, 0.8 mmol, 2.0 equiv.) but using **1*** instead of **1**. The NMR yield of the crude reaction mixture was 48%, referenced against CH_2Br_2 as an internal standard. Purified by column chromatography using hexane/EtOAc (100/0→95/5 (v/v)) as the eluent. Colorless oil (33.3 mg, 169 μmol, 42%).

^{1}H NMR (500 MHz, $CDCl_3$): δ = 8.09 – 8.02 (m, 2H), 7.71 (dd, *J* = 7.8, 1.2 Hz, 0.25H), 7.67 (t, *J* = 7.8 Hz, 1H), 7.55 (ddd, *J* = 7.7, 6.4, 1.0 Hz, 0.55H), 7.47 (t, *J* = 7.4 Hz, 2H), 7.43 – 7.36

(m, 1.26H), 7.28 (d, J = 7.6 Hz, 0.26H), 7.12 (ddd, J = 7.8, 6.6, 1.1 Hz, 0.48H), 6.96 (d, J = 7.7 Hz, 0.26H), 3.16 (sept, J = 6.2 Hz, 1H), 1.38 (s, 3H), 1.37 (s, 3H) ppm.

$^{13}C\{^1H\}$ NMR (126 MHz, $CDCl_3$): δ = 167.3 (dd, J = 36.7, 20.3 Hz), 156.6 (d, J = 21.2 Hz), 156.3 (d, J = 26.2 Hz), 140.0, 137.1 (d, J = 55.3 Hz), 128.8, 128.8, 127.1, **119.0** (labelled carbon atom), **117.8** (labelled carbon atom), 36.6 – 36.5 (m), 22.8 ppm.

^{13}C NMR (126 MHz, $CDCl_3$): δ = **119.7** (dd, J = 6.6, 3.4 Hz), **118.4 – 118.1** (m), **117.1** (d, J = 6.3 Hz) ppm.

HRMS-ESI(+) (m/z) calcd. for $C_{13}{}^{13}CH_{16}N$ $[M+H]^+$: 199.1311; found: 199.1310.

X-RAY CRYSTALLOGRAPHIC ANALYSIS

Compound **1**◦MeCN

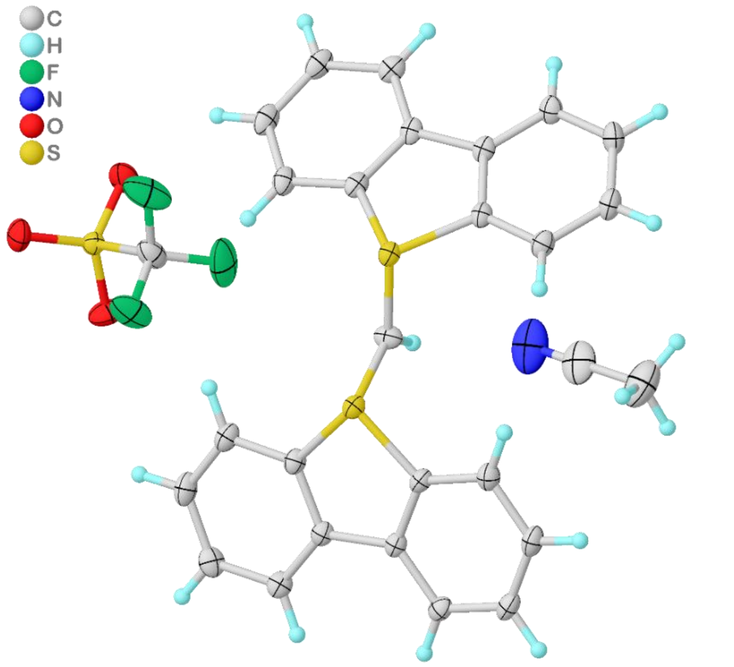

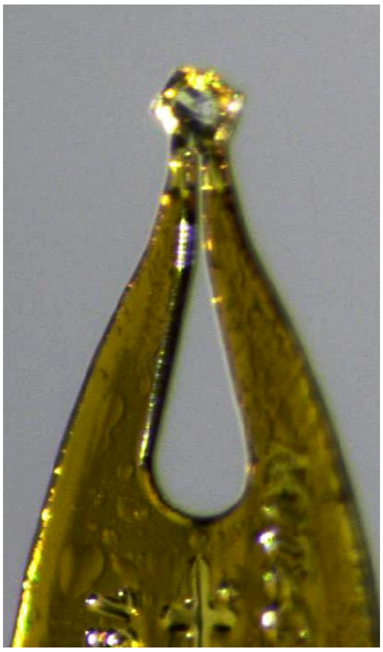

Figure S1: X-Ray solid-state structure of **1**◦MeCN. Ellipsoids are shown at the 50% probability level. Single crystals were obtained from a mixture of acetonitrile and diethyl ether by solvent vapor diffusion method.

CCDC number	2477303
Empirical formula	$C_{28}H_{20}F_3NO_3S_3$
Formula weight	571.63
Temperature [K]	100.00
Crystal system	Orthorhombic
Space group (number)	$P2_12_12_1$ (19)
a [Å]	11.5088(18)
b [Å]	13.078(3)
c [Å]	17.125(4)
α [°]	90
β [°]	90
γ [°]	90
Volume [Å^3]	2577.6(8)
Z	4
ρ_{calc} [gcm^{-3}]	1.473
μ [mm^{-1}]	0.342
F(000)	1176
Crystal size [mm^3]	0.136×0.095×0.062
Crystal colour	Colourless
Crystal shape	Block
Radiation	MoK_α (λ=0.71073 Å)

2θ range [°]	3.92 to 59.27 (0.72 Å)
Index ranges	−15 ≤ h ≤ 15<br>−18 ≤ k ≤ 18<br>−23 ≤ l ≤ 23
Reflections collected	78013
Independent reflections	7238<br>R_{int} = 0.0624<br>R_{sigma} = 0.0292
Completeness to θ = 25.242°	100.0 %
Data / Restraints / Parameters	7238/0/349
Absorption correction T_{min}/T_{max} (method)	0.7763/1.0000 (numerical)
Goodness-of-fit on F^2	1.069
Final *R* indexes [I≥2σ(I)]	R_1 = 0.0339<br>wR_2 = 0.0809
Final *R* indexes [all data]	R_1 = 0.0409<br>wR_2 = 0.0853
Largest peak/hole [eÅ^{-3}]	0.34/−0.32
Flack X parameter	0.51(7)

Compound **8a(exo)**

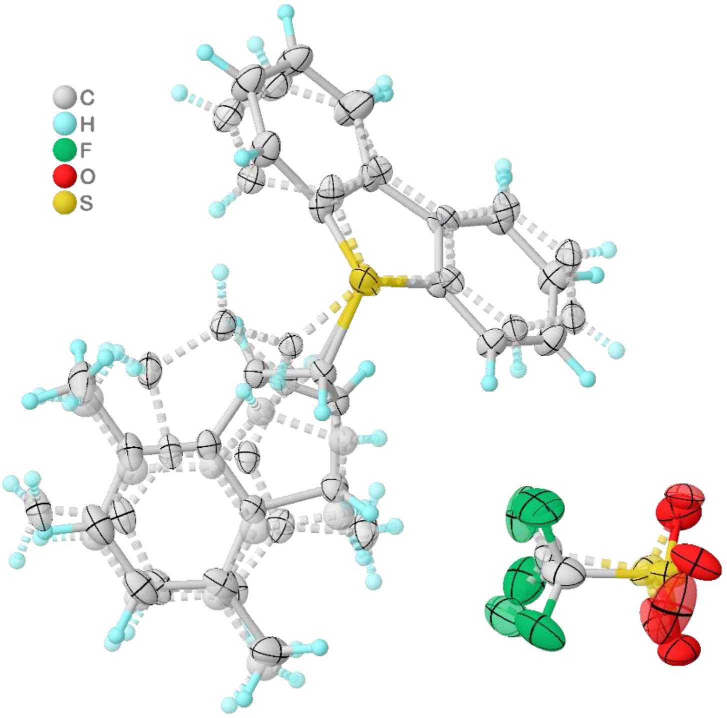

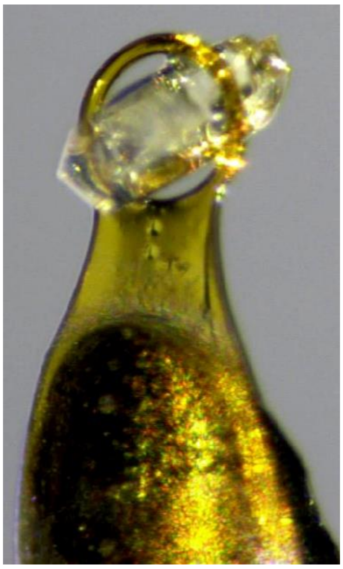

Figure S2: X-Ray solid-state structure of **8a(exo).** Ellipsoids are shown at the 50% probability level. The structure exhibits extensive disorder, which is modelled for the tricycle with three parts of differing conformations. Minor disorder parts are depicted as translucent ellipsoids with stippled bonds; the least occupied conformational disorder position for the tricycle is refined only isotropically due to the low refined occupancy of 0.12312. Single crystals were obtained from a mixture of acetonitrile and diethyl ether by solvent vapor diffusion method.

CCDC number	2477304
Empirical formula	$C_{25}H_{21}F_3O_3S_2$
Formula weight	490.54
Temperature [K]	100.00
Crystal system	Monoclinic
Space group (number)	$P2_1/n$ (14)
a [Å]	10.0623(6)
b [Å]	17.0384(9)
c [Å]	13.7669(9)
α [°]	90
β [°]	110.880(2)
γ [°]	90
Volume [Å^3]	2205.3(2)
Z	4
ρ_{calc} [gcm^{-3}]	1.477
μ [mm^{-1}]	0.293
F(000)	1016
Crystal size [mm^3]	0.192×0.325×0.544
Crystal colour	Colourless
Crystal shape	Block
Radiation	MoK_α (λ=0.71073 Å)

2θ range [°]	3.97 to 63.09 (0.68 Å)
Index ranges	−14 ≤ h ≤ 14<br>−25 ≤ k ≤ 25<br>−20 ≤ l ≤ 20
Reflections collected	61873
Independent reflections	7357<br>R_{int} = 0.0409<br>R_{sigma} = 0.0202
Completeness to θ = 25.242°	100.0 %
Data / Restraints / Parameters	7357 / 642 / 641
Absorption correction T_{min}/T_{max} (method)	0.7644 / 1.0000 (numerical)
Goodness-of-fit on F^2	1.166
Final *R* indexes [I≥2σ(I)]	R_1 = 0.0496<br>wR_2 = 0.1201
Final *R* indexes [all data]	R_1 = 0.0551<br>wR_2 = 0.1232
Largest peak/hole [eÅ^{-3}]	0.36/−0.62

SPECTROSCOPIC DATA

Compound **1**

^{1}H NMR (400 MHz, CD_3CN, 70 °C)

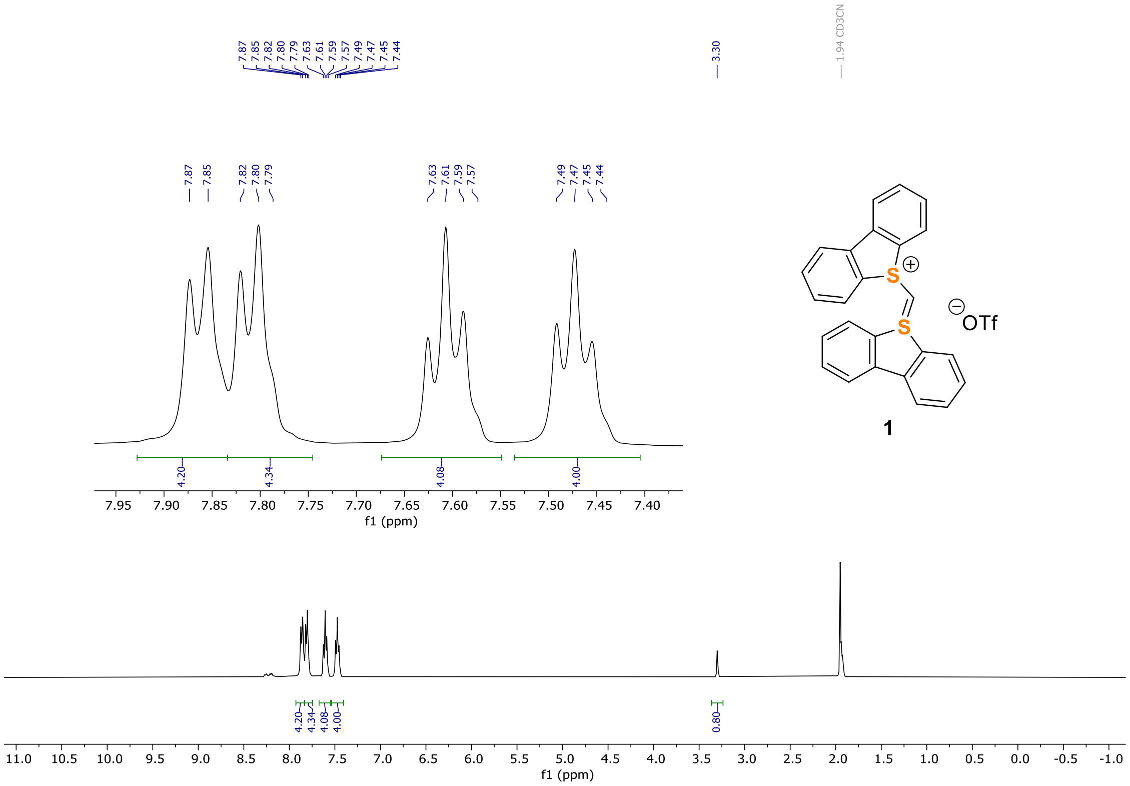

$^{13}C\{^{1}H\}$ NMR (101 MHz, CD_3CN, 70 °C)

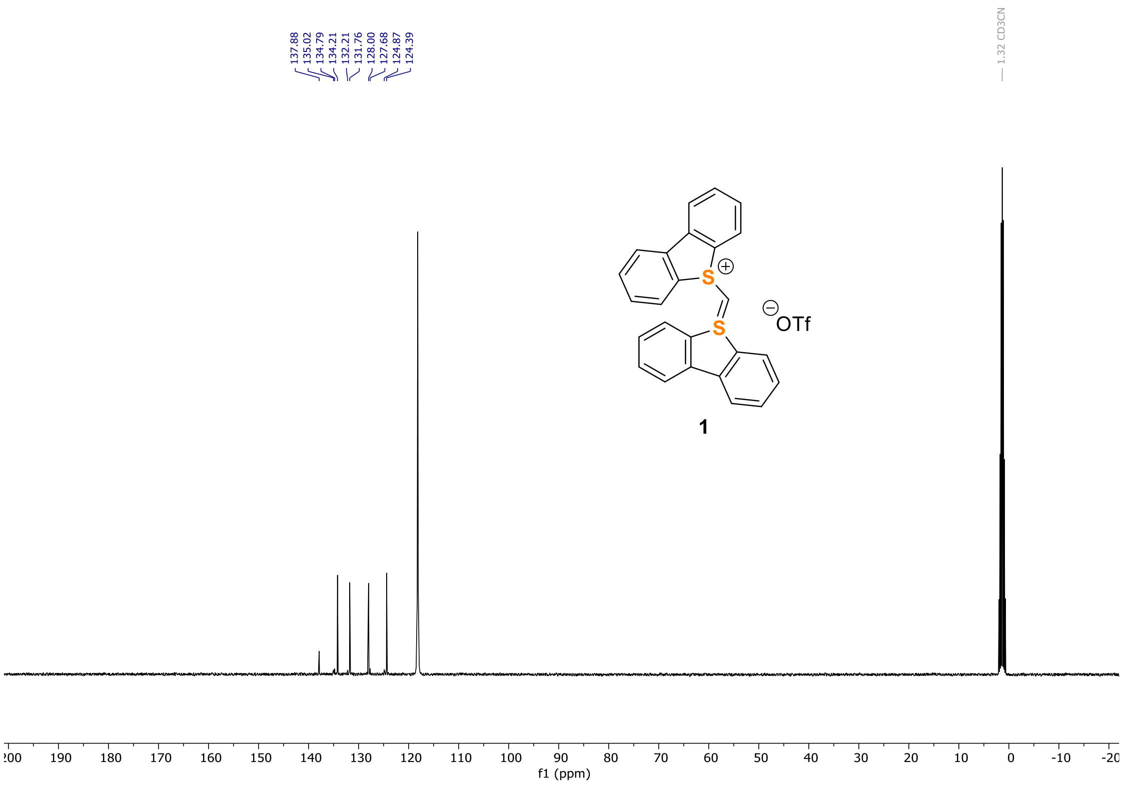

^{19}F NMR (377 MHz, CD_3CN)

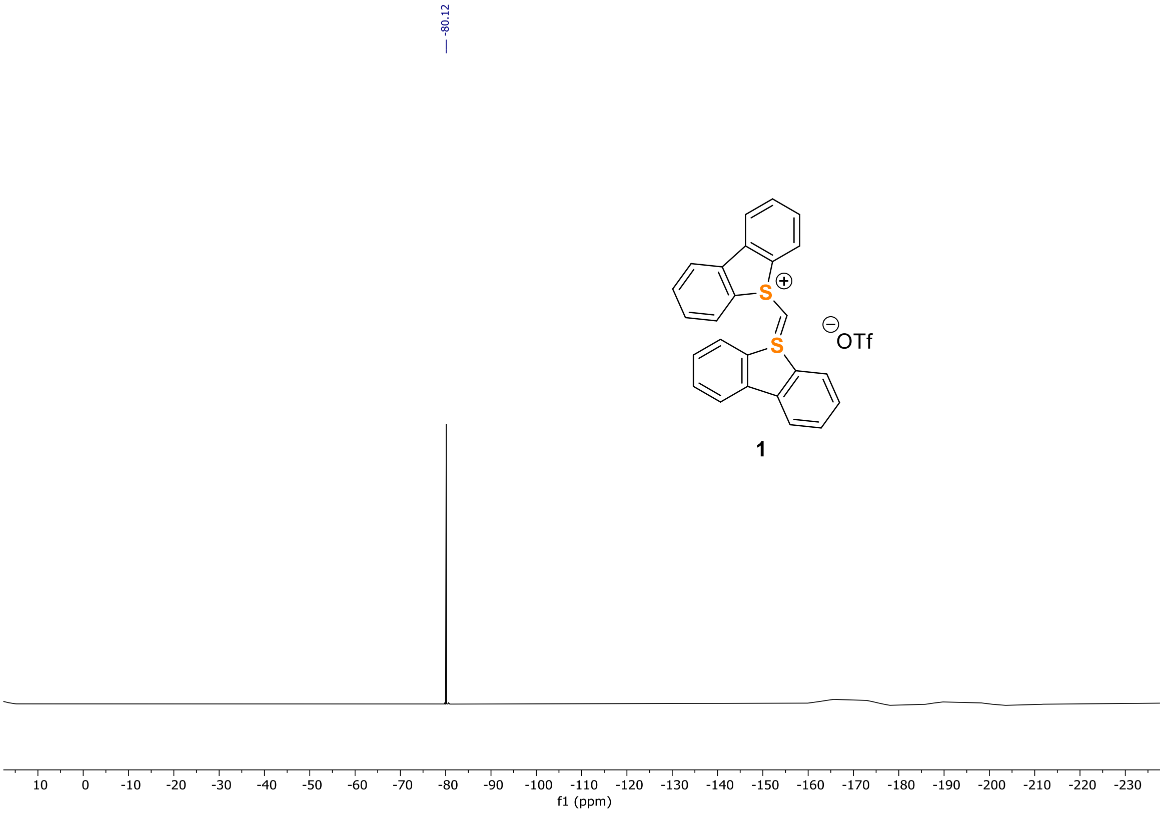

1

Compound **1***

^{1}H NMR (500 MHz, CD_3CN, 70 °C)

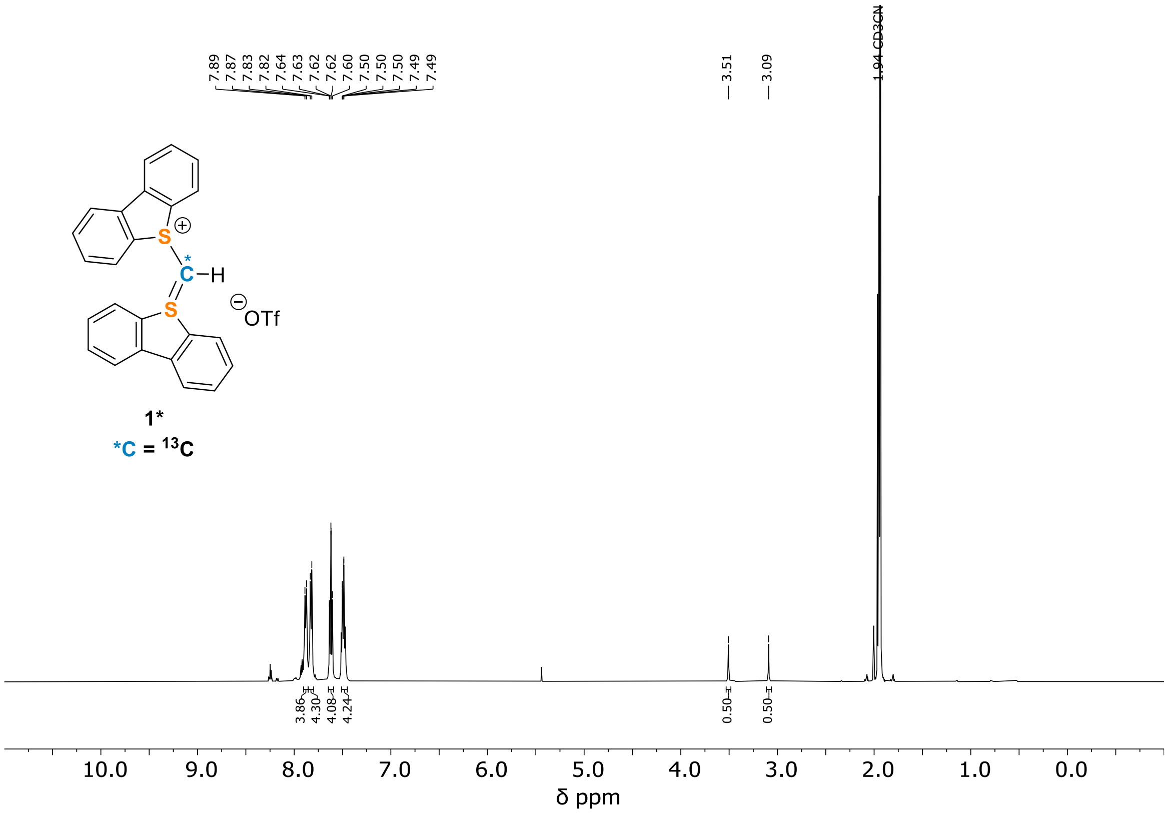

^{13}C{^{1}H} NMR (126 MHz, CD_3CN, 70 °C)

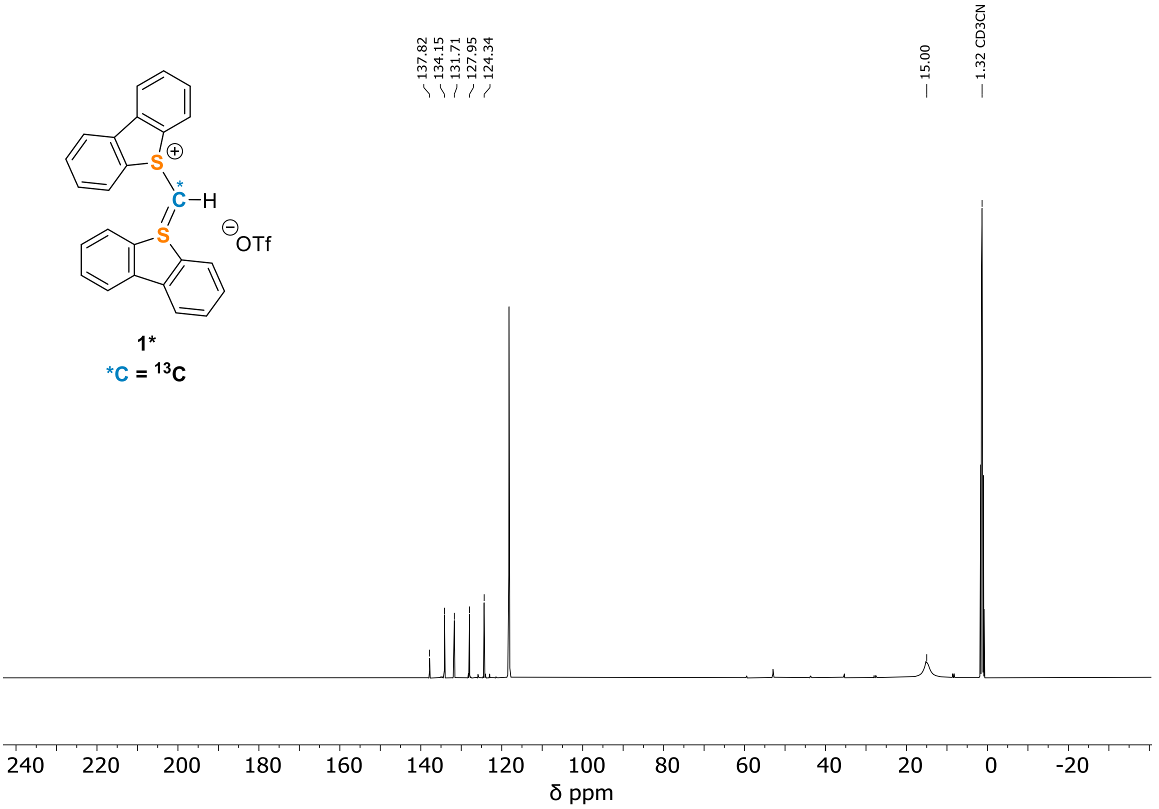

Compound **3**

^{1}H NMR (300 MHz, $CDCl_3$)

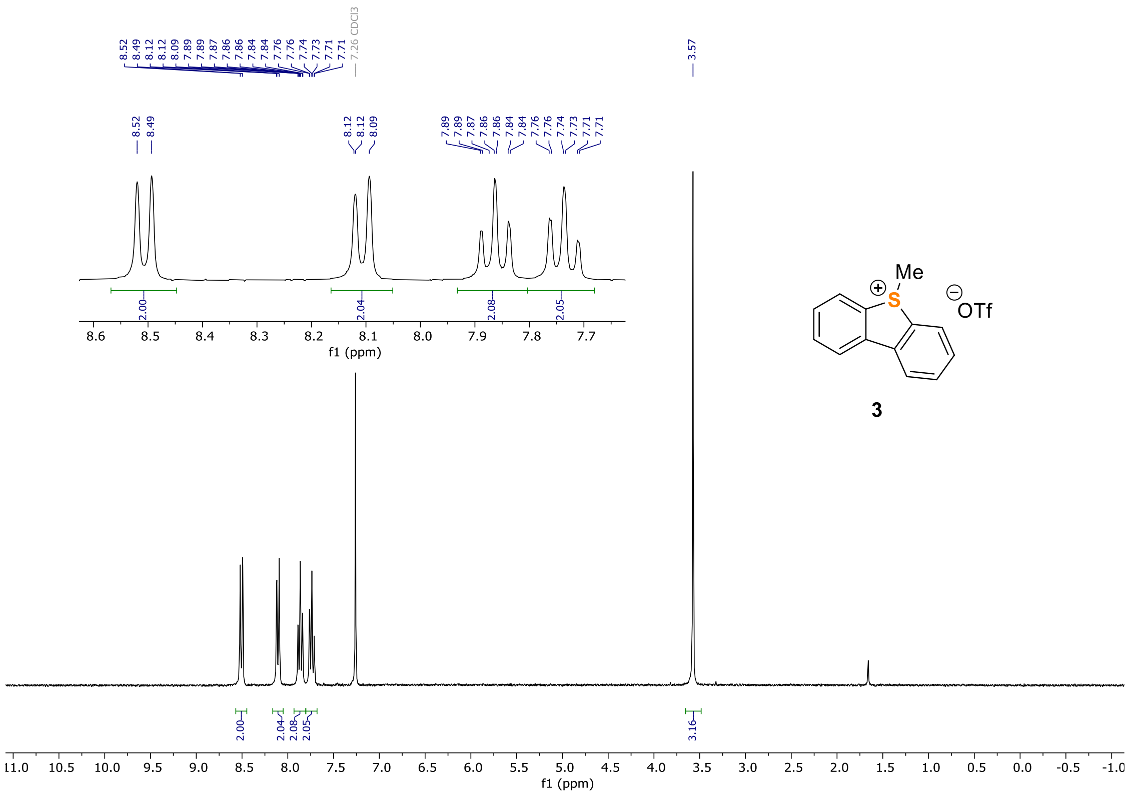

^{13}C{^{1}H} NMR (101 MHz, CD_3CN)

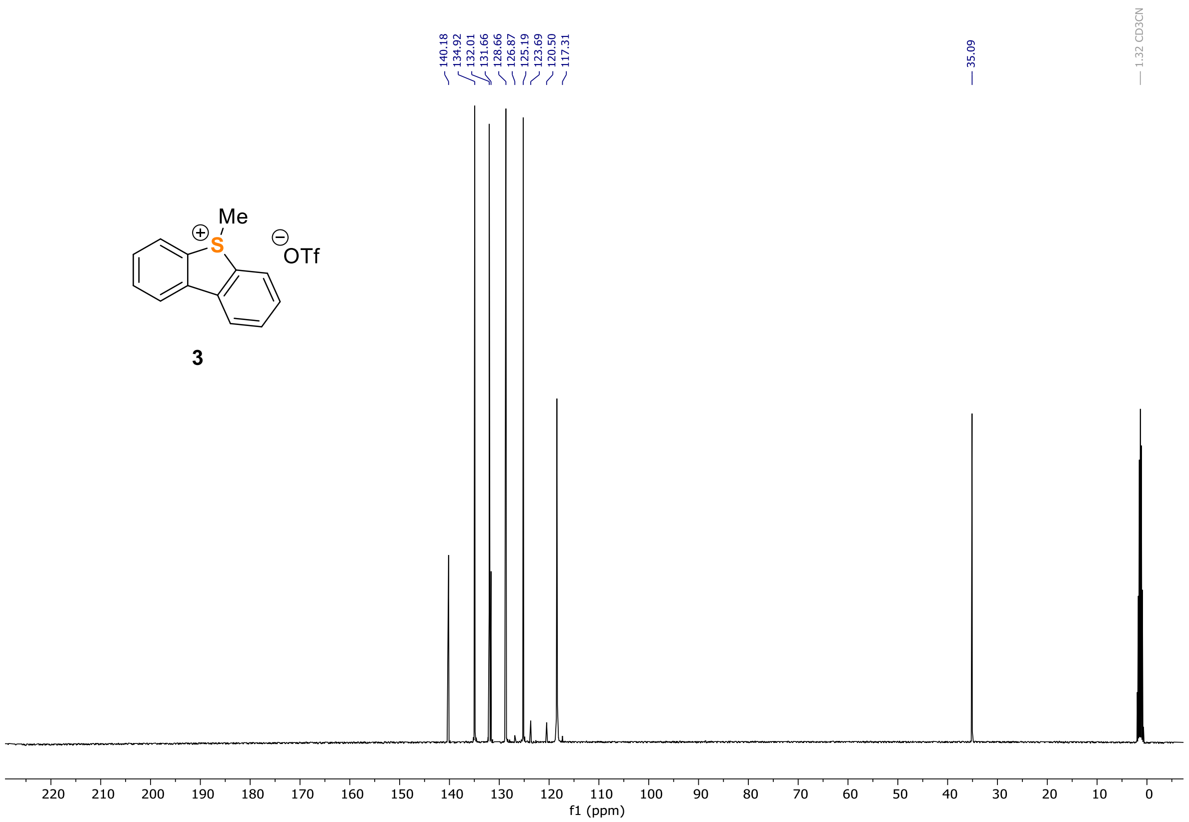

Compound **3***

^{1}H NMR (300 MHz, CD_3CN)

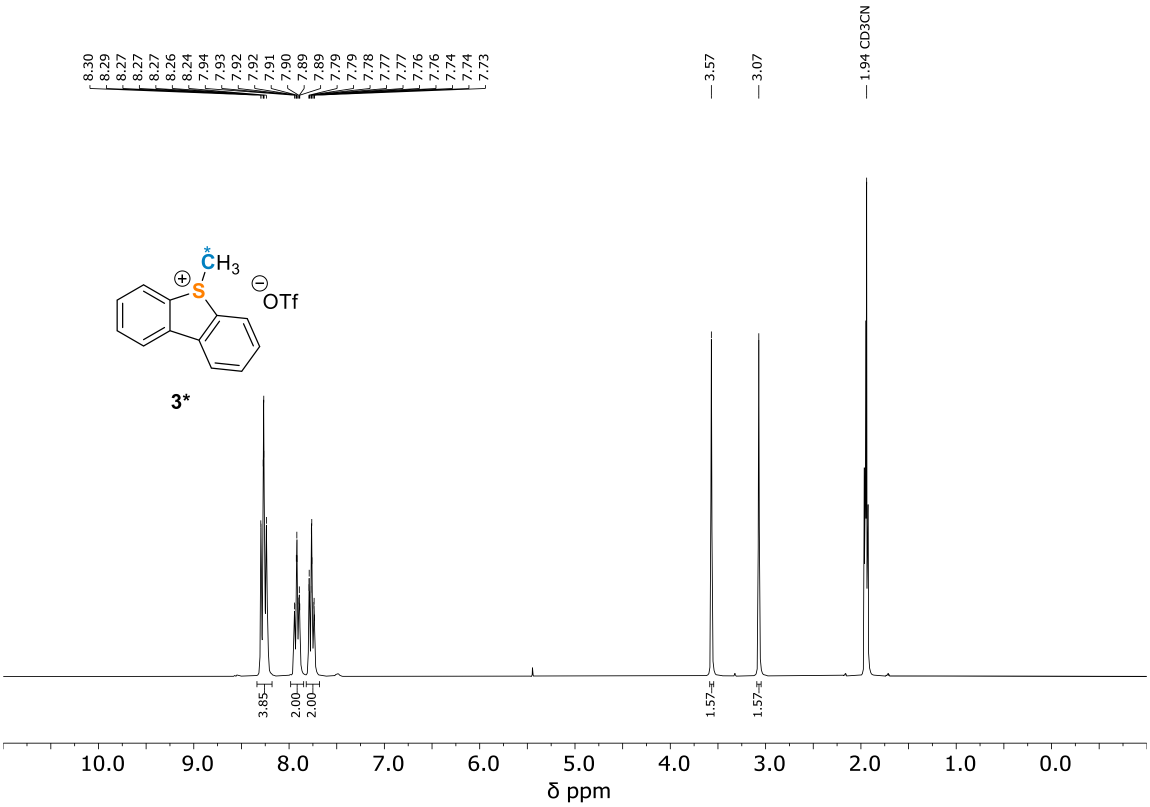

$^{13}C\{^{1}H\}$ NMR (101 MHz, CD_3CN)

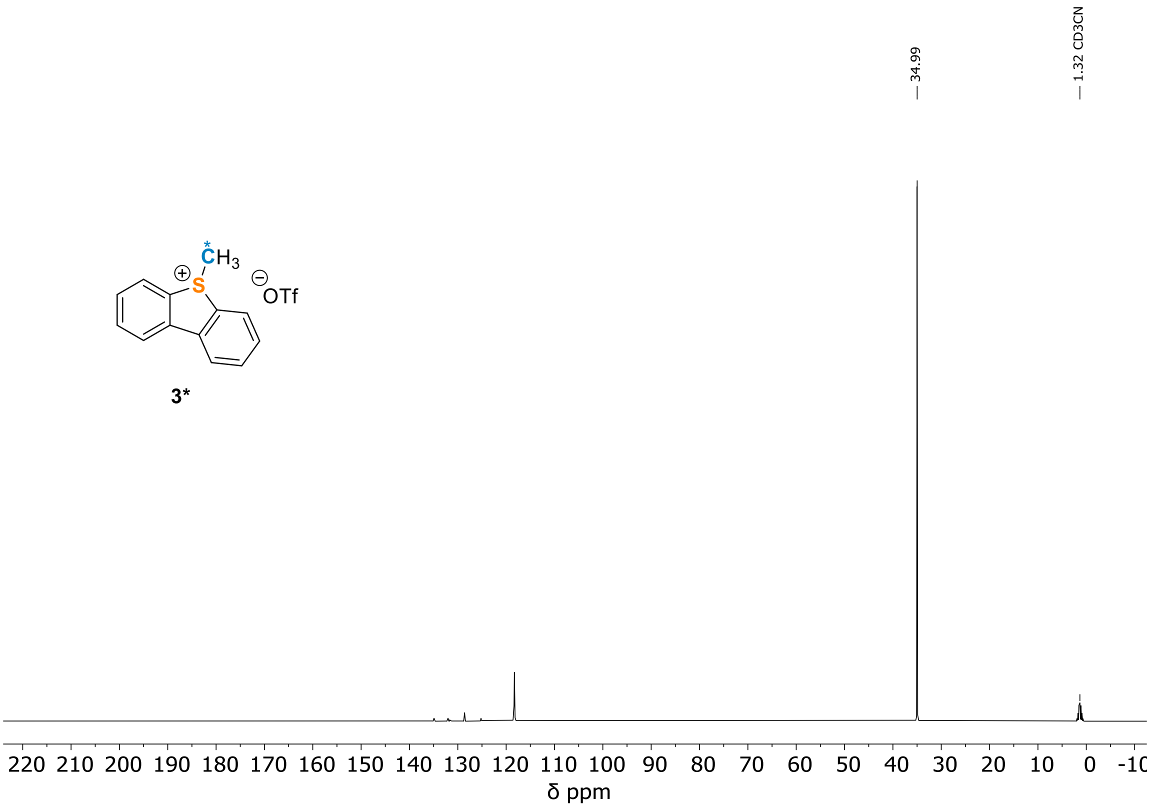

Compound **4**

^{1}H NMR (400 MHz, $CDCl_3$)

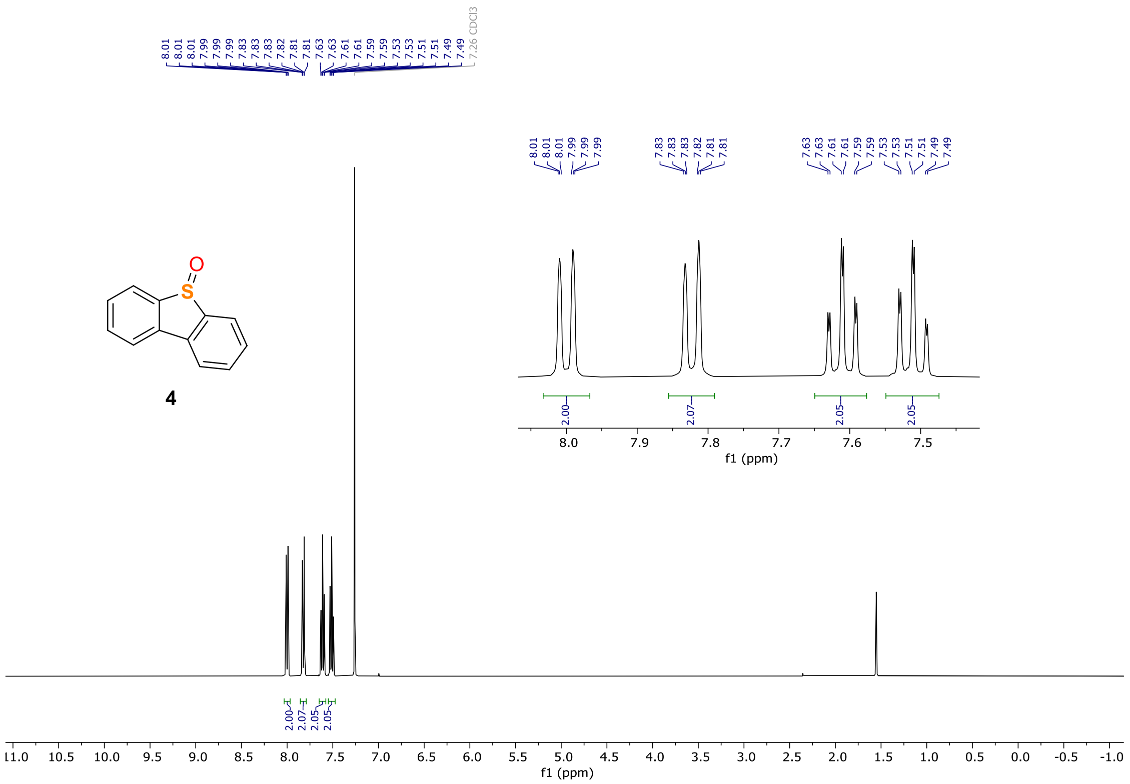

^{13}C{^{1}H} NMR (101 MHz, $CDCl_3$)

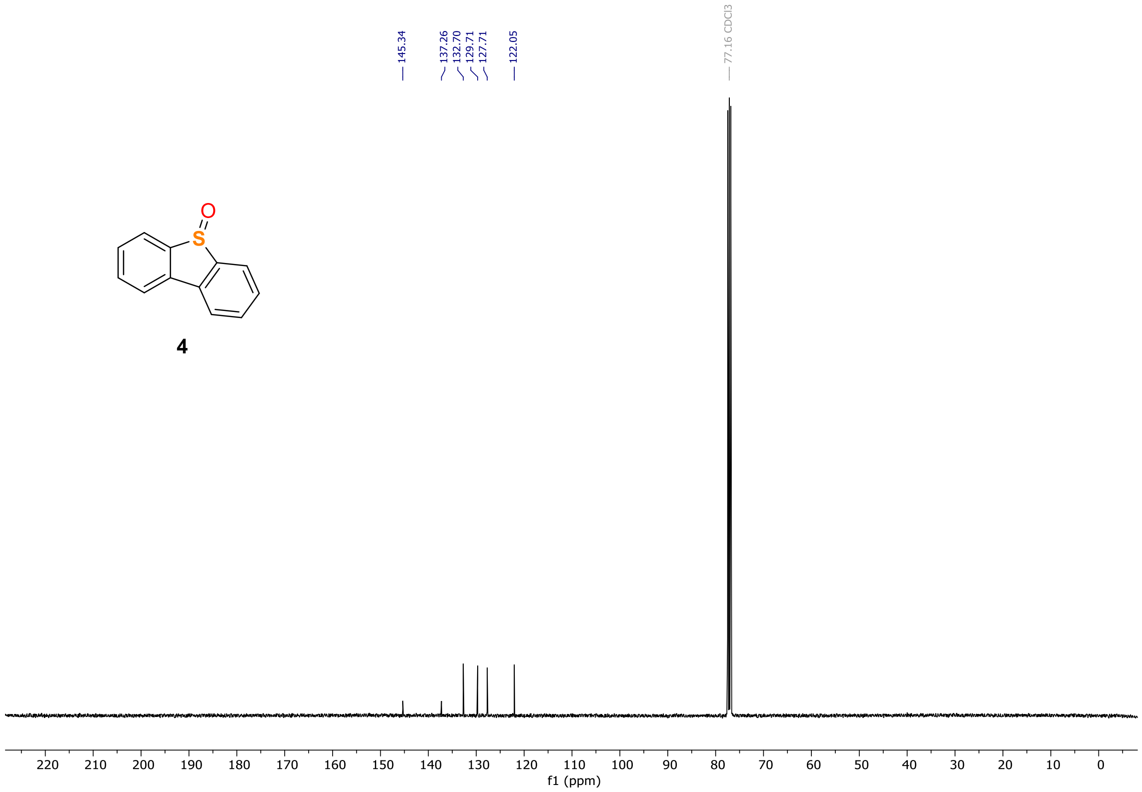

Compound **5**

^{1}H NMR (300 MHz, CD_3CN)

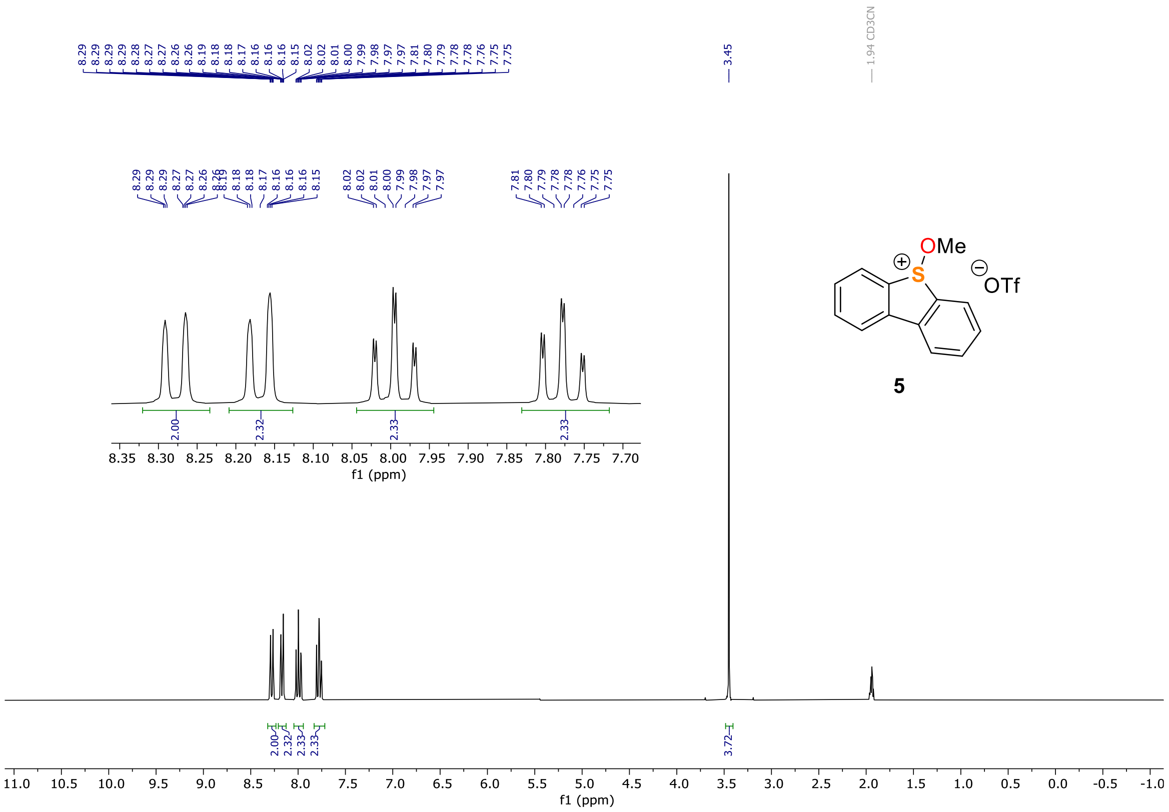

$^{13}C\{^{1}H\}$ NMR (101 MHz, CD_3CN)

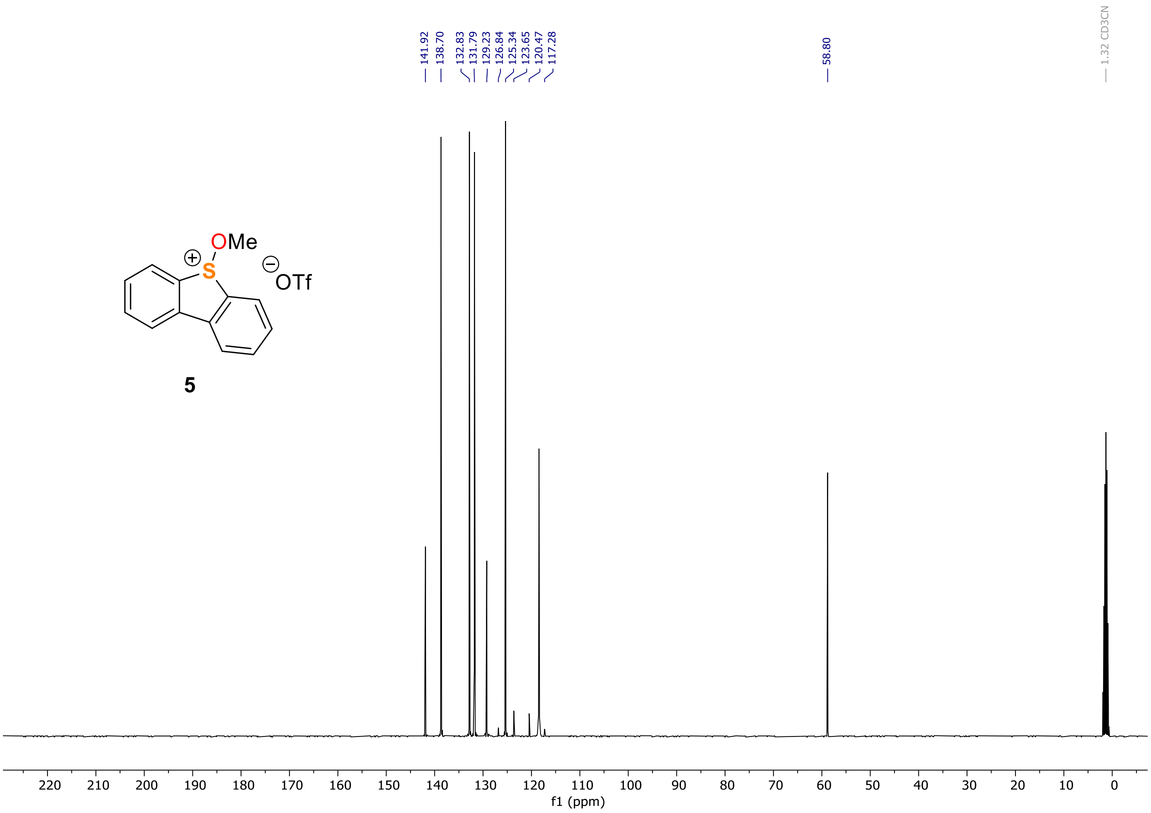

^{19}F NMR (377 MHz, CD_3CN)

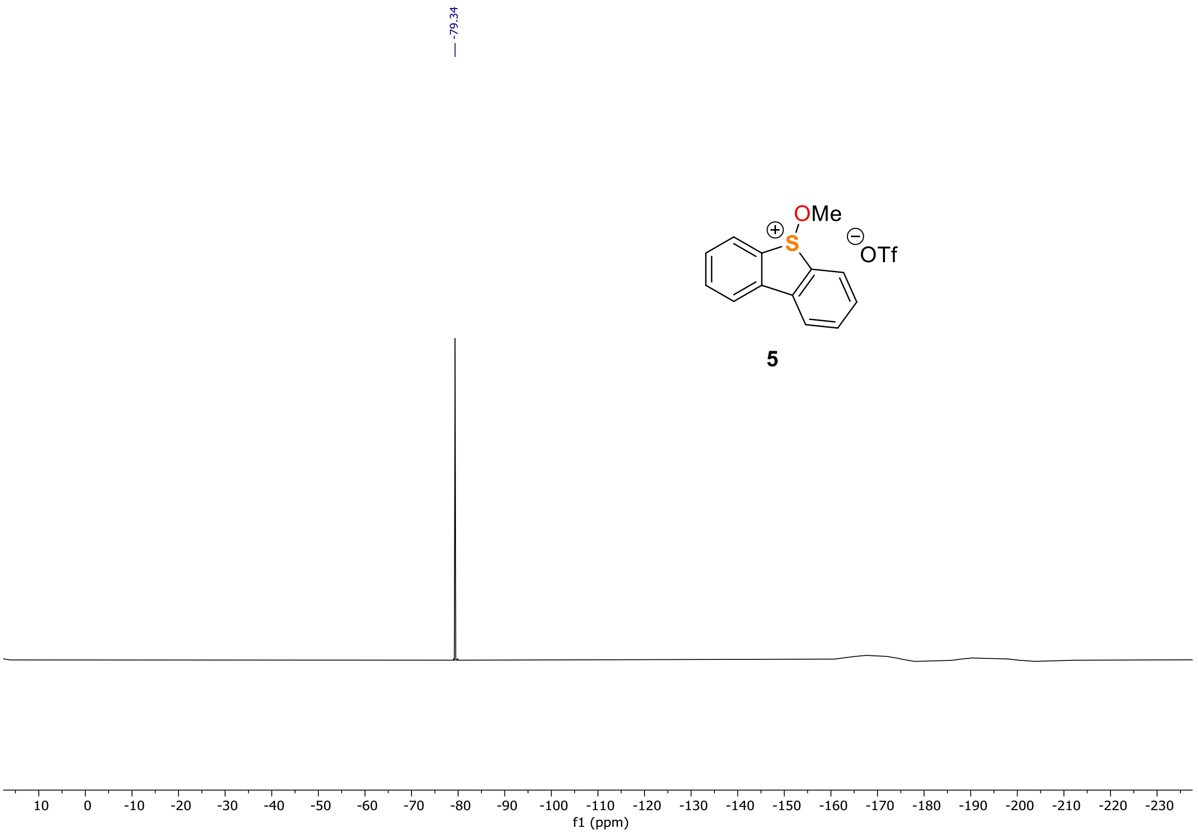

Compound **6i**

^{1}H NMR (400 MHz, $CDCl_3$)

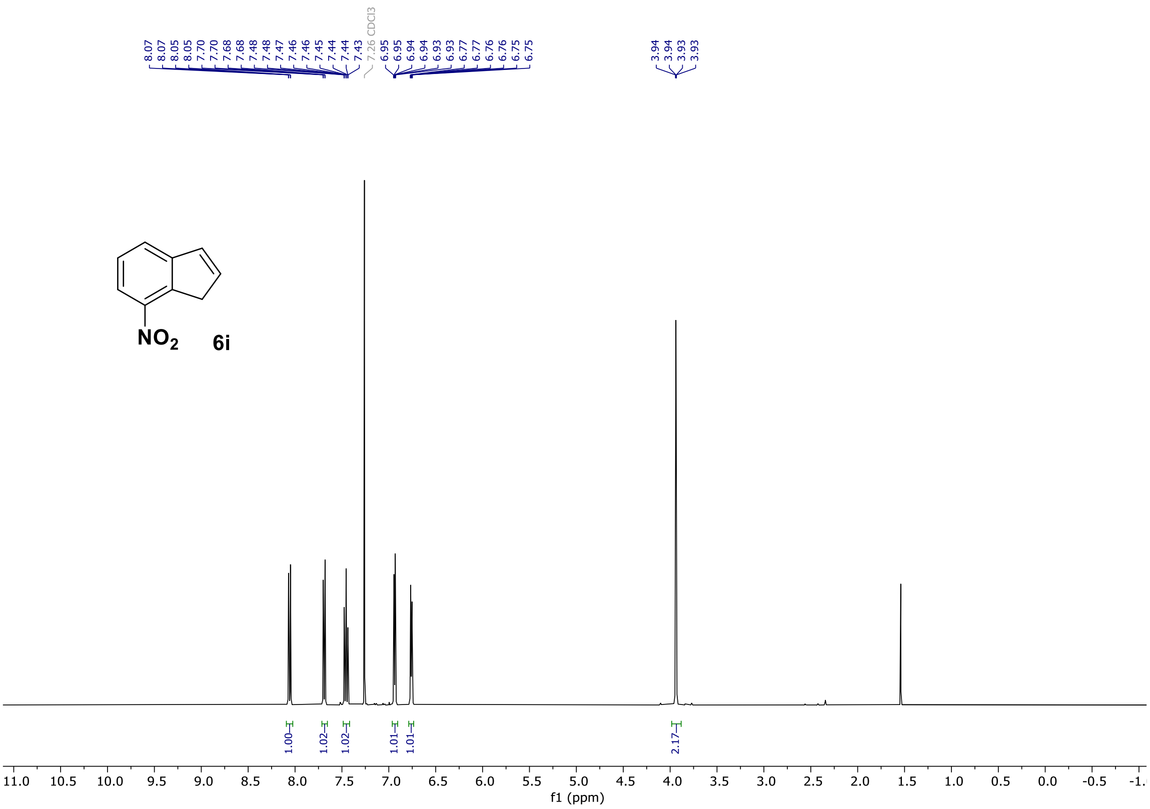

^{13}C{^{1}H} NMR (101 MHz, $CDCl_3$)

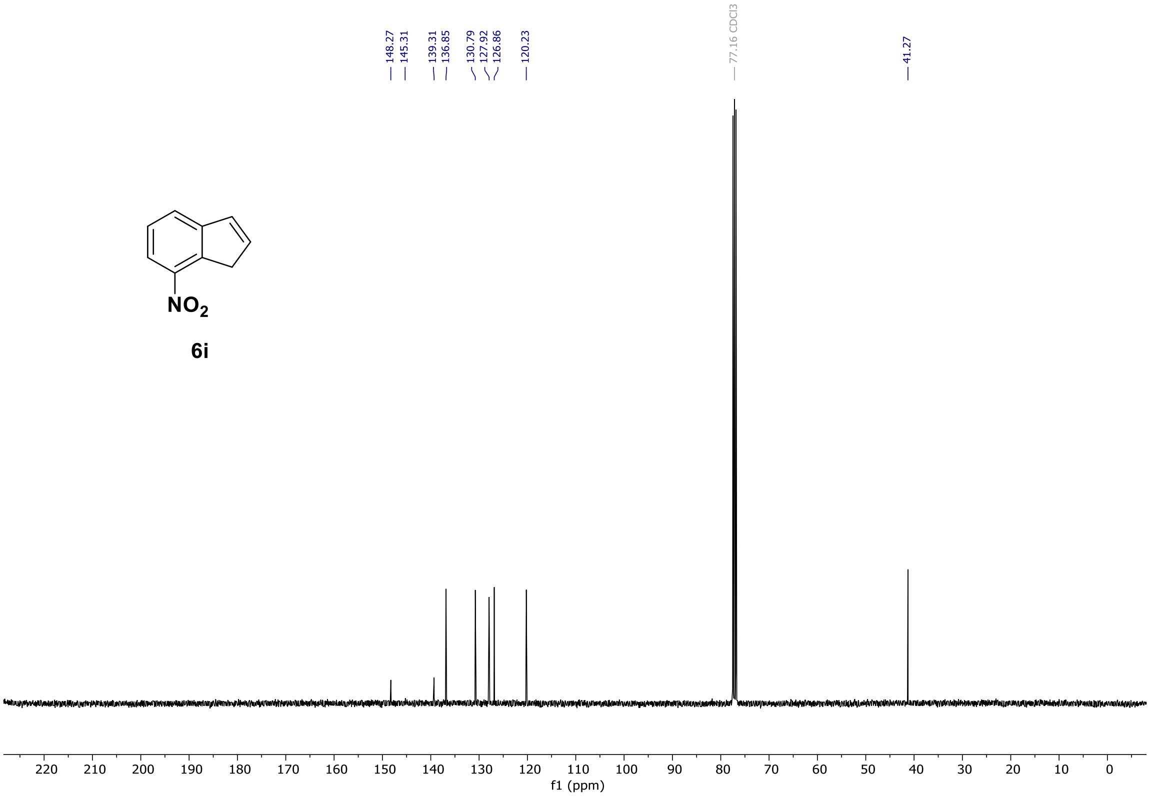

Compound **6k**

^{1}H NMR (400 MHz, $CDCl_3$)

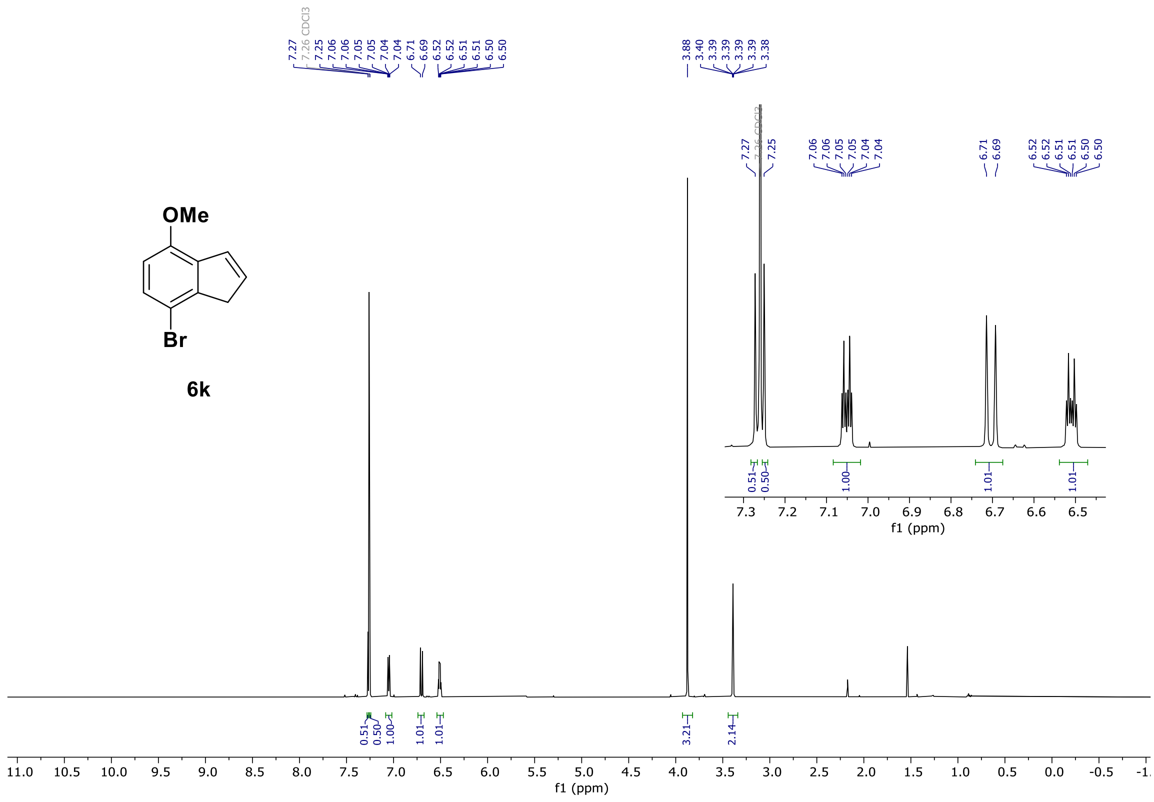

^{13}C{^{1}H} NMR (101 MHz, $CDCl_3$)

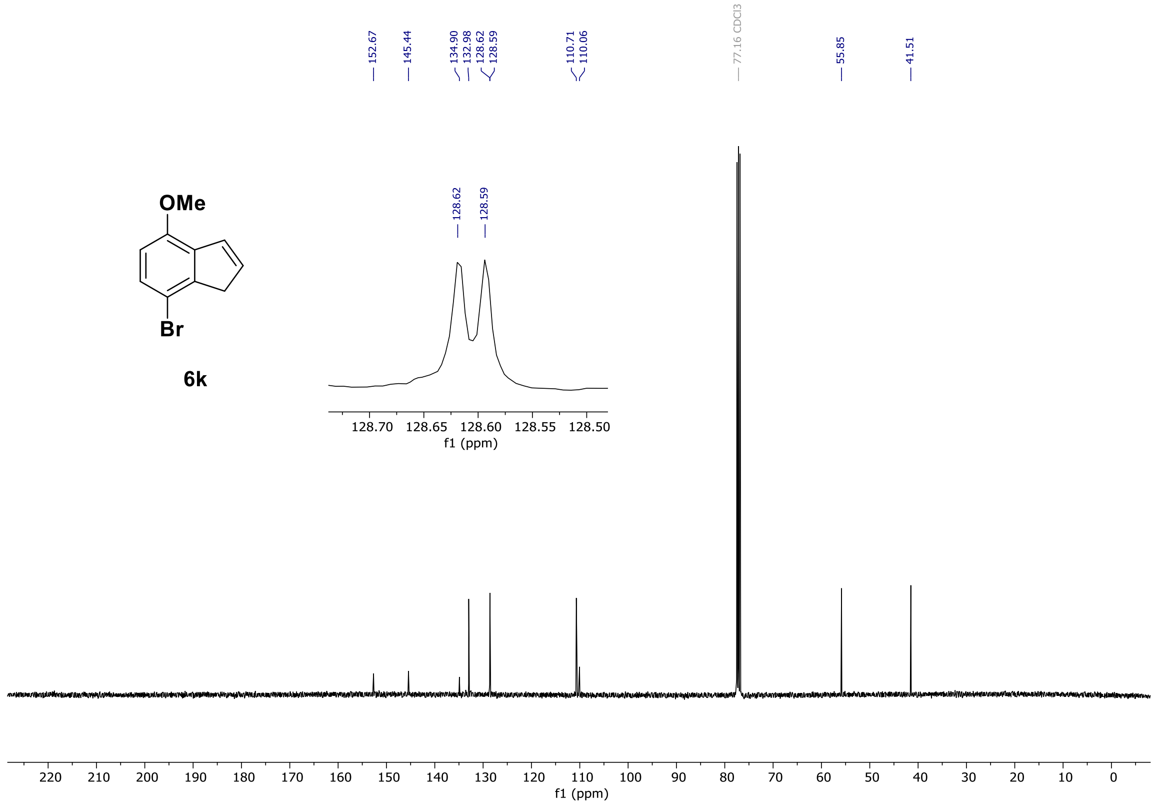

Compound **6n**

^{1}H NMR (400 MHz, $CDCl_3$)

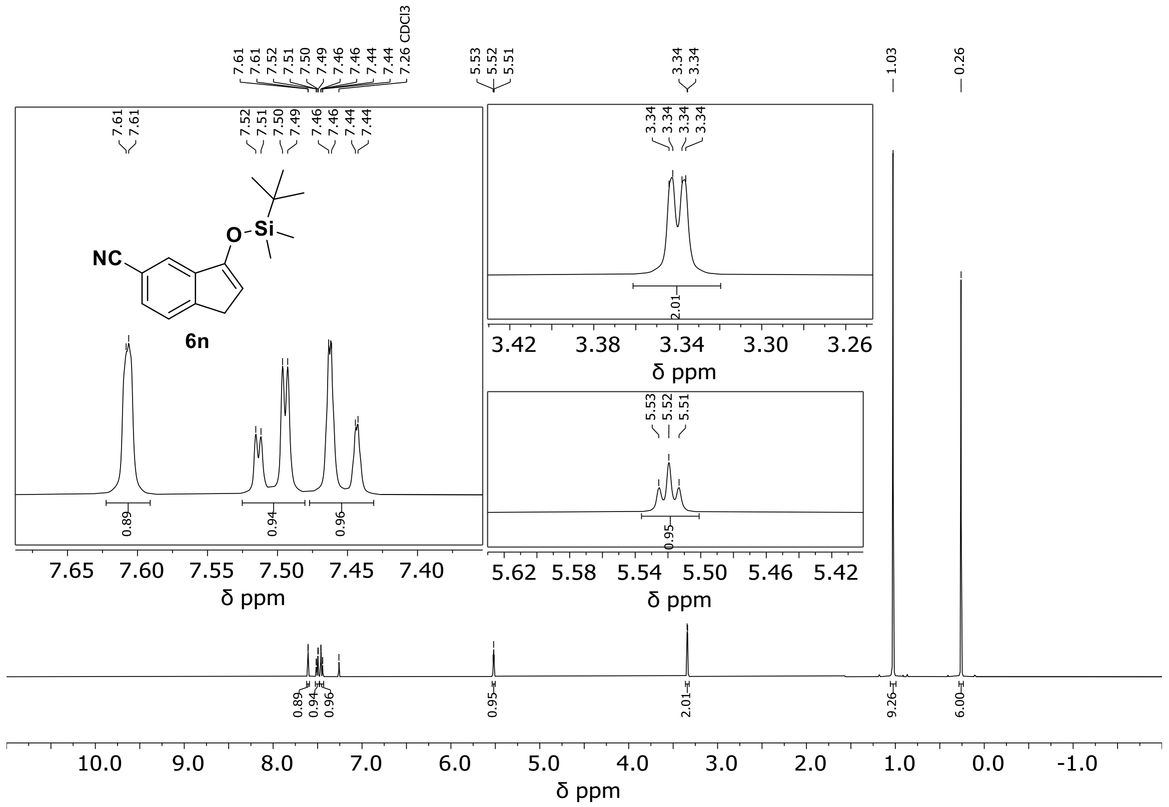

^{13}C{^{1}H} NMR (101 MHz, $CDCl_3$)

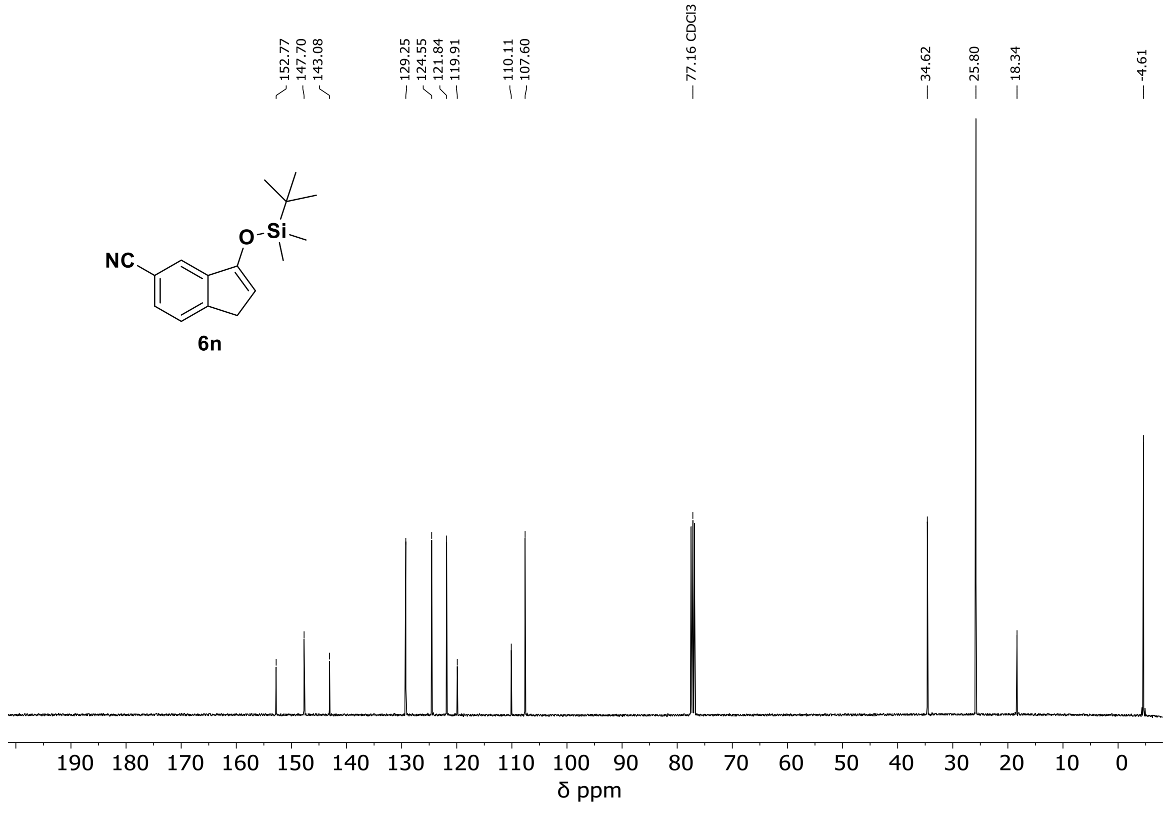

Compound **6t**

^{1}H NMR (400 MHz, $CDCl_3$)

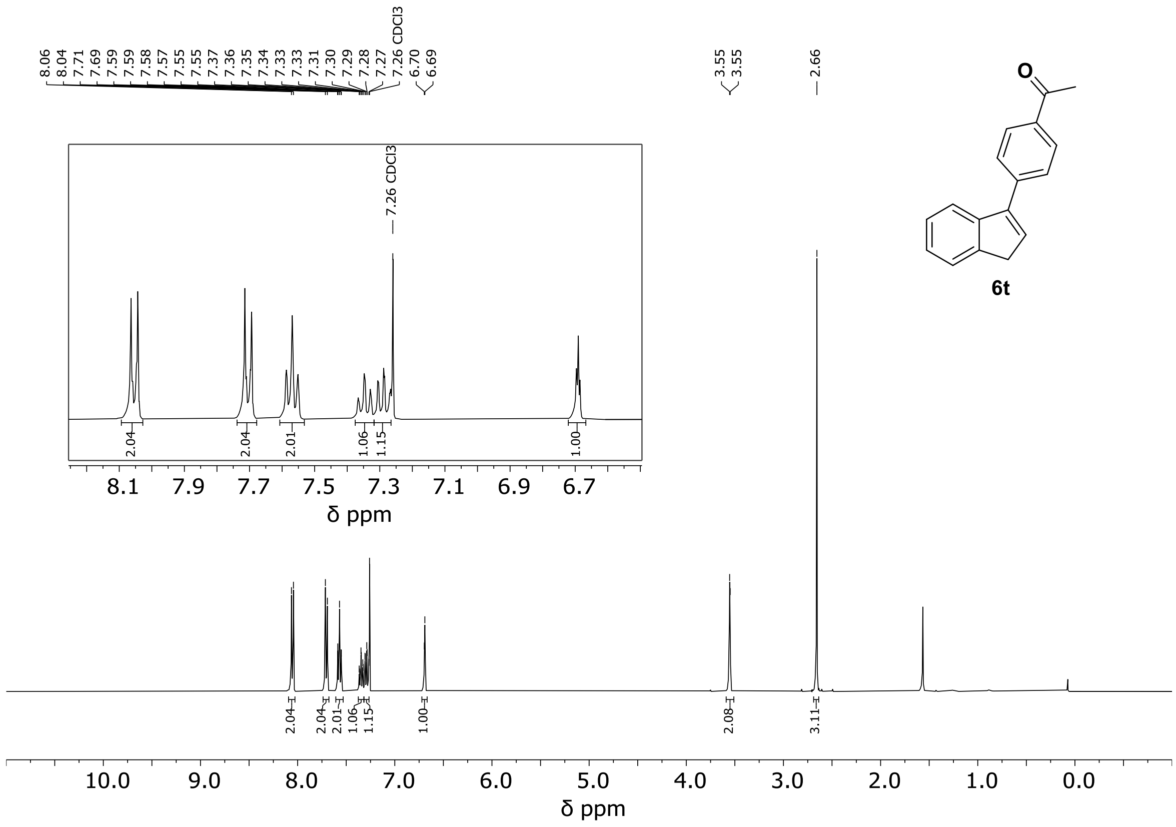

^{13}C{^{1}H} NMR (101 MHz, $CDCl_3$)

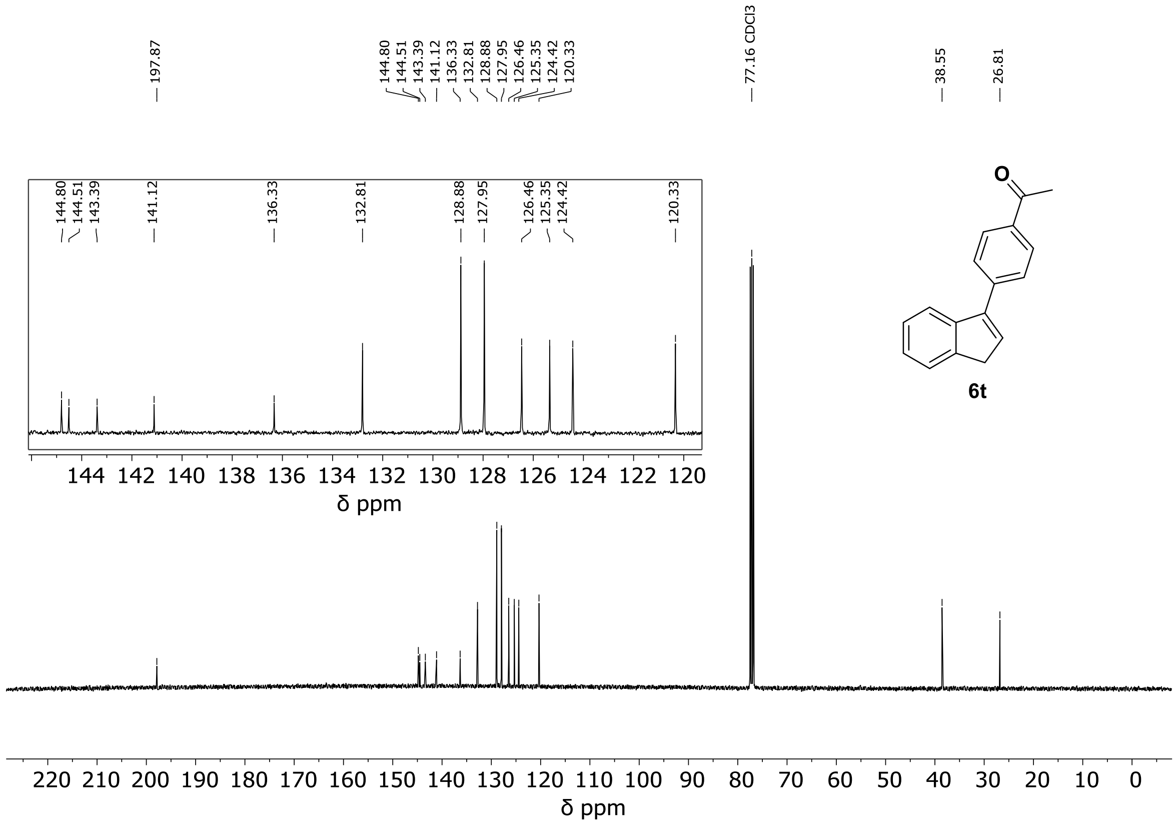

Compound **7a**

^{1}H NMR (400 MHz, $CDCl_3$)

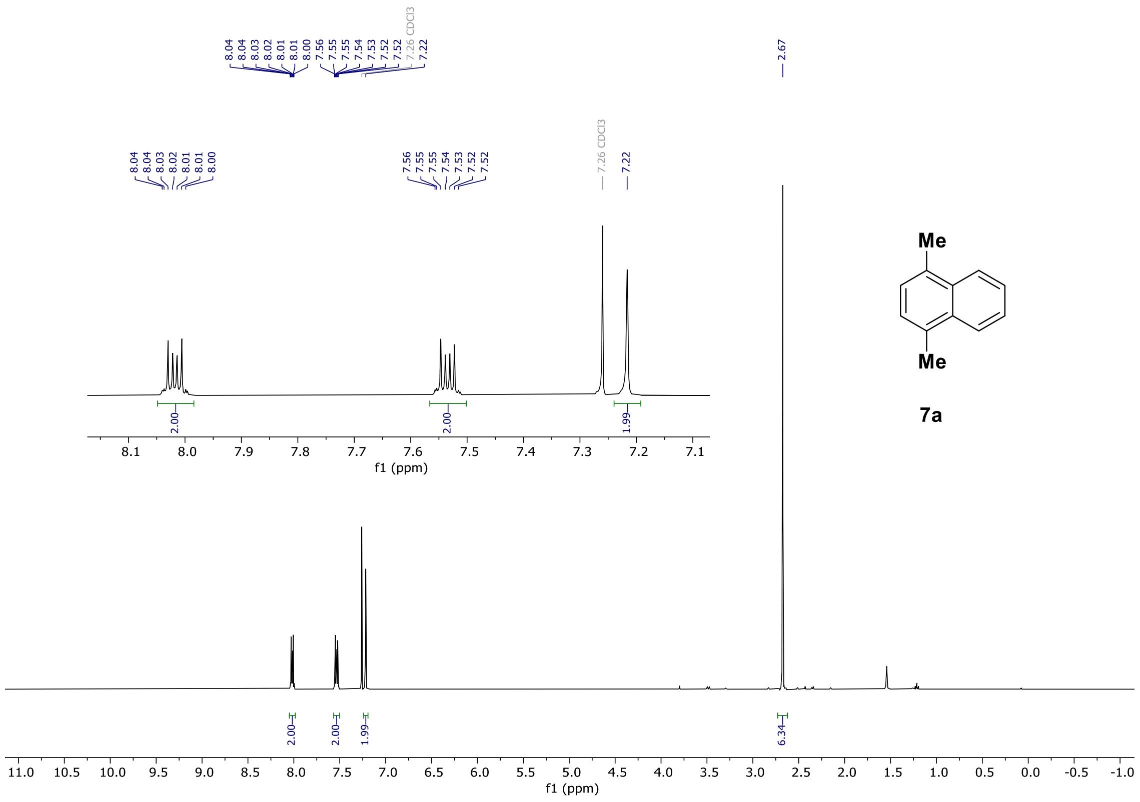

$^{13}C\{^{1}H\}$ NMR (101 MHz, $CDCl_3$)

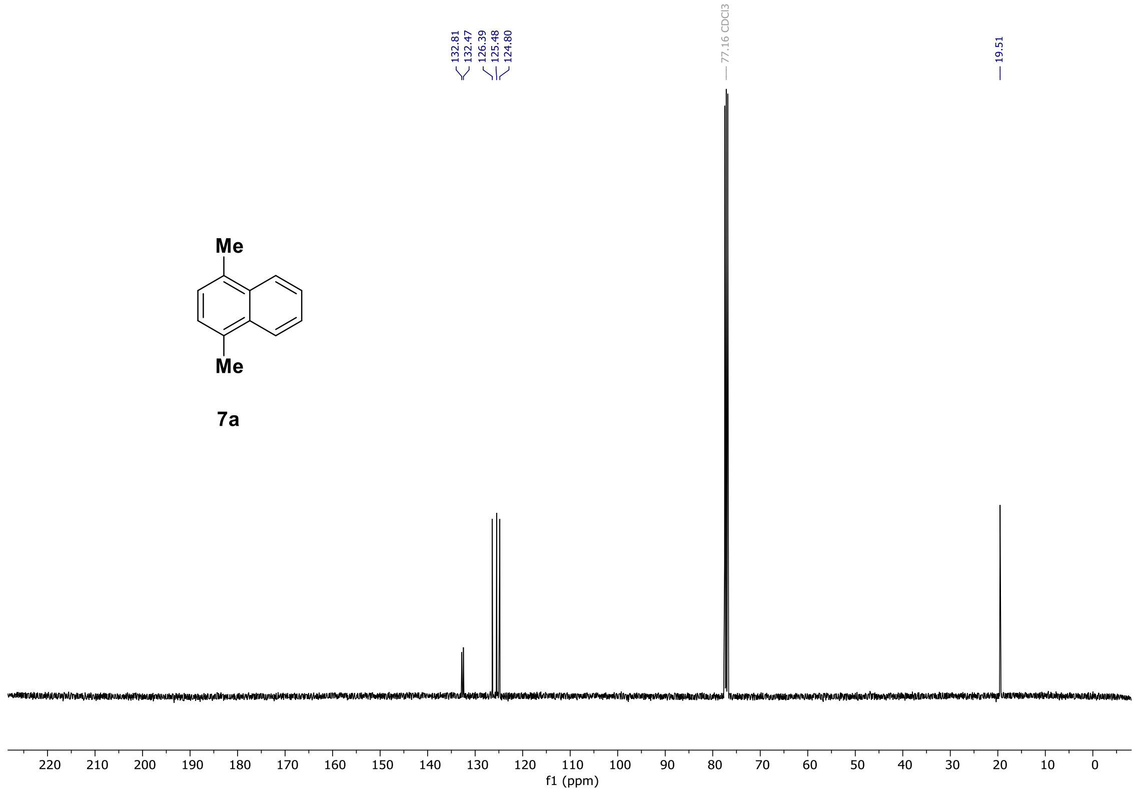

Compound **7b**

^{1}H NMR (400 MHz, $CDCl_3$)

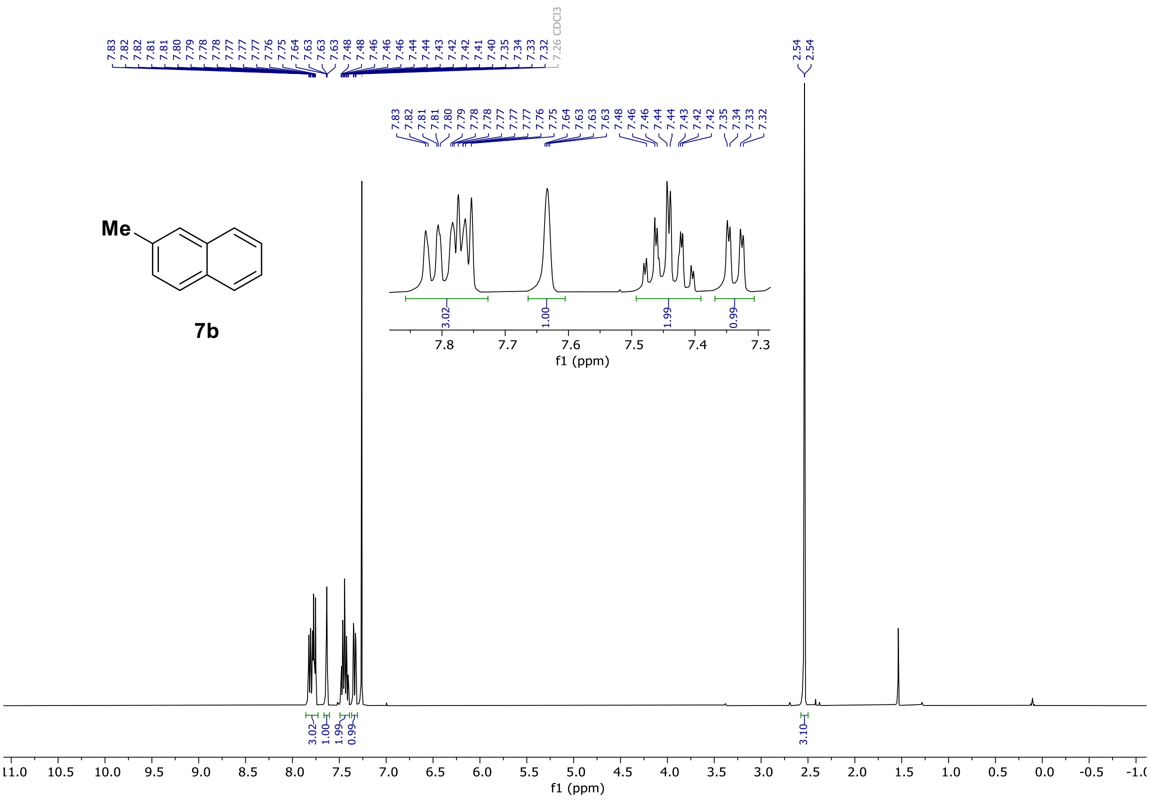

$^{13}C\{^{1}H\}$ NMR (101 MHz, $CDCl_3$)

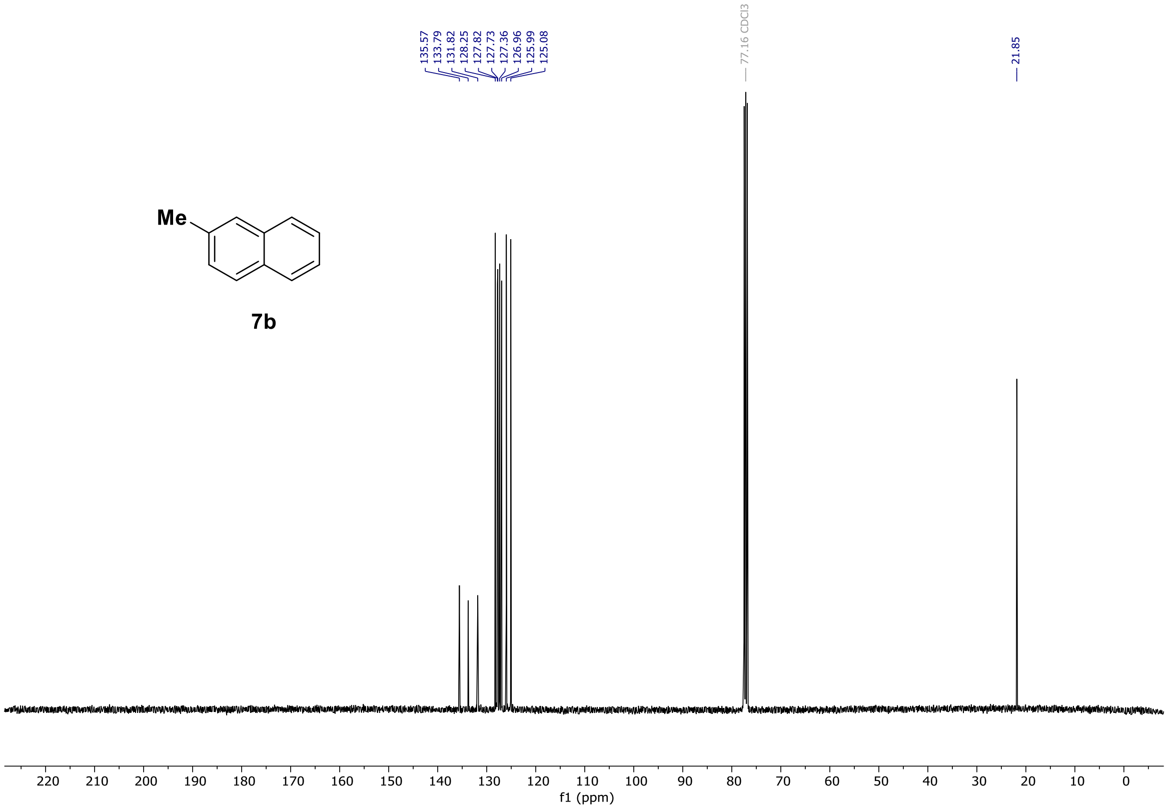

Compound **7c**

^{1}H NMR (400 MHz, $CDCl_3$)

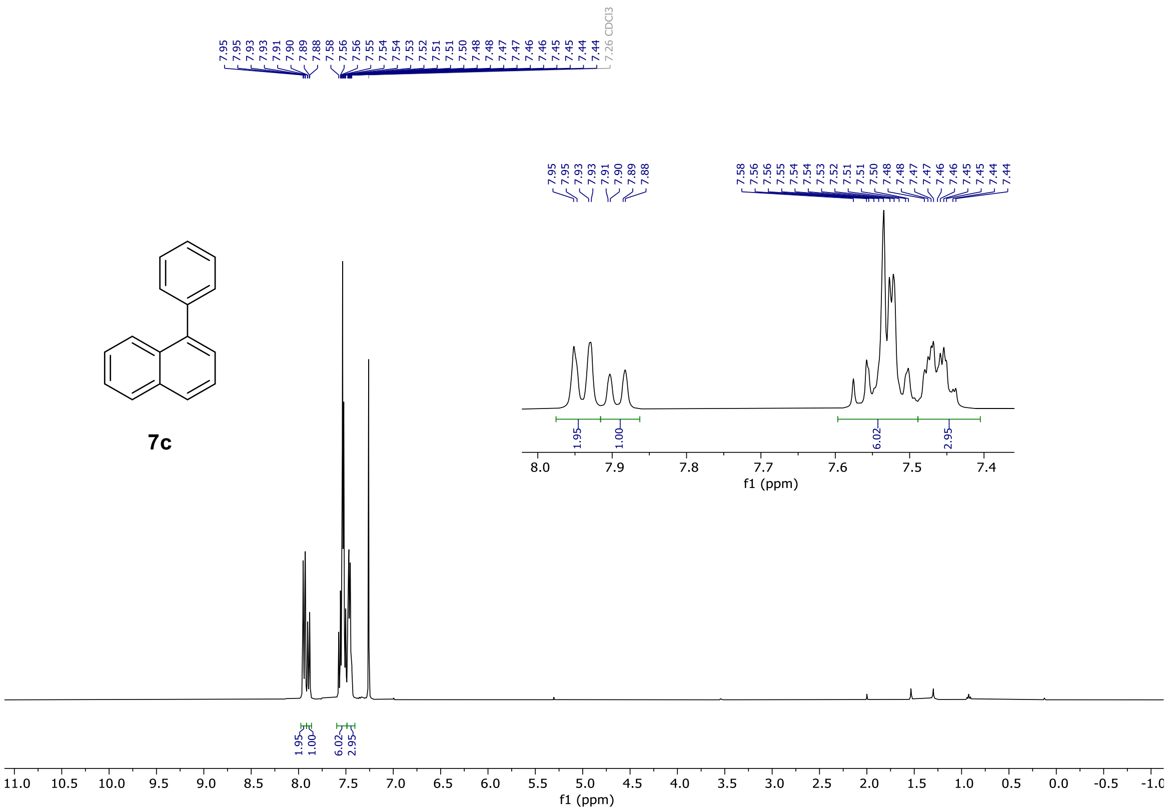

^{13}C{^{1}H} NMR (101 MHz, $CDCl_3$)

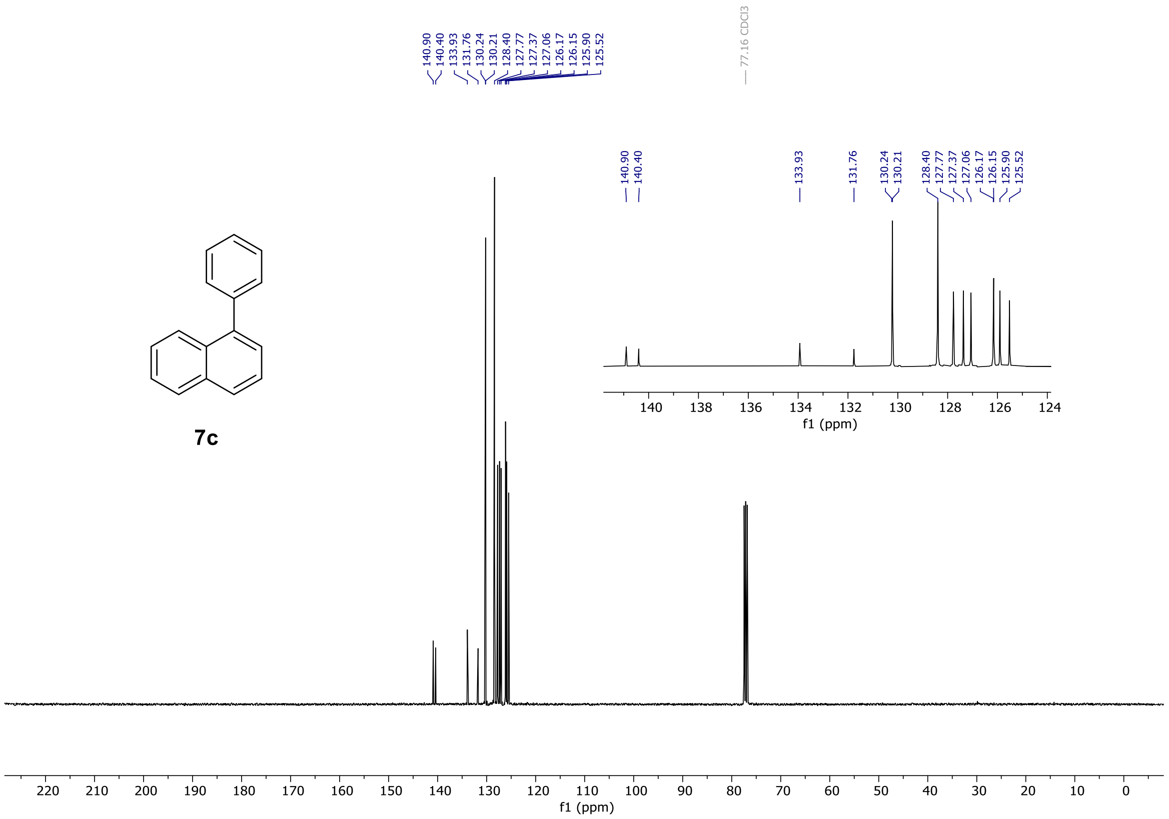

Compound **7c***

^{1}H NMR (500 MHz, $CDCl_3$)

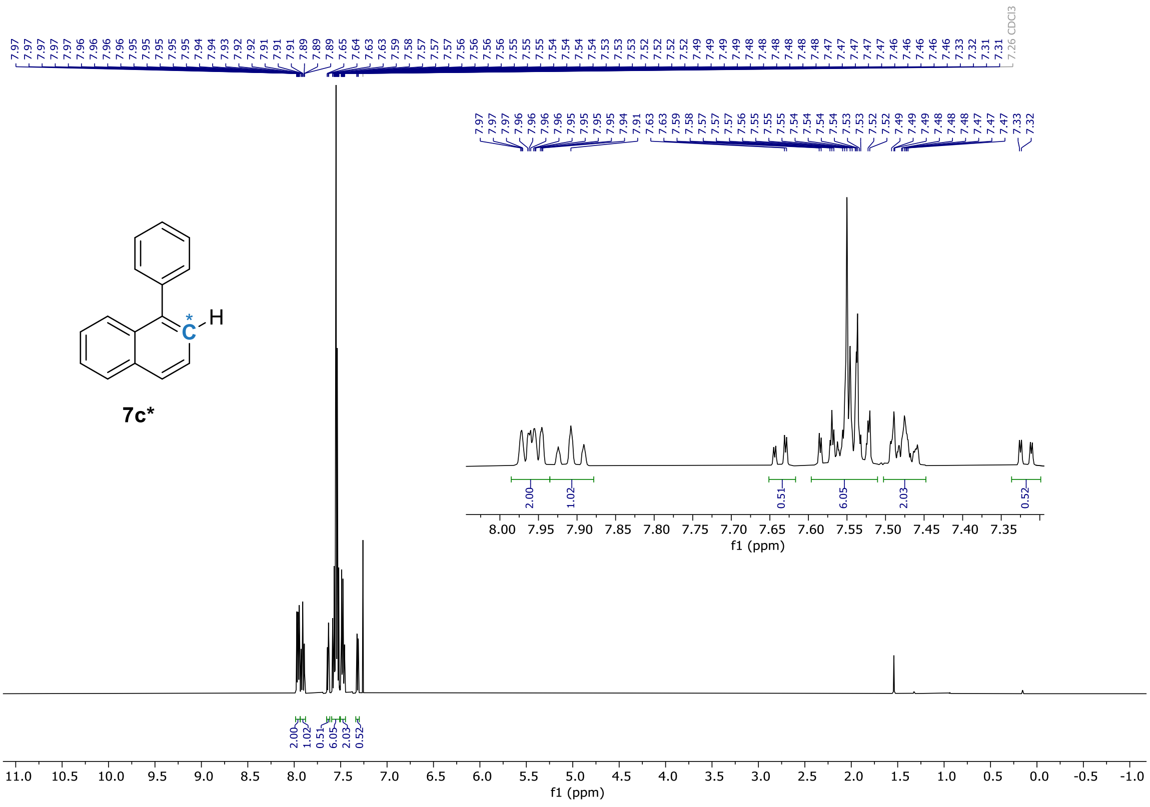

^{13}C{^{1}H} NMR (101 MHz, $CDCl_3$)

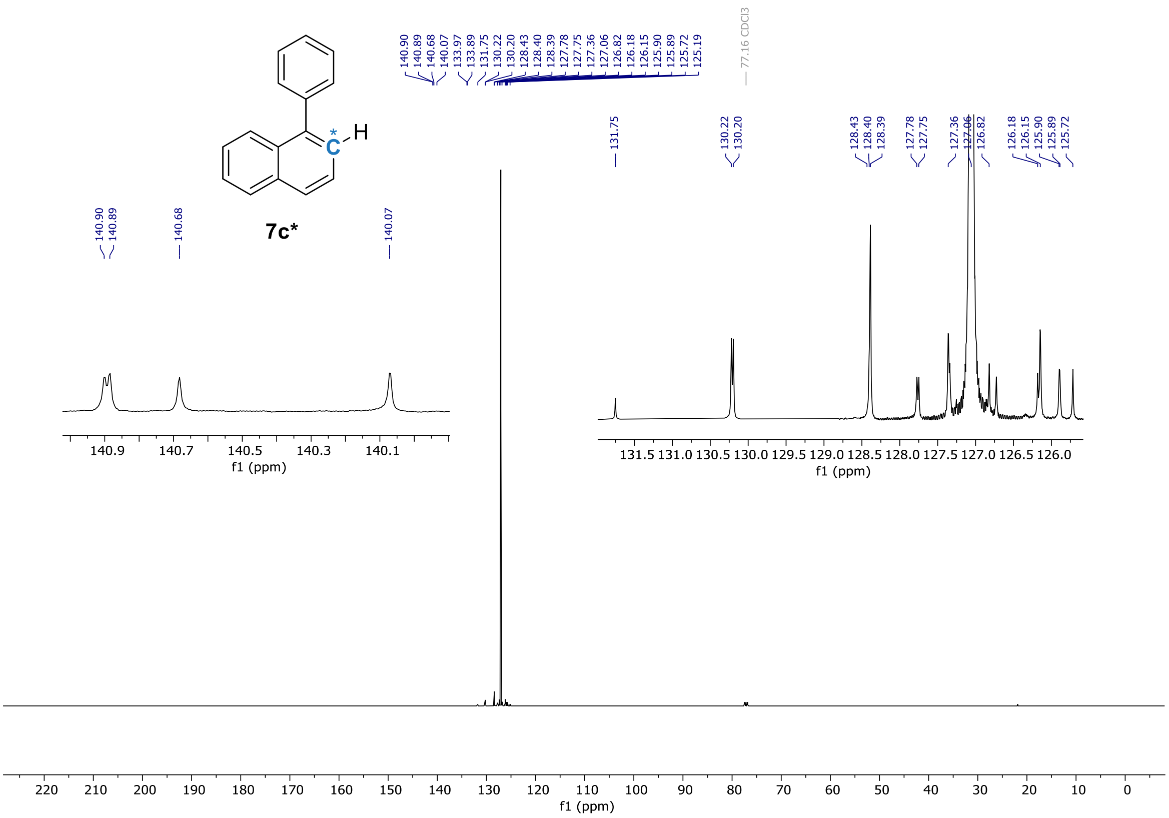

^{13}C NMR (126 MHz, $CDCl_3$)

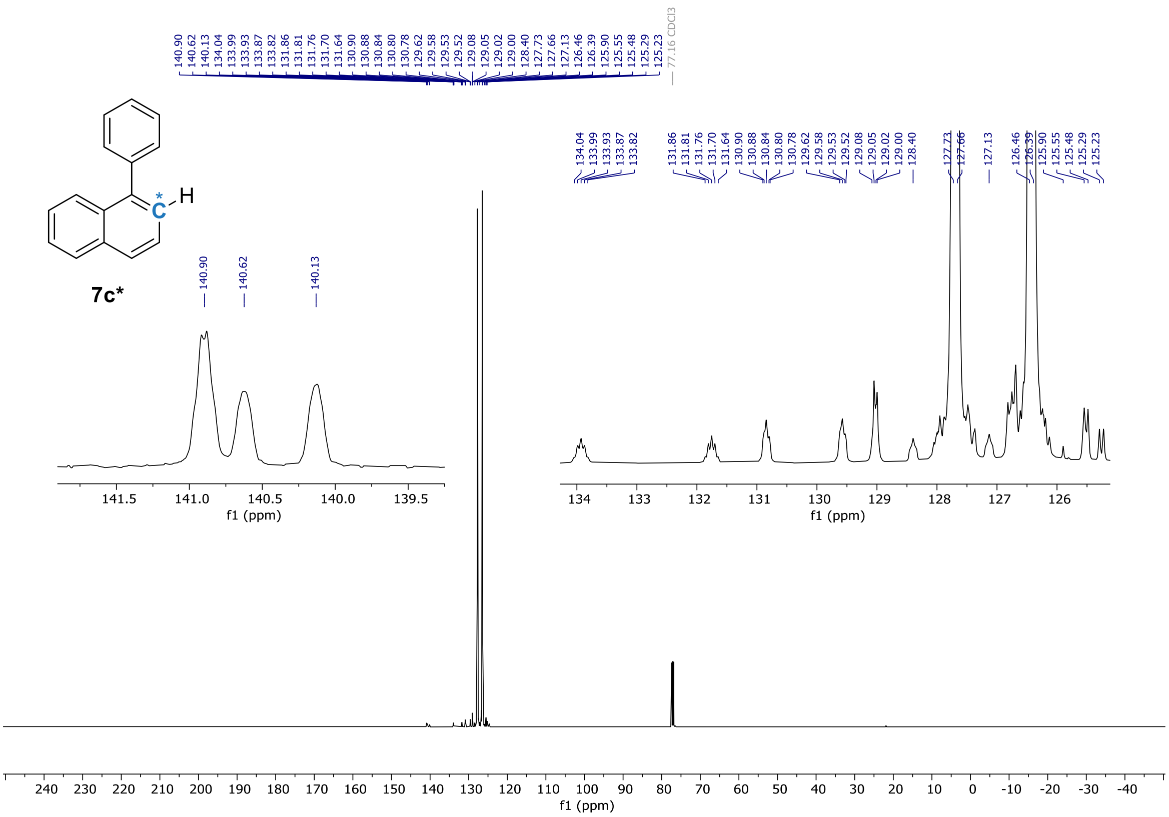

Compound **7d**

^{1}H NMR (400 MHz, $CDCl_3$)

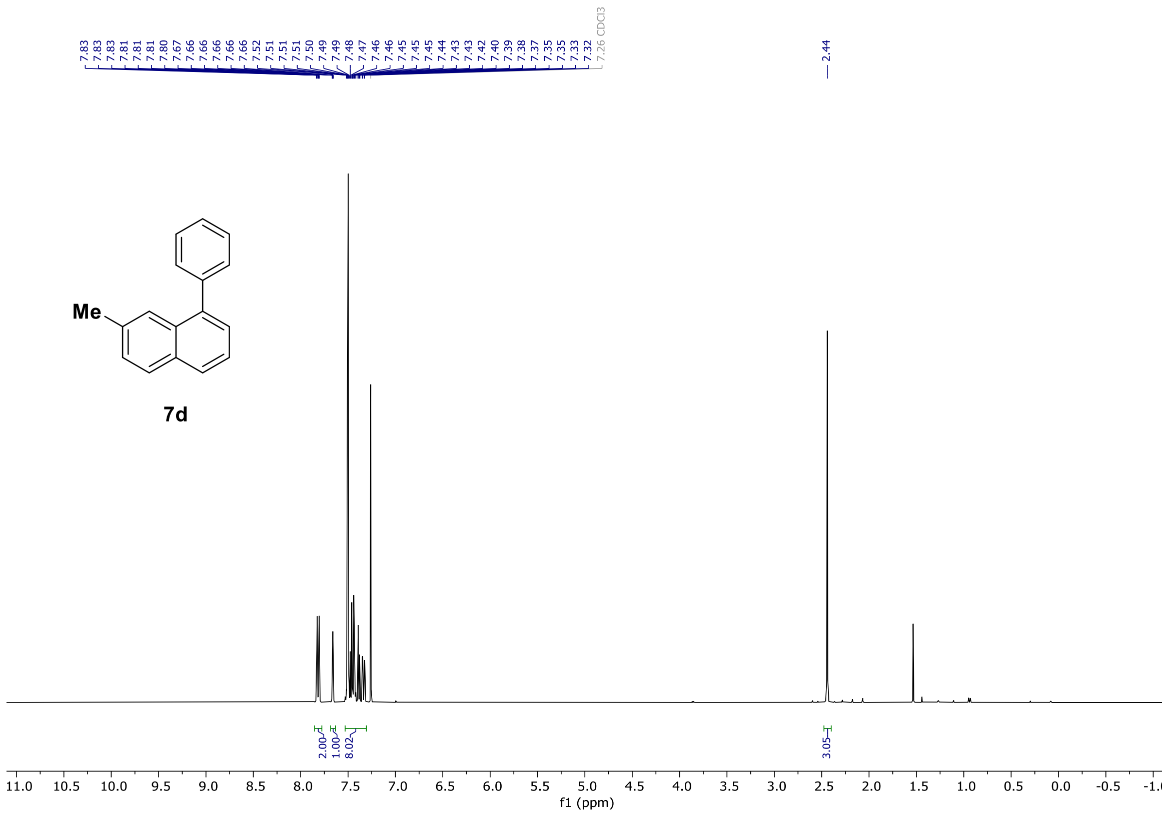

$^{13}C\{^{1}H\}$ NMR (101 MHz, $CDCl_3$)

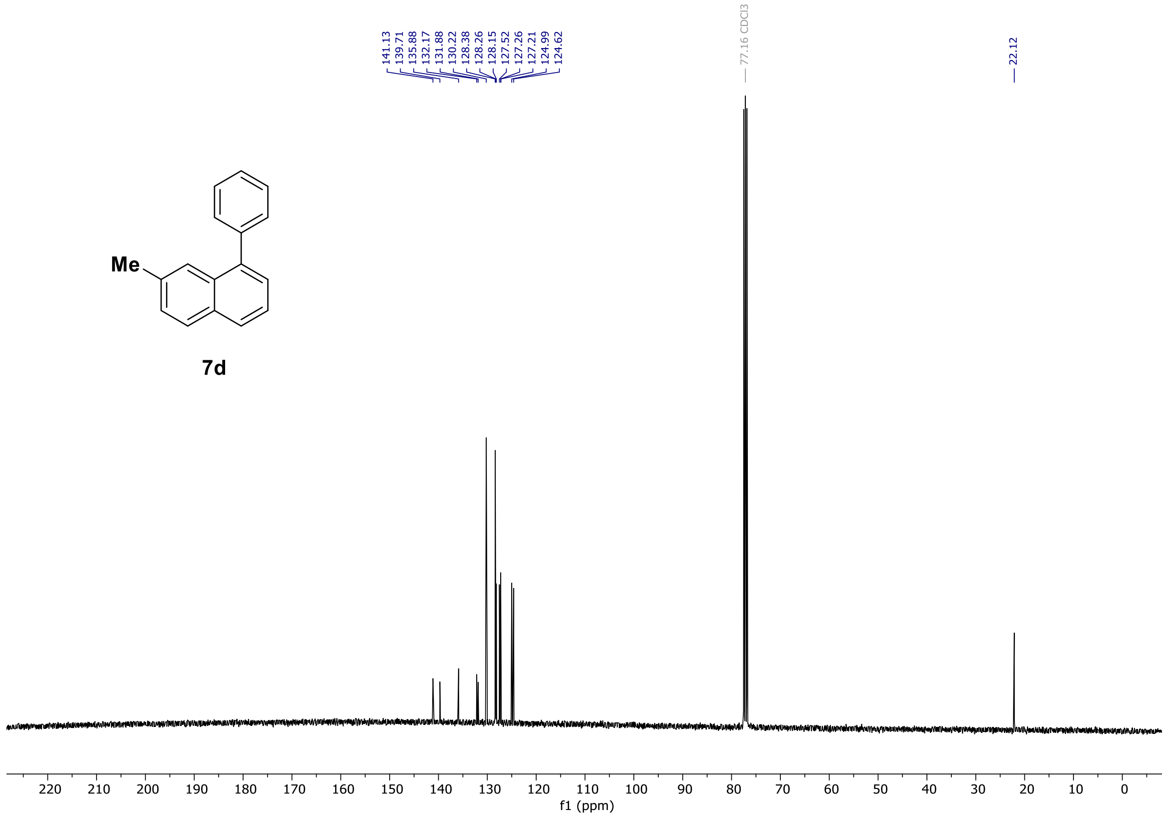

Compound **7e**

^{1}H NMR (400 MHz, $CDCl_3$)

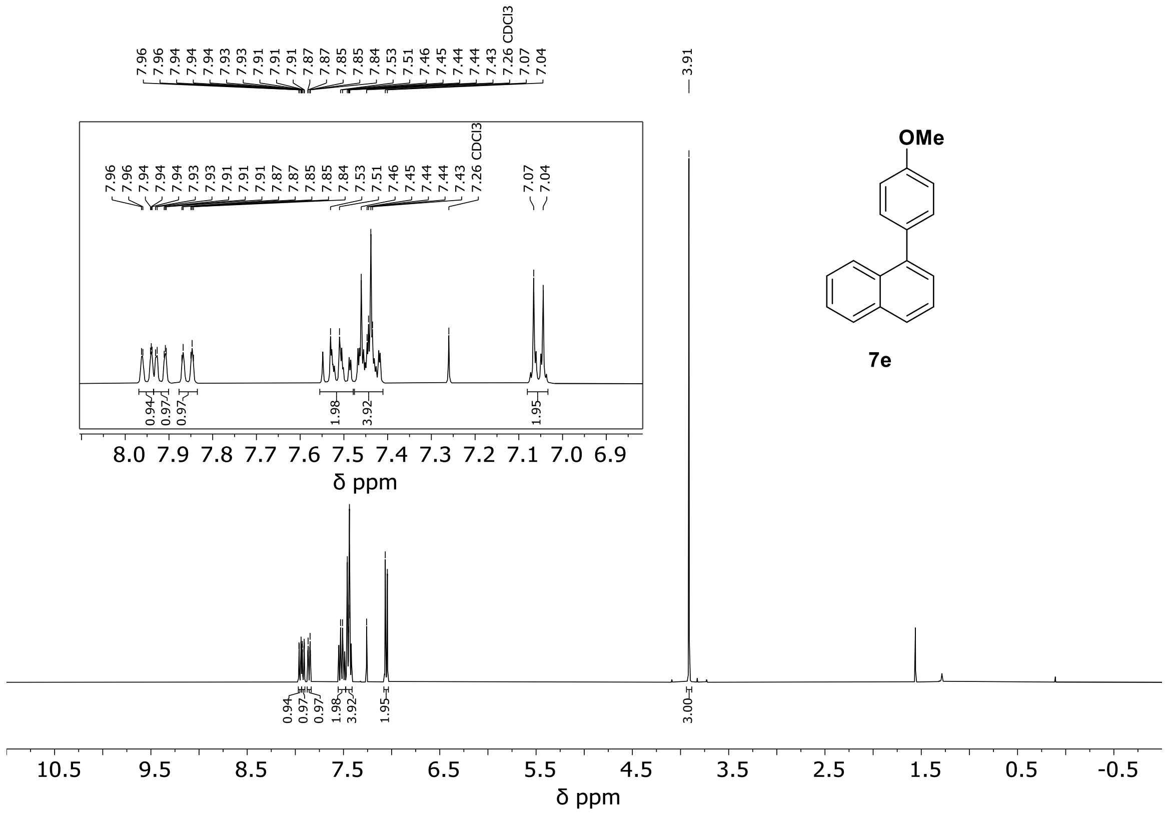

^{13}C{^{1}H} NMR (101 MHz, $CDCl_3$)

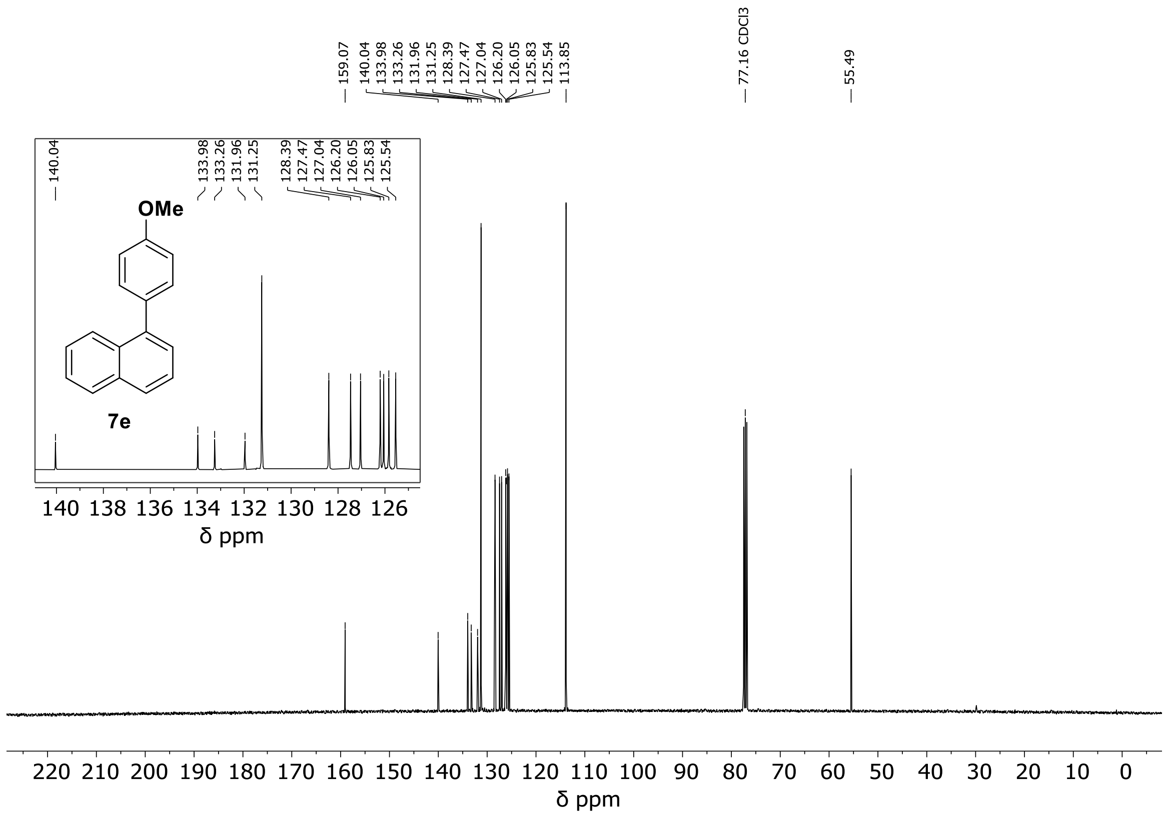

Compound **7e***

^{1}H NMR (500 MHz, $CDCl_3$)

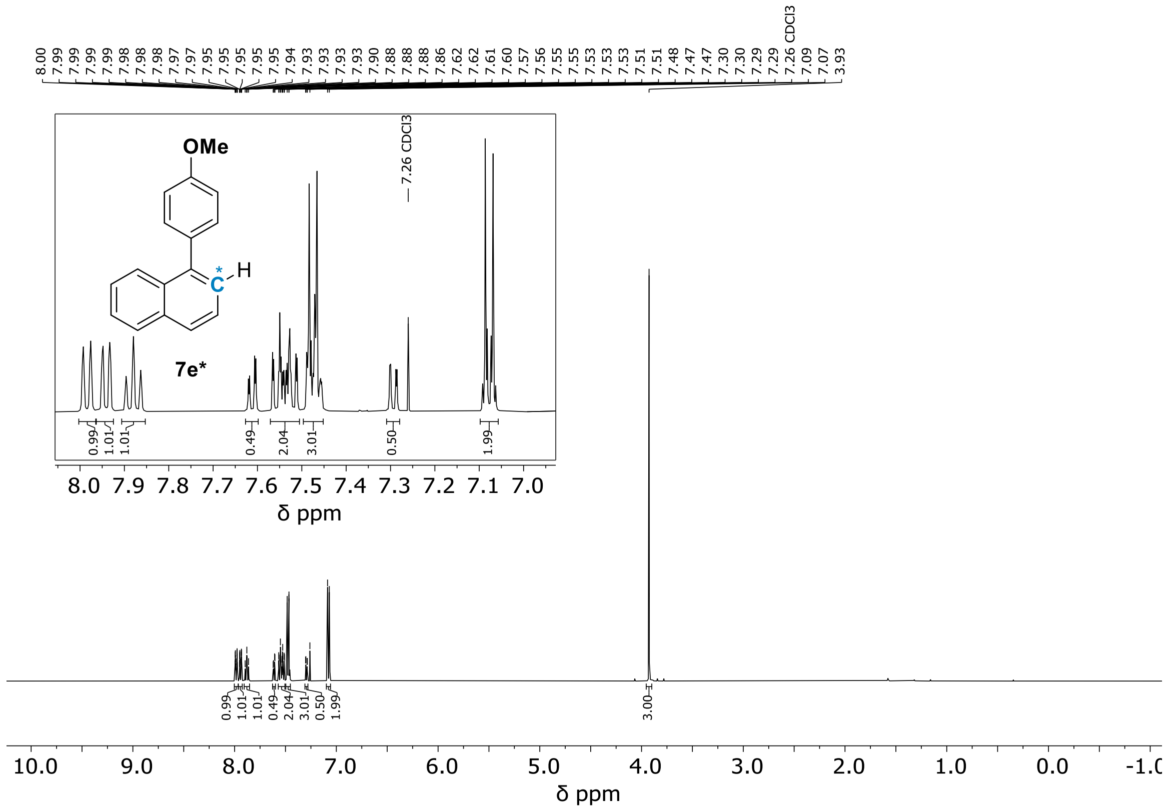

$^{13}C\{^{1}H\}$ NMR (126 MHz, $CDCl_3$)

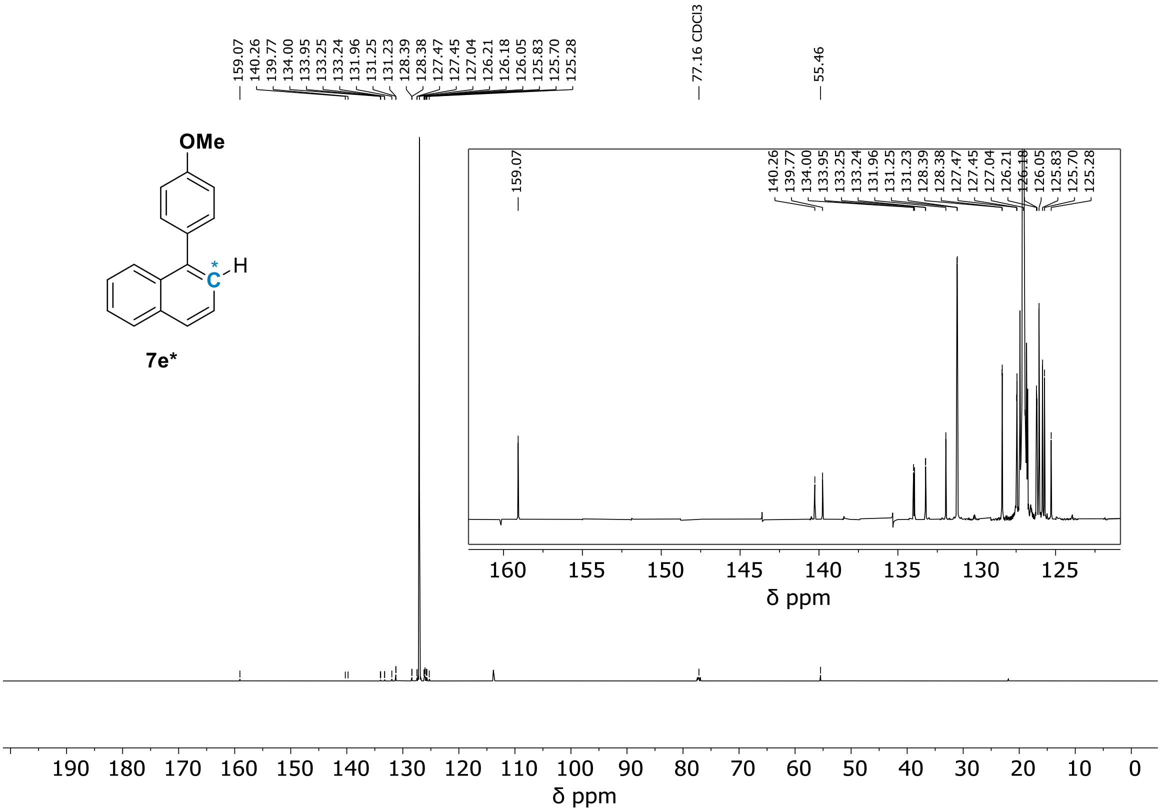

Compound **7f**

^{1}H NMR (400 MHz, $CDCl_3$)

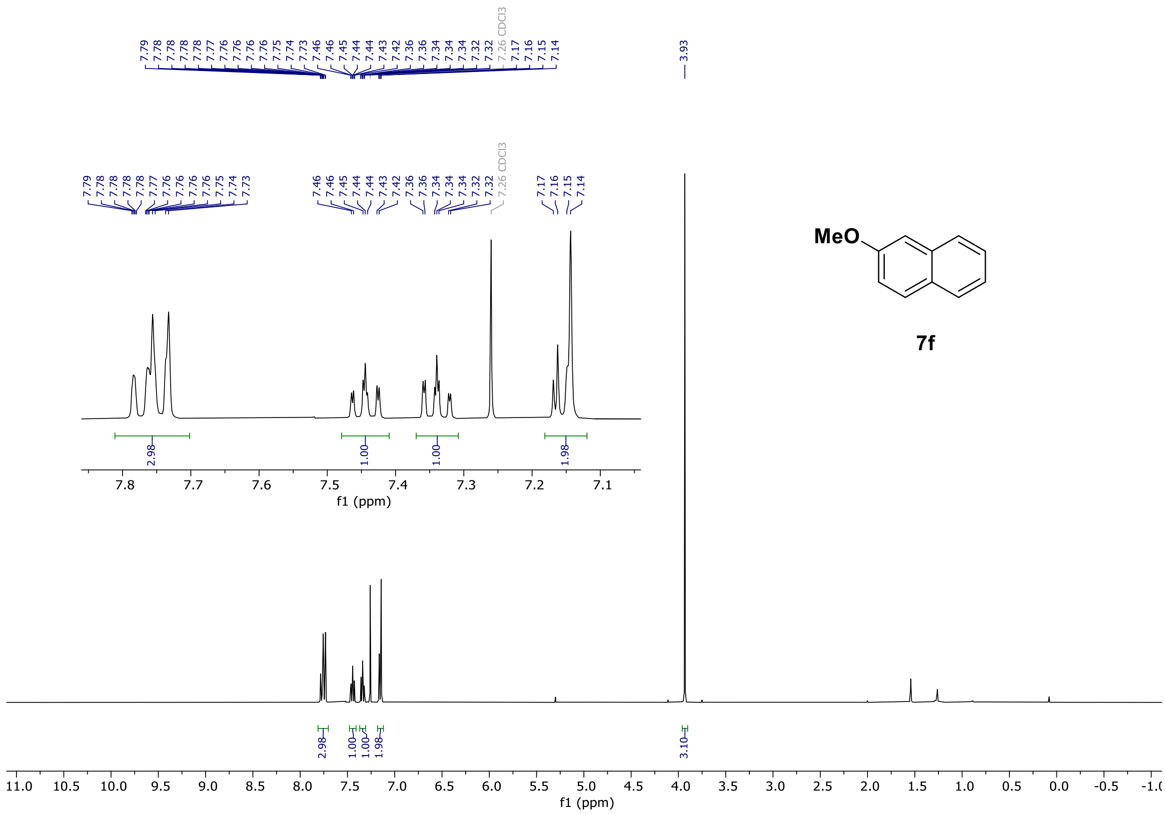

$^{13}C\{^{1}H\}$ NMR (101 MHz, $CDCl_3$)

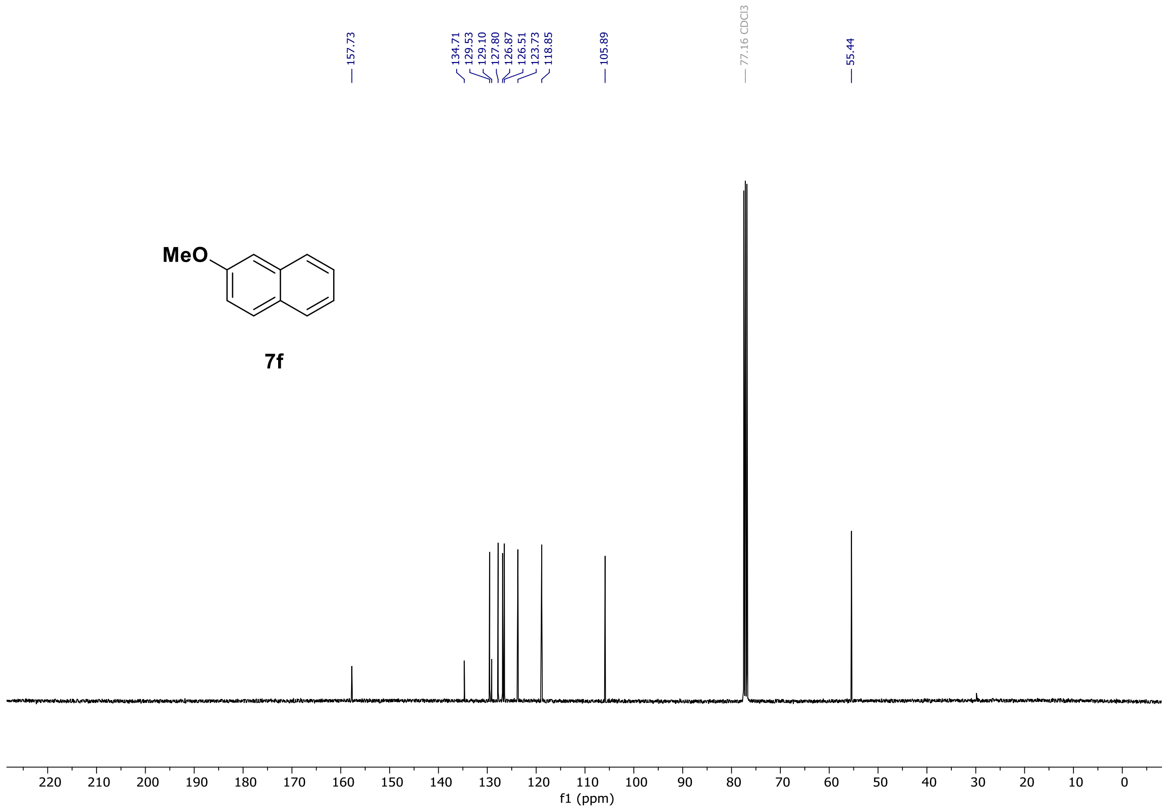

Compound **7g**

^{1}H NMR (300 MHz, $CDCl_3$)

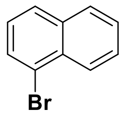

7g

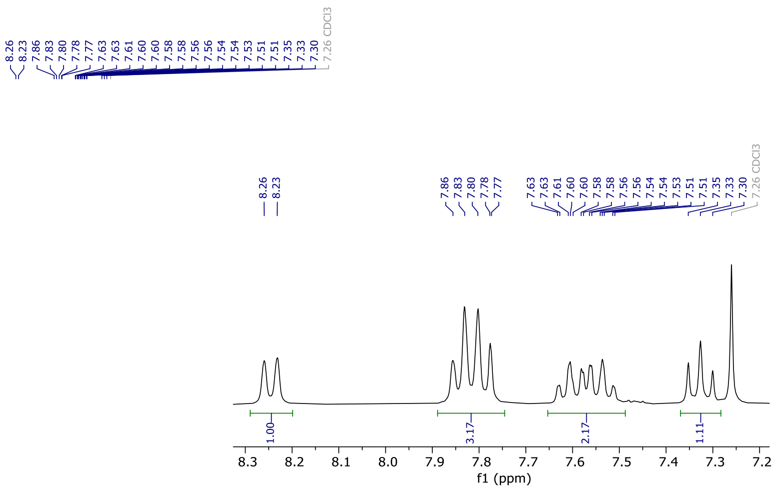

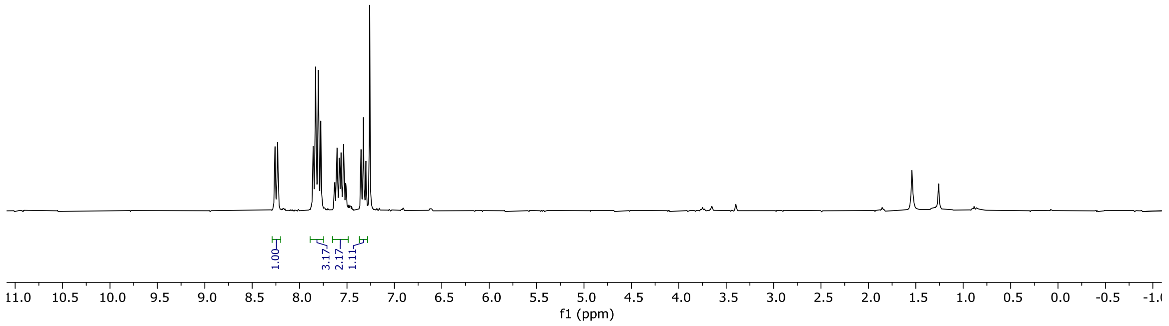

$^{13}C\{^{1}H\}$ NMR (101 MHz, $CDCl_3$)

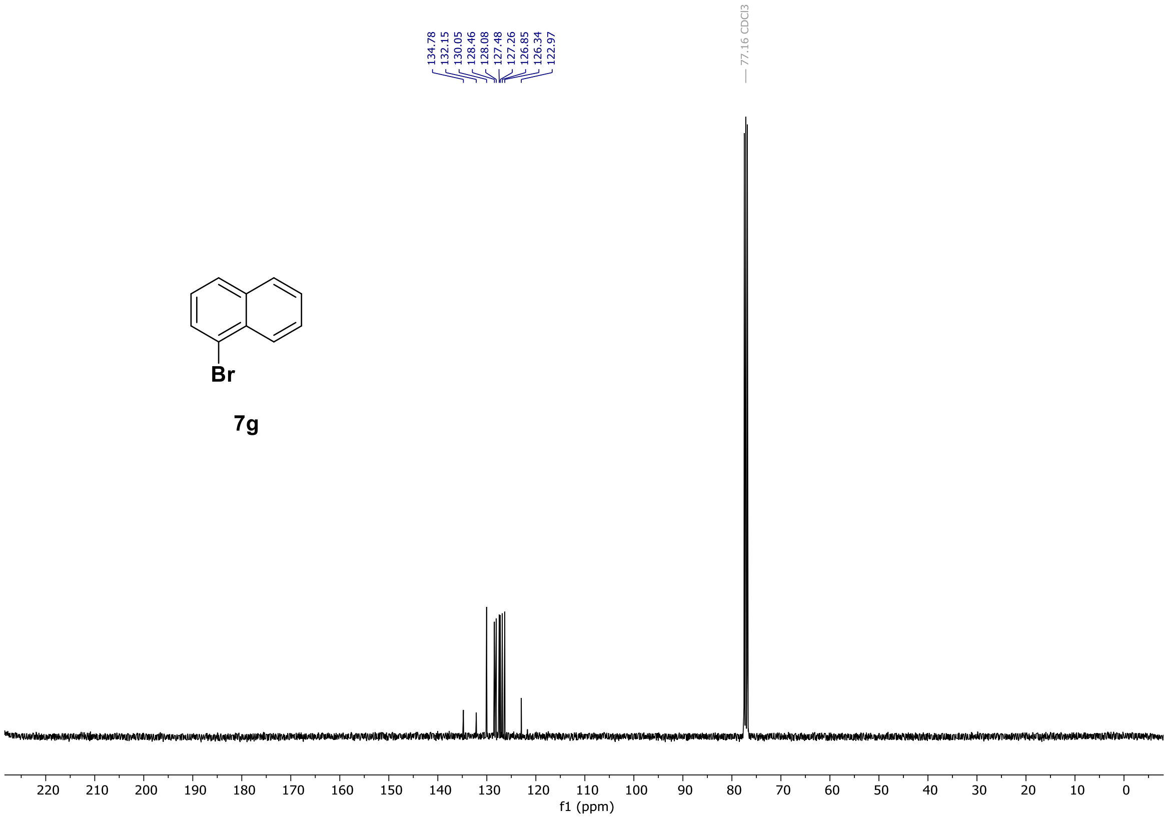

Compound **7h**

^{1}H NMR (400 MHz, $CDCl_3$)

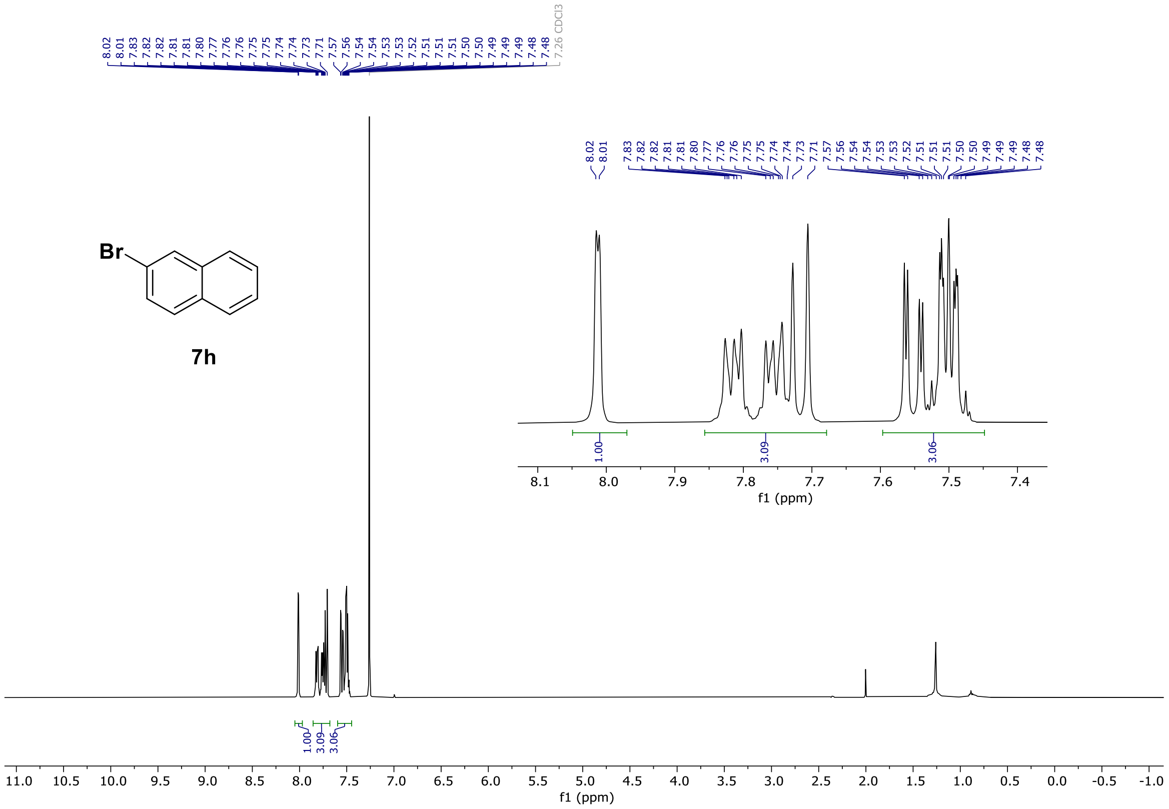

^{13}C{^{1}H} NMR (101 MHz, $CDCl_3$)

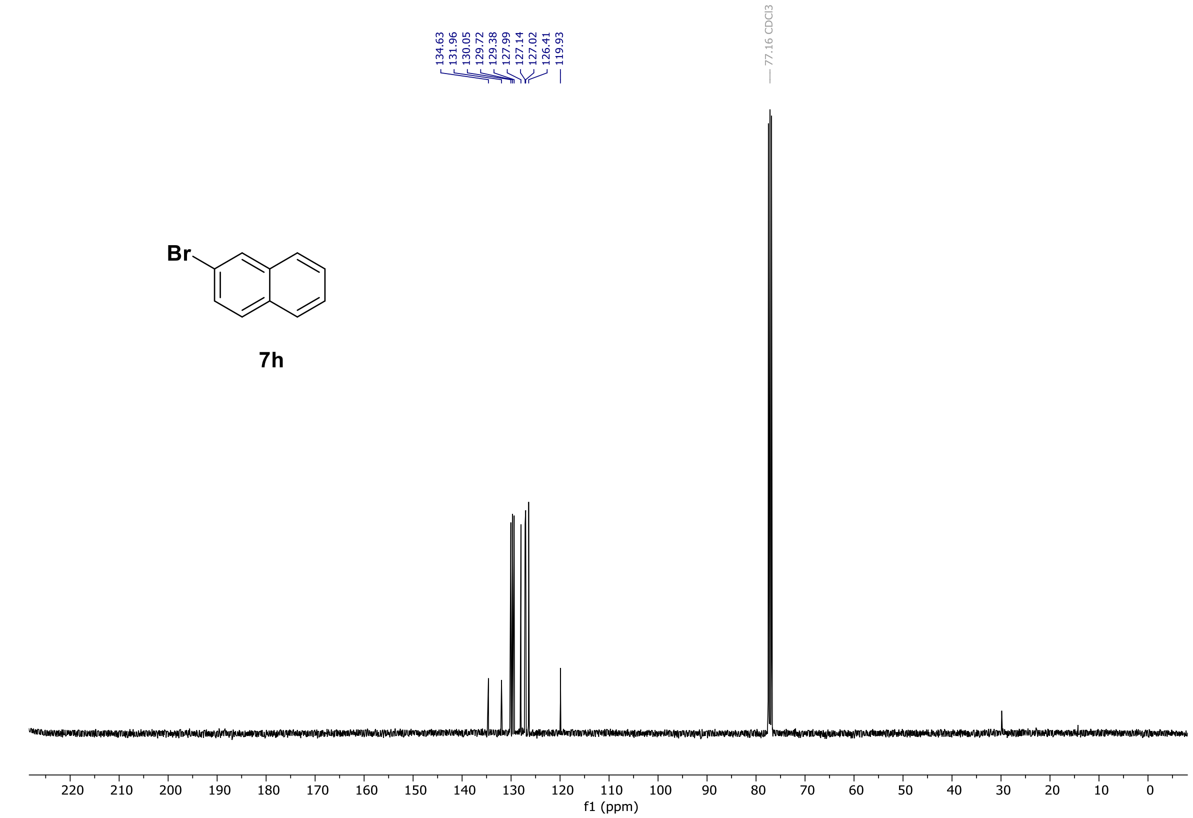

Compound **7h***

^{1}H NMR (500 MHz, $CDCl_3$)

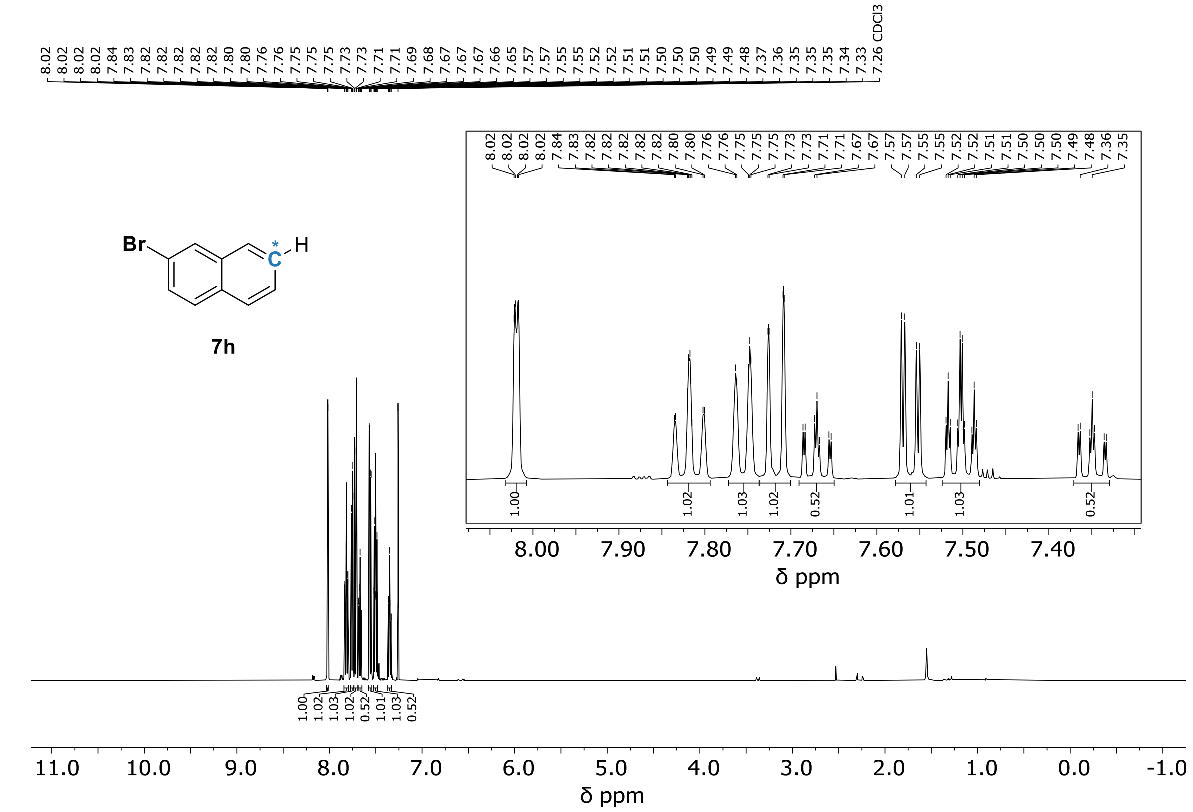

$^{13}C\{^1H\}$ NMR (126 MHz, $CDCl_3$)

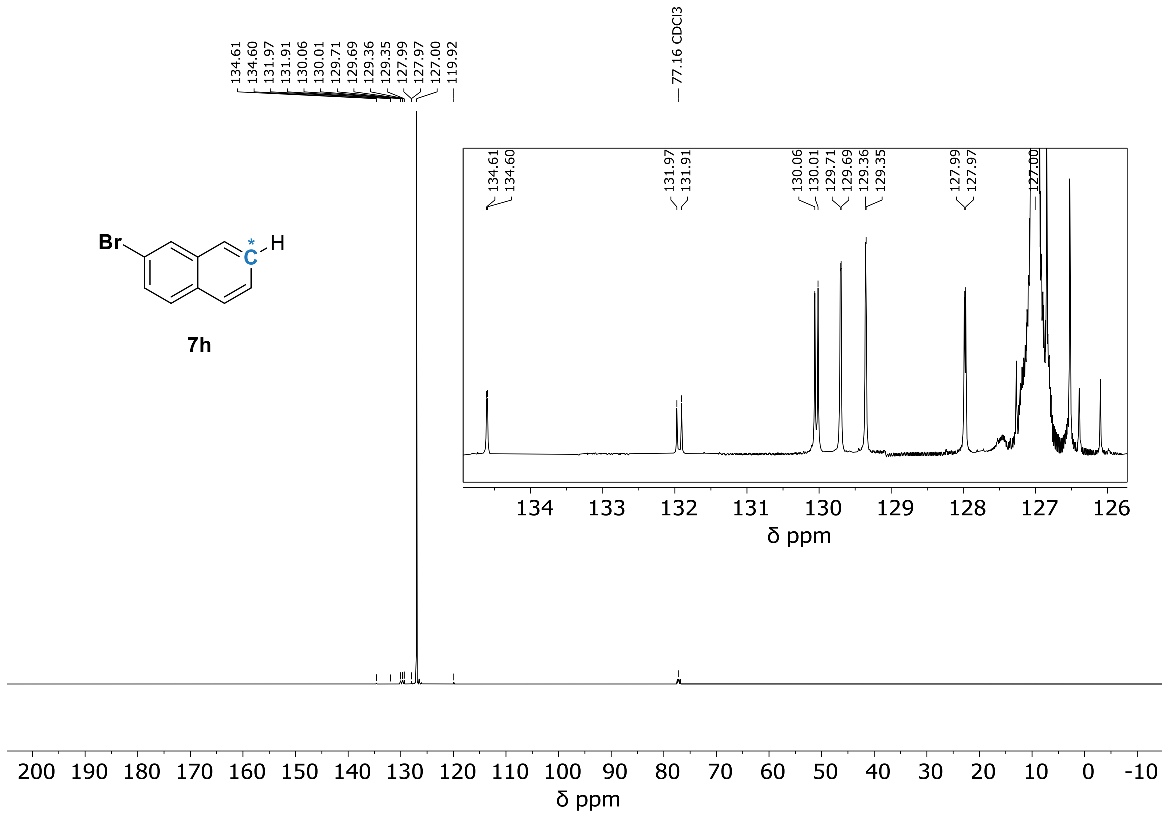

^{13}C NMR (126 MHz, $CDCl_3$)

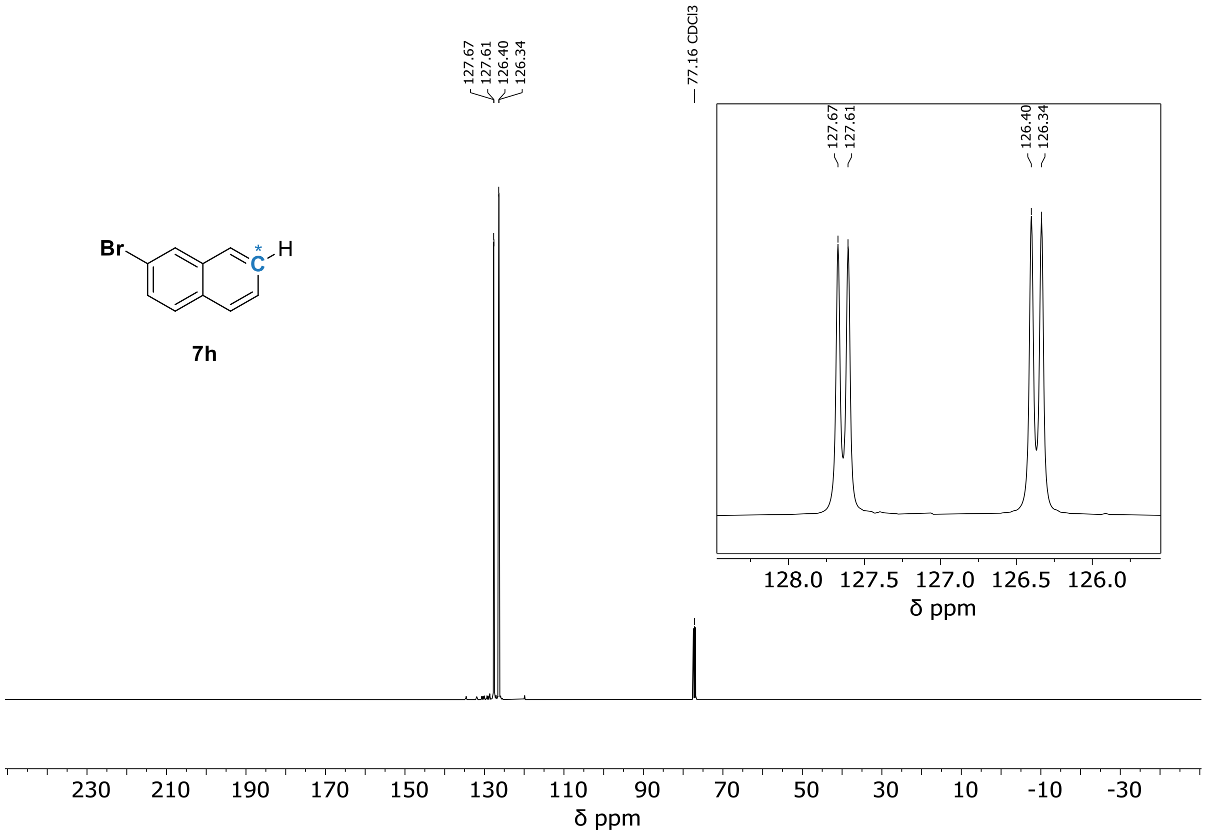

Compound **7i**

^{1}H NMR (400 MHz, $CDCl_3$)

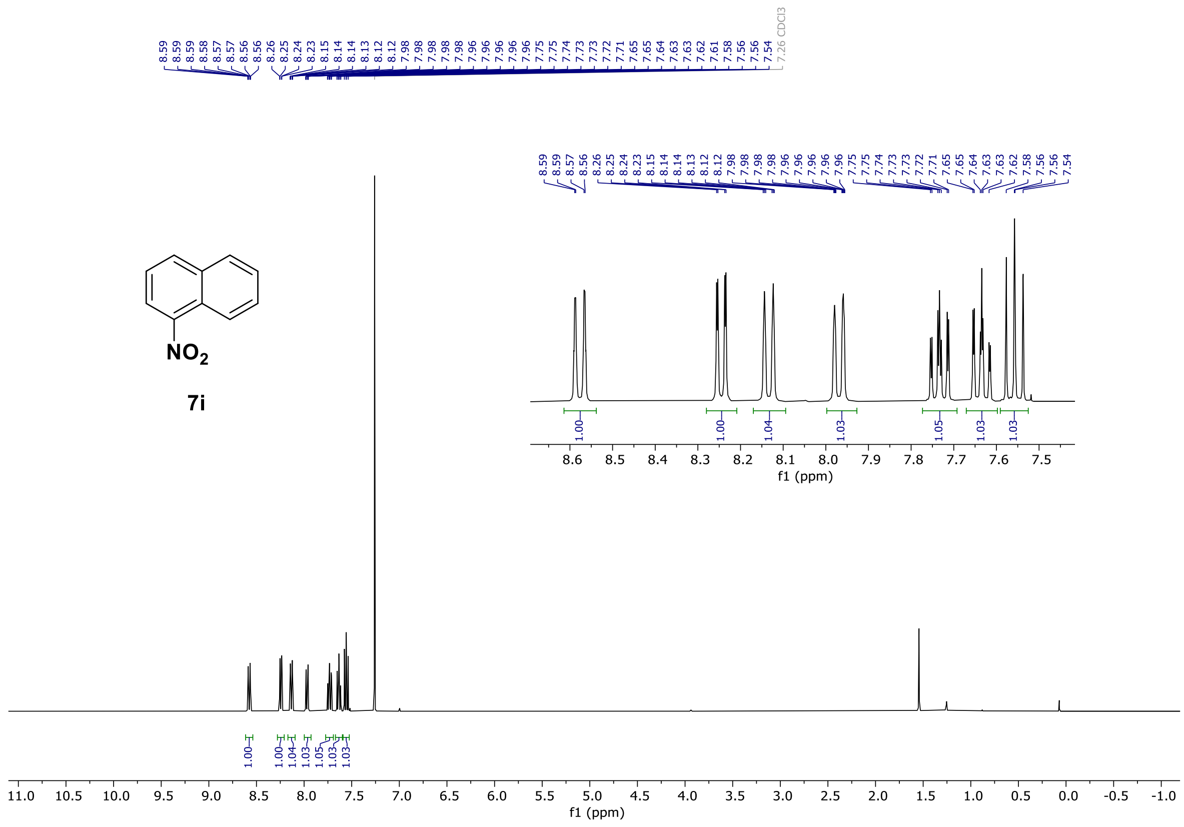

^{13}C{^{1}H} NMR (101 MHz, $CDCl_3$)

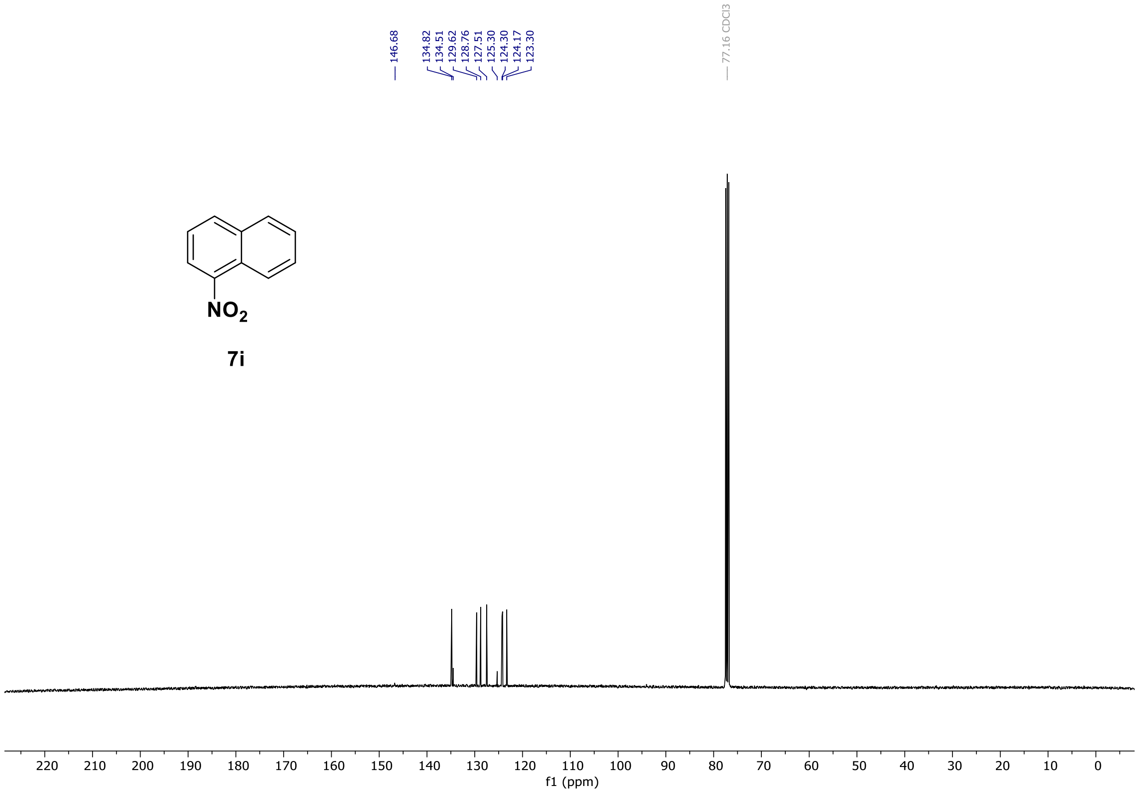

Compound **7j**

^{1}H NMR (400 MHz, $CDCl_3$)

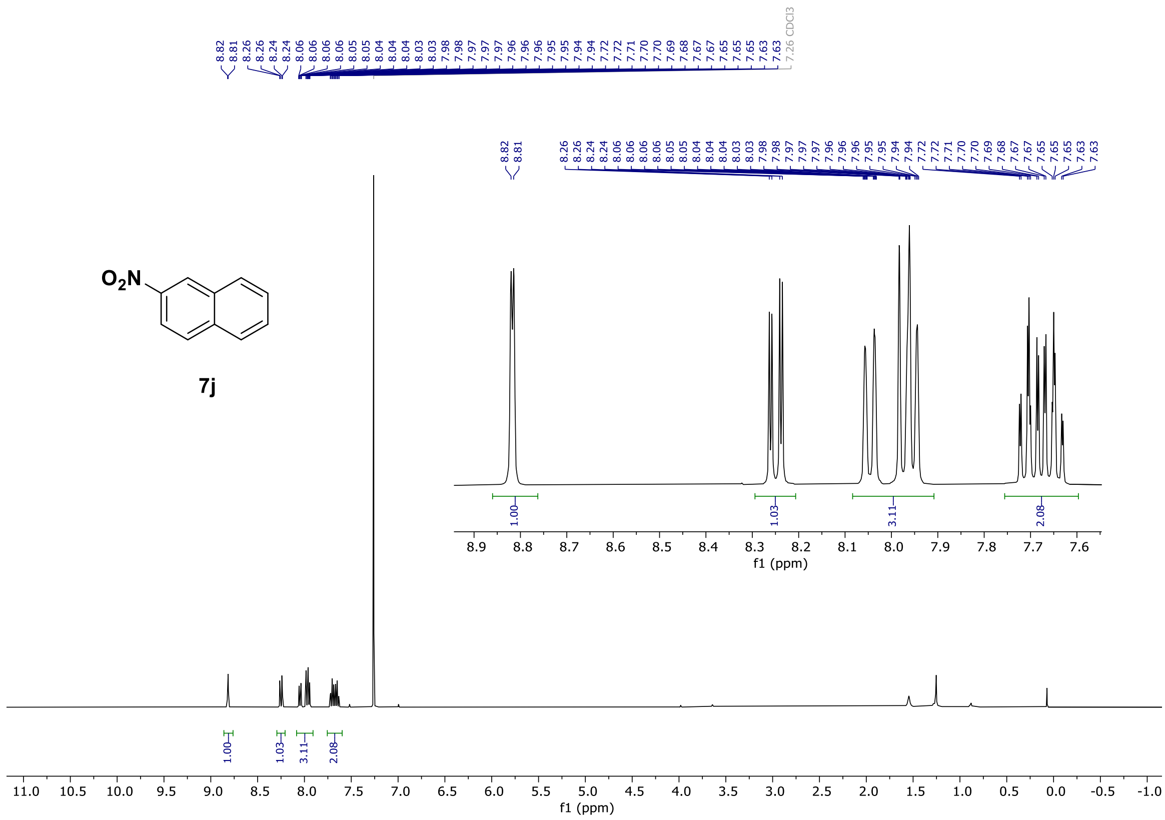

7j

$^{13}C\{^{1}H\}$ NMR (101 MHz, $CDCl_3$)

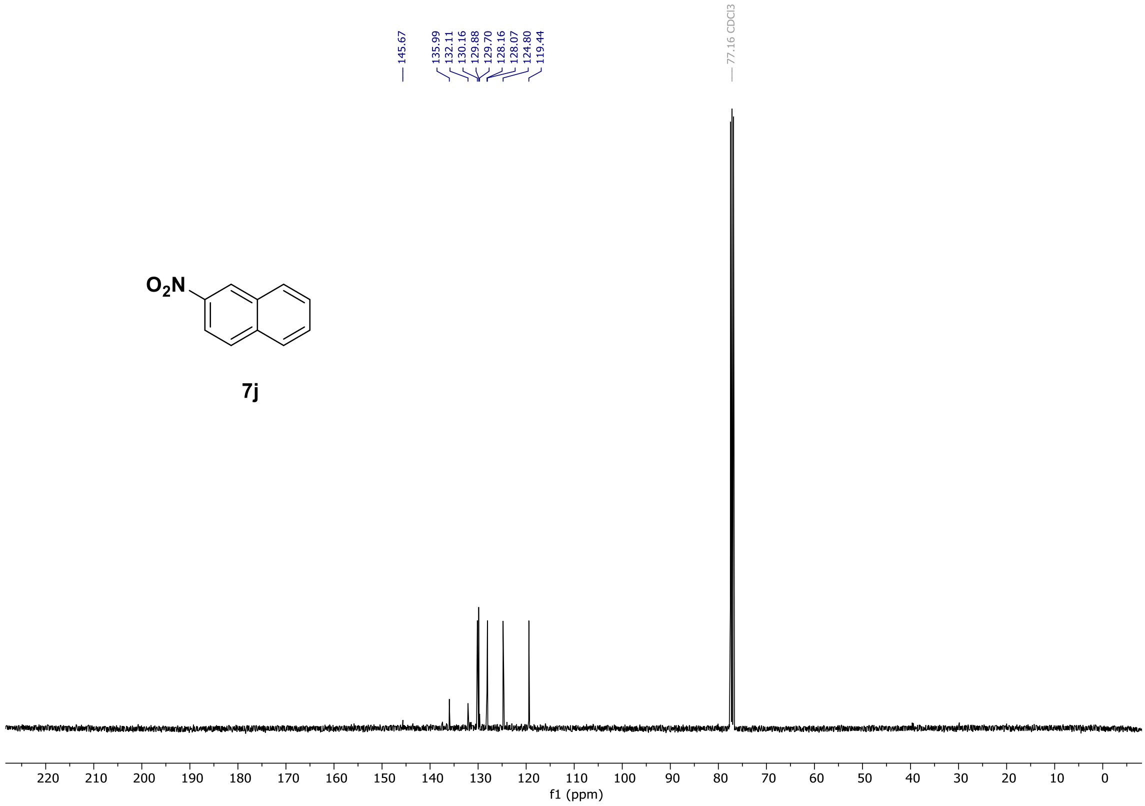

7j

Compound **7k**

^{1}H NMR (400 MHz, $CDCl_3$)

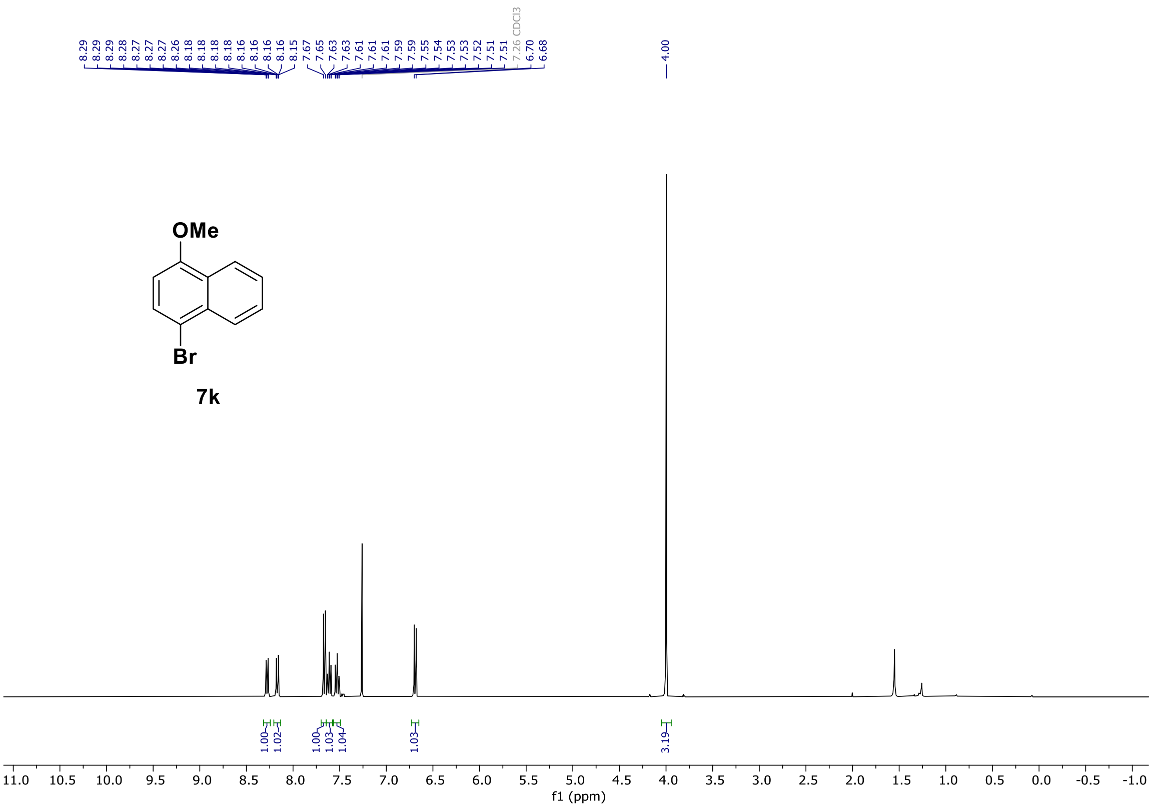

$^{13}C\{^{1}H\}$ NMR (101 MHz, $CDCl_3$)

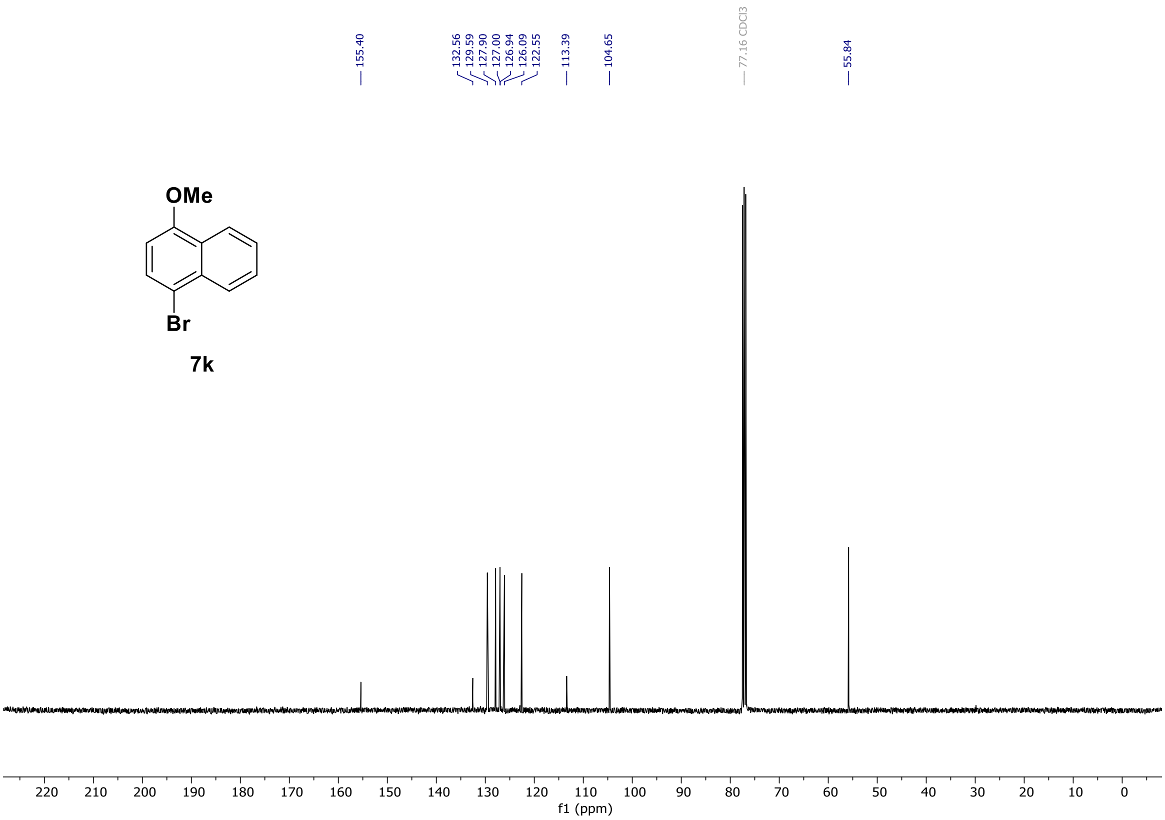

Compound **7l**

^1H NMR (400 MHz, $CDCl_3$)

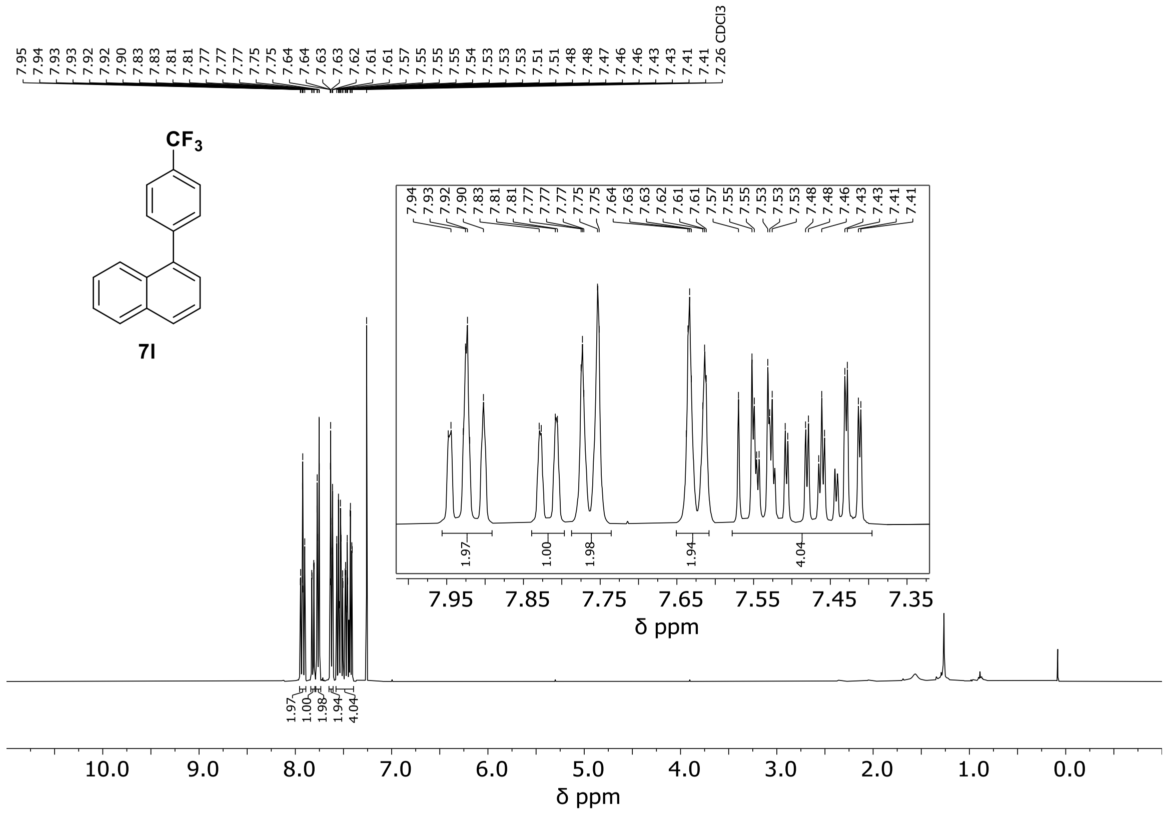

$^{13}C\{^1H\}$ NMR (101 MHz, $CDCl_3$)

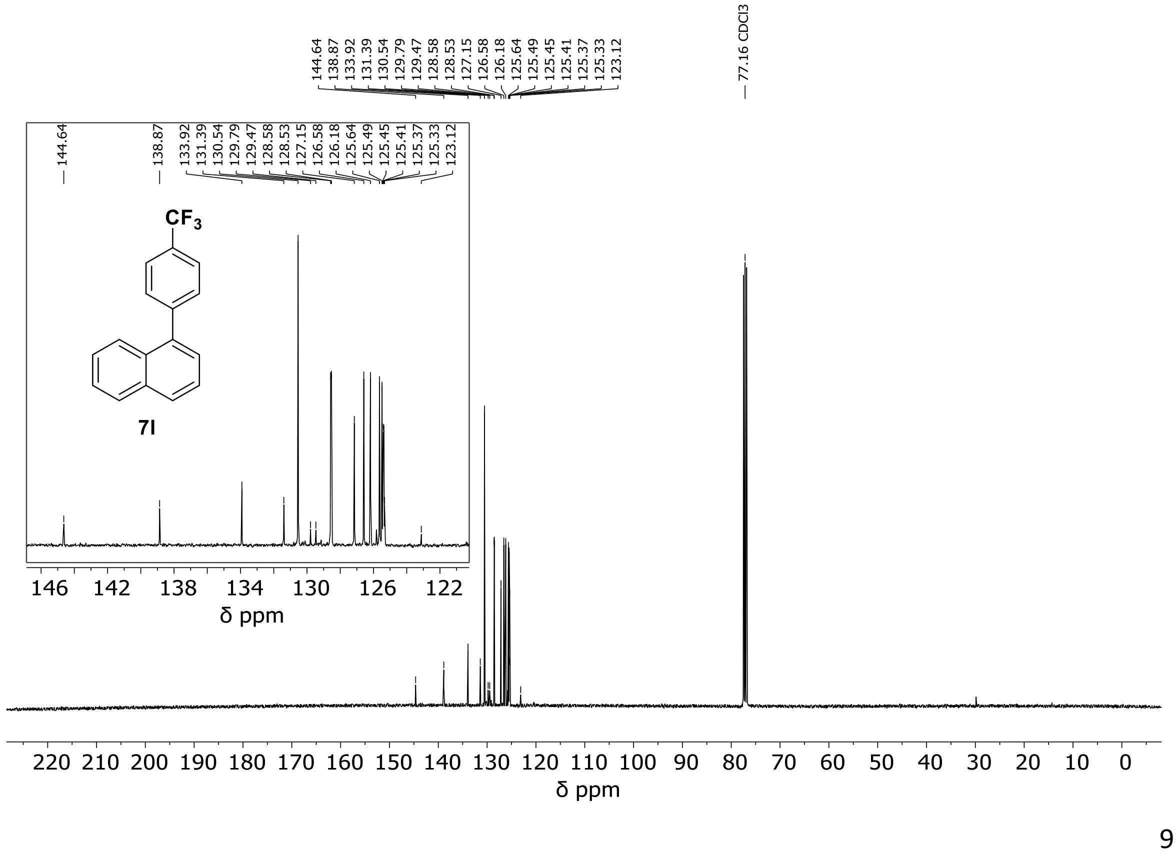

^{19}F NMR (377 MHz, $CDCl_3$)

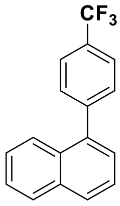

7l

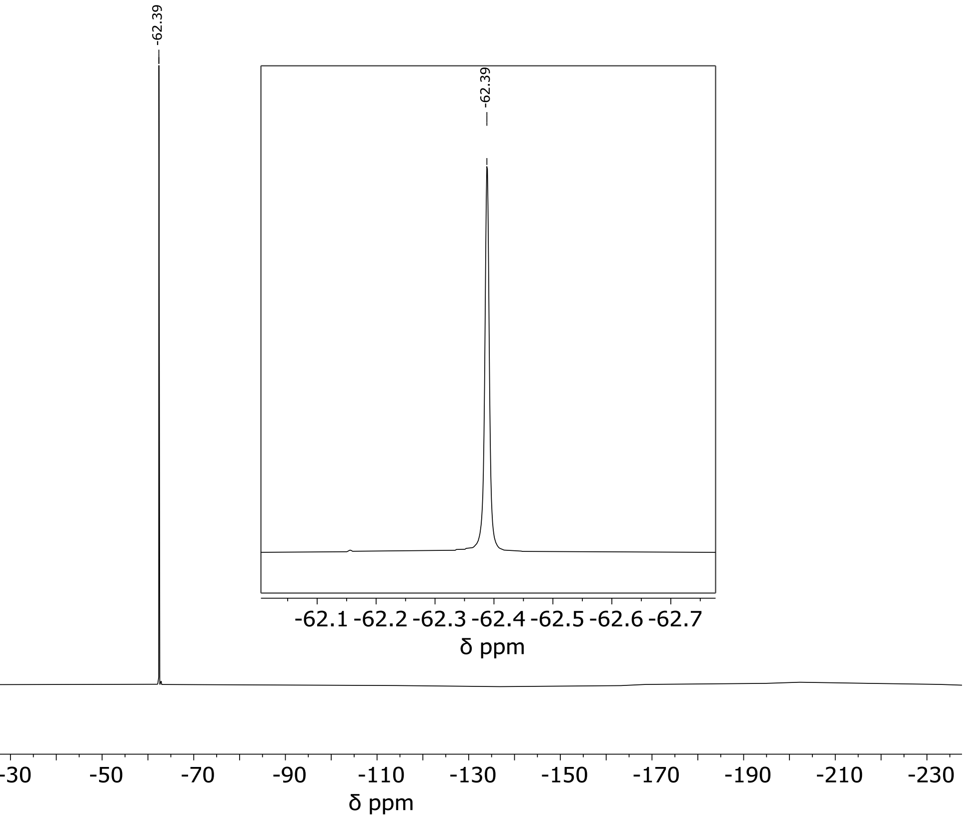

Compound **7m**

^{1}H NMR (400 MHz, $CDCl_3$)

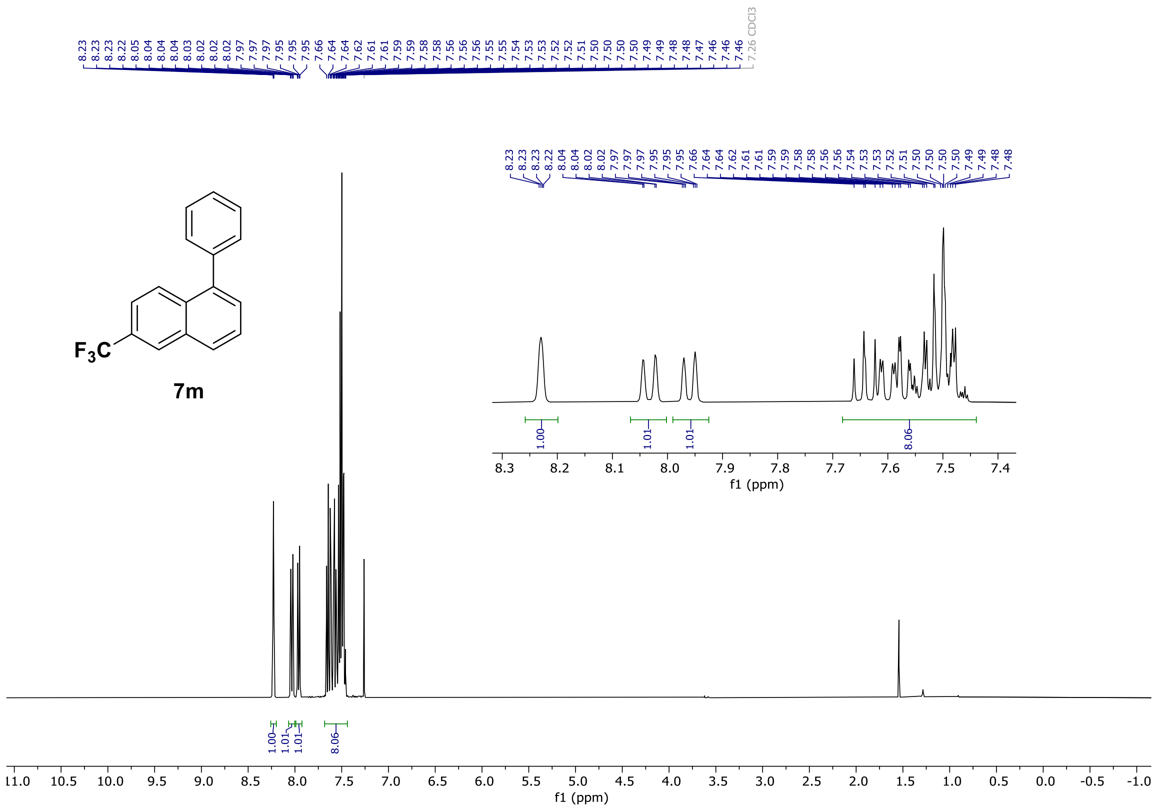

^{13}C{^{1}H} NMR (101 MHz, $CDCl_3$)

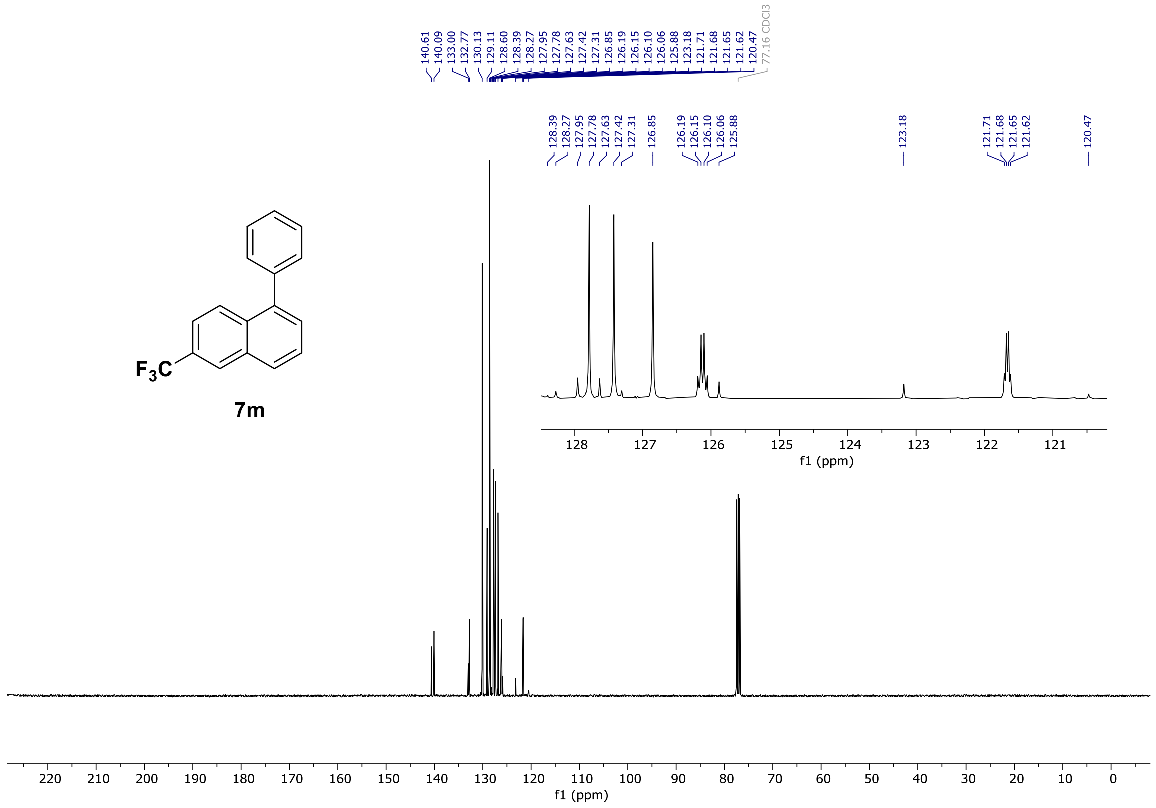

^{19}F NMR (377 MHz, $CDCl_3$)

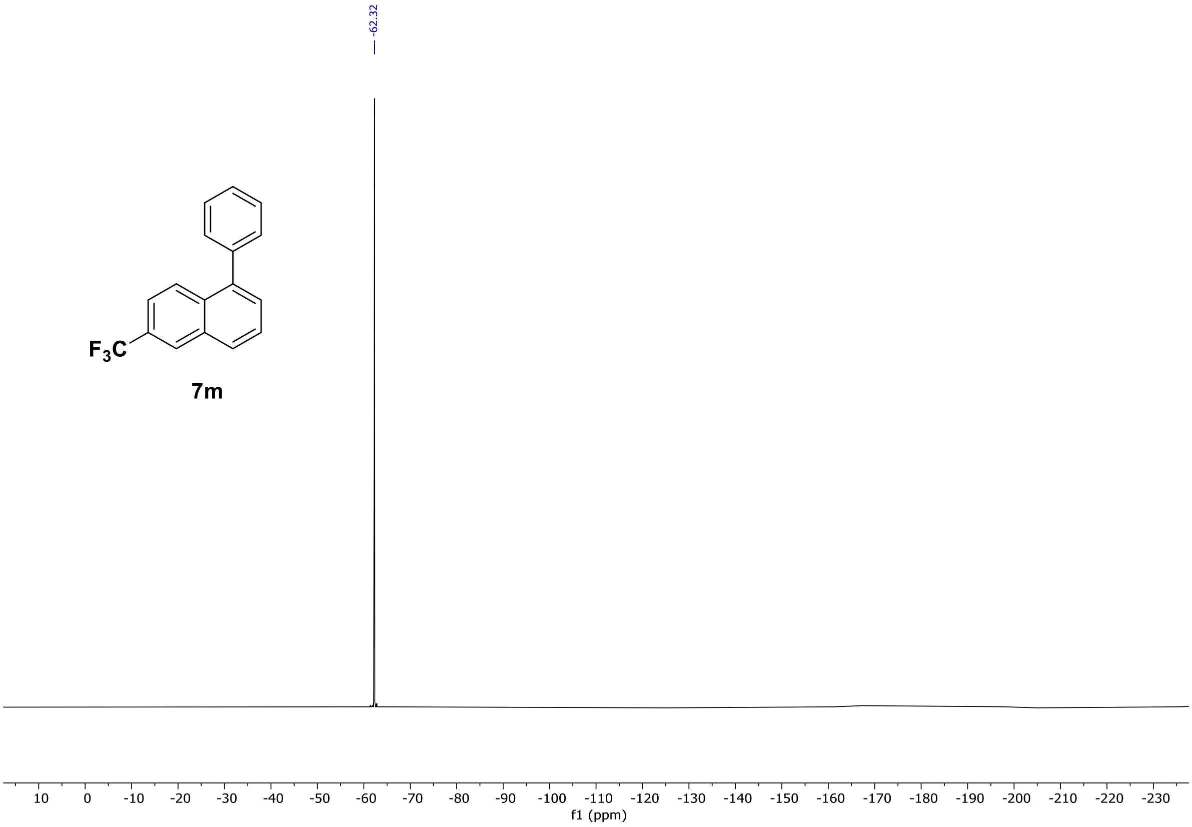

Compound **7n**

^{1}H NMR (400 MHz, $CDCl_3$)

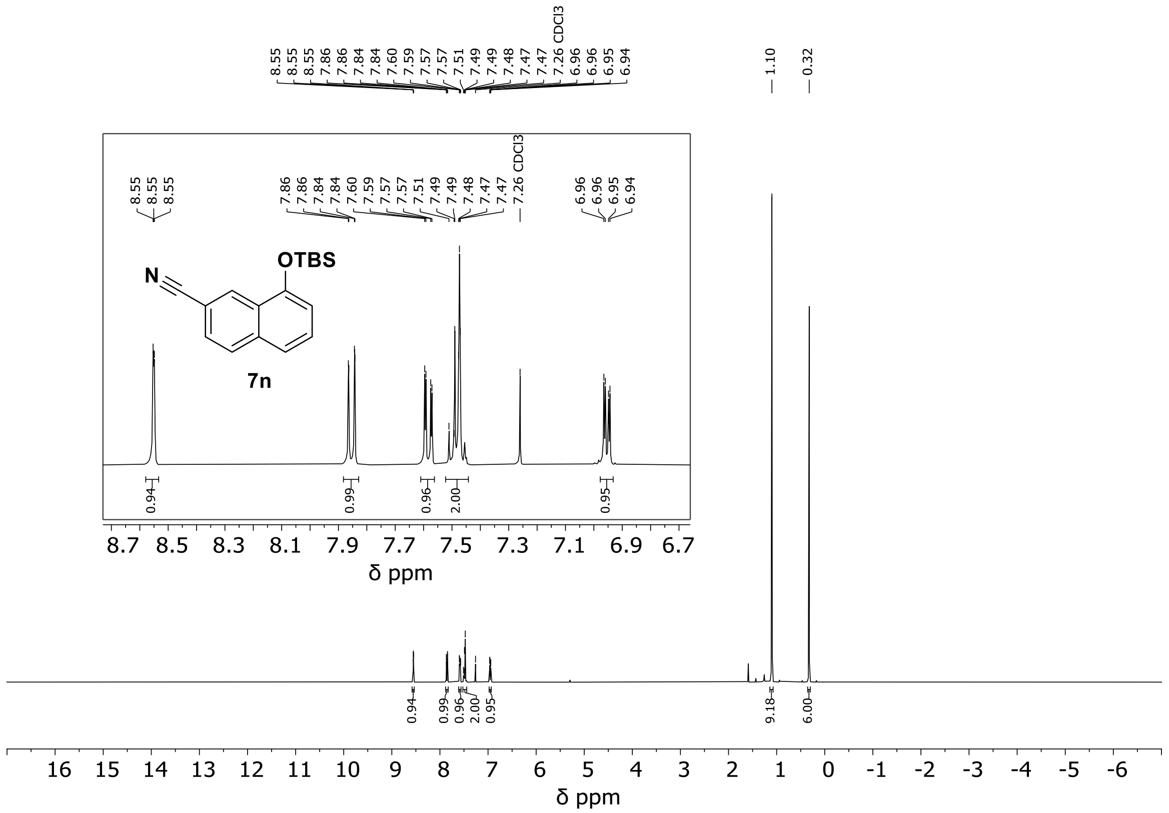

^{13}C{^{1}H} NMR (101 MHz, $CDCl_3$)

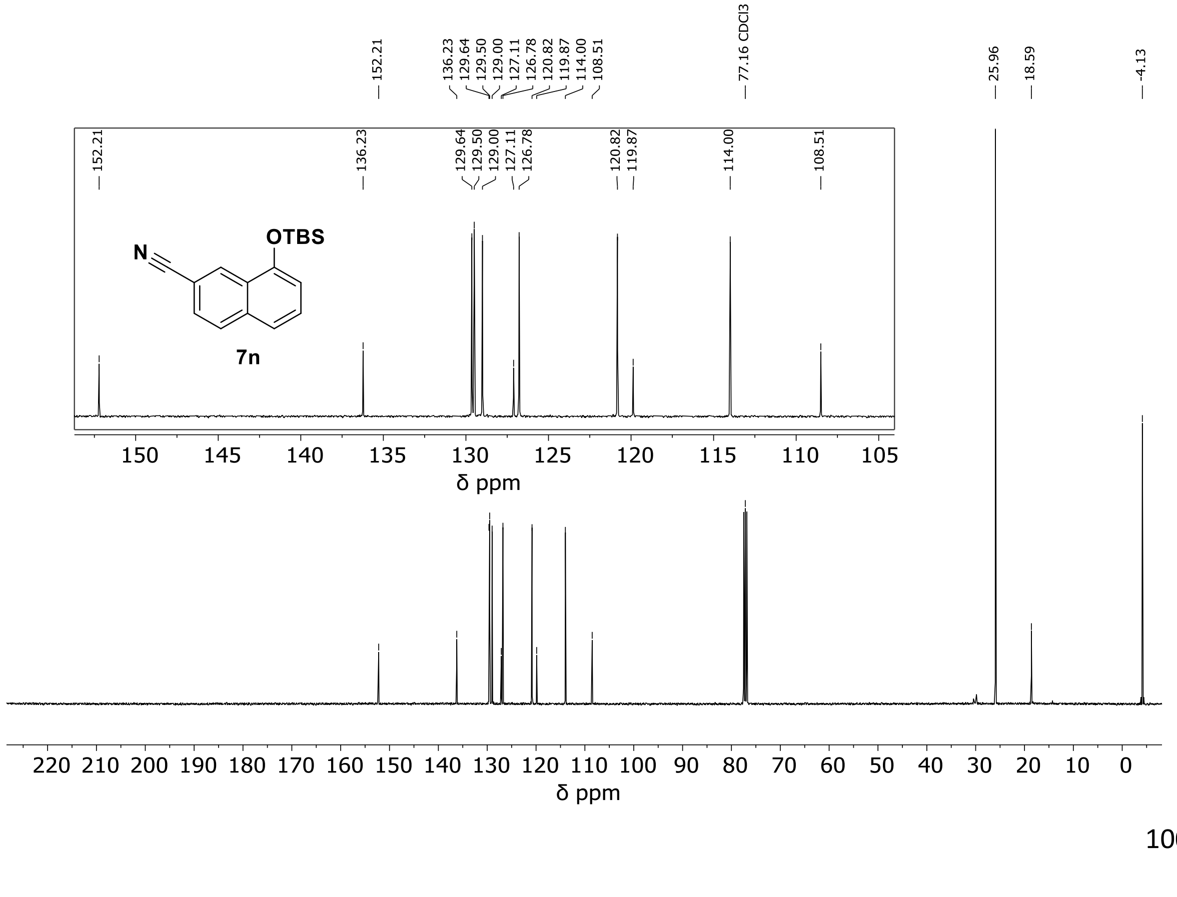

Compound **7o**

^{1}H NMR (400 MHz, $CDCl_3$)

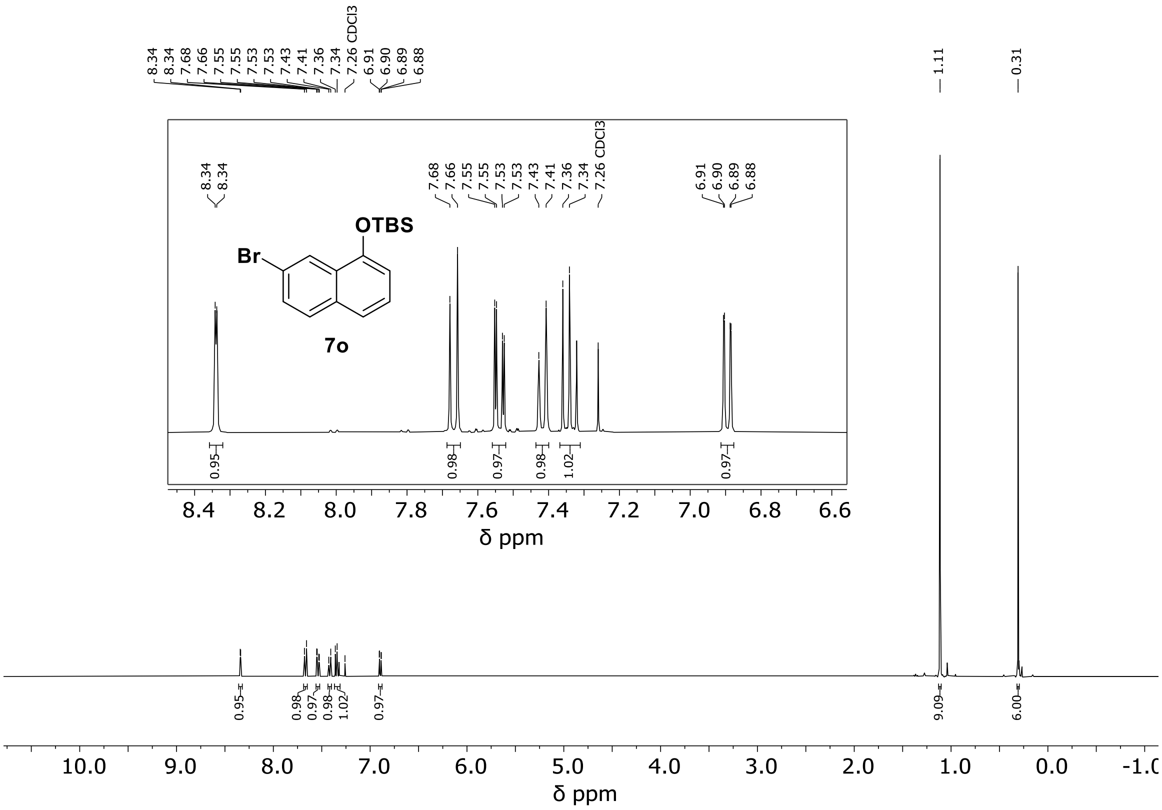

^{13}C{^{1}H} NMR (101 MHz, $CDCl_3$)

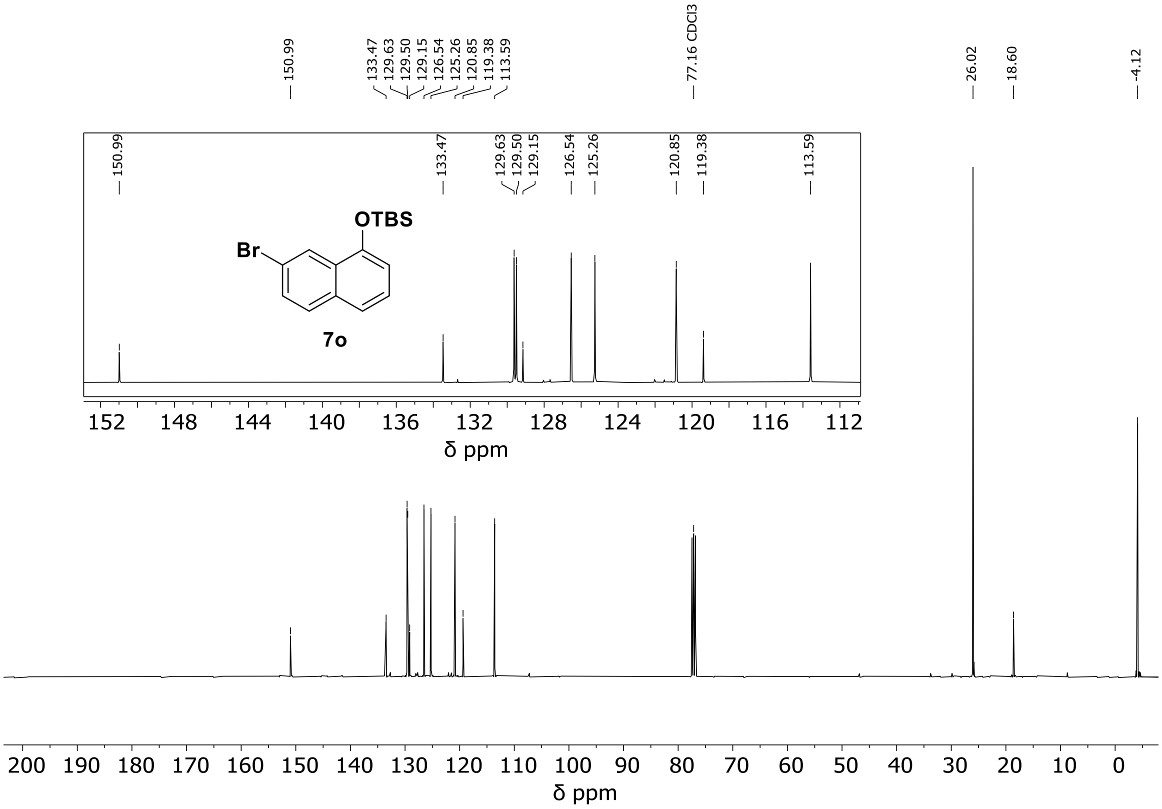

Compound **7p***

^{1}H NMR (400 MHz, $CDCl_3$)

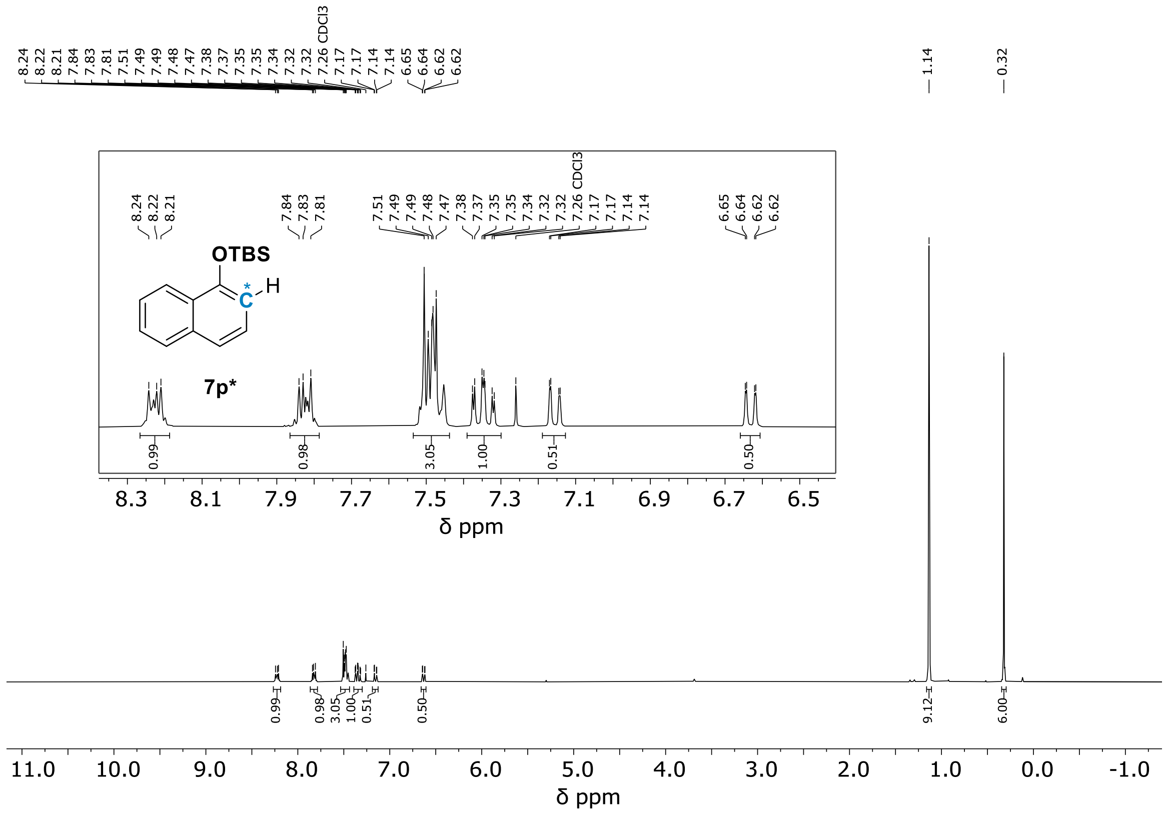

^{13}C{^{1}H} NMR (101 MHz, $CDCl_3$)

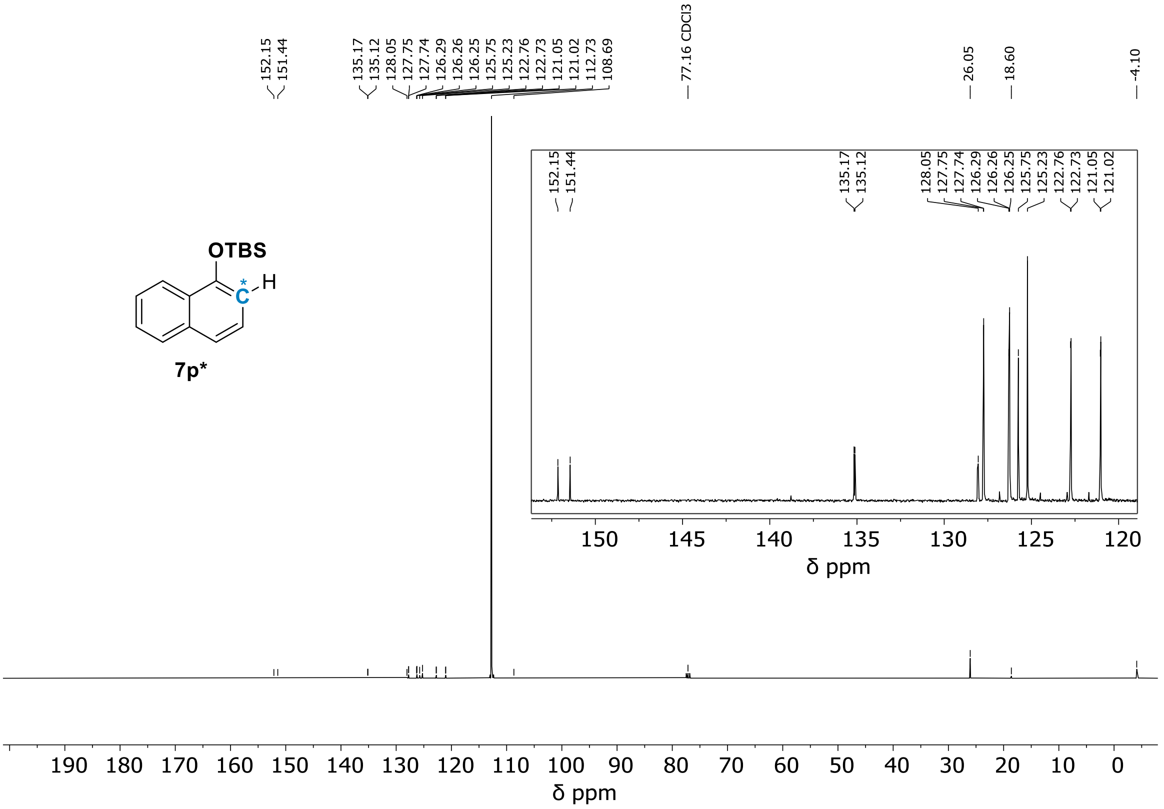

Compound **7q***

^{1}H NMR (600 MHz, $CDCl_3$)

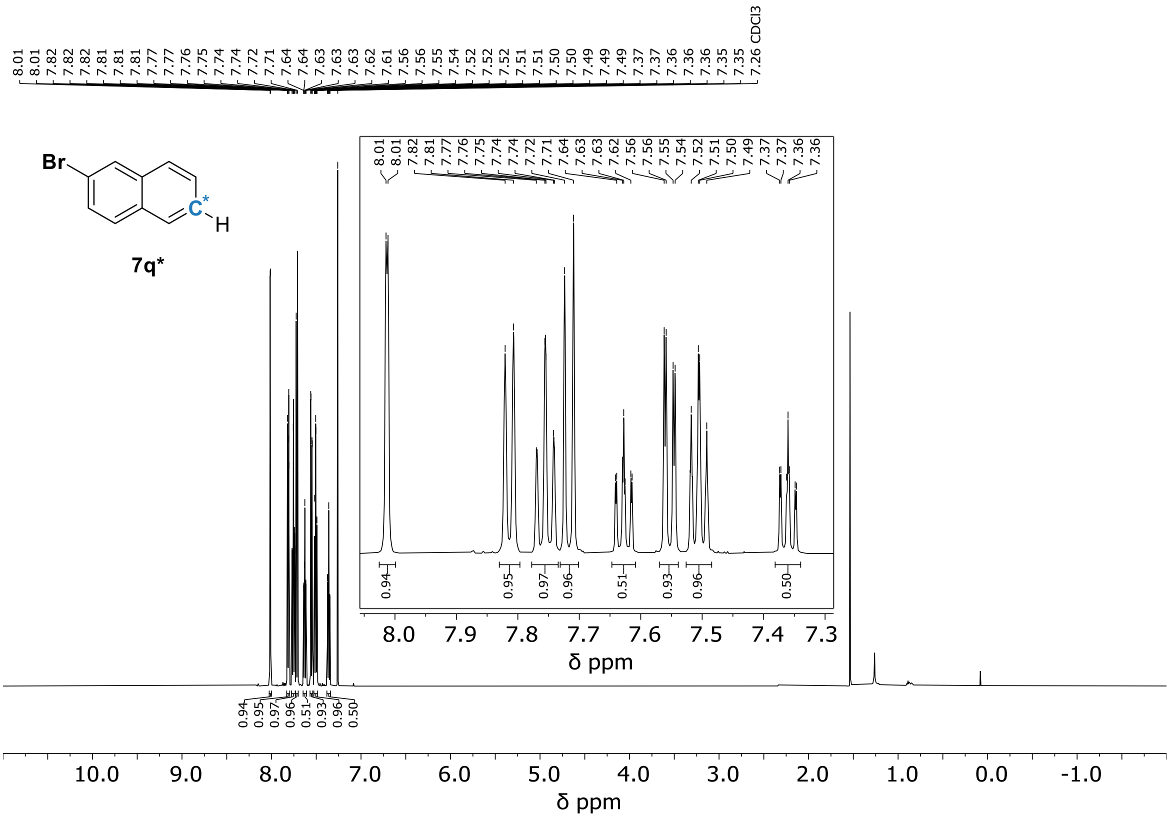

$^{13}C\{^{1}H\}$ NMR (126 MHz, $CDCl_3$)

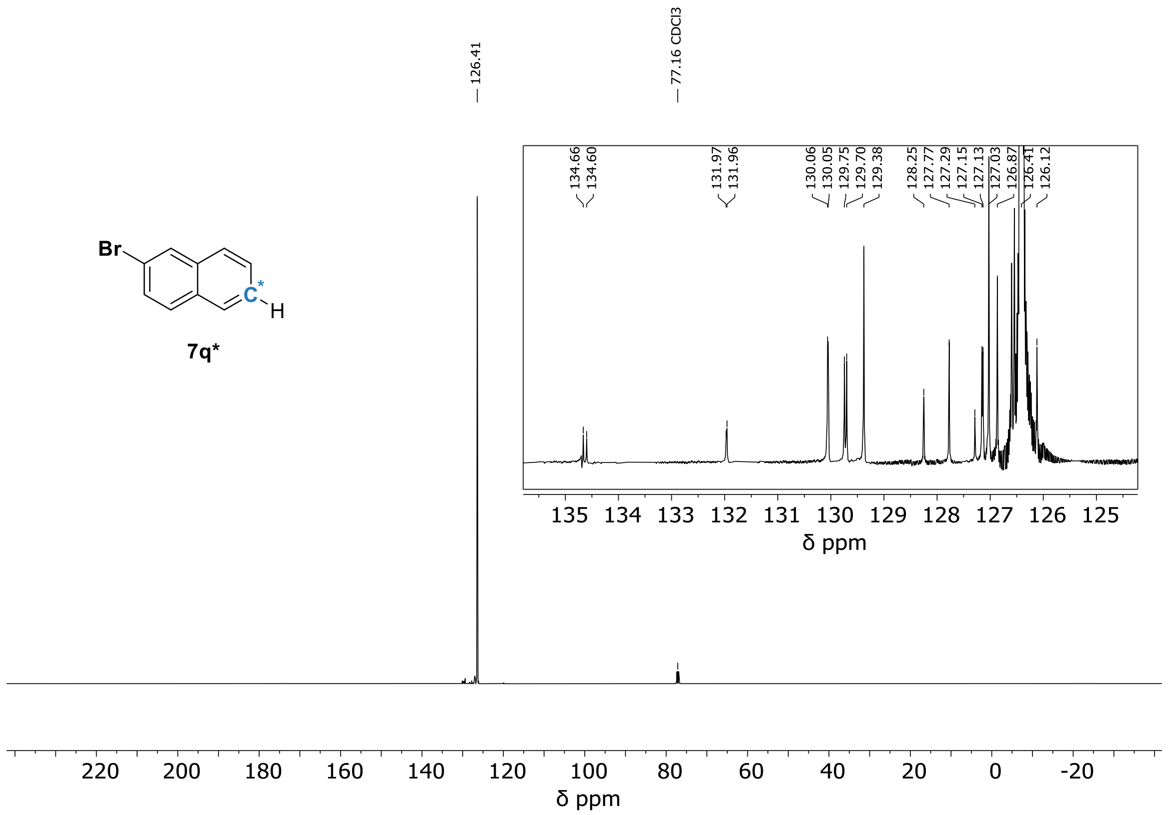

^{13}C NMR (126 MHz, $CDCl_3$)

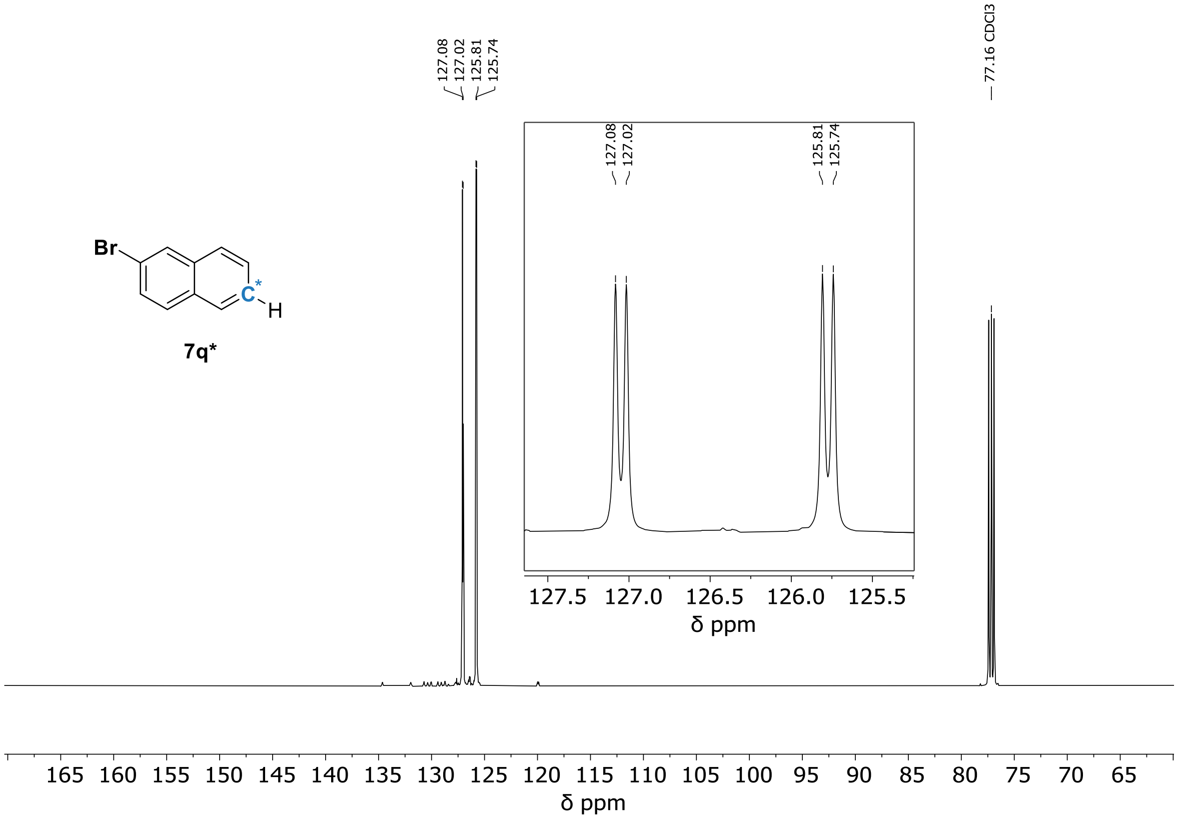

7q*

Compound **7r**

^{1}H NMR (400 MHz, $CDCl_3$)

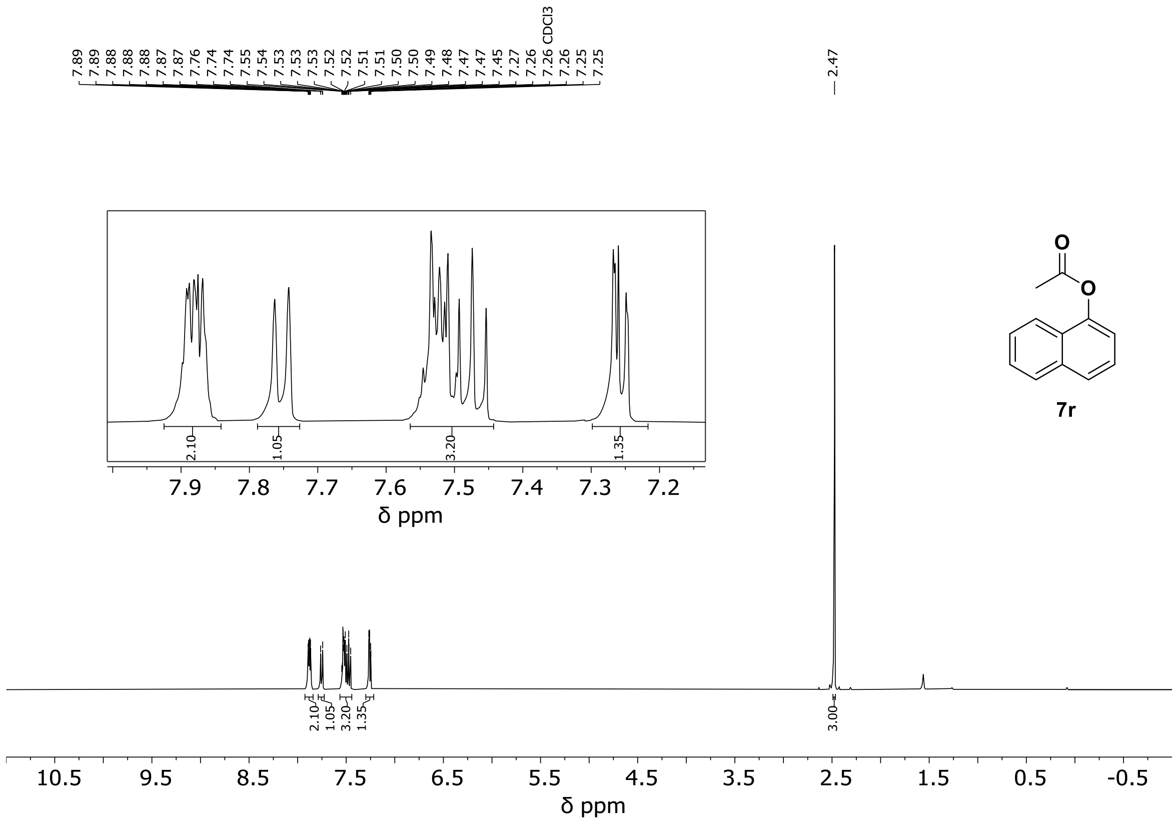

$^{13}C\{^1H\}$ NMR (101 MHz, $CDCl_3$)

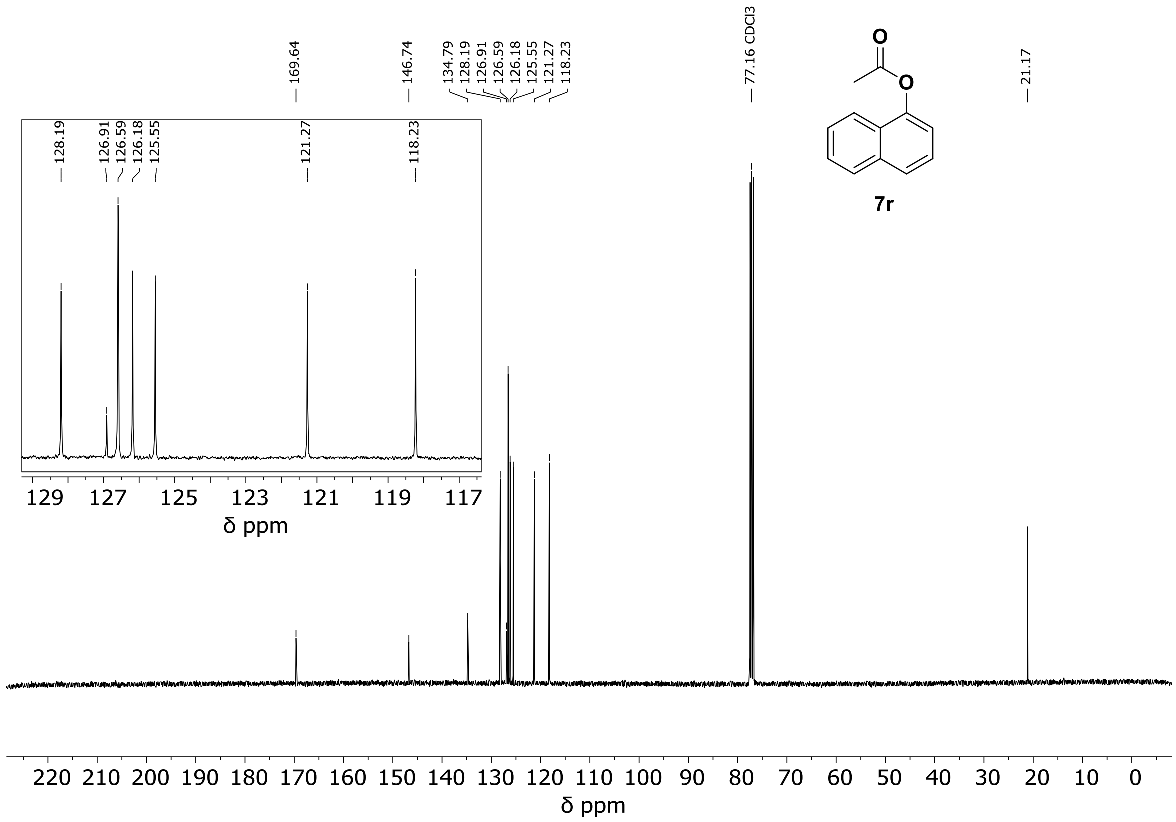

Compound **7s**

^{1}H NMR (400 MHz, $CDCl_3$)

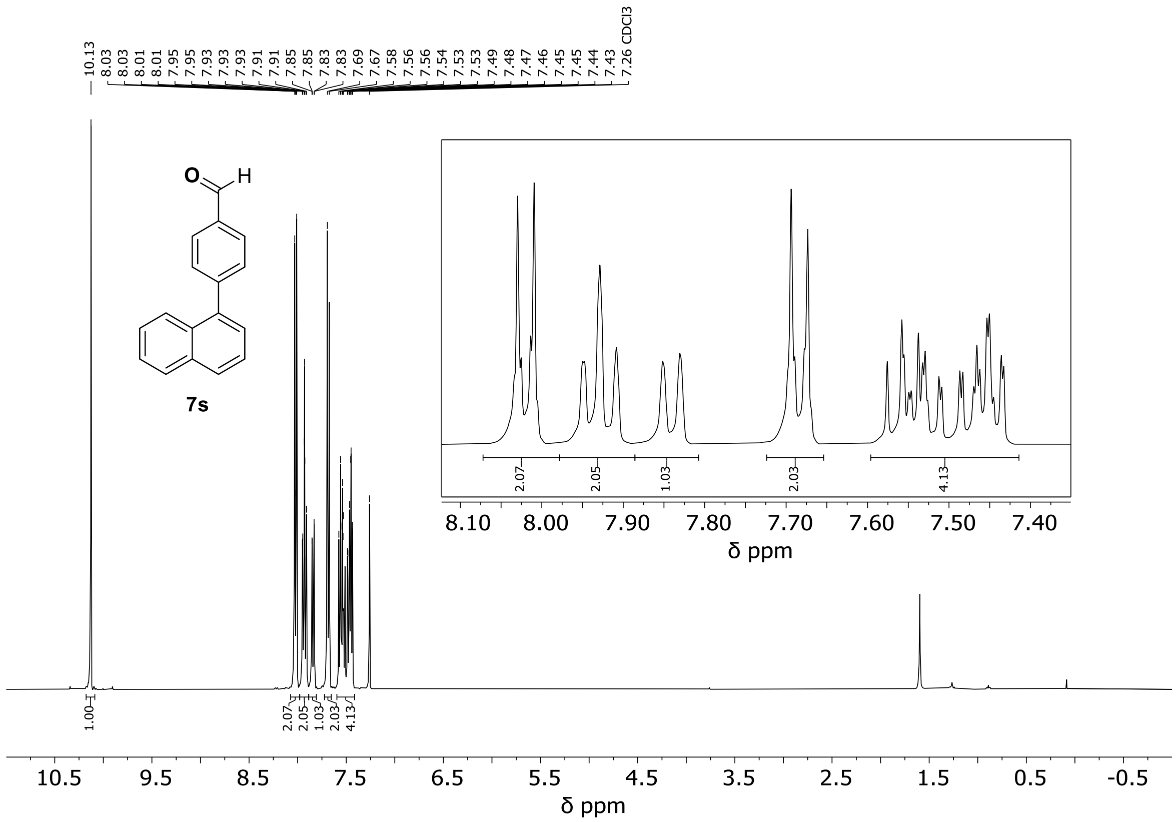

$^{13}C\{^1H\}$ NMR (101 MHz, $CDCl_3$)

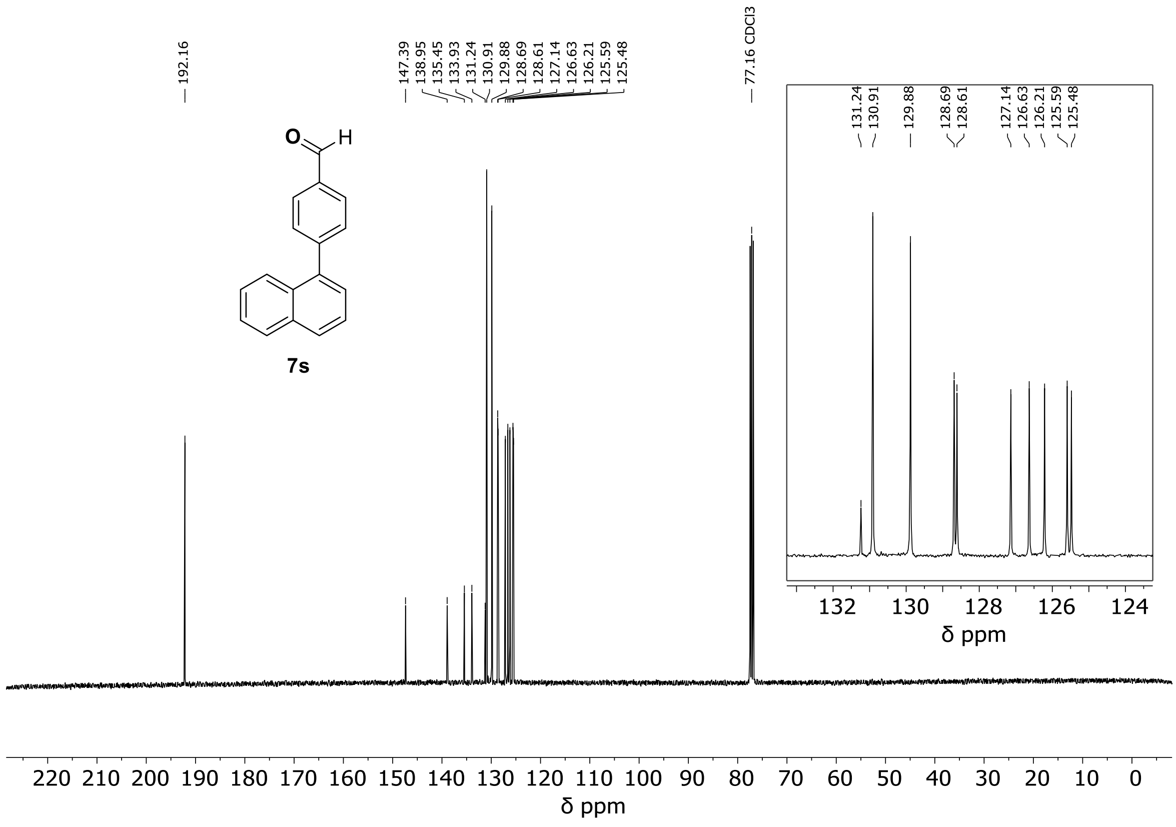

Compound **7t**

^{1}H NMR (400 MHz, $CDCl_3$)

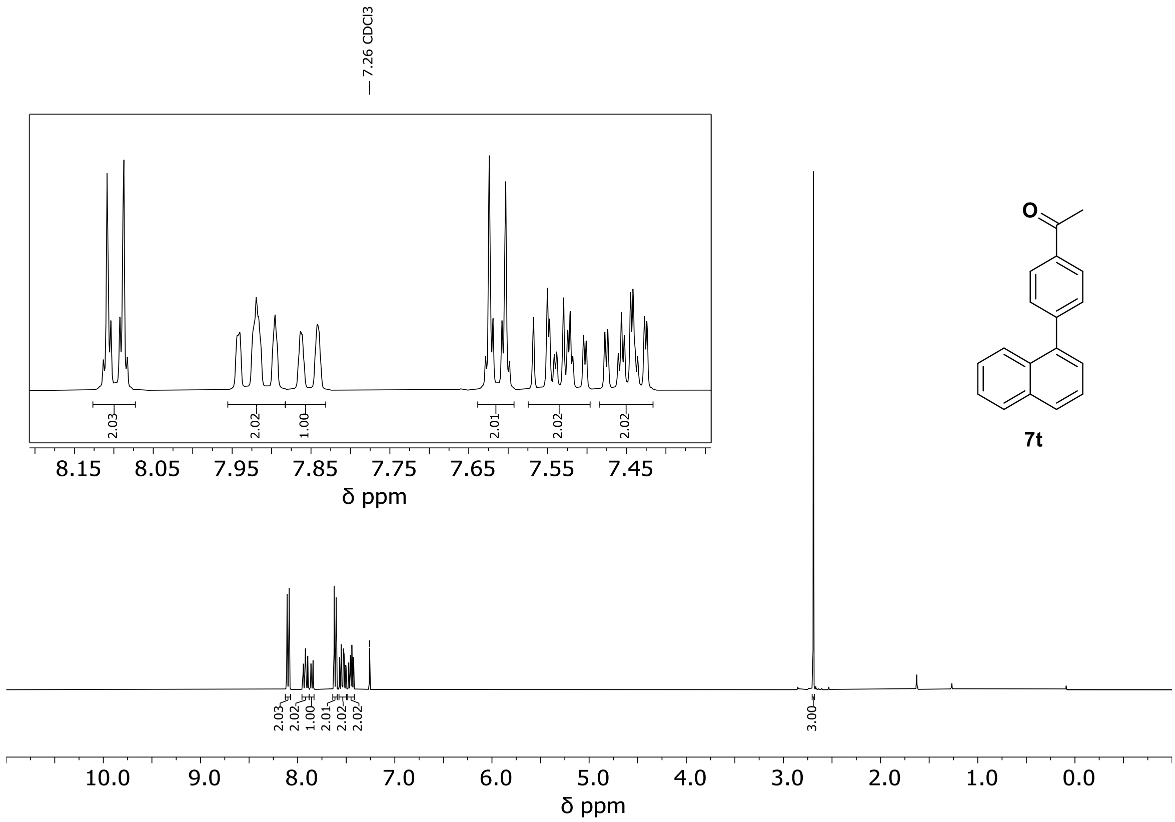

^{13}C{^{1}H} NMR (101 MHz, $CDCl_3$)

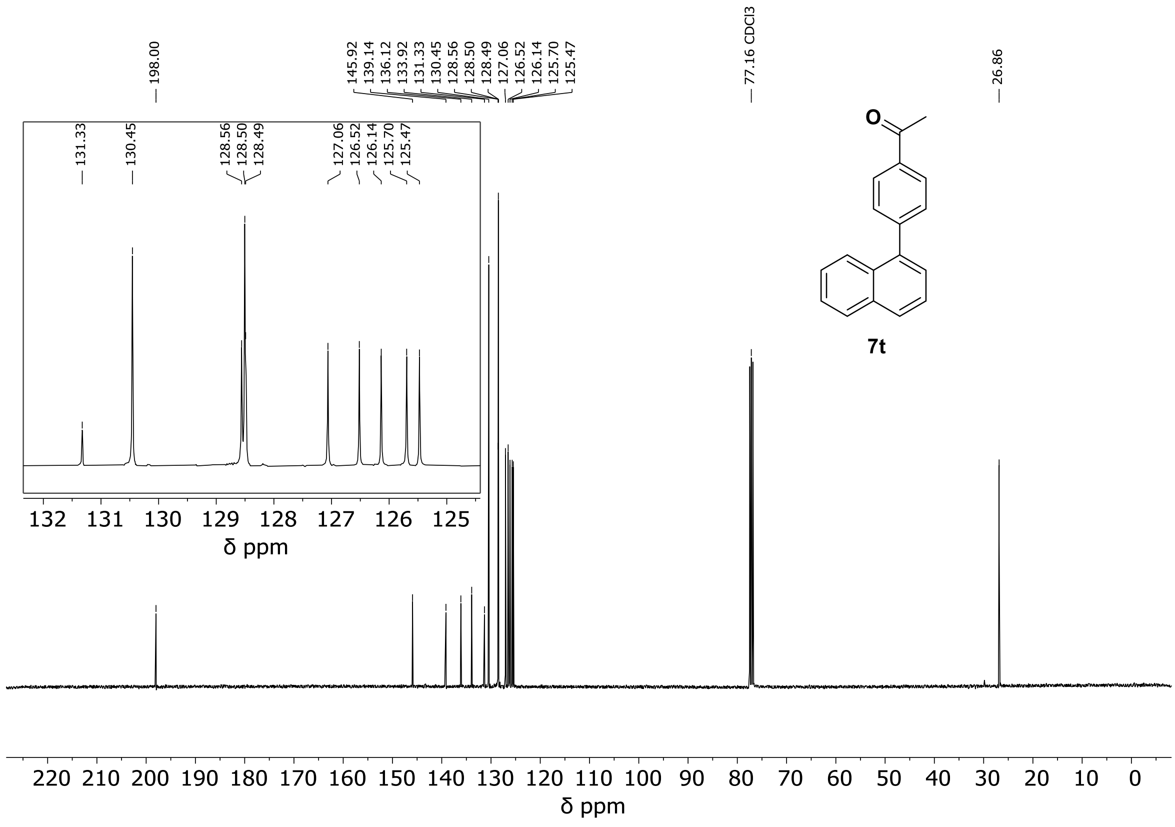

Compound **7u**

^1H NMR (400 MHz, $CDCl_3$)

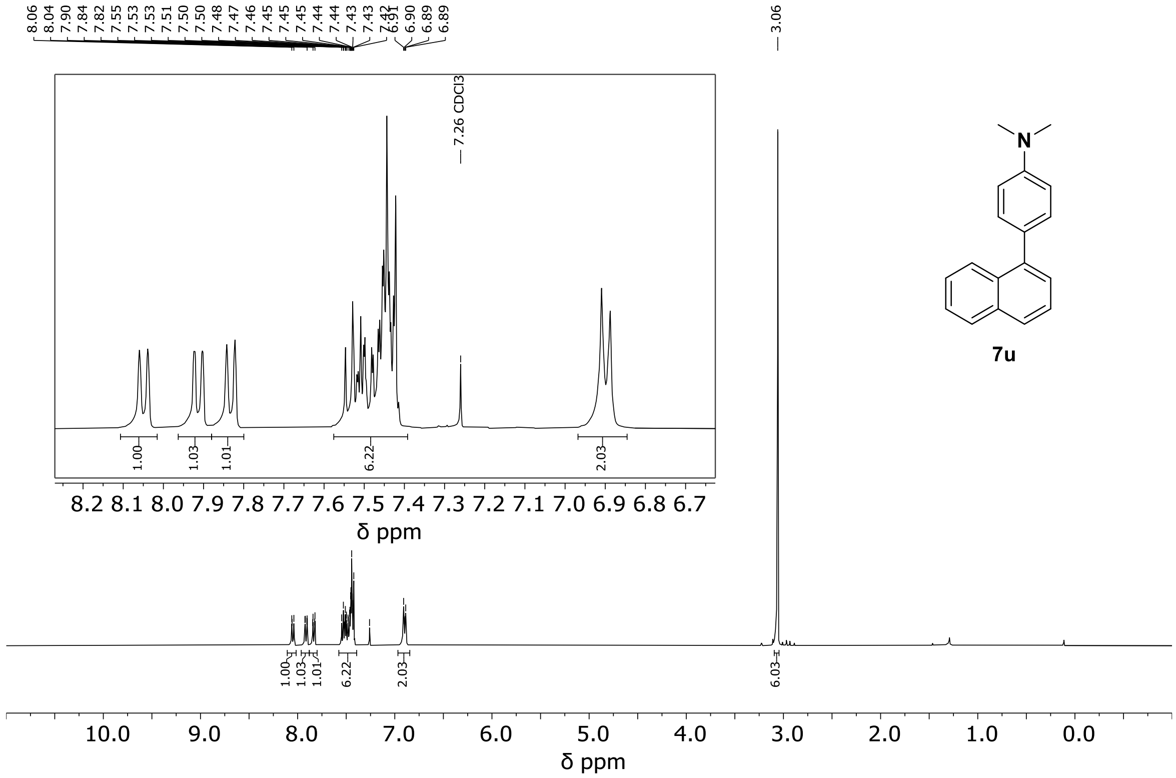

$^{13}C\{^1H\}$ NMR (101 MHz, $CDCl_3$)

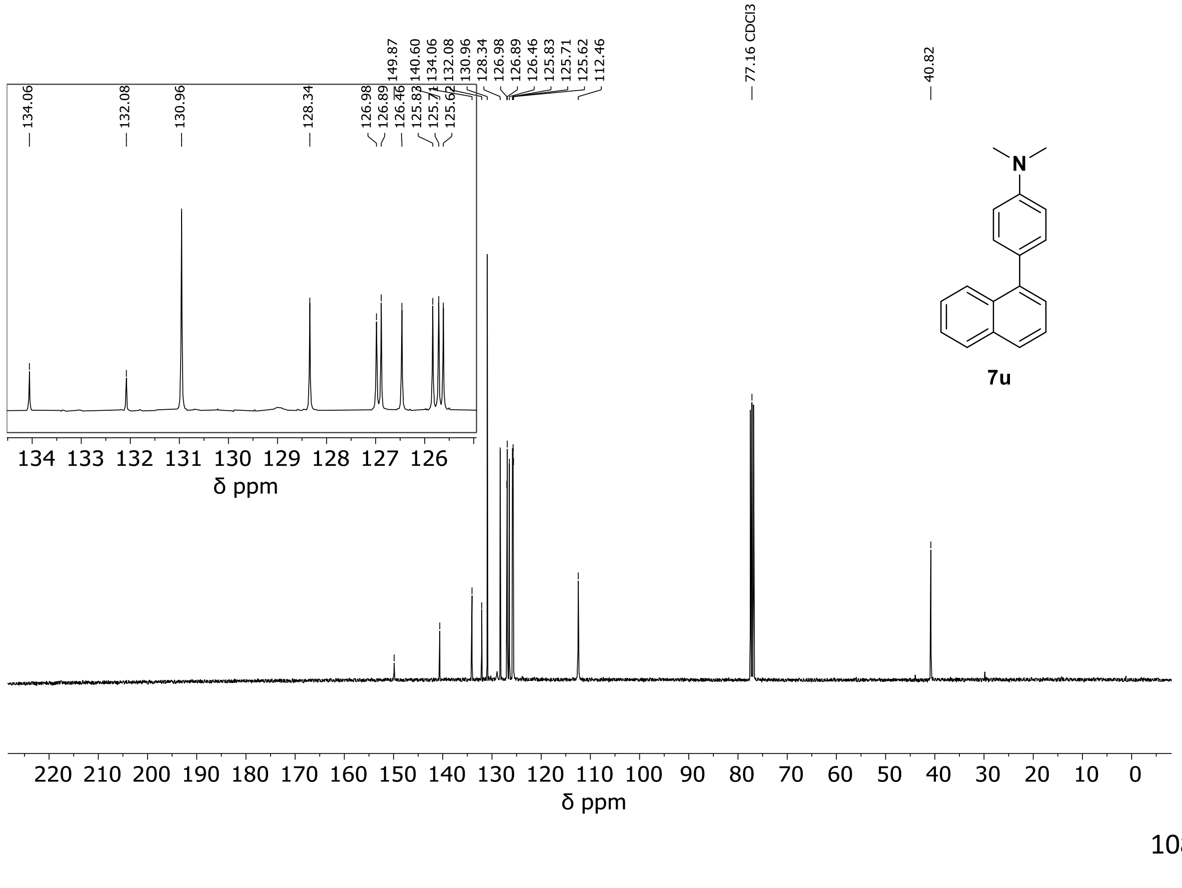

Compound *exo*-8a

^{1}H NMR (400 MHz, CD_3CN)

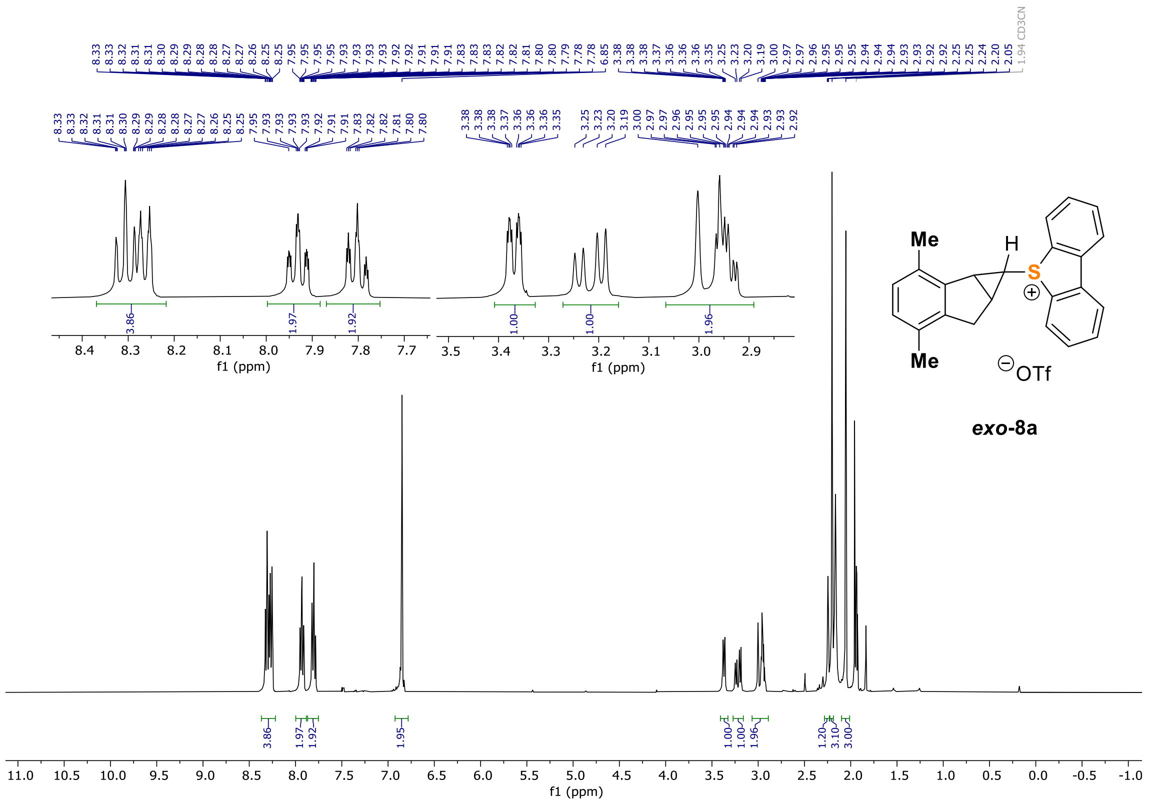

^{13}C{^{1}H} NMR (101 MHz, CD_3CN)

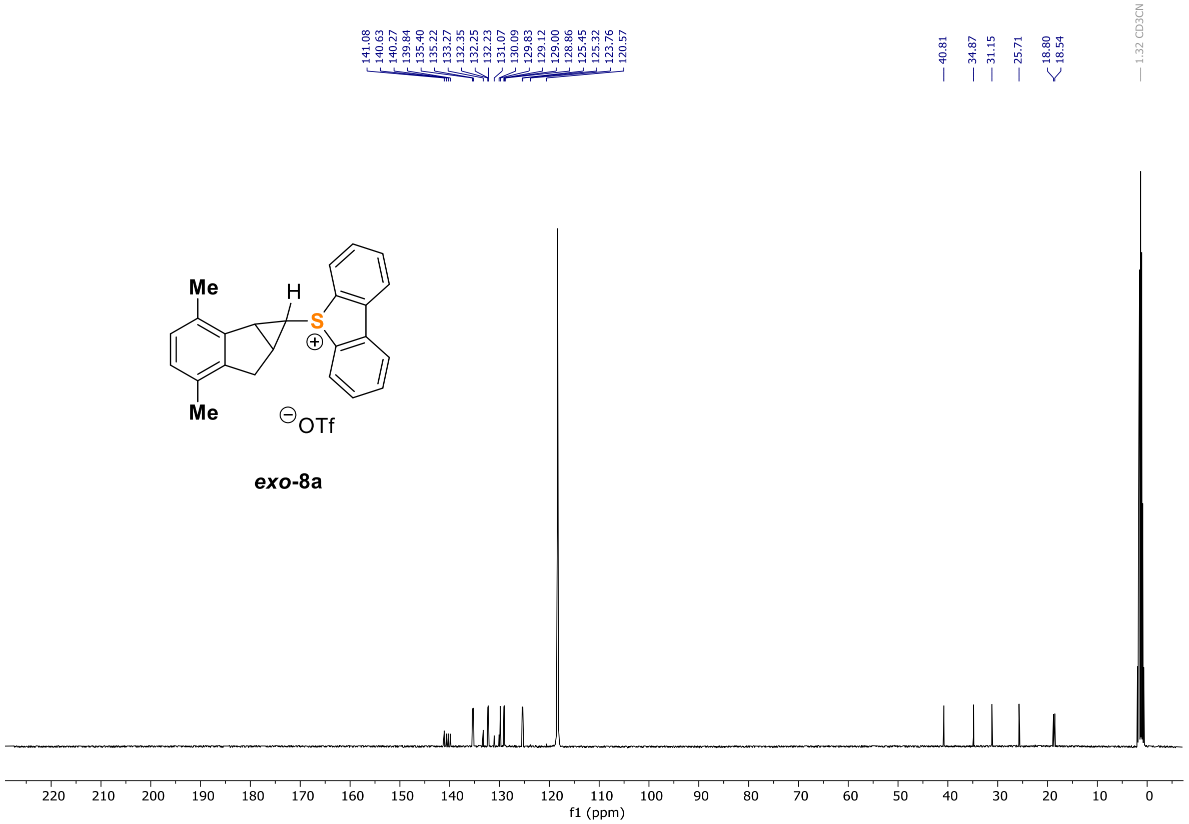

^{19}F NMR (377 MHz, CD_3CN)

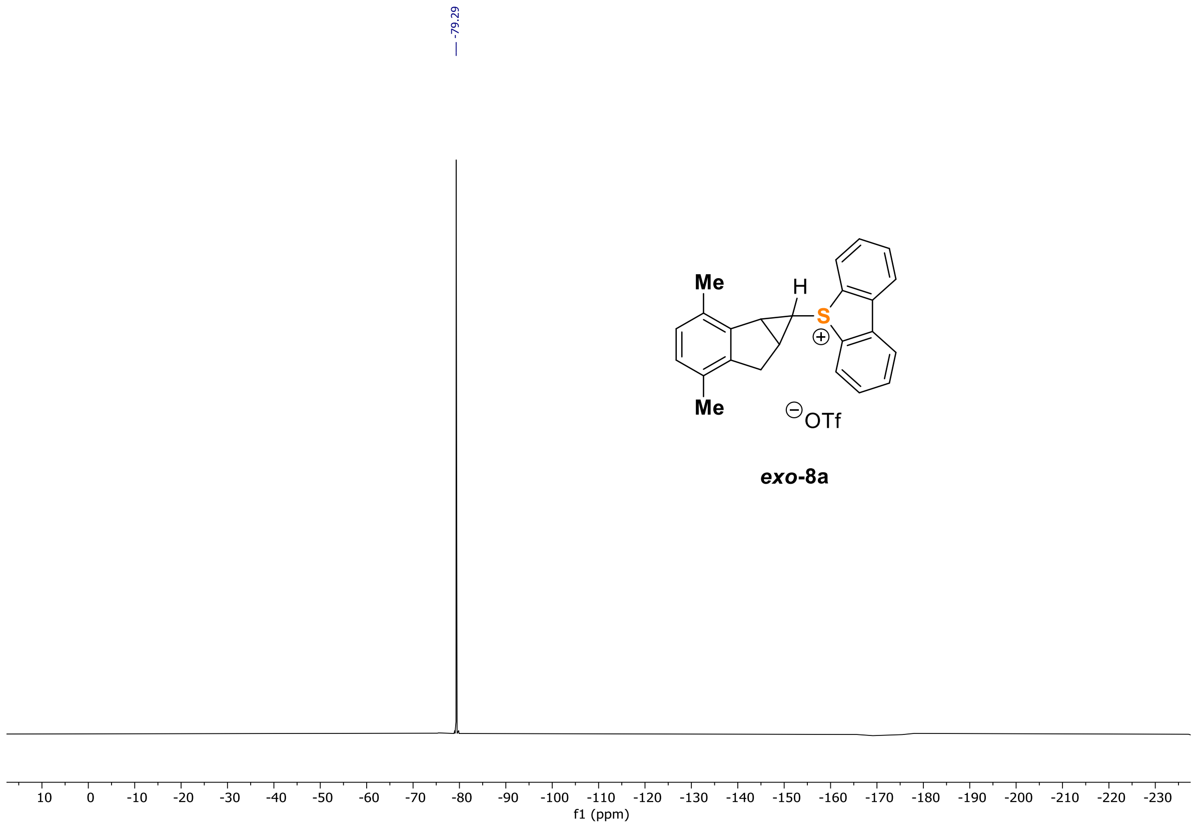

***exo*-8a**

Compound **12***

^{1}H NMR (400 MHz, $CDCl_3$)

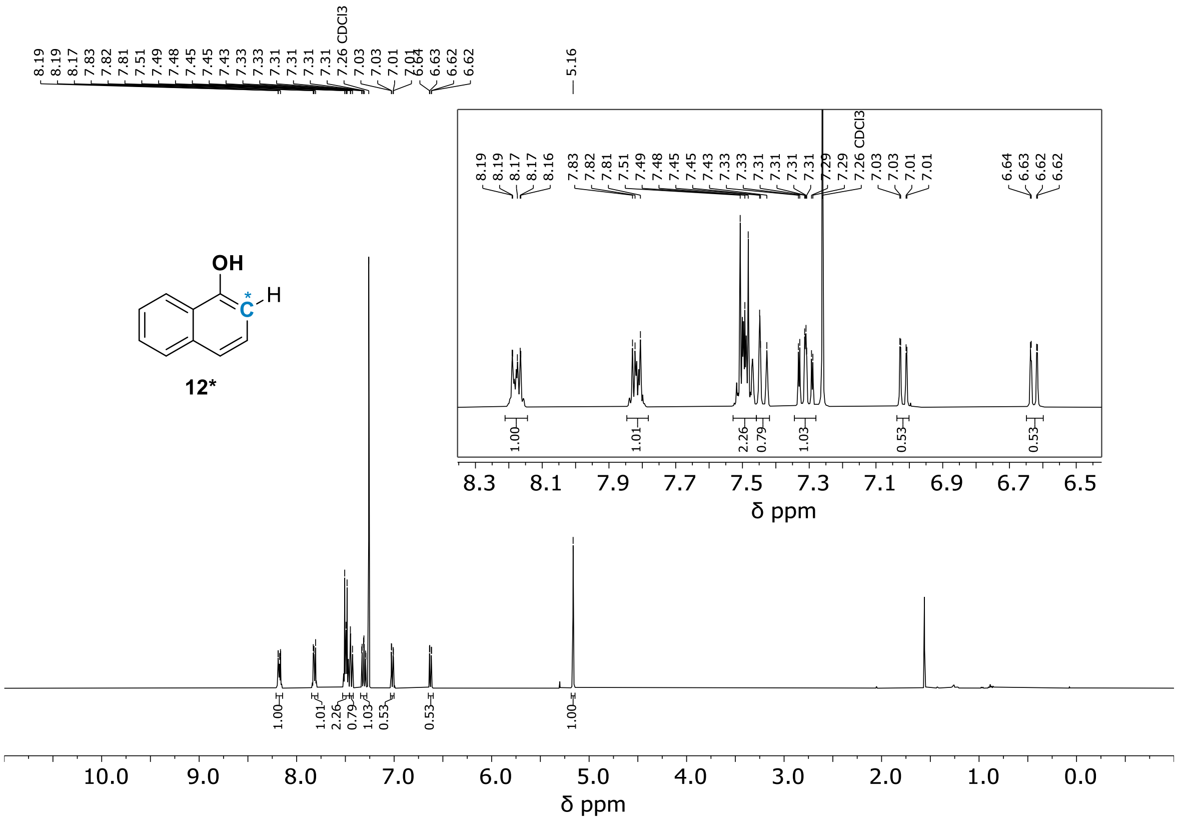

$^{13}C\{^{1}H\}$ NMR (101 MHz, $CDCl_3$)

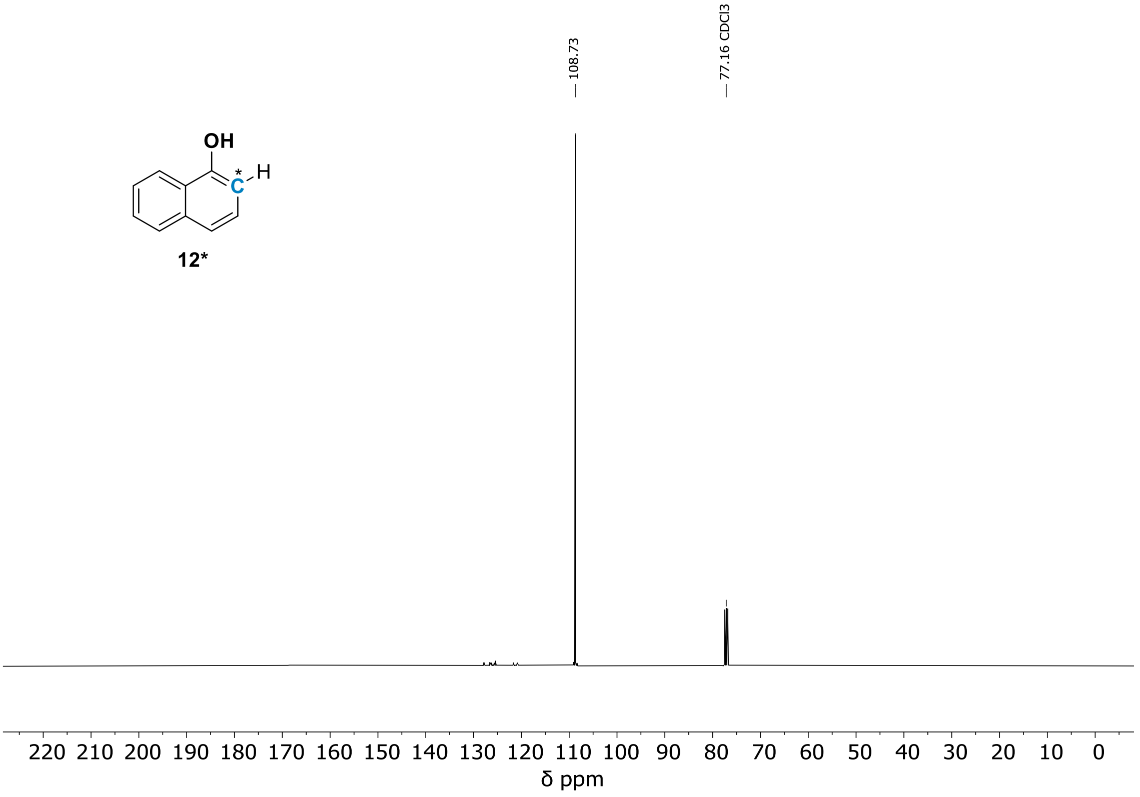

Compound **13***

^{1}H NMR (300 MHz, $CDCl_3$)

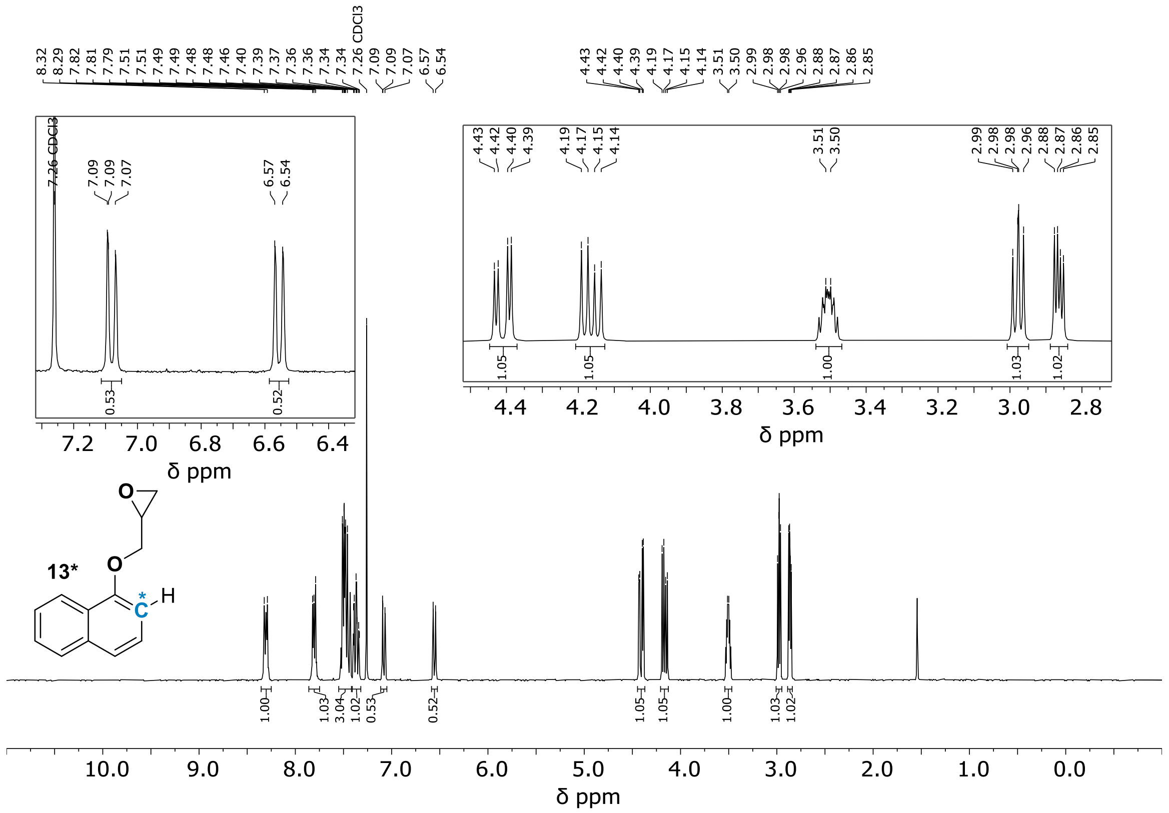

$^{13}C\{^{1}H\}$ NMR (75 MHz, $CDCl_3$)

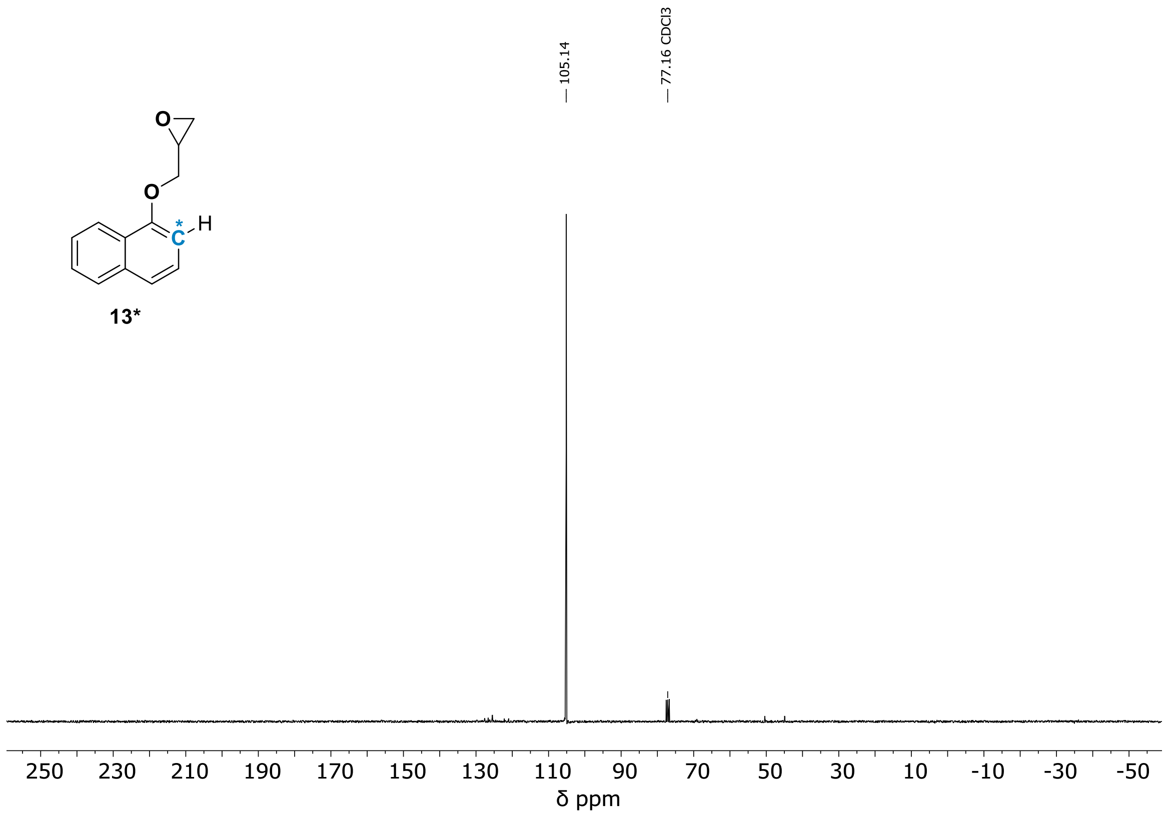

Compound **14***

^{1}H NMR (300 MHz, $CDCl_3$)

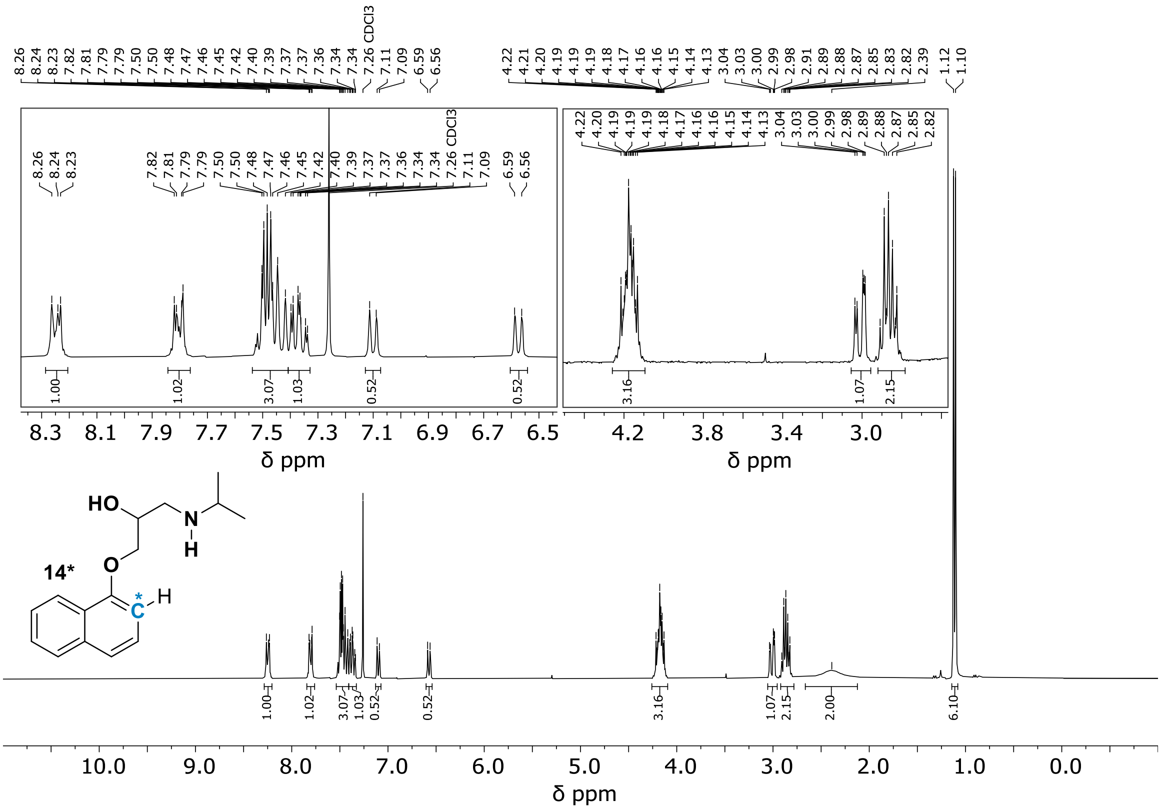

$^{13}C\{^{1}H\}$ NMR (126 MHz, $CDCl_3$)

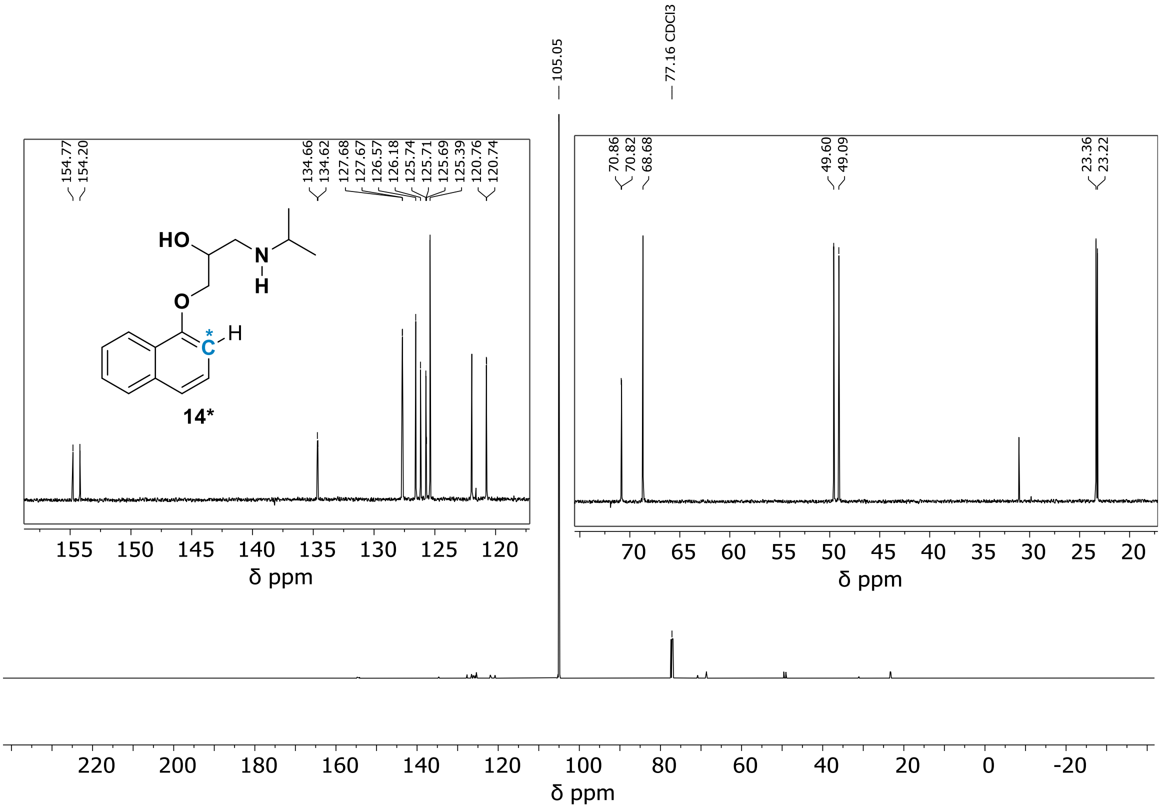

^{13}C NMR (126 MHz, $CDCl_3$)

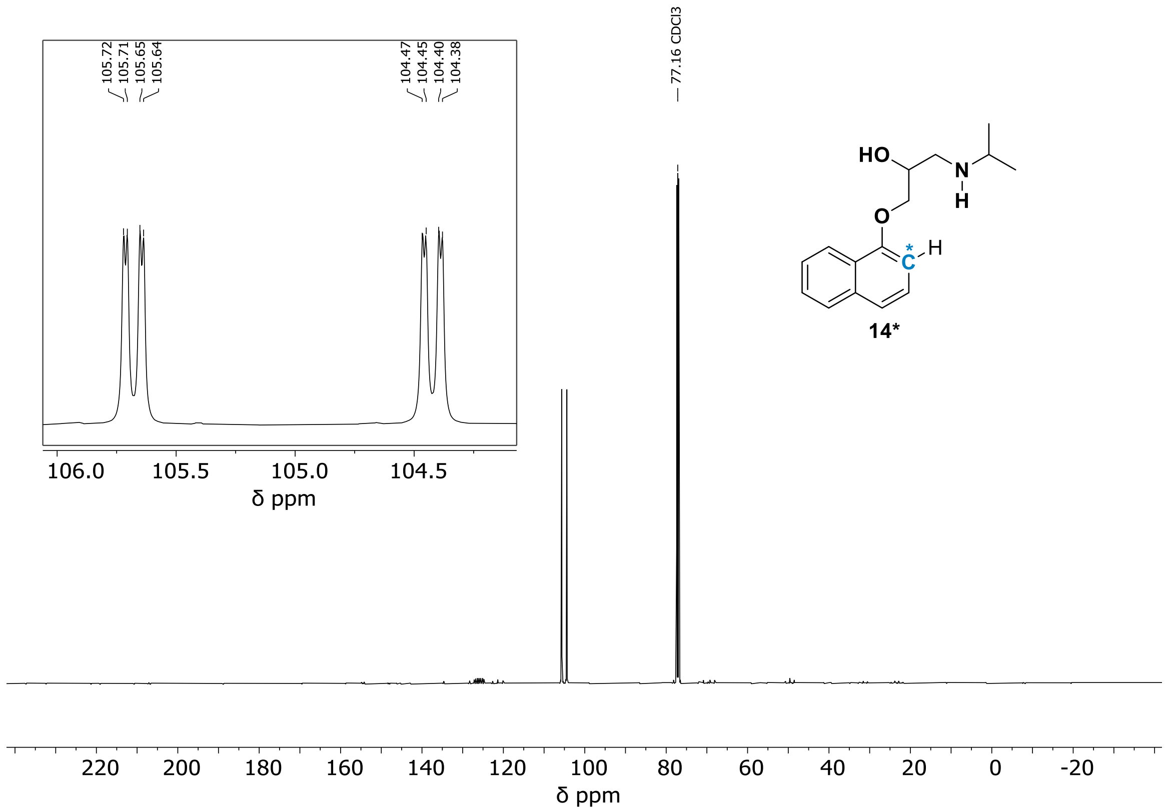

14*

Compound **9c**

^{1}H NMR (400 MHz, $CDCl_3$)

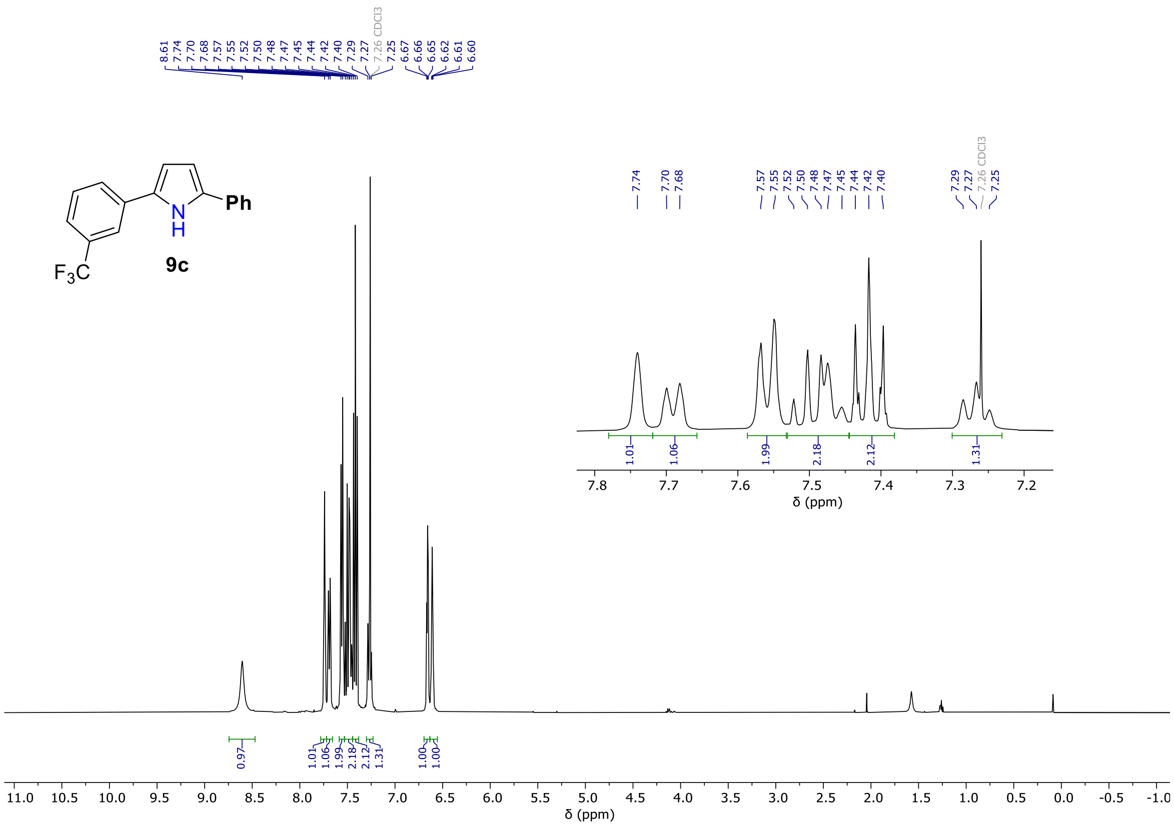

^{13}C{^{1}H} NMR (101 MHz, $CDCl_3$)

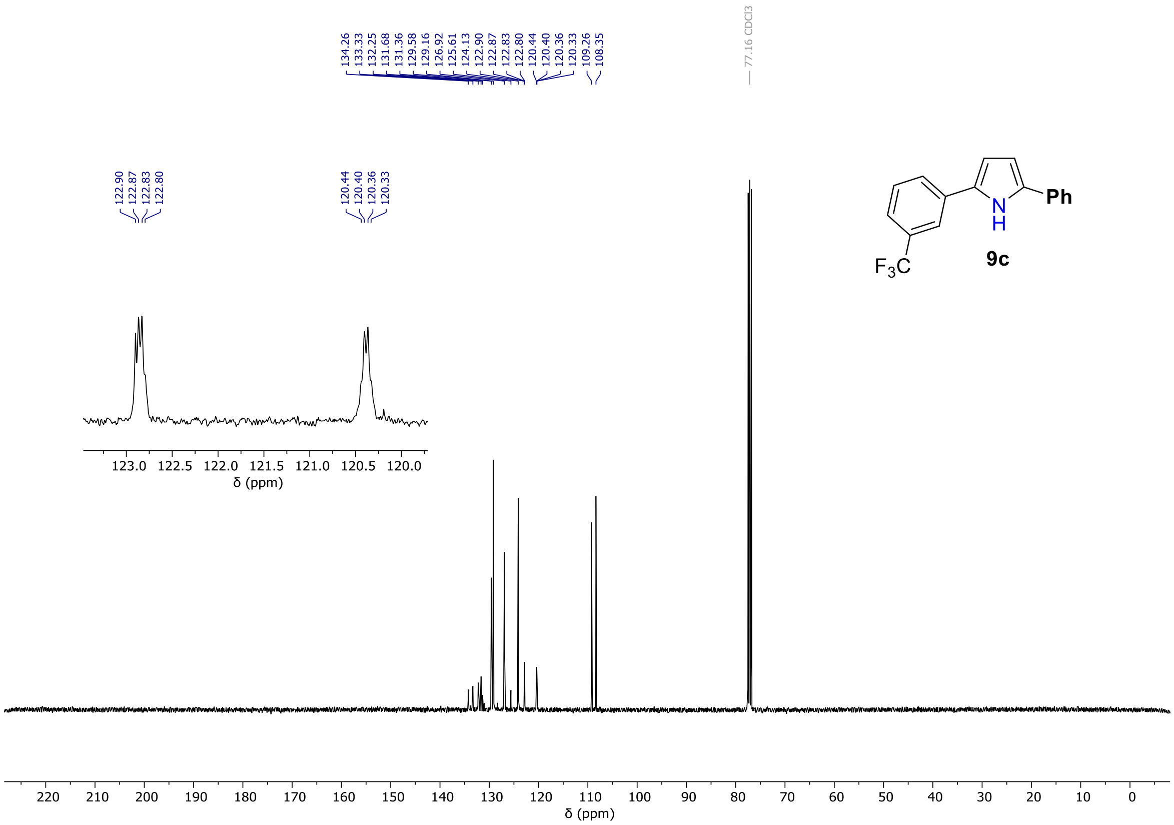

^{19}F NMR (377 MHz, $CDCl_3$)

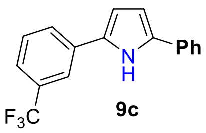

9c

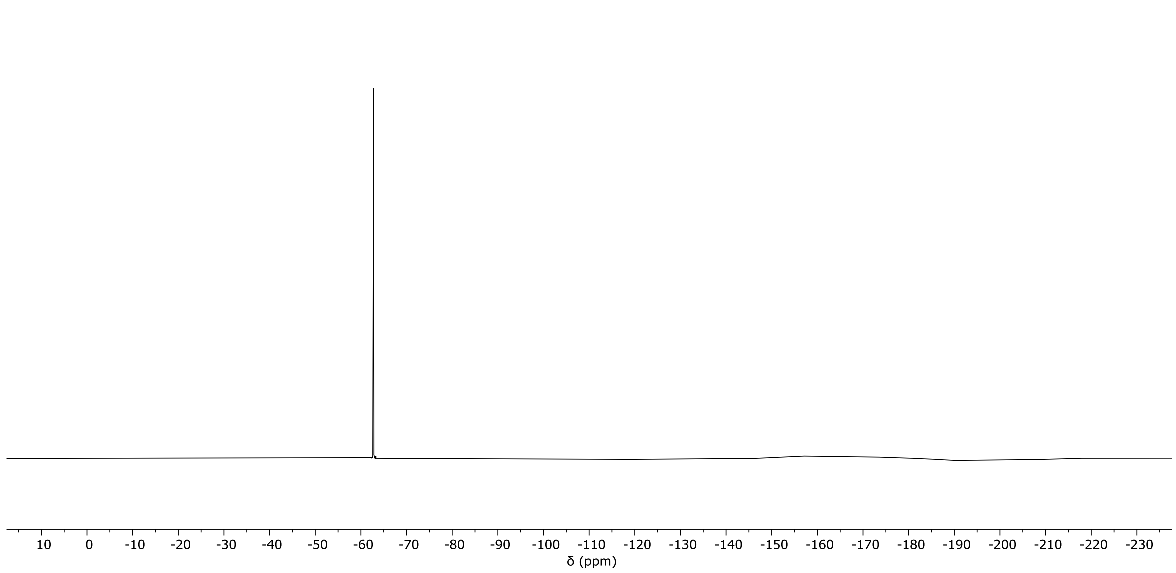

Compound **9j**

^{1}H NMR (400 MHz, $CDCl_3$)

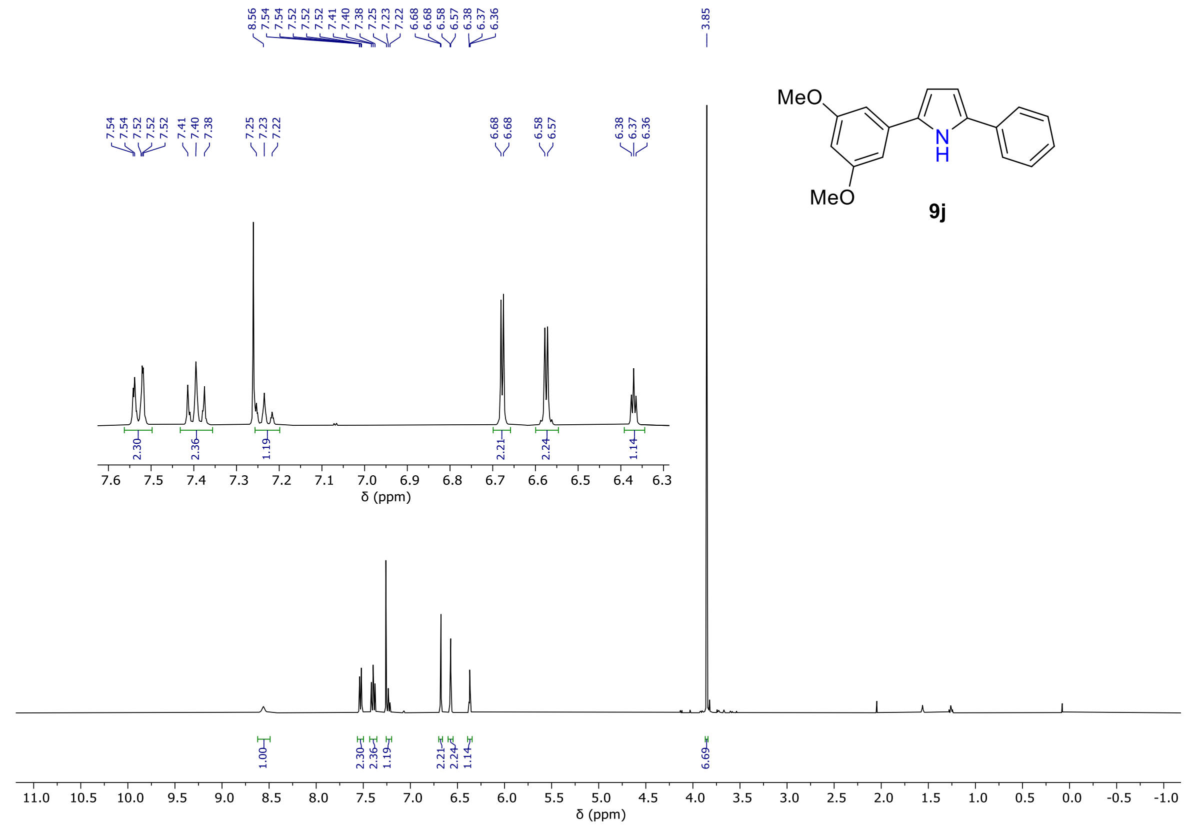

^{13}C{^{1}H} NMR (101 MHz, $CDCl_3$)

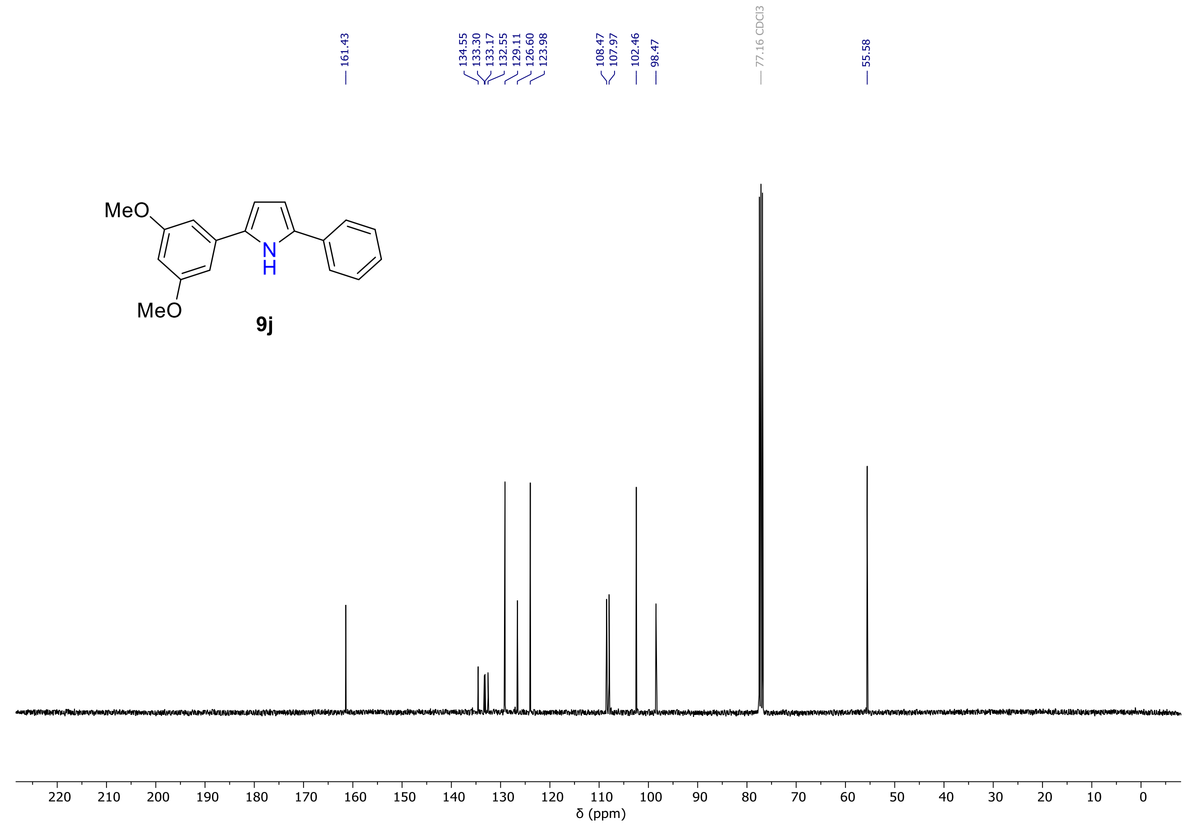

Compound **10a**

^{1}H NMR (400 MHz, $CDCl_3$)

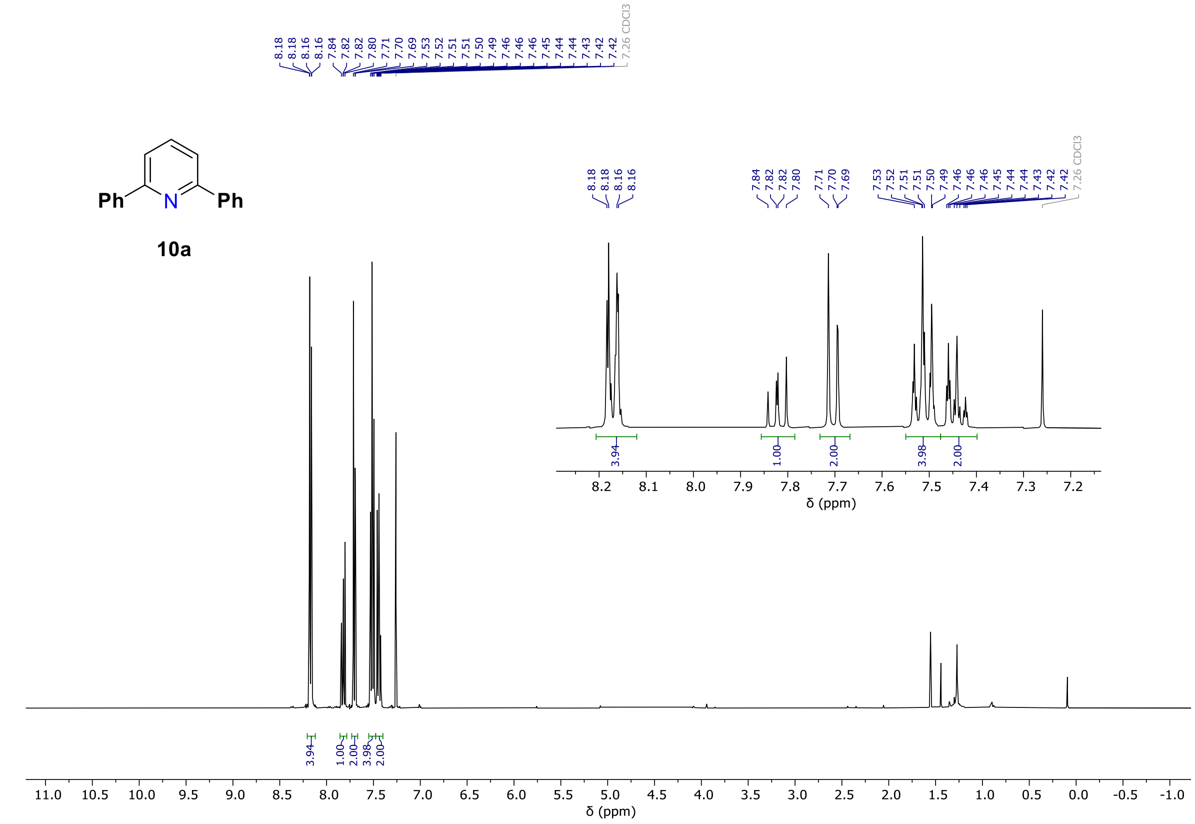

^{13}C{^{1}H} NMR (101 MHz, $CDCl_3$)

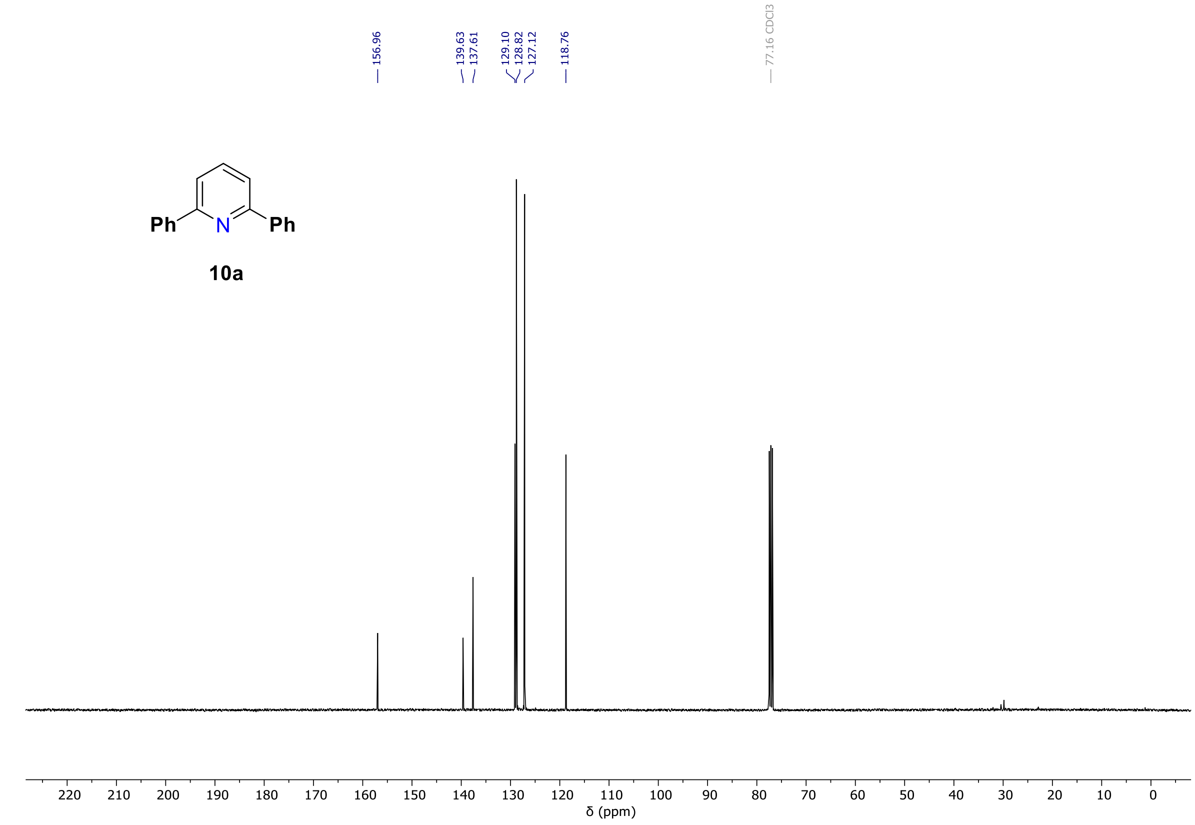

Compound **10a***

^{1}H NMR (500 MHz, $CDCl_3$)

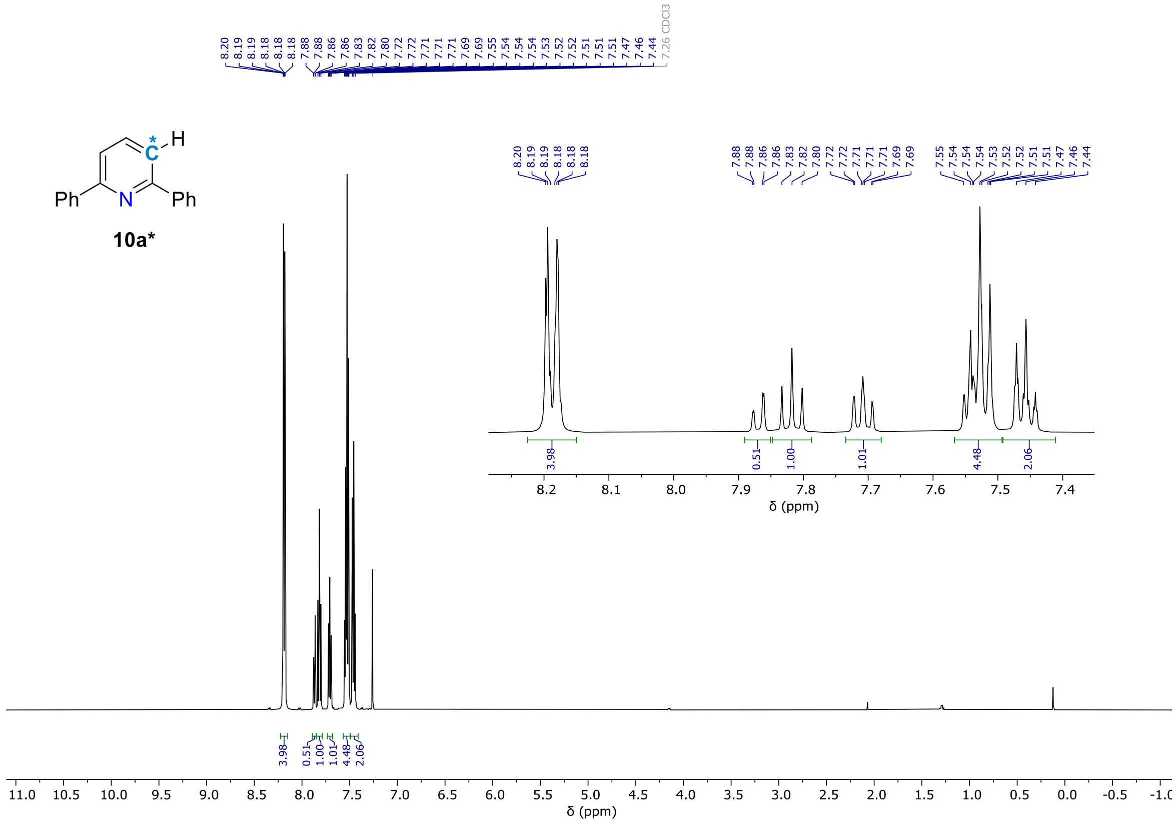

^{13}C{^{1}H} NMR (126 MHz, $CDCl_3$)

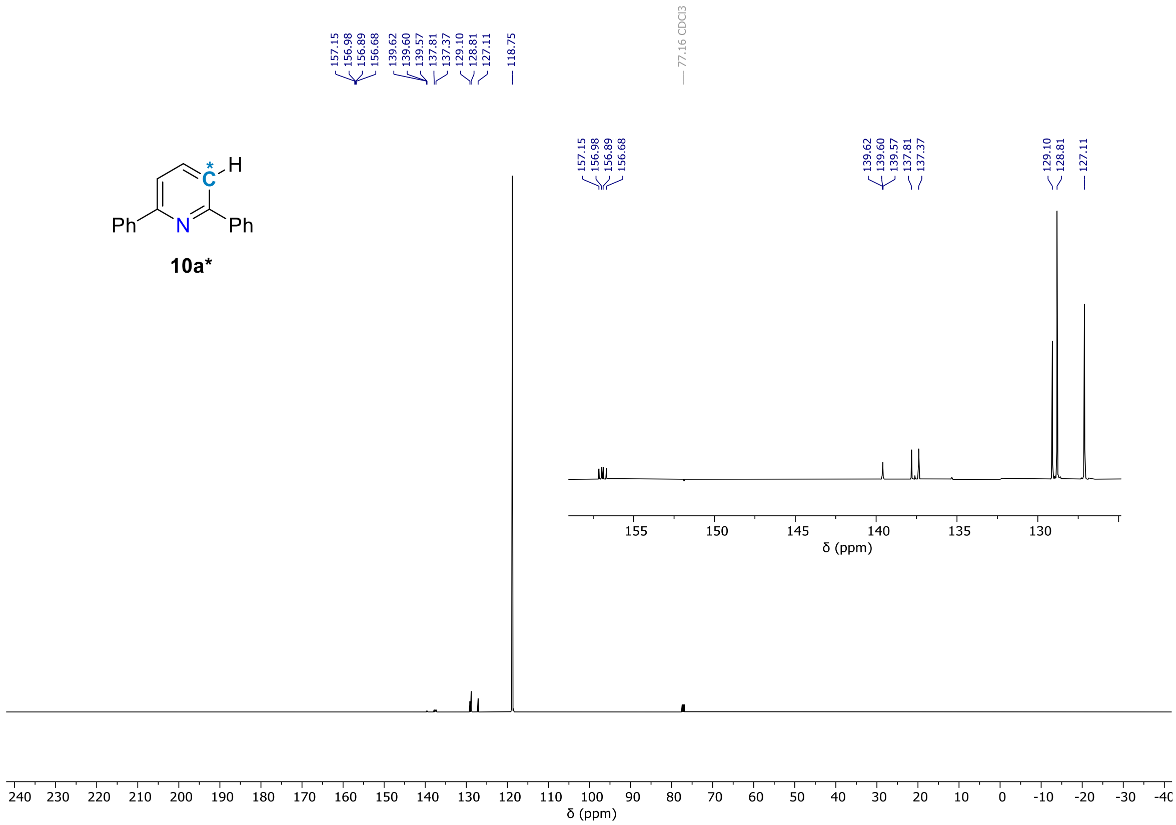

^{13}C NMR (126 MHz, $CDCl_3$)

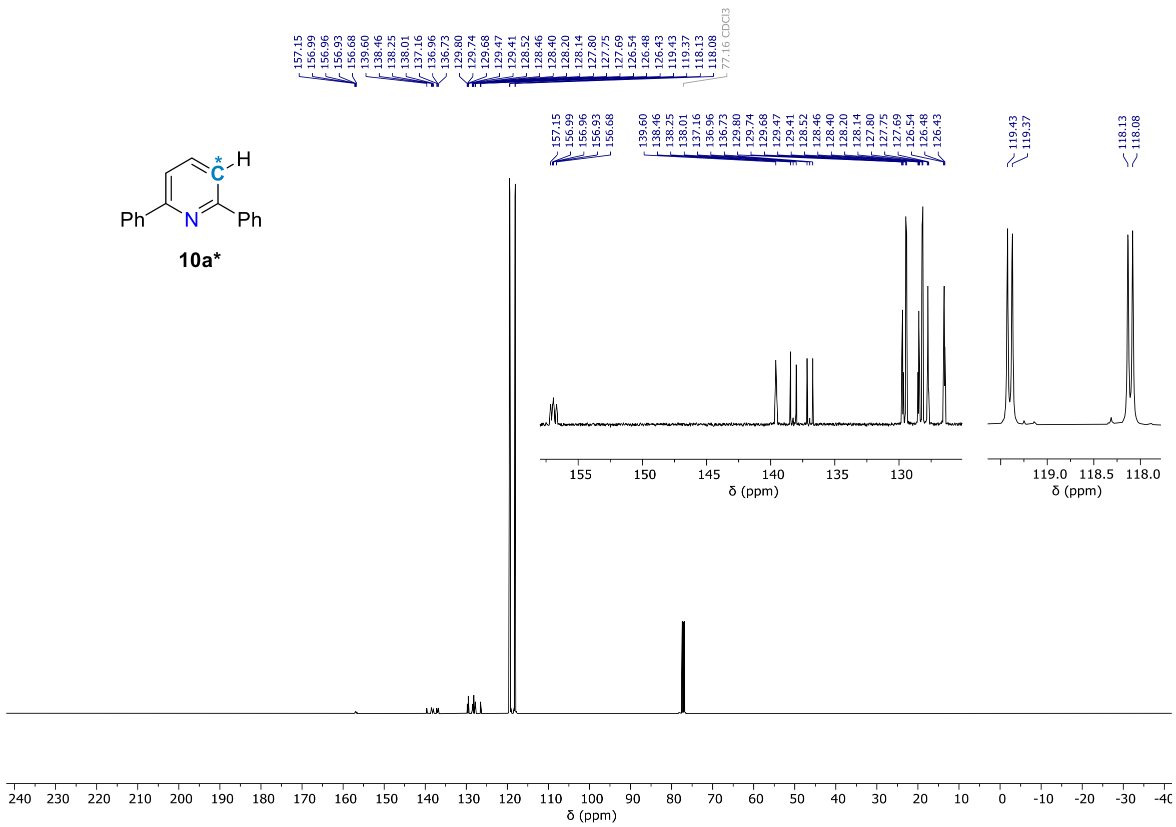

Compound **10b**

^{1}H NMR (400 MHz, $CDCl_3$)

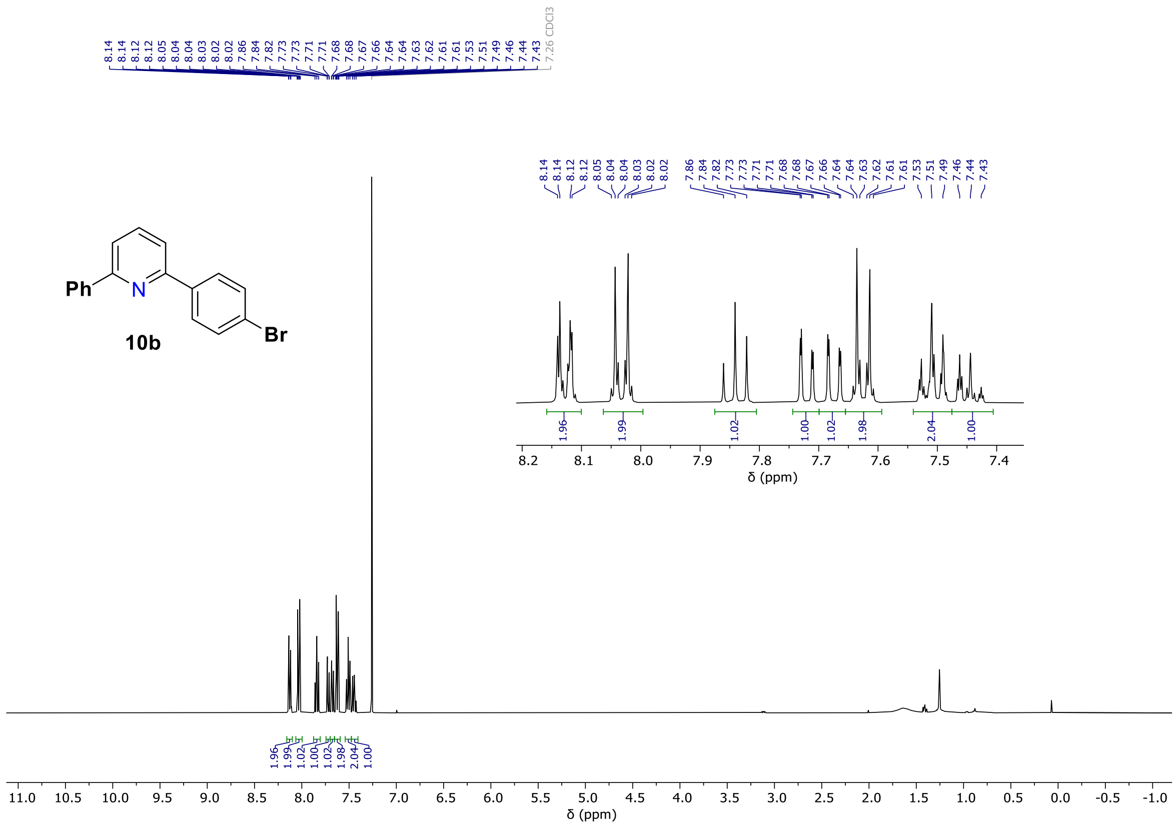

^{13}C{^{1}H} NMR (101 MHz, $CDCl_3$)

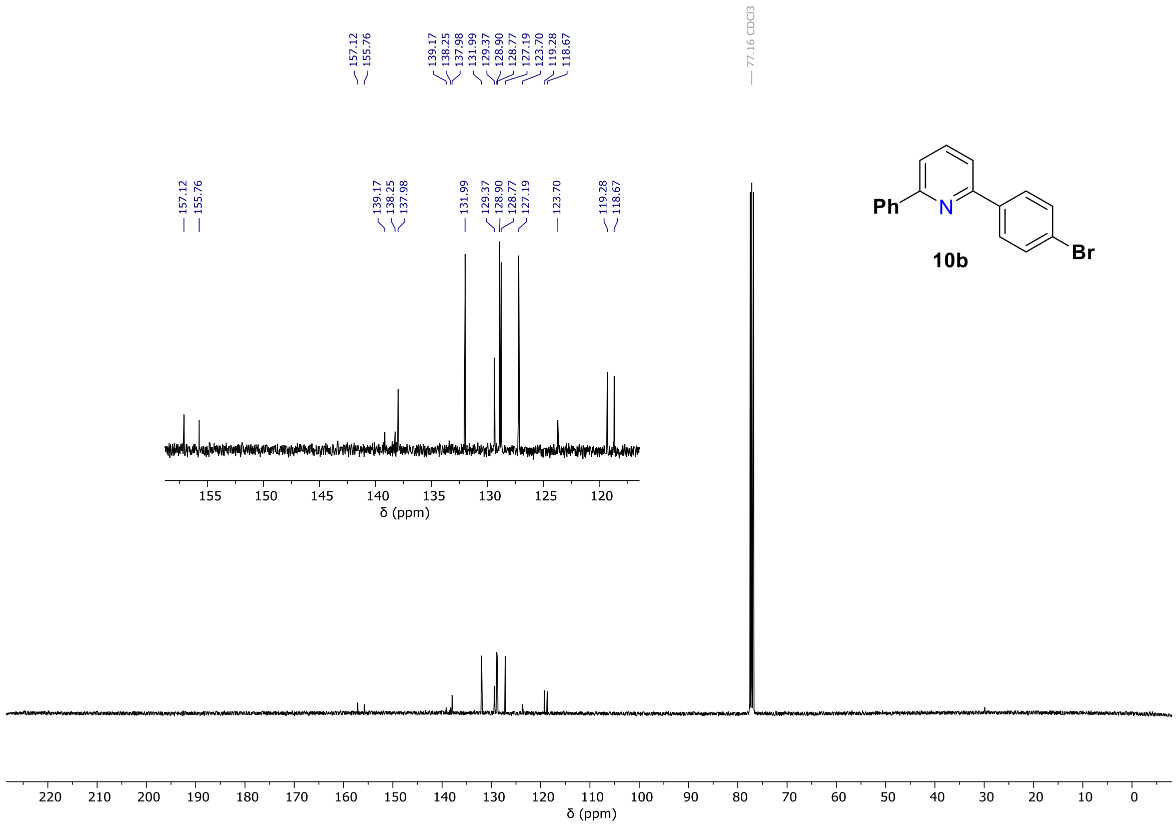

Compound **10c**

^{1}H NMR (400 MHz, $CDCl_3$)

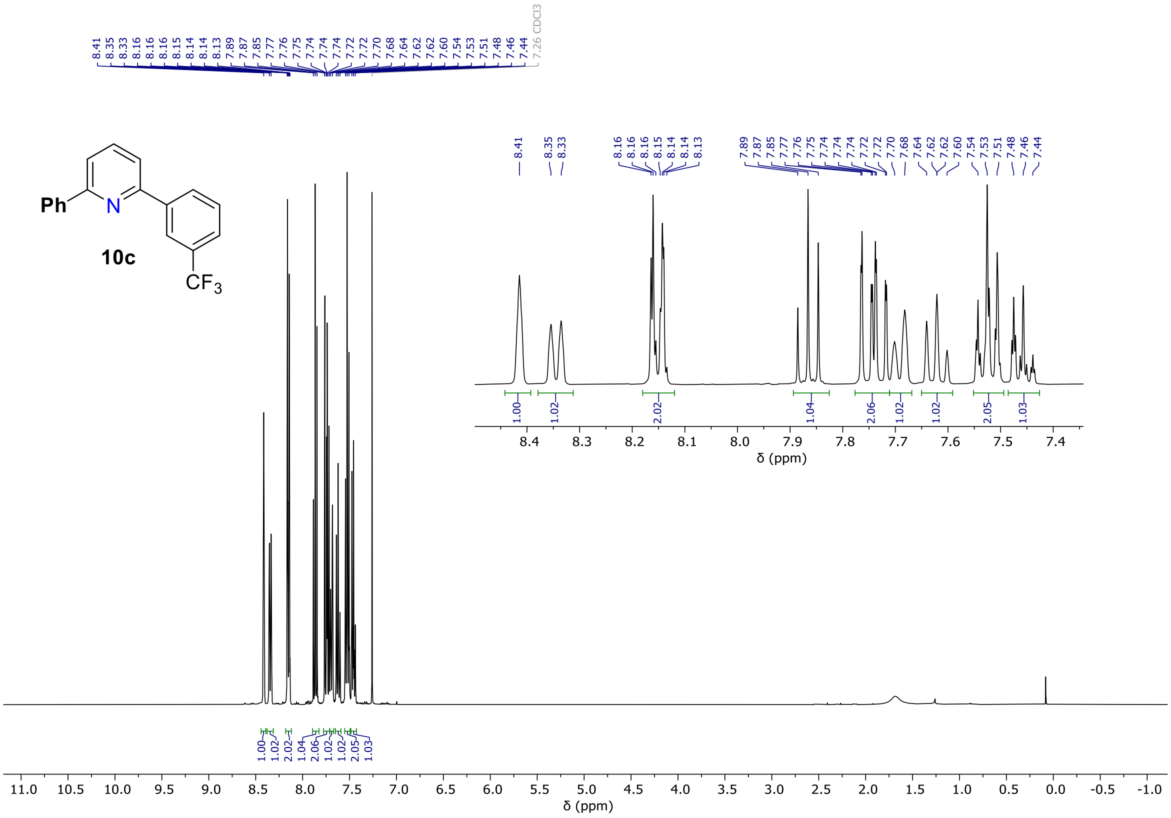

$^{13}C\{^{1}H\}$ NMR (101 MHz, $CDCl_3$)

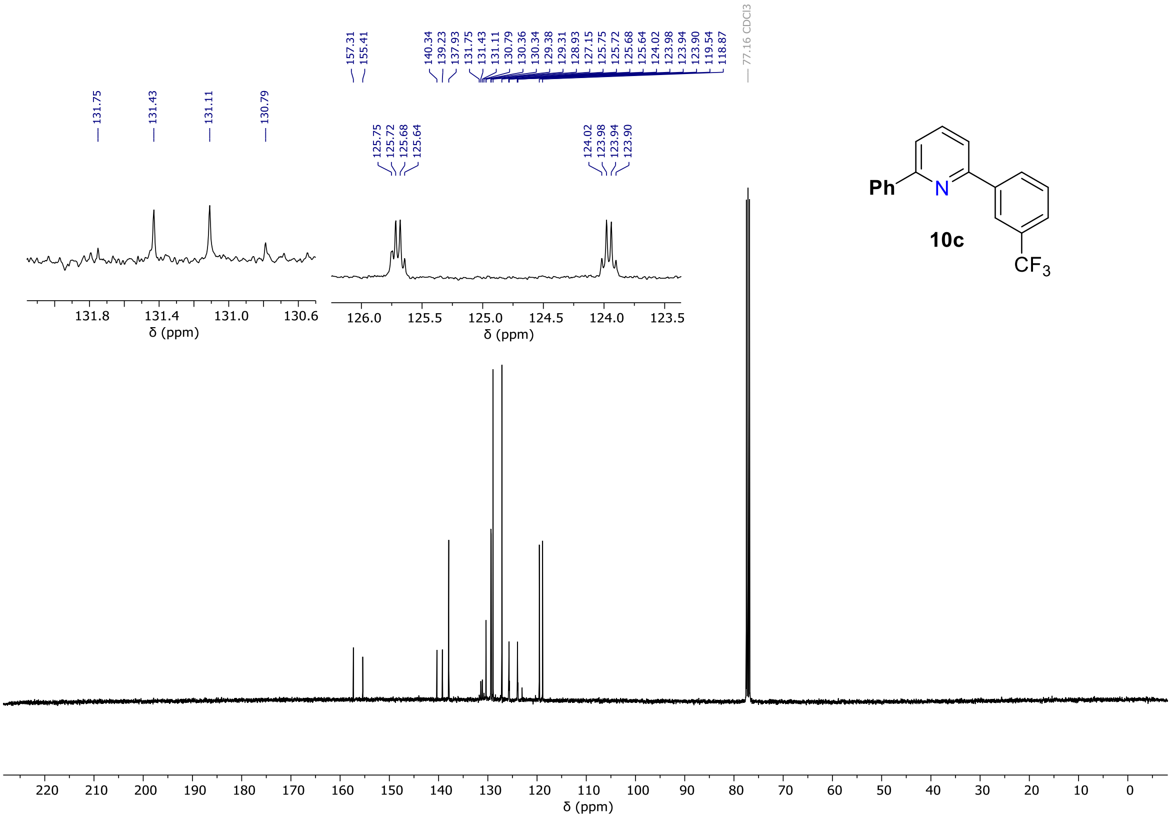

^{19}F NMR (282 MHz, $CDCl_3$)

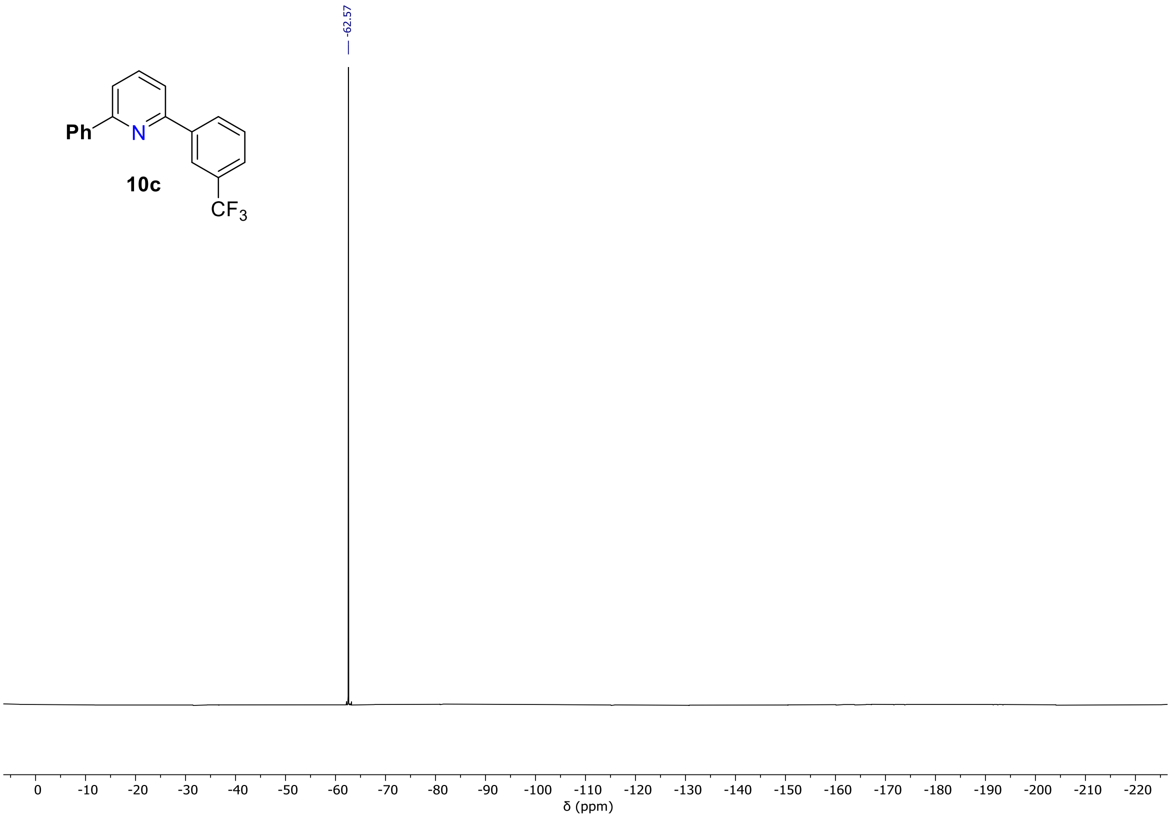

Compound **10d**

^{1}H NMR (400 MHz, $CDCl_3$)

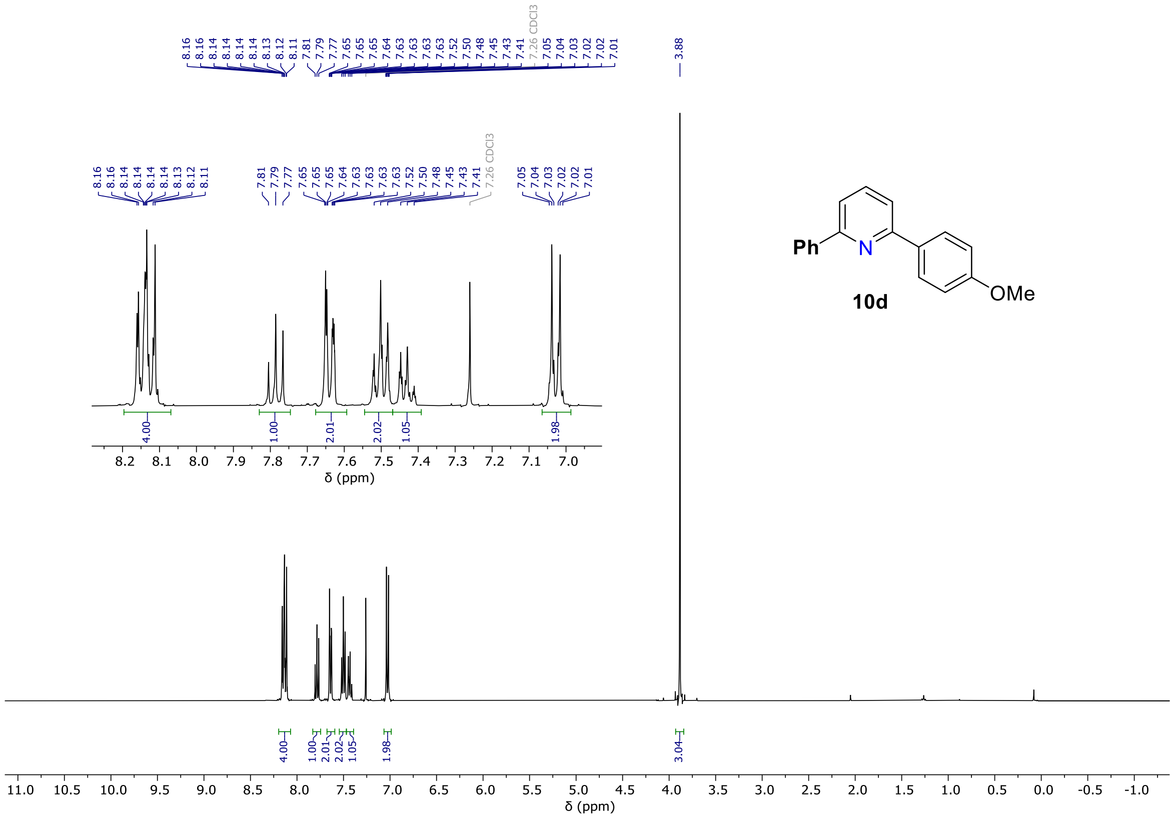

$^{13}C\{^{1}H\}$ NMR (101 MHz, $CDCl_3$)

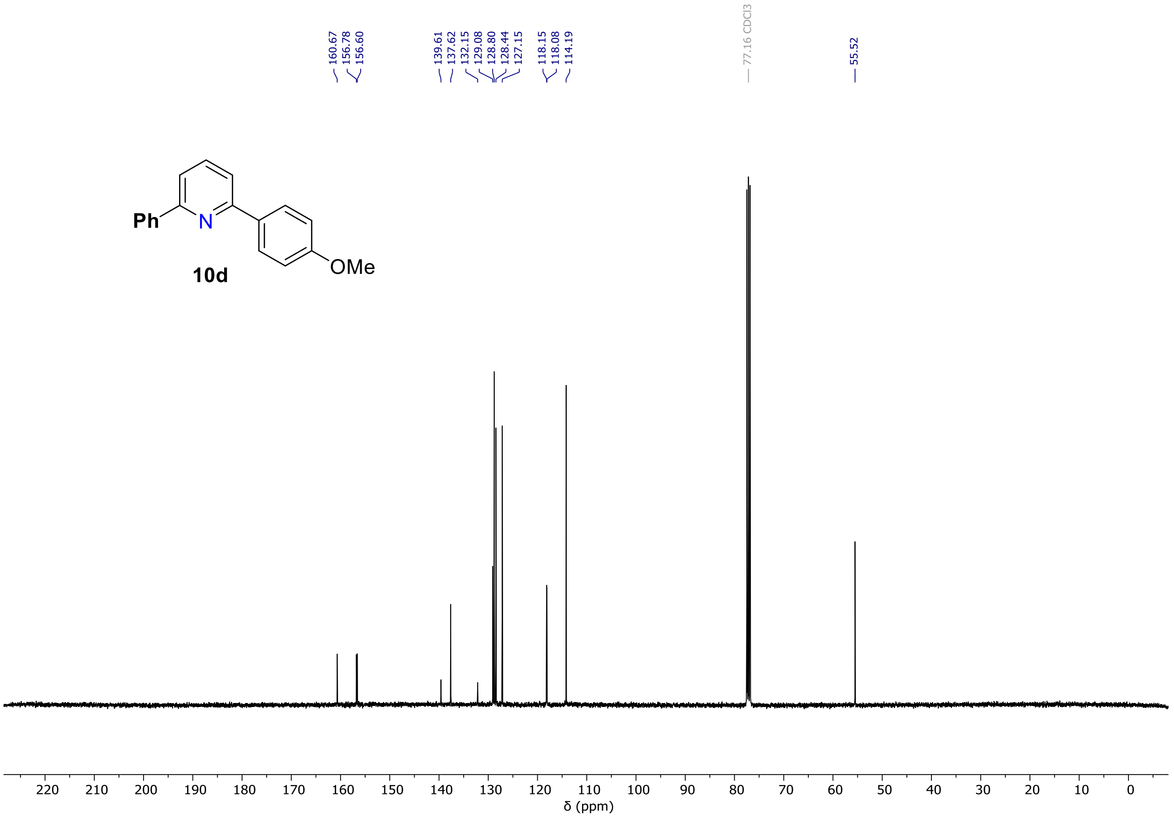

Compound **10e**

^{1}H NMR (400 MHz, $CDCl_3$)

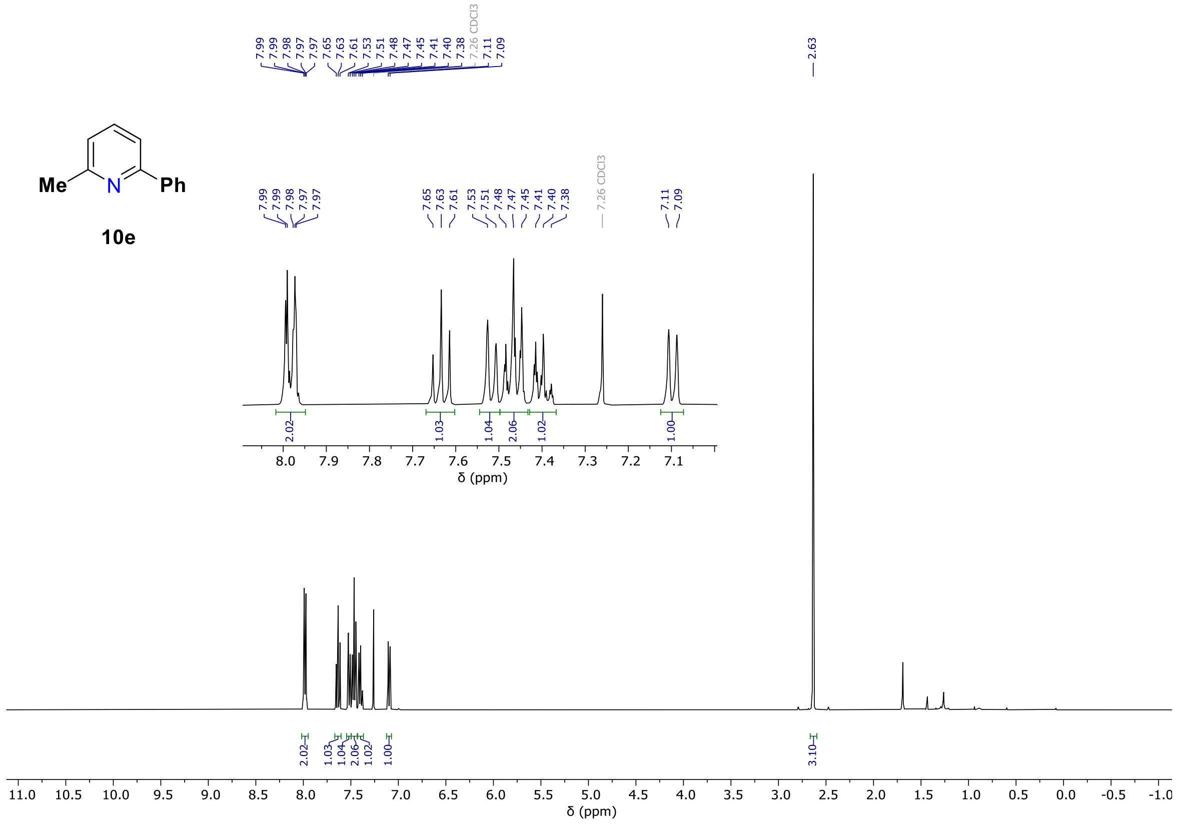

^{13}C{^{1}H} NMR (101 MHz, $CDCl_3$)

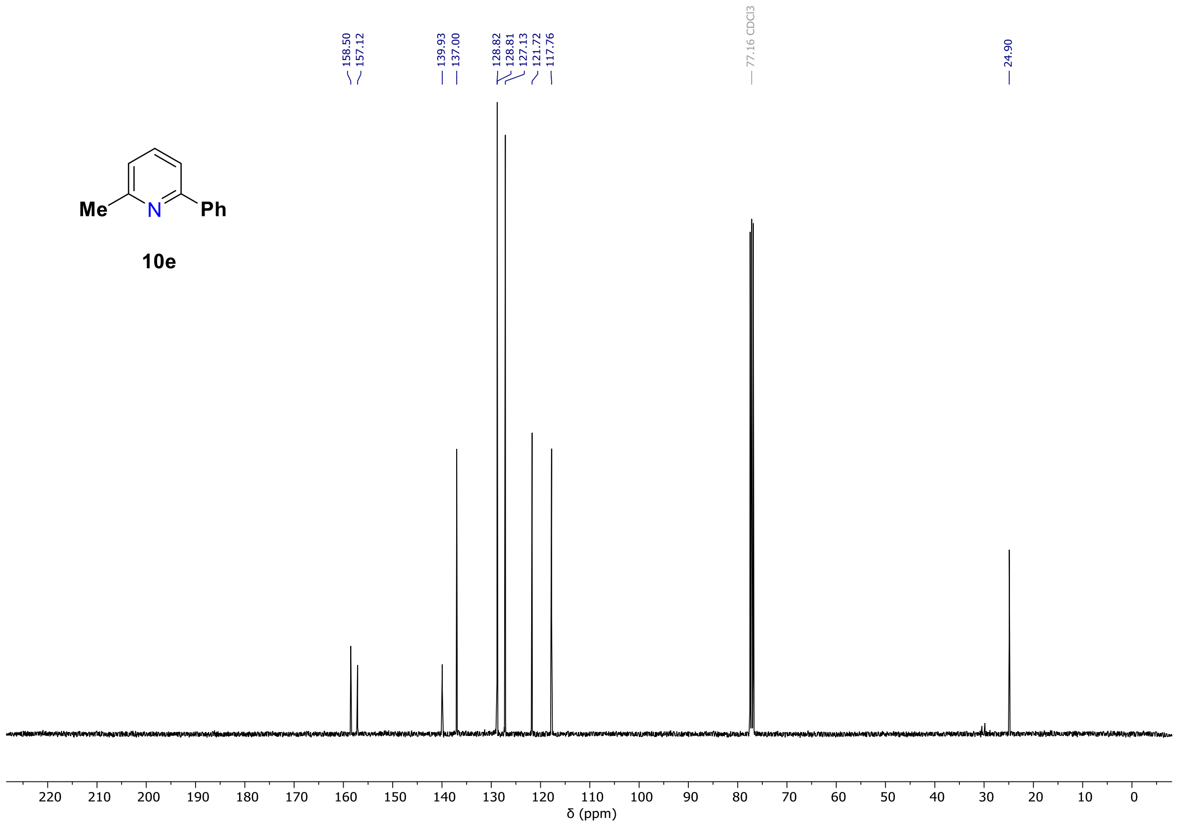

Compound **10f**

^{1}H NMR (400 MHz, $CDCl_3$)

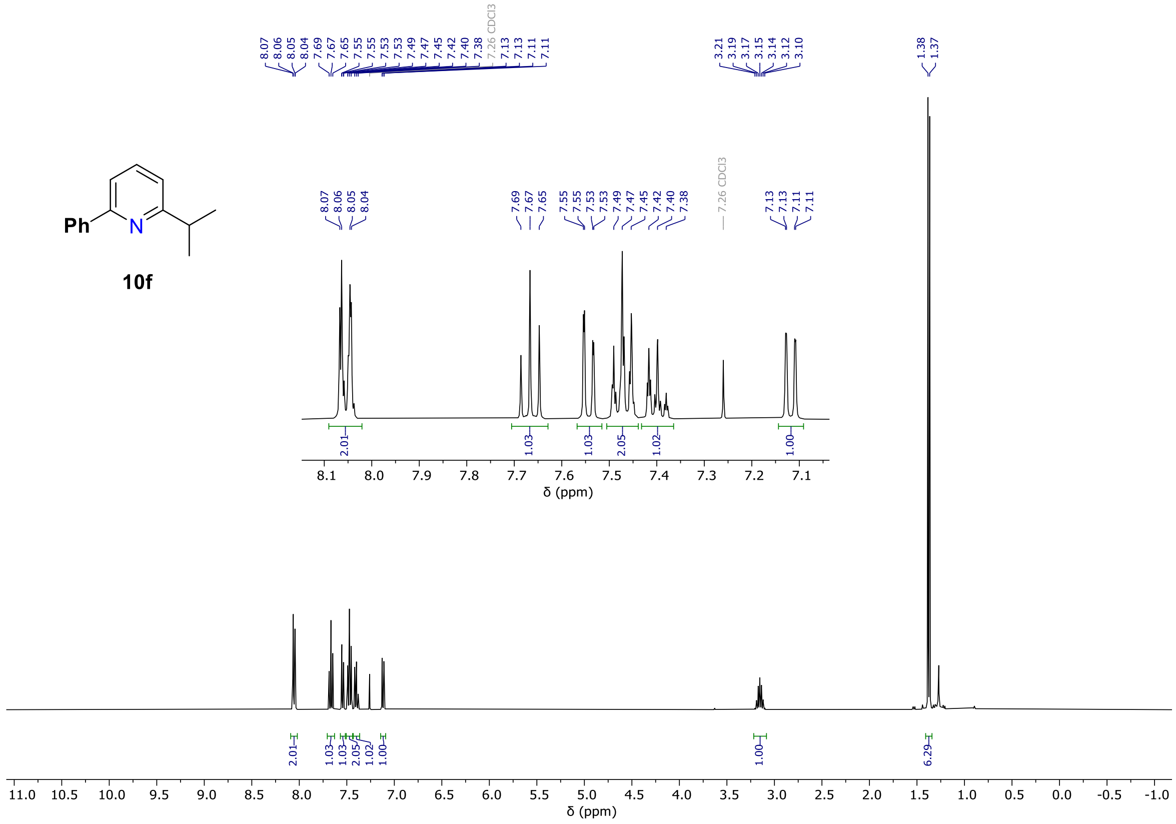

^{13}C{^{1}H} NMR (101 MHz, $CDCl_3$)

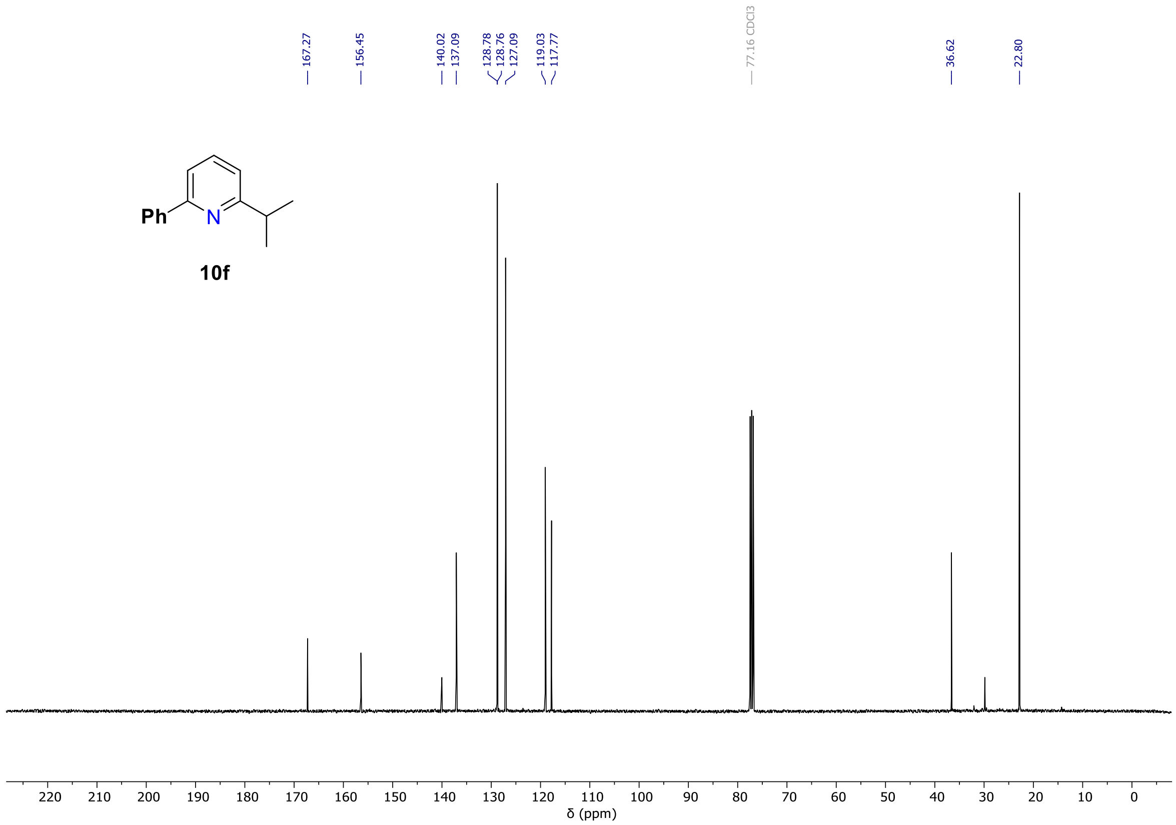

Compound **10f***

^{1}H NMR (500 MHz, $CDCl_3$)

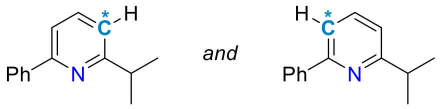

10f*I **10f*II**

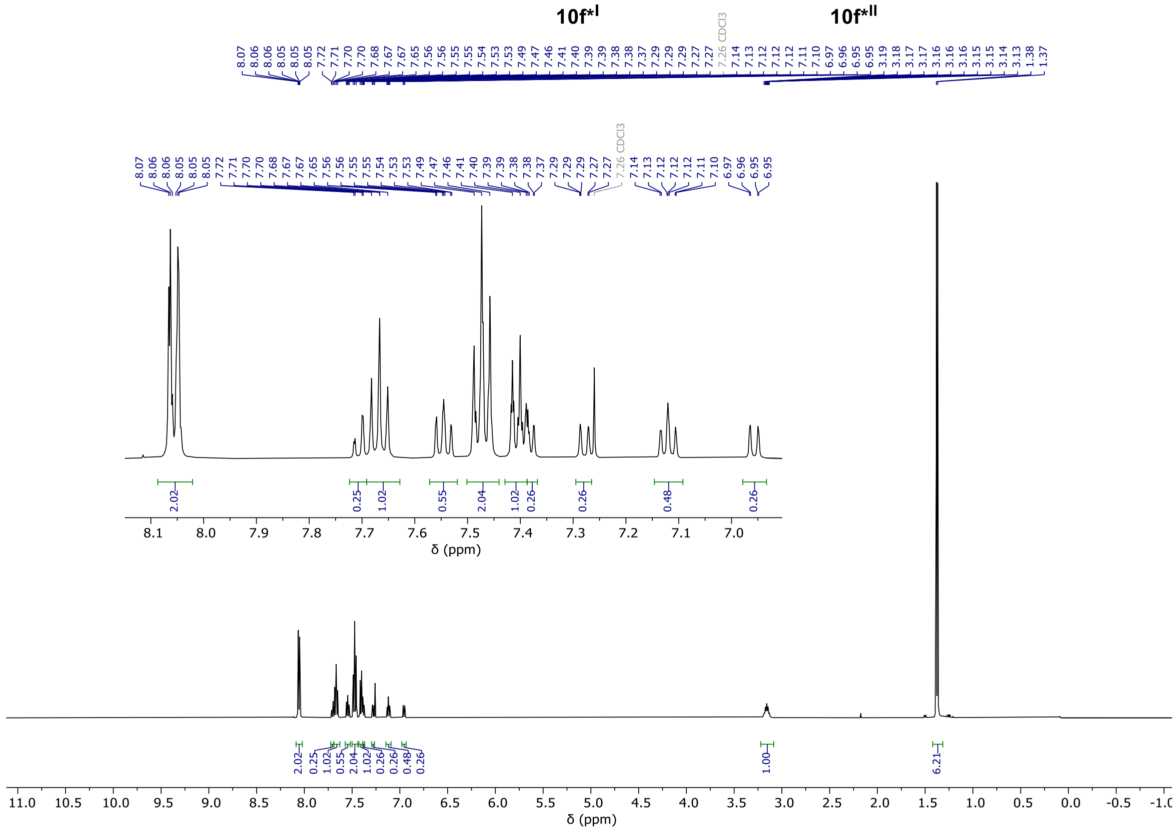

^{13}C{^{1}H} NMR (126 MHz, $CDCl_3$)

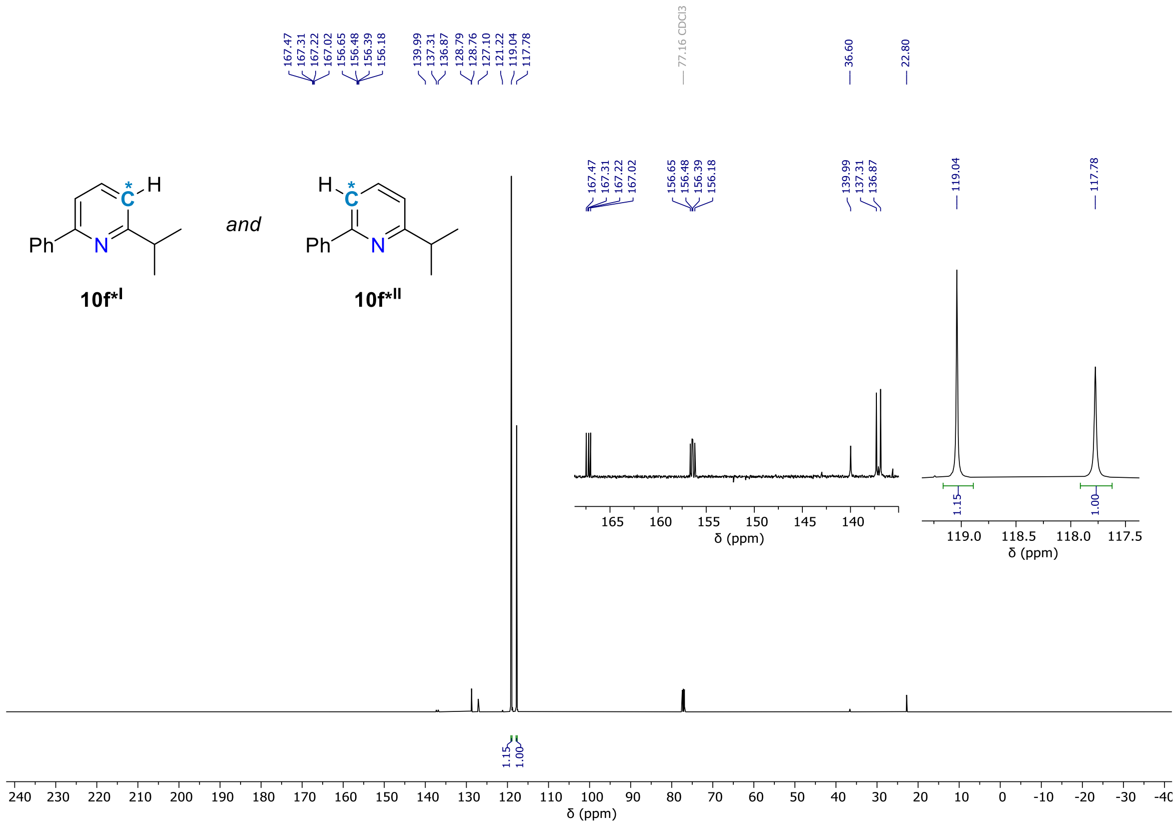

10f*I **10f*II**

^{13}C NMR (126 MHz, $CDCl_3$)

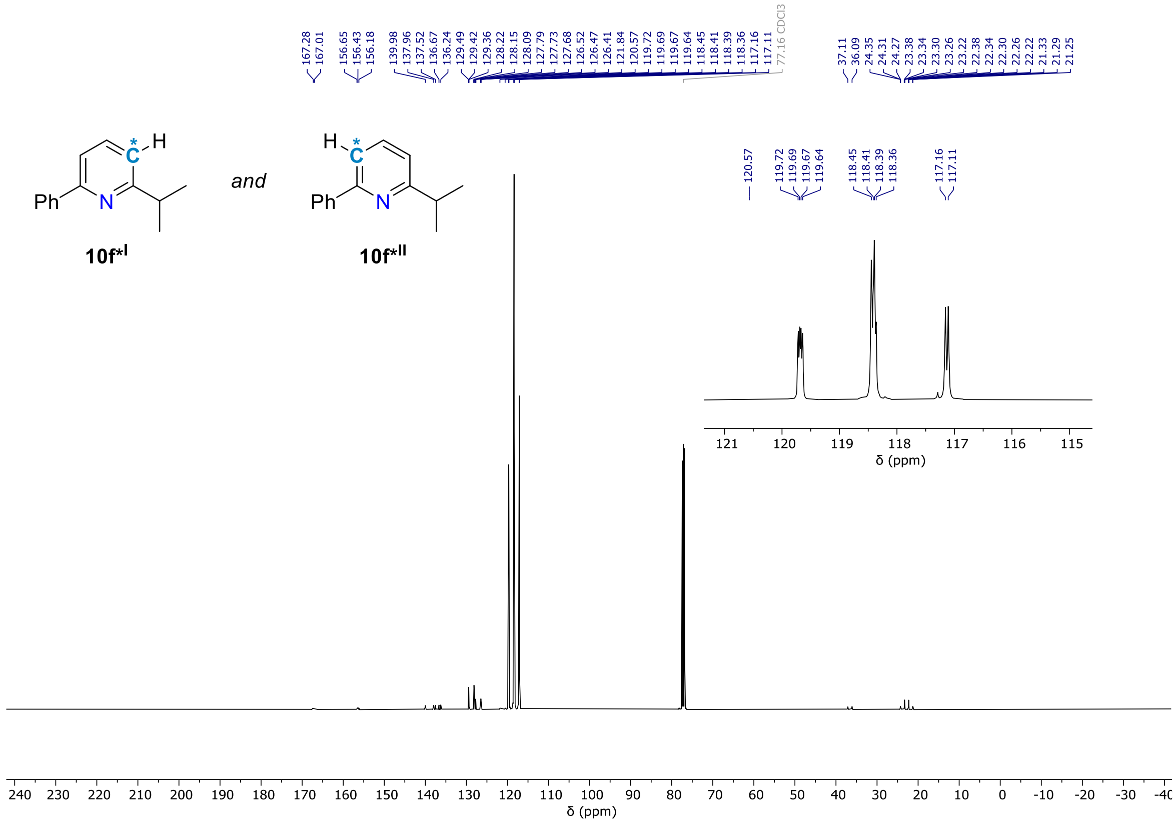

Compound **10g**

^{1}H NMR (400 MHz, $CDCl_3$)

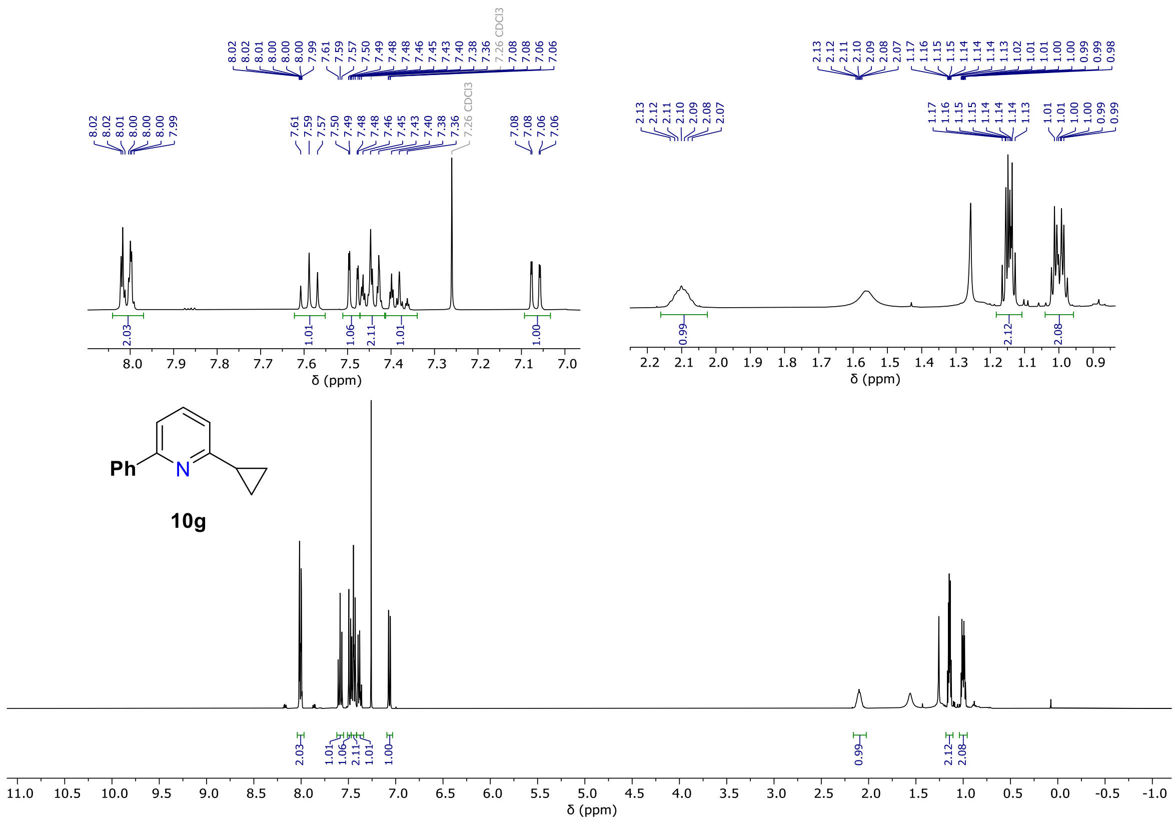

^{13}C{^{1}H} NMR (101 MHz, $CDCl_3$)

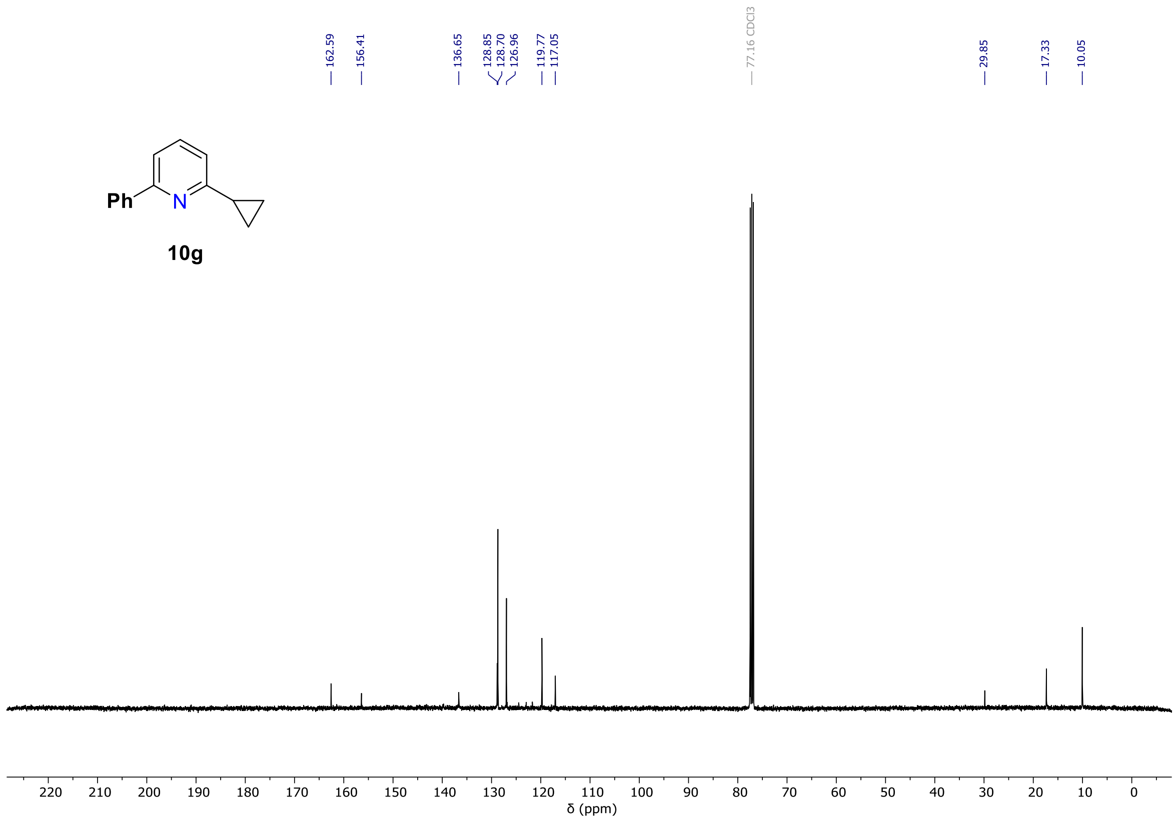

Compound **10h**

^{1}H NMR (400 MHz, $CDCl_3$)

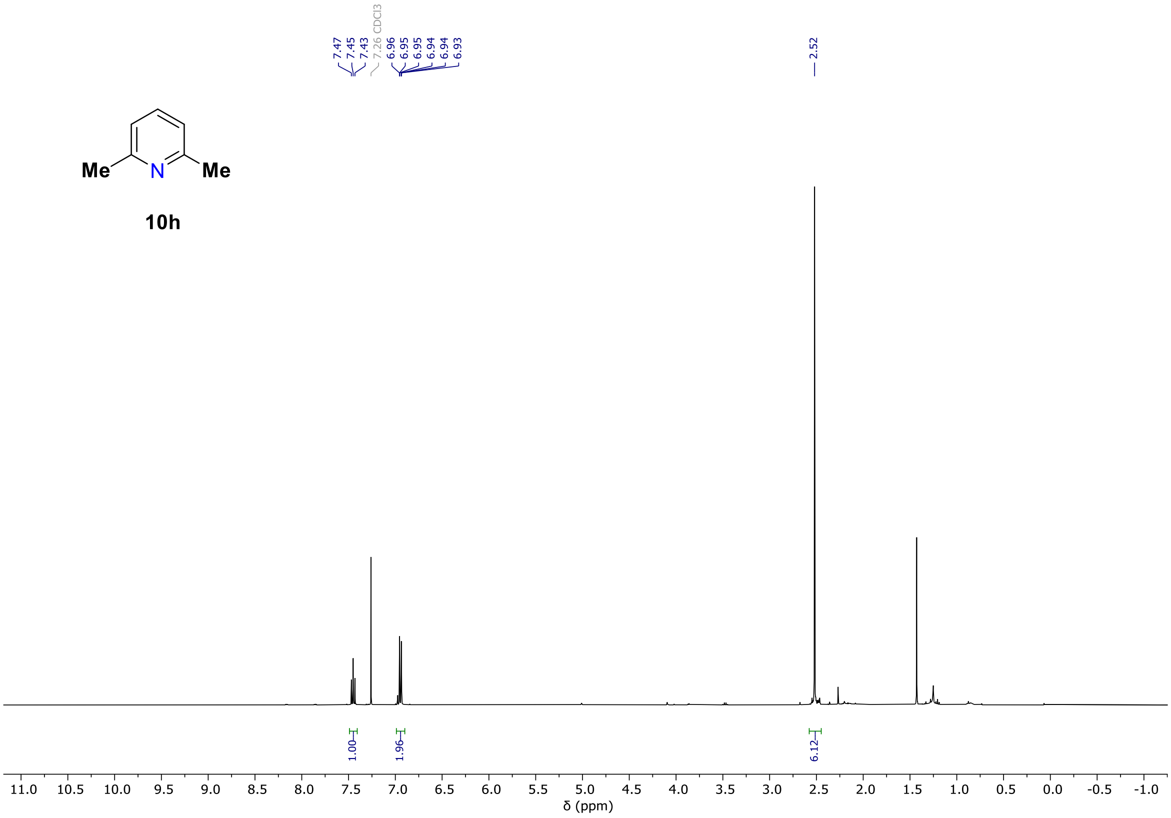

^{13}C{^{1}H} NMR (101 MHz, $CDCl_3$)

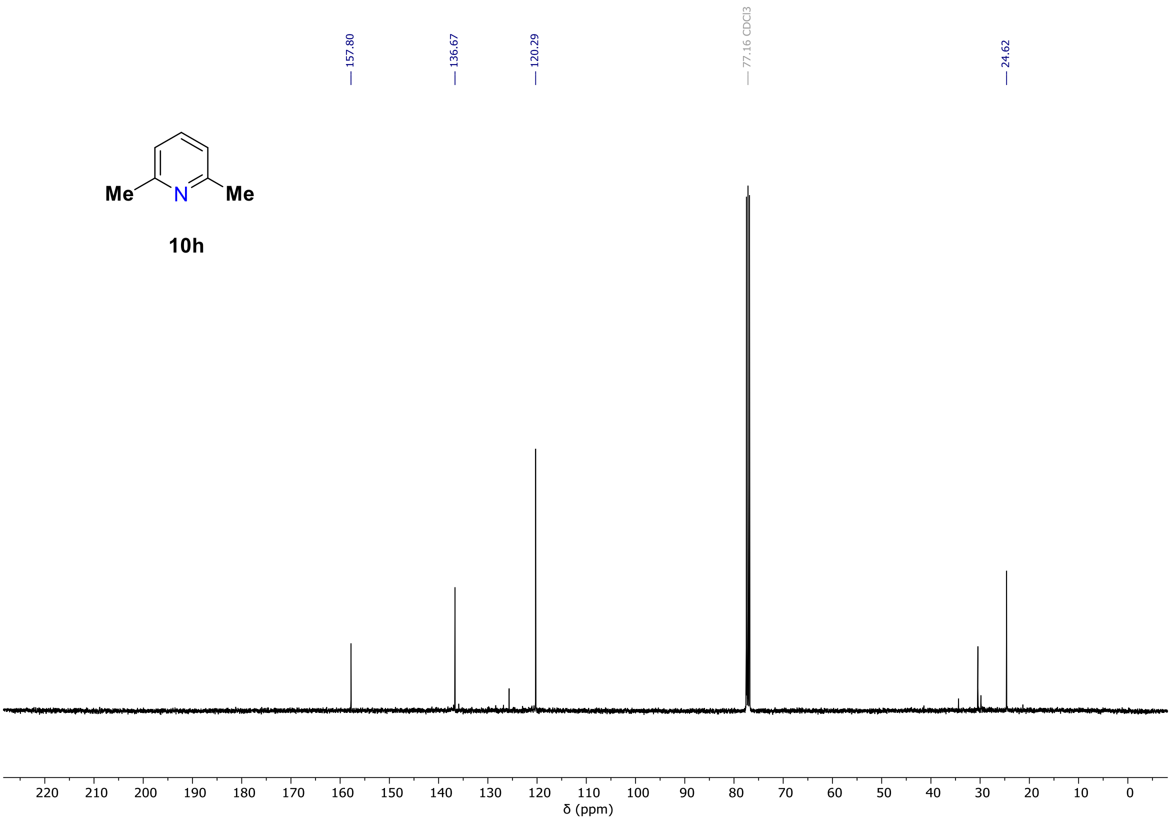

Compound **10i**

^{1}H NMR (400 MHz, $CDCl_3$)

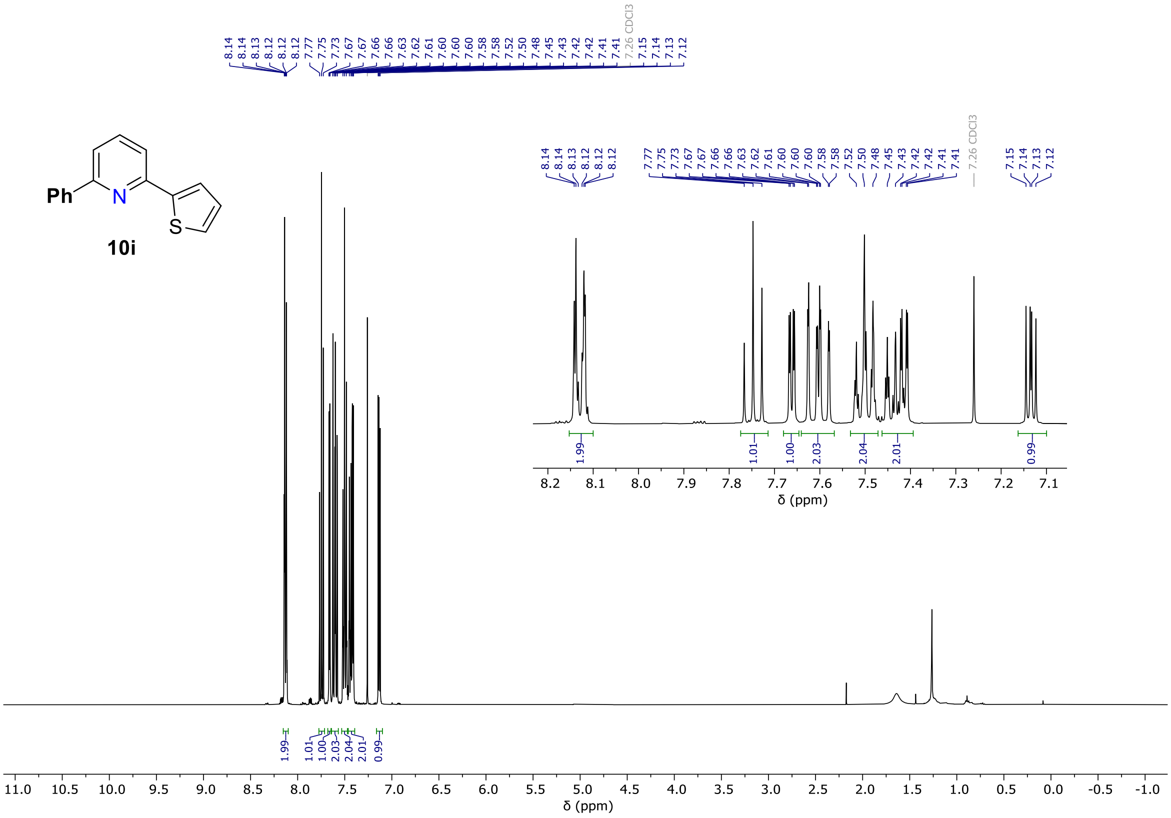

$^{13}C\{^{1}H\}$ NMR (101 MHz, $CDCl_3$)

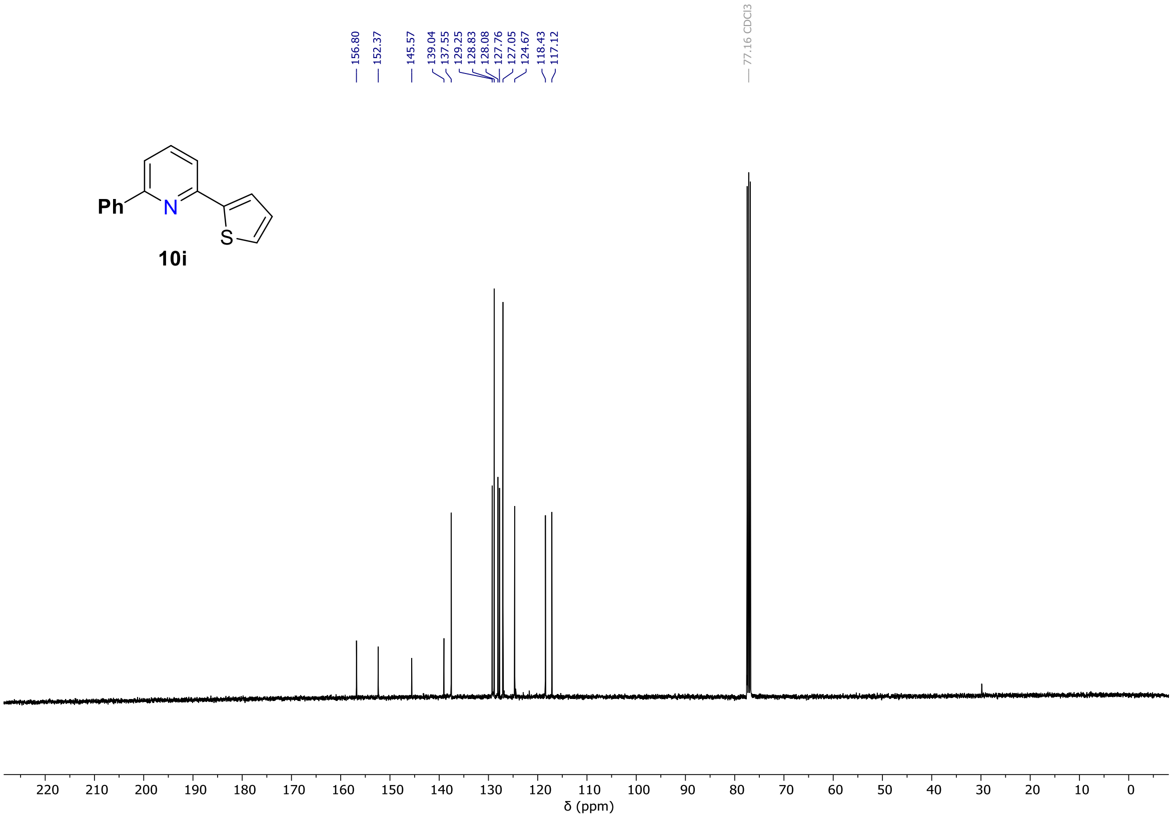

Compound **10j**

^{1}H NMR (400 MHz, $CDCl_3$)

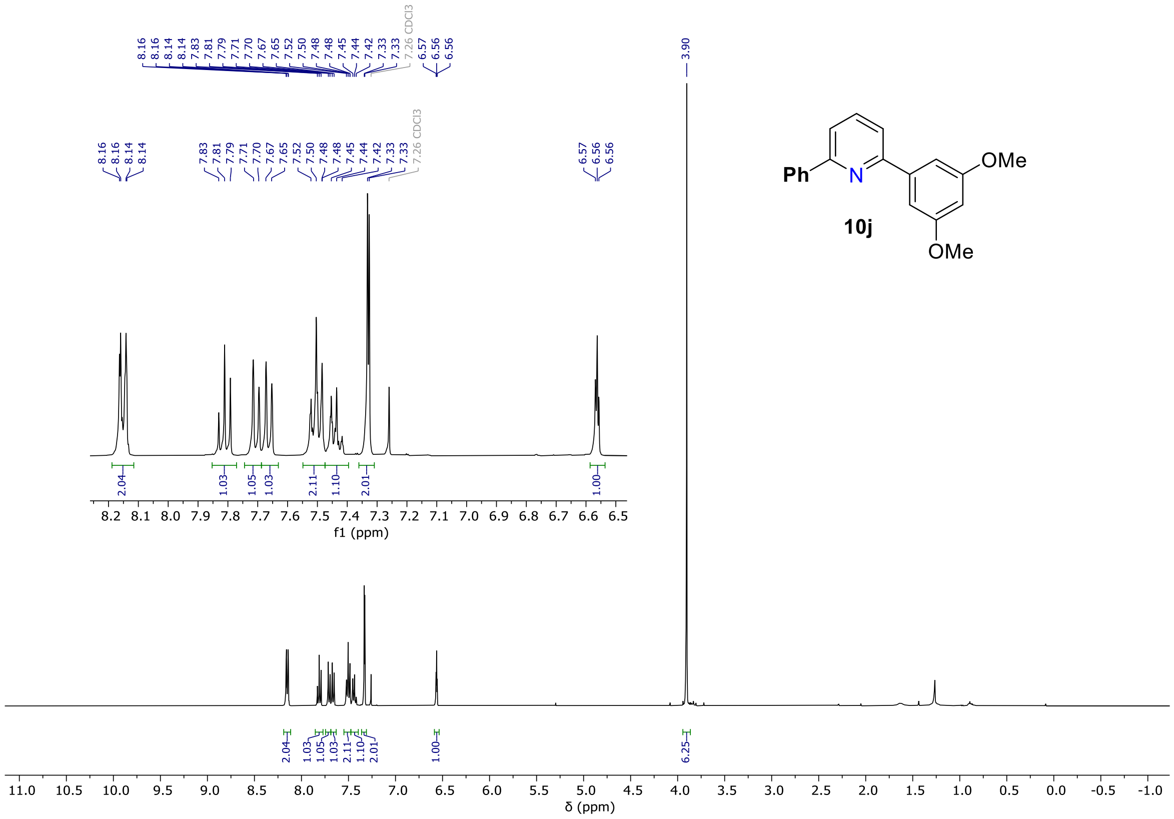

$^{13}C\{^{1}H\}$ NMR (101 MHz, $CDCl_3$)

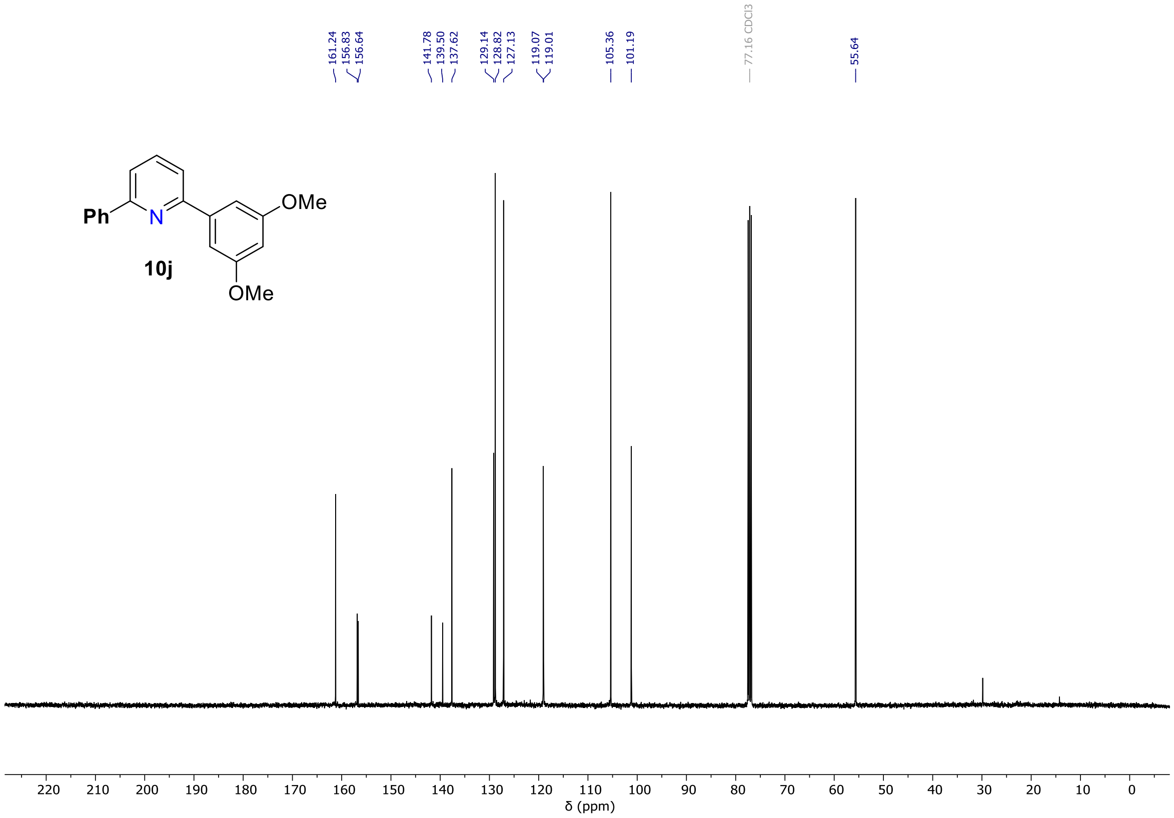

Compound **10k**

^{1}H NMR (400 MHz, $CDCl_3$)

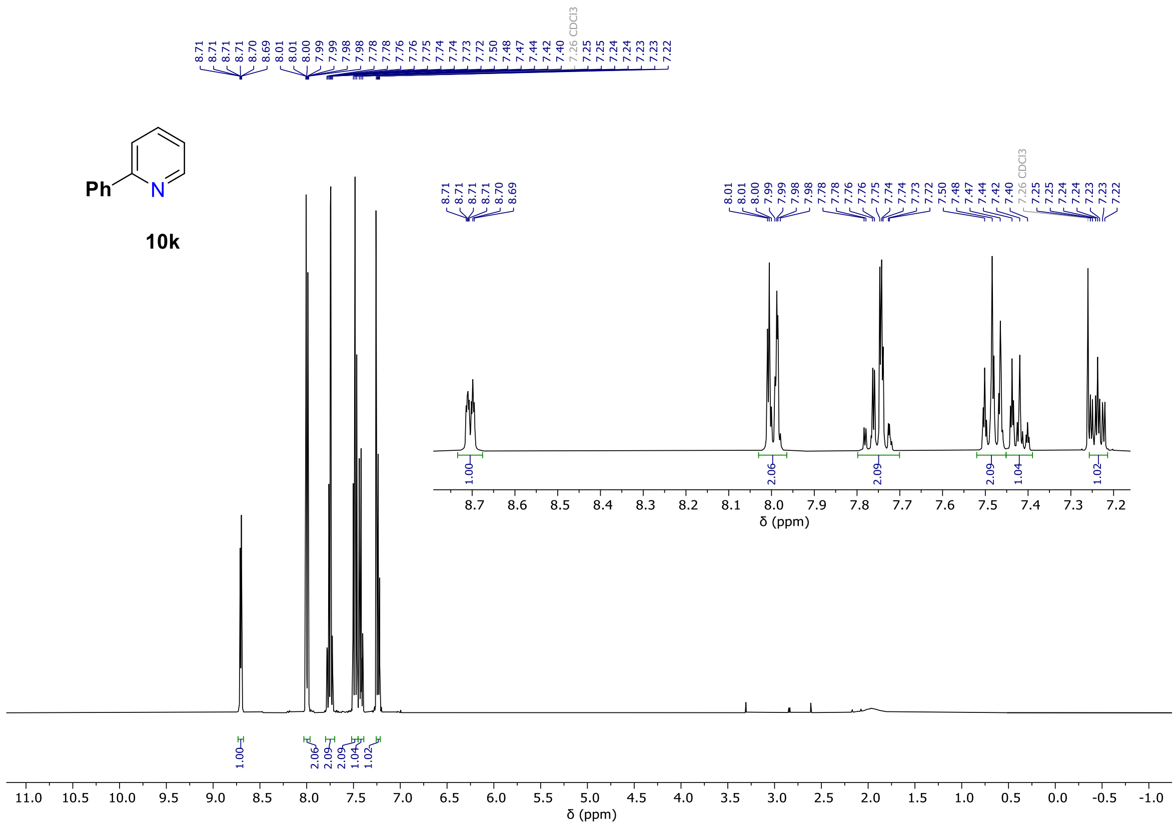

^{13}C{^{1}H} NMR (101 MHz, $CDCl_3$)

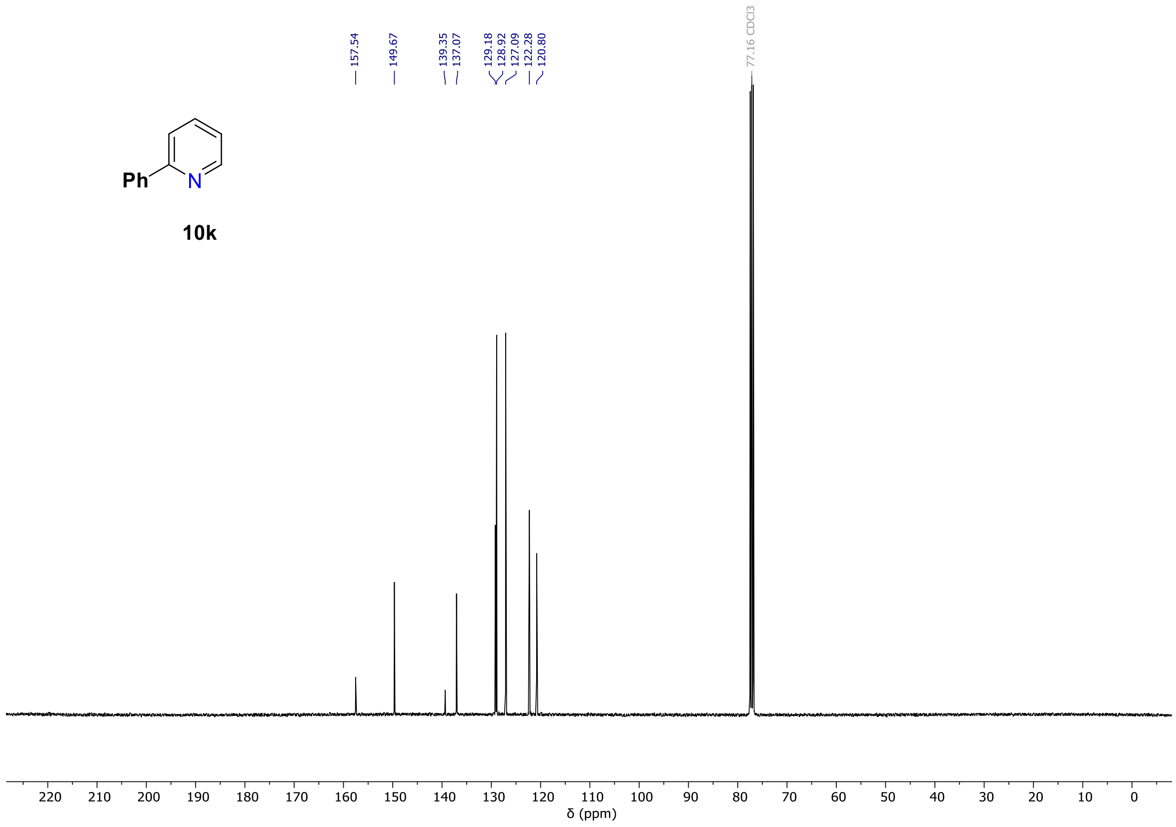

Compound **11a**

^{1}H NMR (400 MHz, $CDCl_3$)

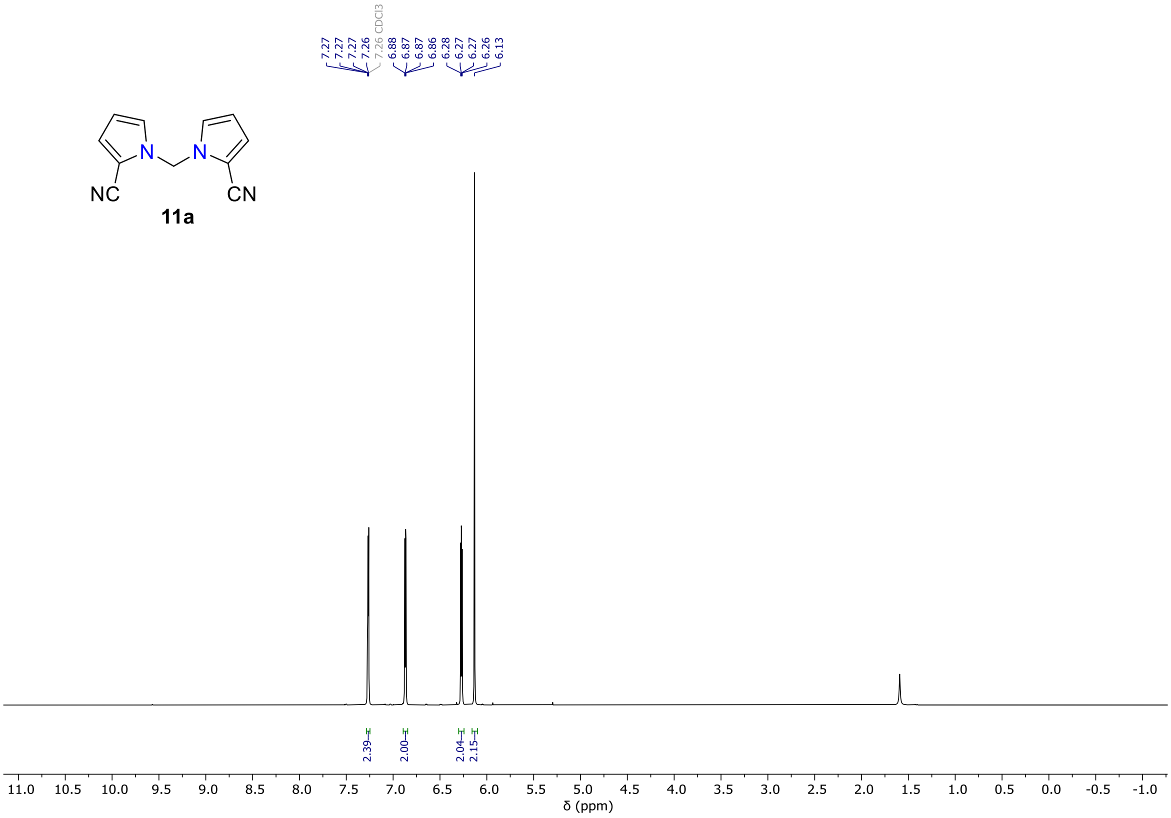

^{13}C{^{1}H} NMR (101 MHz, $CDCl_3$)

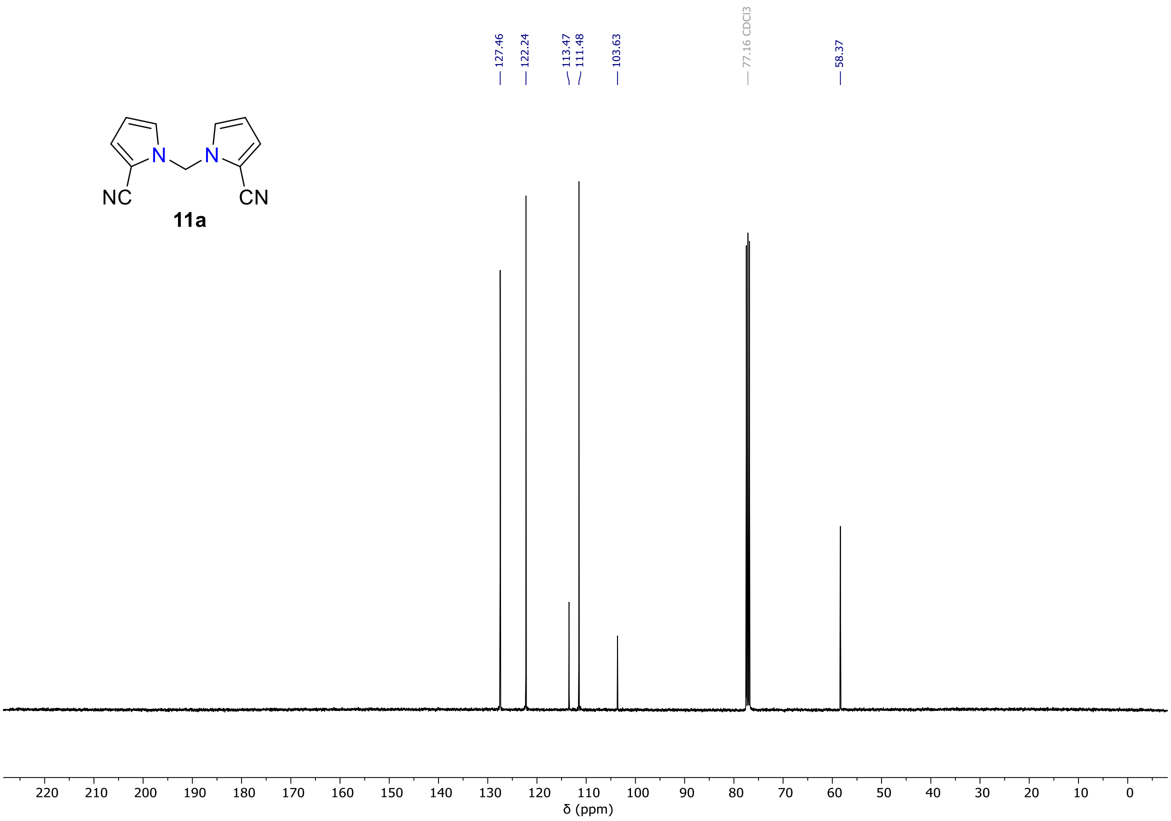

Compound **11b**

^{1}H NMR (400 MHz, $CDCl_3$)

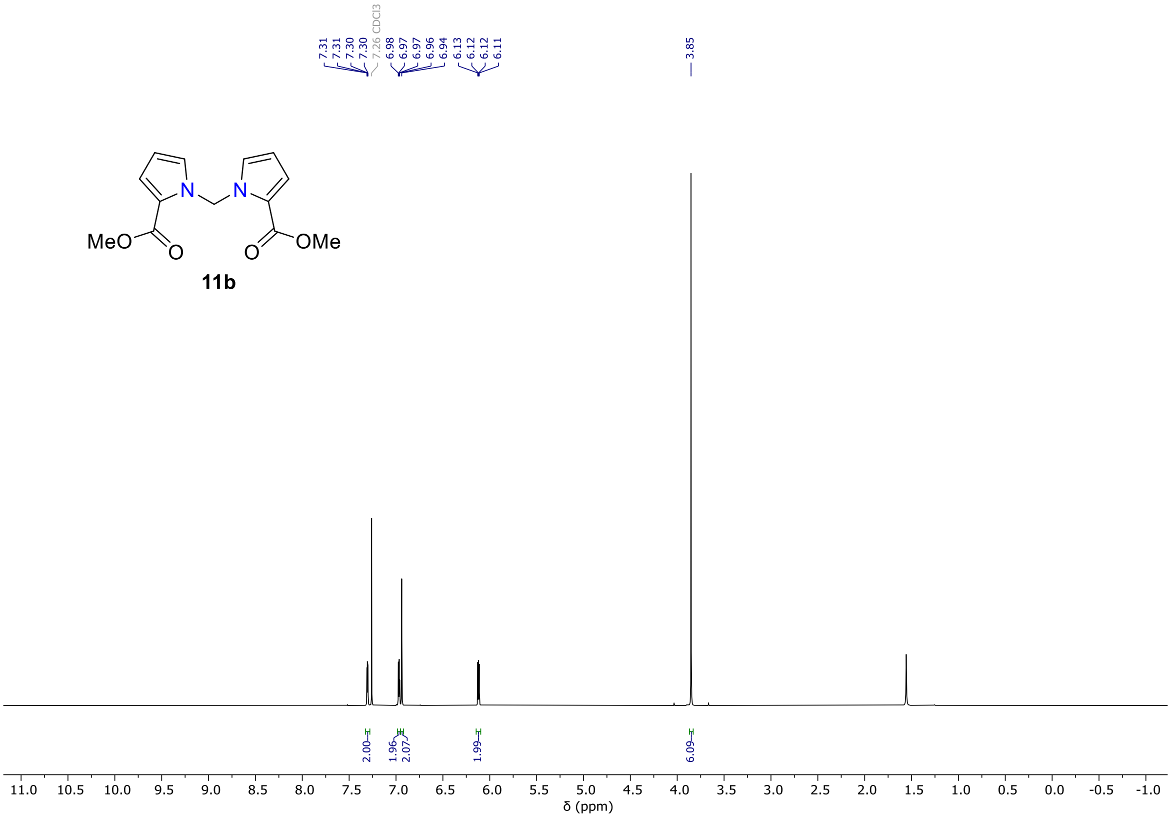

^{13}C{^{1}H} NMR (101 MHz, $CDCl_3$)

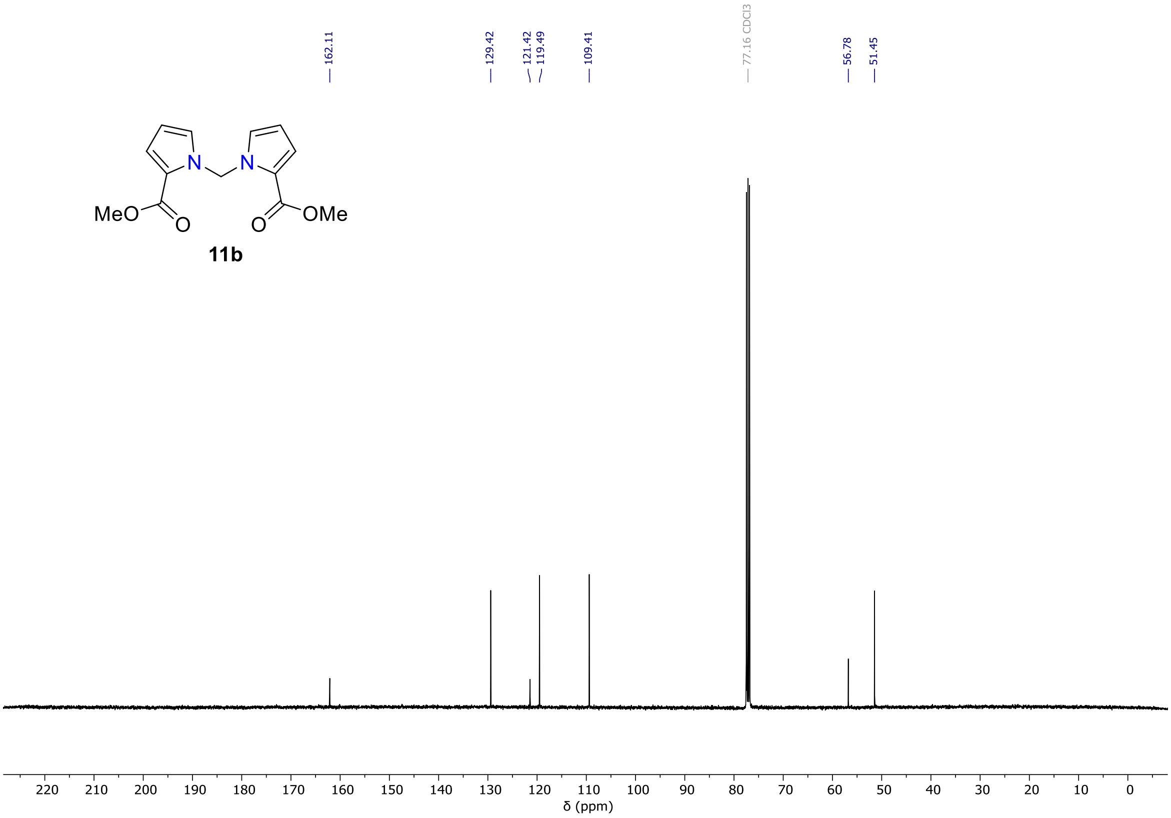

Compound **11c**

^{1}H NMR (400 MHz, $CDCl_3$)

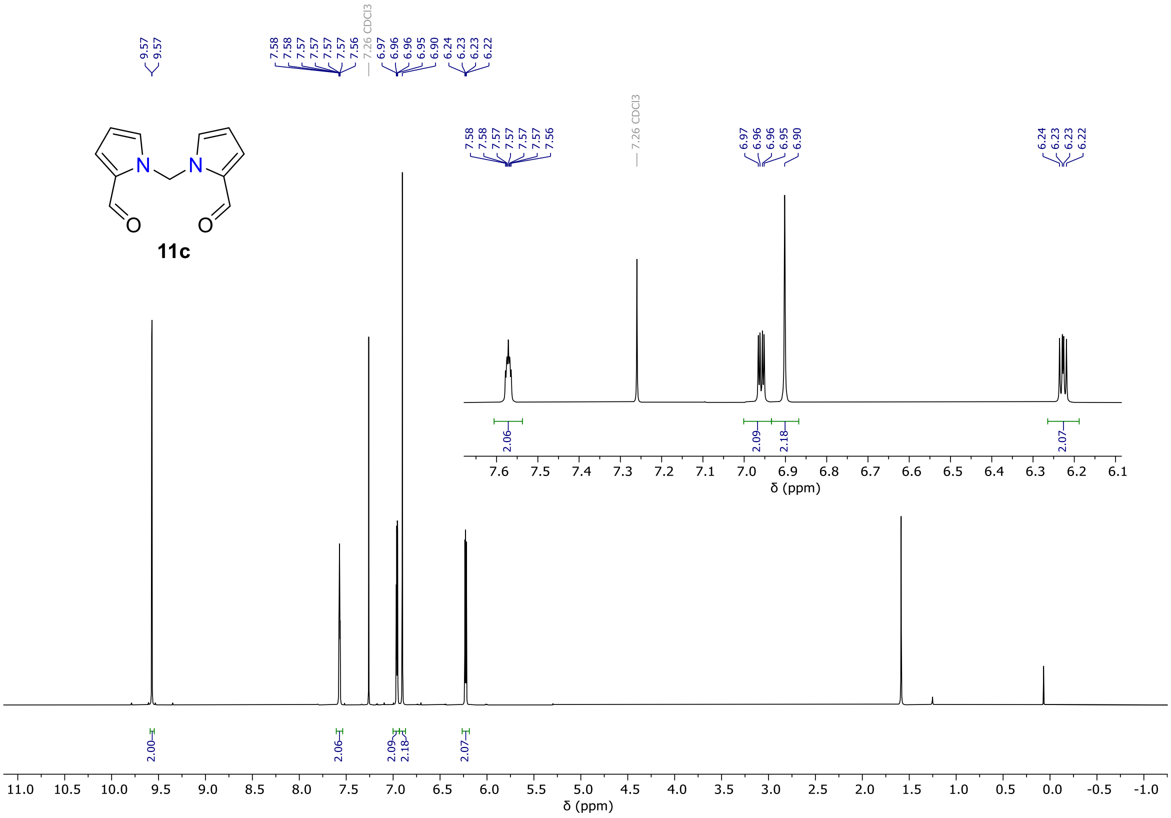

^{13}C{^{1}H} NMR (101 MHz, $CDCl_3$)

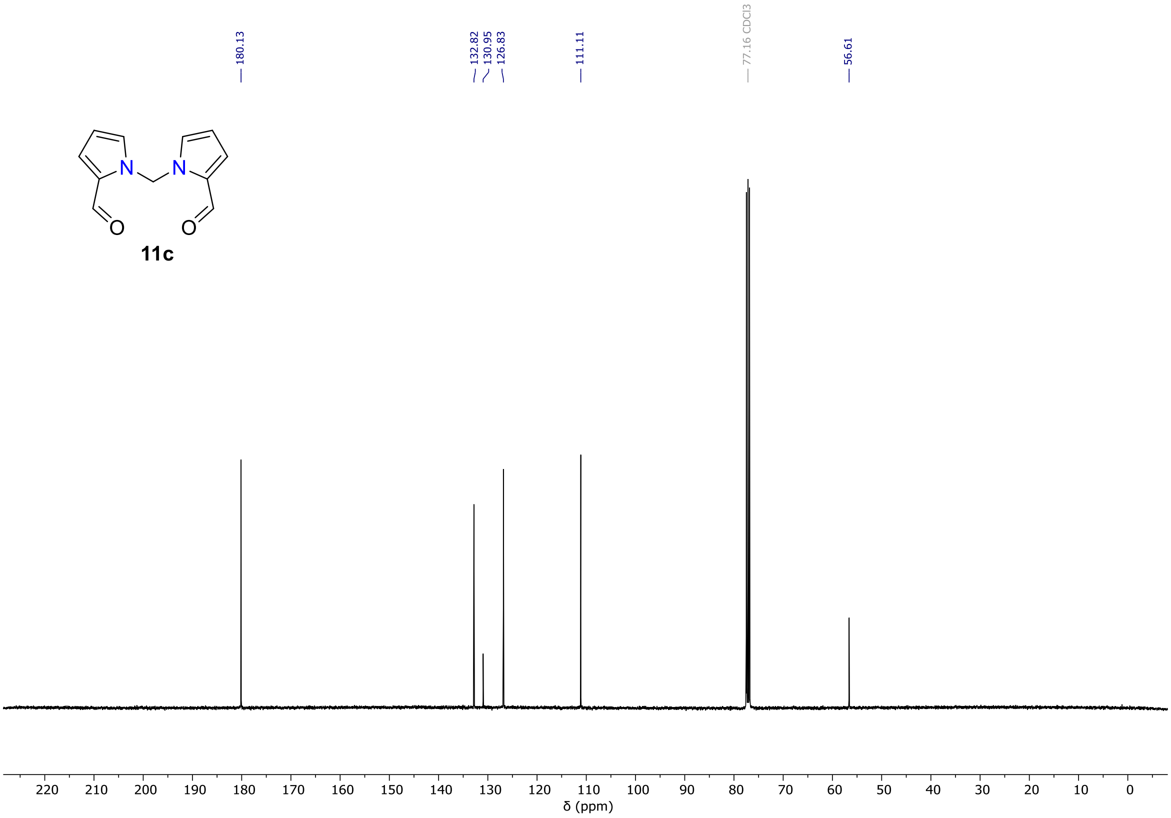

DFT SUPPLEMENT

*-Analysis of **1**:*

Geometry optimizations were carried out with Gaussian 16 Rev. A.03[59] at B3LYP-D3/def2-TZVP level[60]–[66] of theory. Local minima were additionally confirmed by frequency calculation jobs, yielding no imaginary frequencies. Output files were analyzed with GaussView 6.0.19. IBO orbitals were calculated at default (PBE/def2TZVP/univ-JFIT) settings in IBOview v20211019.[67],[68] Wiberg bond indices were obtained by analysing the output files in multiWFN version 3.6.[69]

Two conformers, *transoid* and *cisoid*, were calculated and analysed, because both were previously observed in single crystal structures.[9] The difference between these conformers is barely noteworthy in the context of this work and are shown here only for completeness.

	**Transoid**	**Cisoid**
	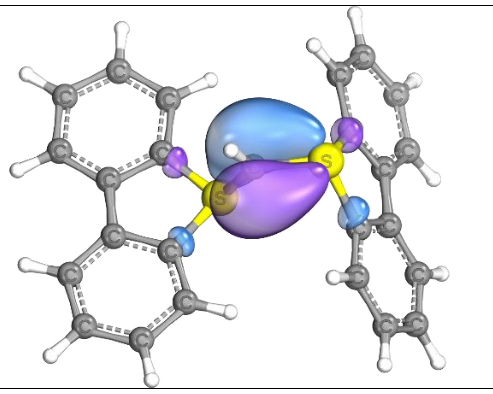	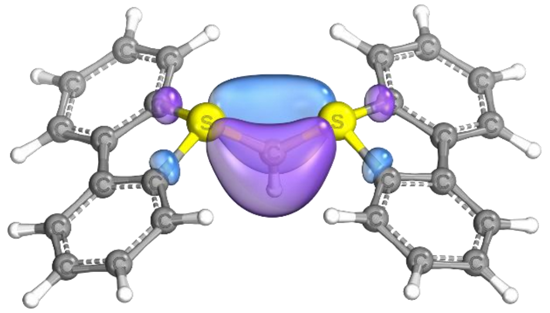
Electronic energy /hartree	-1759.532331	-1759.530815
	IBO electron count	
C	1.620 e	1.578 e
S1	0.099 e	0.105 e
S2	0.086 e	0.105 e
	NBO charge	
C	-0.983	-0.945
S1	0.951	0.957
S2	0.985	0.957
	Wiberg bond index	
C-S1/2	1.56 / 1.63	1.61 / 1.61
	Mayer bond index	
	1.22 / 1.26	1.30

Figure S3: Theoretical analysis of **1**

XYZ Structures

Coordinates cisoid **1**

S -0.79027200 -0.00001900 0.00751700

S 1.60710600 -0.00003000 1.66347400

C -2.03956200 1.27408000 -0.08764800

C	-1.79002600	2.63154800	-0.04579400
H	-0.78615500	3.01259800	0.08393900
C	-2.87715300	3.48998100	-0.17200200
H	-2.72280500	4.55958900	-0.13738900
C	-4.16185700	2.97828400	-0.34863900
H	-4.99582100	3.65939900	-0.45028300
C	-4.38996000	1.60816500	-0.39682900
H	-5.39306800	1.22870500	-0.53651500
C	-3.31695400	0.73235500	-0.26026200
C	-3.31698100	-0.73230000	-0.26025200
C	-4.39002000	-1.60807300	-0.39680700
H	-5.39311400	-1.22857700	-0.53649800
C	-4.16196800	-2.97820000	-0.34859800
H	-4.99595800	-3.65928500	-0.45023300
C	-2.87728400	-3.48994200	-0.17195300
H	-2.72297500	-4.55955500	-0.13732400
C	-1.79012500	-2.63154800	-0.04575600
H	-0.78626800	-3.01263300	0.08398400
C	-2.03961000	-1.27407100	-0.08763000
C	2.32714500	-1.27180900	0.62900800
C	3.11131600	-0.73259100	-0.39458200
C	3.11132100	0.73257000	-0.39456600
C	3.75791100	1.60816700	-1.26203800
H	4.37764100	1.22910700	-2.06332200
C	3.59890300	2.97833800	-1.09103200
H	4.09927500	3.65960600	-1.76575800
C	2.80865900	3.48958800	-0.06242300
H	2.70388800	4.55923600	0.05664700
C	2.16467100	2.63013000	0.82195600
H	1.55868300	3.01190000	1.63307600

C	2.32715300	1.27177000	0.62903600
C	3.75790100	-1.60817300	-1.26207400
H	4.37763300	-1.22909800	-2.06334900
C	3.59888400	-2.97834700	-1.09109900
H	4.09925100	-3.65960300	-1.76584000
C	2.80863500	-3.48961400	-0.06250300
H	2.70385700	-4.55926500	0.05654300
C	2.16465300	-2.63017300	0.82189600
H	1.55866100	-3.01195700	1.63300700
C	-0.07077100	-0.00003100	1.53619600
H	-0.64136500	-0.00001400	2.44984200

Coordinates transoid **1**

S	-1.42371200	0.00003700	0.89942400
S	1.42371200	-0.00008200	0.89942300
C	-2.53014300	-1.27280000	0.30124100
C	-2.29697900	-2.63063600	0.39237900
H	-1.37882800	-3.01503100	0.81561000
C	-3.28177300	-3.48941800	-0.08429000
H	-3.13095800	-4.55894100	-0.03528500
C	-4.46340800	-2.97713900	-0.61759100
H	-5.22263600	-3.65774500	-0.97828500
C	-4.68287300	-1.60686900	-0.69378300
H	-5.60609200	-1.22667100	-1.10950800
C	-3.70289900	-0.73233800	-0.23384600
C	-3.70285100	0.73243000	-0.23392700
C	-4.68277000	1.60697200	-0.69396000
H	-5.60601500	1.22678500	-1.10963800
C	-4.46321700	2.97723700	-0.61792700
H	-5.22240100	3.65785100	-0.97869800

C	-3.28154700	3.48950200	-0.08468800
H	-3.13066300	4.55902000	-0.03581200
C	-2.29680800	2.63071200	0.39207700
H	-1.37862800	3.01509700	0.81525400
C	-2.53006100	1.27288000	0.30109900
C	2.53008400	-1.27289400	0.30107300
C	3.70286400	-0.73241300	-0.23394400
C	3.70288600	0.73235400	-0.23383600
C	4.68284500	1.60691100	-0.69375600
H	5.60607000	1.22673600	-1.10948900
C	4.46335700	2.97717600	-0.61753800
H	5.22257400	3.65780200	-0.97821900
C	3.28171400	3.48942500	-0.08422500
H	3.13088200	4.55894400	-0.03519800
C	2.29693500	2.63061700	0.39242800
H	1.37877900	3.01498900	0.81566900
C	2.53012100	1.27278600	0.30126200
C	4.68279700	-1.60693000	-0.69399600
H	5.60603500	-1.22671900	-1.10966900
C	4.46326700	-2.97720000	-0.61799000
H	5.22246200	-3.65779400	-0.97877500
C	3.28160500	-3.48949500	-0.08476000
H	3.13074000	-4.55901700	-0.03590500
C	2.29685200	-2.63073100	0.39202300
H	1.37867800	-3.01513900	0.81519200
C	0.00000000	-0.00003900	0.00142100
H	-0.00000100	-0.00002400	-1.07846000

Calculated reaction pathways:

-General comments on computational details

Initial structures were optimized at the GFN2-xTB level of theory.[70] The resulting structures were manually altered to produce the ensuing intermediates along the pathway, from the coordination of compound **1** to the Rh-catalyst up to the formation of the cyclopropane ring. The latter step is concurrent with the loss of coordination to the Rh center. The intermediates and transition states for this pathway were obtained with the use of the composite electronic structure method r2SCAN-3c[71] and the Orca 6.0.1 software package.[72] The nature of the transition states were confirmed by the existence of an imaginary frequency. It should be noted that the potential energy surface around both TS2 and TS3 are extremely flat. This comes about through the dispersion interactions between the substrate and the DBT ring systems as well as with the catalyst ligands, leading to rather low imaginary frequencies for the aforementioned transition states. This also shows in the relative free energy between **TS2** and the **B**+pyrrole. The **B** structure is slightly higher in energy. This could also be linked to the combination of different levels of theory. The reported **B** structure was the most stable minimum found with r2SCAN-3c. A constrained search along the imaginary frequency of **TS2** or the C-C distance for the bond being formed consistently lead to the generation of **C** structures.

Zero-point and thermal corrections were obtained with the standard harmonic approximation to the vibrational degrees of freedom. In all cases the temperature used was 298.15 K and the vibrational entropy was computed following the quasi-harmonic approximation suggested by Grimme[73] with a threshold of 100 cm^{-1}. A standard state correction (from gas to solution phase) was applied to the free energies of each system. This correction only impacts the energy going from **TS3** to **D**, and in the reactant energy. Otherwise, all of the reaction steps were modeled keeping the molarity fixed.

The electronic energy and continuum solvent corrections were computed using Gaussian 16, Rev. A03[59], with the B3LYP functional[66], including the D3 correction proposed by Grimme and coworkers[61] and Becke-Johnson damping[62]. The Karlsruhe def2-TZVP basis set[65] and associated Stuttgart/Dresden pseudopotentials for Rh were used. The SMD solvation model[74] was applied for dichloromethane as solvent. In the case of intermediates **A** and **B** the structures were reoptimized at the B3LYP-D3(BJ)/def2-SVP level but only used to report bond distances (Figure 5B and 5C) as well as the bond orders reported in the main manuscript.

$NICS(1)_{zz}$ were computed on the B3LYP-D3BJ/def2-TZVPPD level of theory.[75]

- Comparison between r2SCAN-3c and B3LYP-D3(BJ)/def2-TZVP optimized reaction steps

Given the smaller molecular sizes that need to be considered in the final computed step of the naphthalene pathway, the elementary step was computed at the SMD/B3LYP-D3(BJ)/def2-TZVP//B3LYP-D3(BJ)/def2-TZVP level of theory. Below we compare the results to the composite method SMD/B3LYP-D3(BJ)/def2-TZVP//r2SCAN-3c we applied for the remainder of the mechanisms, the only difference being how the geometries were optimized. The resulting free energies are compared in the Table below. The results show that the level of theory used for the optimizations has a minimal impact on the obtained energies.

Table 1: Relative free energies (ΔG) in kcal/mol for the final reaction step in the naphthalene pathway. The procedure is the same as described in the Computational Details, with the only difference being in the level of theory used for the optimization. The electronic energy is in both cases refined at the SMD/B3LYP-D3(BJ)/def2-TZVP level of theory.

**Stationary point/ optimization level**	**B3LYP-D3(BJ)/def2-TZVP**	**r2SCAN-3c**
D_exo	0.0	0.0
D_endo	1.4	1.1
TS4_exo	32.9	32.9
TS4_endo	13.2	12.9
protonated naphthalene + DBT	-32.1	-32.4

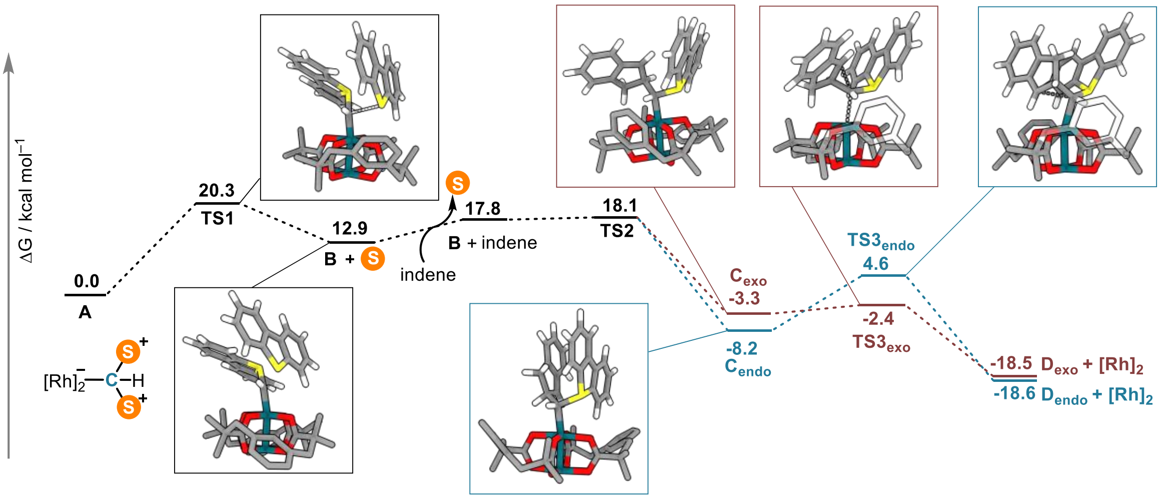

Figure S4: From indenes to naphthalenes

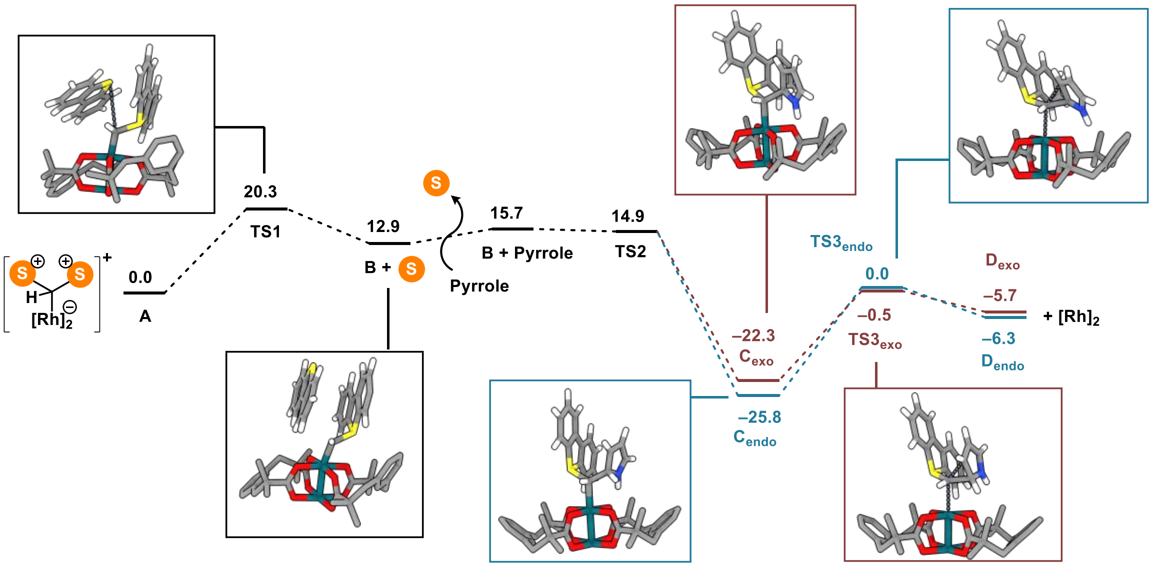

Figure S5: From pyrroles to pyridines.

XYZ Structures (shared between naphthalene and pyridine pathways)

21

DBT r2SCAN-3c

S	-1.76481846048611	2.10200193889503	0.25050488294239
C	0.10384998634007	3.62437843299254	-1.20176639500512
C	-1.17708265772589	3.48029151815016	-0.67528395363294
C	-3.34945611999933	2.85271238574533	0.41713406322303

C	-4.44426046459681	2.31080785334312	1.08531262538950
C	0.39986273847351	4.78135973881192	-1.90926551280342
C	-2.16477095443596	4.47329007541166	-0.84478616960419
C	-3.41591490324418	4.11177921561560	-0.21582339622622
C	-5.62377417278002	3.04189673377590	1.11967857689502
C	-0.56644885916270	5.77846426185142	-2.08833837080596
C	-1.84006803633246	5.62987842105075	-1.56197335986361
C	-4.61691096368797	4.82785744700949	-0.16644430647906
C	-5.70990957560312	4.29314715330052	0.49751027319325
H	0.85338634818534	2.85115542683560	-1.06304354793531
H	-4.37845584543379	1.34048314672553	1.56803079129454
H	1.39347381383606	4.91177254933861	-2.32735645342586
H	-6.48833703412346	2.63574364110748	1.63606224734244
H	-0.31506767247226	6.67626784415720	-2.64463972015433
H	-2.58397664032563	6.40856171879796	-1.70567264132444
H	-4.69129542248376	5.79909732788423	-0.64741824986480
H	-6.64174510394153	4.84899316919993	0.53576861684511

82

Rh2 r2scan-3c

C	5.51281273093108	-3.34758911834661	-0.53460649746521
C	5.62786109673396	-2.22165898980167	-1.34353004789522
C	5.27691258577307	-0.96177124906896	-0.85366489873061
C	5.03066459440132	-3.23231993931699	0.76512308949844
C	4.67362515419285	-1.98298524763062	1.27747327733367
C	4.82539041466740	-0.85998228702800	0.46314481520701
H	5.80139275474386	-4.32168416964957	-0.91964612534913
H	6.00026445277765	-2.31960313135384	-2.36058227805431
H	4.57905517516582	0.11926160765703	0.86279266003414
H	4.93773846941269	-4.11735972981451	1.39023148342936
C	5.33840287443231	0.26865822774029	-1.72126236772351
C	4.01092745486548	0.58363148450047	-2.47191495368345
H	6.11860973514402	0.15503546399090	-2.48466654000140
H	5.59965937072124	1.13837815758290	-1.10889595198556
C	3.67592402639806	-0.51925611688186	-3.47632237081755

C	4.16591913747299	1.92938660280150	-3.20222927167074
H	4.97524177542789	1.85813882948523	-3.93733696142870
H	4.39631687837854	2.73424702679878	-2.49894997220271
H	3.24480285191170	2.18799475888874	-3.73578342316839
H	4.50159739186858	-0.62214755232351	-4.18973120810549
H	2.76646173271607	-0.27798398115618	-4.03313841515804
H	3.52126440491142	-1.48061502315003	-2.98090980999201
C	4.09972636682542	-1.83163264261028	2.66259574933111
C	2.54501254205489	-1.90595091463617	2.71988929477297
H	4.41162156681246	-0.87138385437110	3.08710018332739
H	4.48190789390278	-2.62407016145792	3.31854959186202
C	1.96200525555426	-0.81605126043859	1.81769811389528
C	2.09218009997031	-1.60469336271827	4.15961475454727
C	2.04836507650556	-3.28790776802394	2.29526964780323
H	2.42738116379123	-0.61427457648985	4.47948955285784
H	2.50753317134512	-2.35582464627054	4.84055934391973
H	1.00022726196417	-1.64355555031298	4.23818054438307
H	2.32825166391679	-3.51517259372270	1.26408516621572
H	0.95963062543998	-3.35182484565744	2.37091397755017
H	2.48622989533318	-4.04755695688773	2.95294655671392
O	2.34822195637009	0.37692725228331	2.04290406920954
O	1.12624764407522	-1.16490422261453	0.92832650239988
C	2.88752922370827	0.74593231165841	-1.44606395165282
O	1.84352138920224	0.03896874160309	-1.59272877300199
O	3.07772478072959	1.60287093045271	-0.52291719603036
H	1.00103679277020	3.86553036915148	-3.57770691999937
H	1.34321933615945	5.03004996766162	-2.28091032180056
C	0.56127018683020	4.63744247860858	-2.93668402267868
H	0.19996033004951	5.44667000031587	-3.58099414204575
O	-0.45286245341098	1.77026891631327	-1.42254896530808
O	0.81091244970515	3.31119029811302	-0.35412488690751
C	-0.04772315241442	2.95696641716137	-1.22611609112637
C	-0.61069229376049	4.06316123806049	-2.12156082420891
H	-1.26005488453749	2.74428333254131	-3.72358692275331
H	-0.36857157345868	5.63204946066506	-0.64992418519757

C	-1.67924120848166	3.50910494707545	-3.06441721620335
H	-1.58363065206798	5.96856831532477	-1.89227465855614
C	-1.19316533698100	5.19276202396596	-1.22131164076171
H	-2.07224811037814	4.32355411410163	-3.68360855988077
H	-2.50756899266079	3.05977263336918	-2.51177424613482
H	-3.91408995388406	5.16151579244727	-1.60398263030780
C	-2.27293757186291	4.72990660142929	-0.27796789198773
C	-3.62196058927837	4.75575729942715	-0.63824149424265
H	-0.88413956746868	4.23332695308116	1.27837681841356
C	-1.92750186745000	4.22793696843816	0.97764741412732
C	-4.59207486484596	4.27571567981481	0.23554816201275
H	-3.71598917516180	1.03665548880190	1.71935450094480
H	-5.64040115352854	4.31320872946237	-0.04730926319093
C	-2.88437585263342	3.71248565045359	1.85305432881330
O	-1.17274265376502	0.56970779768275	1.09956068337298
C	-4.22696274974680	3.74862611754775	1.47016697980697
C	-3.32299315157548	0.75788158392707	2.69983002266764
C	-0.98022362886644	1.40255268566277	2.03775091994931
O	0.07830776874162	2.08984172000425	2.21185095507235
H	-3.07871741023742	-0.30738209166096	2.67370059346455
C	-2.09436010440282	1.58912297864147	3.06987814919701
C	-2.45140910557193	3.10223227533028	3.16104930208138
H	-4.10684416669738	0.91575795432849	3.44950188439116
H	-4.99027921359014	3.37037405106513	2.14614981930845
H	-1.57622898970961	3.63729848699295	3.54506252414636
H	-3.25184826616948	3.19754026832239	3.90569185965034
C	-1.54218411263979	1.13164342203996	4.43156368481176
H	-1.27122234571695	0.07063679606286	4.40191011877501
H	-0.65579310138370	1.70753391025901	4.71139156249023
H	-2.30909226876112	1.26413168499789	5.20276725398139
Rh	1.60837088745344	1.88199990945489	0.87275054646073
Rh	0.31008413084026	0.27024226984728	-0.26959956082187

126

A r2SCAN-3c

Rh	-0.18390872044199	-0.47533501042813	0.12719184505416
Rh	-1.46908715787337	-2.50634105168912	0.34135303393332
O	-2.48655300875947	-1.93282269020406	-1.32780755643356
O	-0.09512702508177	-3.46445723337074	-0.84254228212739
O	-0.36646901240093	-3.02177426526580	1.98501439810145
O	-2.75653597940668	-1.45963629079400	1.51641208438205
O	-1.33591344191730	0.01038116215512	-1.51669253195155
O	-1.54722734108809	0.45247027453047	1.35783757726474
O	0.83658536740956	-1.11730942528573	1.79628715853626
O	1.03154054723729	-1.53751275741793	-1.16291534325007
C	6.12350633638100	3.54905284723979	-0.00035449519771
C	5.42212717254132	3.46071760602581	-1.19810114589192
C	3.48103527552978	2.90511089848536	-3.69495979412797
C	2.52114127386146	2.52912258819543	-4.62839858300265
C	5.82870159968394	2.70665213076910	1.07391907520627
C	4.40080041952050	2.51955947850649	-1.32483220118324
C	3.52359901036960	2.25957994849326	-2.45949539952858
C	1.59339108831871	1.52616591909777	-4.34316962985317
C	4.82141222832864	1.75125767886230	0.96606699700378
C	4.12215493187170	1.68981317464168	-0.22883022616960
C	2.58961544004379	1.24080595122024	-2.21041189384300
C	1.61466552689585	0.86168895508325	-3.11795899178547
C	-6.77385513602263	-0.14477439514212	0.38003691247156
C	-7.20131069702460	-0.11955616371234	-0.94034418750114
C	-5.44550163844898	0.14587661143303	0.71208517715690
C	-6.30536185713671	0.18013657592004	-1.96306523919790
C	-4.57434585985196	0.48430218064776	-0.32155831136999
C	-4.97939147684503	0.48024148937750	-1.66010481638222
C	4.77168700154776	-2.88154487349621	-0.55634304965274
C	5.38912690348837	-2.09734646114780	0.41470293334376
C	3.53584342028768	-3.47329986433366	-0.29244878732607
C	4.77411515709822	-1.88979691500048	1.64416134980483
C	2.94174321148462	-3.26398139498699	0.95453053557431
C	3.53208366036908	-2.46695152933449	1.93302169875961
C	2.81609736389872	-4.31251404813535	-1.31527276787544

C	2.89727166573429	-2.17774316698891	3.27897515425161
C	-5.02708225028791	-0.00459541021027	2.16103800916484
C	-3.96933157186628	0.75628953502920	-2.74198889569692
C	-3.89661899180127	-1.63403743410575	-3.59781465233415
C	-2.09592314214620	0.00293161794242	-4.23134141820272
C	-3.36907224847100	0.03334009989721	4.03738544512660
C	-3.51526100516749	1.99862953995136	2.48993193980227
C	1.36391084215705	-4.13720960238029	3.81814277095079
C	0.88140143213406	-1.86085782563059	4.73341089356244
C	1.08436223688179	-4.54183523545182	-3.12187600550455
C	2.61460338870769	-2.55875969839358	-3.14127205021289
C	1.83882676152687	-3.52843543000365	-2.24858303861991
C	-3.05626072045211	-0.44996251406963	-3.12004411425207
C	-3.62404030509059	0.47627320335336	2.58119337116615
C	1.44310594846486	-2.63513431794707	3.52123192016125
C	0.56441958448771	-2.24268530770006	2.33384724973933
C	0.83915054605781	-2.78844989270302	-1.35586839011658
C	-2.22845657765093	-0.83221555975808	-1.88964313692906
C	-2.55766205144604	-0.23092839950943	1.74164753230598
H	6.91862605389014	4.28124292620787	0.09870351919169
H	5.67279271672561	4.11753413952608	-2.02551529085985
H	4.18859243093829	3.69538436088403	-3.92591569106875
H	2.49137527236560	3.02752680526945	-5.59236700714188
H	6.39722407357721	2.78600577091810	1.99505480024345
H	0.84684526891986	1.25128847834265	-5.08216443332229
H	4.59956415147199	1.06789764486638	1.78038603403240
H	0.89546000815269	0.08787179272518	-2.87305339390217
H	-7.47694584360042	-0.40482346402887	1.16845354421427
H	-8.23706382226453	-0.34571029772964	-1.17632766823999
H	-6.63802231231840	0.17855286313761	-2.99826525866182
H	-3.54314525888574	0.75316926852110	-0.11413882203671
H	5.26253424770323	-3.05085599743188	-1.51161549071924
H	6.36232342727469	-1.65634445448295	0.21599661888504
H	5.27714510462284	-1.29545859718886	2.40548934446449
H	1.98626169494828	-3.74589934698392	1.13923644057744

H	2.23796018666872	-5.09176553194385	-0.80574497751309
H	3.53995931528081	-4.81518895490233	-1.96866117612902
H	2.95157767543151	-1.09303533751384	3.43950246253978
H	3.51437178667061	-2.62921231107745	4.06895875829059
H	-5.75291352771121	0.52022970876847	2.79751106094535
H	-5.11382704262789	-1.06887393668593	2.41389790043847
H	-3.31646590758149	1.57816908323436	-2.43089478235336
H	-4.48254117677530	1.06723908567158	-3.66121402985678
H	-3.26096984405582	-2.47701094612843	-3.88461077377435
H	-4.48574921047741	-1.33235107048154	-4.47106817313321
H	-4.58077046710079	-1.97634151895240	-2.81923548221617
H	-1.52268467873745	0.88111298688268	-3.91783696376853
H	-2.66401445950529	0.25612005388501	-5.13268931591810
H	-1.39312476202880	-0.79840977063864	-4.48947980398719
H	-2.37935936398129	0.36255019143726	4.37292112698118
H	-3.42673027082442	-1.05502779882250	4.13014490582496
H	-4.12027885267783	0.48049769549799	4.69743364770834
H	-2.51678723999116	2.33691796367425	2.77749163452406
H	-4.24690440475979	2.44932011519793	3.16991032904743
H	-3.72522314040704	2.35697240258152	1.47916000719245
H	0.32766595279246	-4.45477689412602	3.95222123670681
H	1.91291073958075	-4.34683243500865	4.74209285042903
H	1.80389793268071	-4.74484926648205	3.02359456611655
H	-0.13946608994434	-2.18671426606995	4.95669425510642
H	0.87152287241129	-0.78299714815378	4.54112516929420
H	1.50254935418628	-2.05360727580197	5.61493890842520
H	0.37446387723232	-4.03142253098399	-3.78150557392805
H	0.52821247195994	-5.25739268724073	-2.51284932349149
H	1.79687949914332	-5.08929598886930	-3.74773516460683
H	1.93737339148458	-2.01560989605231	-3.80806390973303
H	3.31558404937613	-3.12378483415160	-3.76517546256956
H	3.18483984209304	-1.83003272875575	-2.56053961001763
H	-3.91794569985052	4.82881701280579	-1.36285091830519
C	-2.94052811446471	4.42522035266112	-1.11812897467318
H	-2.55983633201161	3.76350060924814	-3.13124466155880

C	-2.17734022204941	3.81928644336652	-2.11725381483744
C	-2.46184033151315	4.53471461870120	0.18226705619628
H	-3.05749740134597	5.02715458825033	0.94466898582619
C	-1.20408181864954	4.02215163491459	0.49456749419802
C	-0.47762613060062	4.09642285252199	1.75483786058171
H	-1.88421286642027	5.04677048774119	3.08479253386061
C	-0.88916194867542	4.62605402396476	2.97725127551416
C	-0.01895688831391	4.60240770627719	4.06144976871737
H	-0.34243120342128	5.01070552524461	5.01362666544918
C	1.26520990589887	4.06761486998671	3.94459366585526
H	1.93211403280668	4.06693364255503	4.80054919577943
C	-0.47573875210224	3.39317240774509	-0.52501895852454
C	-0.92255715247236	3.29004976060577	-1.82900206238219
H	-0.32228659209375	2.81045376089091	-2.59512996069537
C	1.70461526301092	3.54434952855530	2.73144658020058
H	2.70847723198783	3.14361634913283	2.62348809369623
C	0.81741960824373	3.56897633160170	1.66899489390225
S	2.81710863302956	0.52084493693054	-0.58498055018723
C	1.25288456722382	1.06756887683051	0.29660402191855
S	1.16520430368732	2.96316494820721	0.02850655785729
H	1.54735106112915	1.02078137845973	1.35825558207624

126

TS1 r2SCAN-3c

Rh	-0.22829394082668	-0.66086789520444	0.30235841925829
Rh	-1.47650907668939	-2.71557023563105	0.45765061785634
O	-2.51695269997339	-2.10023981795374	-1.17644606905466
O	-0.12954486948192	-3.62962405637711	-0.77171784435421
O	-0.39744283757492	-3.34076965525607	2.05881171863358
O	-2.77362446203224	-1.75534390694700	1.67930469226327
O	-1.40042037329093	-0.14230860556114	-1.28562910207101
O	-1.60770041257301	0.18329003492240	1.56984786134799
O	0.74621925482933	-1.39120831729150	1.98295846019339
O	0.96658616315310	-1.67846692043499	-1.04915067691670
C	5.93089986161238	3.50243084661022	0.31056872715977

C	5.24548352437724	3.43104963329873	-0.89736322024194
C	3.38748501456995	2.89631210625175	-3.45565283680976
C	2.44100324983189	2.54512552772161	-4.41228905352515
C	5.65785400593792	2.60974996044566	1.34853150848742
C	4.25635463383441	2.46246971372048	-1.07118408490388
C	3.41180231502216	2.21917888527431	-2.23575588476010
C	1.50574255400229	1.53961129041736	-4.16716662124842
C	4.67085410241153	1.63755381057165	1.20273073047661
C	3.97768868991044	1.60721529446553	0.00520221153902
C	2.47694256216122	1.19647930297807	-2.03984432131903
C	1.51270789189005	0.84274925730540	-2.96169148161008
C	-6.83387889363307	-0.40347002849318	0.61195568990059
C	-7.26624699568663	-0.31975368043643	-0.70430039058612
C	-5.50127923008346	-0.13798765652856	0.94778034389555
C	-6.37286150438406	0.01583137061987	-1.71869815347885
C	-4.63497056282475	0.24368747311962	-0.07465871725129
C	-5.04472949902572	0.30251189730989	-1.41035581234327
C	4.69077599801315	-2.96094721113863	-0.50728471878675
C	5.29170420596811	-2.19647462087200	0.48949143183621
C	3.47995704826844	-3.60631571675852	-0.25532844037740
C	4.68520295151606	-2.06264201374737	1.73297959701416
C	2.89540181056030	-3.47409791885525	1.00703921140391
C	3.46977302042897	-2.69852817332666	2.01212512553119
C	2.77602570586901	-4.42135413799910	-1.30760643810907
C	2.84868957521862	-2.49651731401202	3.37988614411655
C	-5.07426199118952	-0.35135938858966	2.38628978410107
C	-4.03977383155251	0.63746189827043	-2.48308762056570
C	-3.94934141038691	-1.72089721325134	-3.43165520023415
C	-2.15811497356223	-0.04593120272427	-4.00540469529020
C	-3.40198932651363	-0.37540131884987	4.25684091907301
C	-3.58354350285942	1.65285920681830	2.79820264822983
C	1.33691722087247	-4.49721095207997	3.84156906540824
C	0.83723637769717	-2.26652349486655	4.86134965237115
C	1.02394250579049	-4.62273370345350	-3.09879886879601
C	2.52672588236239	-2.61446665386985	-3.08151923329254

C	1.77889656339532	-3.62320827179577	-2.20954358901782
C	-3.11630276782337	-0.54740888451323	-2.91145812599286
C	-3.67287526348409	0.12645935323217	2.82093074814939
C	1.40135760013674	-2.98226865115375	3.61459830379251
C	0.50879032035472	-2.55444607972258	2.45358531290768
C	0.78714749533184	-2.92643228407935	-1.27990188063440
C	-2.28682221188895	-0.97579657094910	-1.70215927263855
C	-2.60332905637326	-0.52982306782450	1.94690826214321
H	6.69560536083177	4.26177804634847	0.44564713645643
H	5.47602281938316	4.13152789704015	-1.69454240741630
H	4.09028560524266	3.69997791537772	-3.65372906719693
H	2.42382861289152	3.06935975661829	-5.36312107490150
H	6.22067876922132	2.66833692890091	2.27466262203153
H	0.76342914868717	1.29066290919467	-4.91878377455481
H	4.45542835785234	0.93139864223655	1.99749341352395
H	0.78858687341271	0.06700463937984	-2.74494349026012
H	-7.53739852871916	-0.68334453506867	1.39317442845301
H	-8.30615126703273	-0.52278966052761	-0.94314494668180
H	-6.71314855311483	0.06025042376730	-2.75037853352584
H	-3.60644822080783	0.51627389659191	0.14006960618478
H	5.17516063330736	-3.07131545153428	-1.47427856230484
H	6.24457735535306	-1.71050610019834	0.29831866094313
H	5.17438263327046	-1.47950191510859	2.51129061377151
H	1.96364306818158	-4.00358684062975	1.18331602750950
H	2.21771319747296	-5.23362062269774	-0.82839891304566
H	3.50640632799195	-4.88103350997207	-1.98450721011689
H	2.89861899077036	-1.42424036701489	3.60677147799185
H	3.47674925981016	-2.99201392330557	4.13367901607247
H	-5.79678890431862	0.14511798757837	3.04814881989750
H	-5.15813492532780	-1.42509295230013	2.59787438086645
H	-3.39977173854191	1.46661990375516	-2.15672620576732
H	-4.56314027485992	0.96412357786976	-3.39053176553405
H	-3.31118449018780	-2.54973137684803	-3.75279271523988
H	-4.53675436176717	-1.38701090792391	-4.29381411038826
H	-4.63706085505072	-2.09500577402494	-2.67081321833117

H	-1.59445463234020	0.82799699264366	-3.66540715309900
H	-2.73454866824387	0.23307101586308	-4.89350572552577
H	-1.45368334523434	-0.83426194860622	-4.29816530254743
H	-2.41156520777627	-0.05471073530393	4.59799635810775
H	-3.45897044567917	-1.46643874183337	4.31452135224959
H	-4.14798766203347	0.04786168971506	4.93769422424623
H	-2.59283779854869	1.99041659958732	3.10783319506220
H	-4.32568690654439	2.05843003807436	3.49350673264189
H	-3.79573022124758	2.05637977876110	1.80461696455421
H	0.30518566970972	-4.83081261006310	3.97182081268607
H	1.89615795395351	-4.74084100543900	4.75038828934226
H	1.77585260098950	-5.06426650422466	3.01712282364173
H	-0.17697410699846	-2.61608071173139	5.07895442258593
H	0.81395246541376	-1.18192495232211	4.71679557443797
H	1.46924235979562	-2.49041957080143	5.72696132428828
H	0.29871580452124	-4.10693522707377	-3.73692663667648
H	0.48872892588013	-5.36511948947628	-2.50306350580008
H	1.73784623979459	-5.14109613495762	-3.74693976588055
H	1.83265372213634	-2.06195040337134	-3.72236385070102
H	3.22707648504802	-3.15095317795923	-3.72985789516897
H	3.09254983490697	-1.89461581657541	-2.48607087166313
H	-3.58403481147184	4.26558150327684	-2.16564625896933
C	-2.52752617143804	4.28974820332767	-1.91707285928850
H	-1.90535716675090	4.54403793165289	-3.96598903941231
C	-1.57970857451558	4.46515872262067	-2.93342276347914
C	-2.13300986078690	4.18031473534511	-0.59263267298628
H	-2.87676688847532	4.07423275865855	0.19255873118186
C	-0.77353236761029	4.24149300840535	-0.26921108996764
C	-0.14029315955616	4.14975678989599	1.02672524875641
H	-1.81073983974433	3.98497028905829	2.37835899778058
C	-0.72933396263663	4.00126954236502	2.28544724791718
C	0.07086723222615	3.91342641203610	3.41390101721662
H	-0.38867138837292	3.80369838349165	4.39125805078460
C	1.46455890344020	4.00734668113978	3.30992271288118
H	2.07703662182722	3.94895185298782	4.20463426971299

C	0.16075354468780	4.45186816249845	-1.30375428925788
C	-0.22820139029719	4.57035605536371	-2.63448481996845
H	0.50213536660952	4.76545402053780	-3.41423918665306
C	2.07209172920787	4.22204518006361	2.07901851377079
H	3.14488254342395	4.37853038386010	2.00642374592572
C	1.26231885878607	4.28142446656025	0.94759267806613
S	2.72540856721894	0.40605095972204	-0.44471232723235
C	1.20176388093816	0.63675924521583	0.48829357716725
S	1.80673433376151	4.57797096106224	-0.69718960161583
H	1.48260762646003	0.98655483563847	1.49452370957105

126

B+DBT r2SCAN-3c

Rh	-0.73889832981653	0.49941898857510	-0.13576551712464
Rh	-2.94601661272799	1.16972814278992	0.54150957126465
O	-3.03374358302062	-0.57304653344898	1.59114093139922
O	-2.14803482572370	2.09298204072436	2.18487712207639
O	-2.80533443720748	2.92453218203031	-0.47384843769984
O	-3.62522955423442	0.22191171748389	-1.13310649059148
O	-0.99280809138357	-1.24858835506758	0.89748926594428
O	-1.54486476139459	-0.39218876555152	-1.77958641488940
O	-0.76358891728161	2.27780997515369	-1.19839129297341
O	-0.09438974141554	1.33536624504073	1.63640216165333
C	6.42723656523419	1.01209127003278	-1.73401415582847
C	6.11666216890389	0.14886814288782	-0.68954001744820
C	4.90823250345869	-1.64871376776298	1.67285009285572
C	4.18031368008367	-2.33697837444234	2.63715687121601
C	5.49271208643011	1.93033455776812	-2.21659669234852
C	4.84599425687028	0.19601209721596	-0.11669460756922
C	4.29665509743125	-0.61966822748440	0.95991385914743
C	2.84839318660082	-2.01899067655535	2.89838970465228
C	4.22398333452064	2.01003351385606	-1.64928388029301
C	3.93429391979093	1.13187157447978	-0.62105006587671
C	2.96458577280838	-0.31295703113452	1.26827242969596
C	2.21676810140827	-0.98115687133481	2.21625796909073

C	-5.79011355915108	-3.57821280755511	-1.20521904707042
C	-5.82113754236551	-4.43533673192031	-0.11418897036024
C	-4.64209348357249	-2.83474515448693	-1.50190429384185
C	-4.71071124378778	-4.55653045282686	0.71715538962825
C	-3.53027904716552	-2.99661910132210	-0.67917254088143
C	-3.55441867712550	-3.83110853461101	0.44222321131589
C	2.03015268941495	4.57749982600029	2.04986817111239
C	2.72284869424724	4.80223148850034	0.86320579988544
C	0.64094128610439	4.44919458328440	2.03236813804230
C	2.03768178178428	4.89127790078824	-0.34315729925285
C	-0.03158535070583	4.56614808954352	0.81300460685054
C	0.64363458856349	4.77499136778724	-0.38823349000039
C	-0.14858837848435	4.16385481712218	3.28294410047151
C	-0.04391561049811	4.87296286355846	-1.73513045468331
C	-4.70768765978923	-1.85500528182723	-2.65547438111435
C	-2.35242212737761	-3.89418536347277	1.34753231596534
C	-3.45457211867915	-2.64180877382945	3.26006868773963
C	-0.95255141696229	-2.90226025391304	3.18698153630566
C	-3.78431297645281	-0.04670773452986	-4.12237978948978
C	-2.41185434127811	-2.10540573475107	-3.71738971239165
C	-2.48470127797569	5.51483381211900	-1.35358159055579
C	-1.83231117948929	4.12739481517144	-3.32919745620861
C	-1.30308501174034	2.52624737964221	4.80326253445961
C	1.02650796768325	2.02859436169286	4.01401486914305
C	-0.31902745280951	2.64806398133616	3.63178060037903
C	-2.22706534995560	-2.70422060940616	2.35010098660967
C	-3.41532137337922	-1.13368464102026	-3.08843882889538
C	-1.52080182798032	4.42890780827811	-1.84690572795678
C	-1.72504646685771	3.11284302926522	-1.10100083504861
C	-0.90906075917587	1.96609352038265	2.39759951615811
C	-2.08624530822799	-1.40485287705951	1.55816689673237
C	-2.82171078288100	-0.38139579417356	-1.90071941186444
H	7.41622276601474	0.97390688321318	-2.18022044797370
H	6.85304315964066	-0.56191375188716	-0.32795503793135
H	5.93843620295513	-1.92004313367951	1.46529374639033

H	4.65332103229023	-3.14505038055136	3.18554270868668
H	5.75705079381600	2.59597937961644	-3.03186606896253
H	2.29254738583836	-2.58452668184596	3.63941976271497
H	3.49273380855302	2.73192241683951	-1.99884670577210
H	1.18079465975142	-0.72070340051185	2.40019316411656
H	-6.67045219106819	-3.47709284219482	-1.83613550019258
H	-6.71915528186150	-5.00888621255929	0.09528744295846
H	-4.74505511212862	-5.21942061700954	1.57833387723626
H	-2.60359532937226	-2.47183513163555	-0.89023586546241
H	2.56923248129462	4.52012891192804	2.99204626148091
H	3.80316067386408	4.91813912555032	0.88025294749689
H	2.58800514155929	5.08457885397375	-1.26237502031534
H	-1.11450756696452	4.48354347309358	0.82908390222216
H	-1.14954893417346	4.60034952858053	3.19116551482645
H	0.33319244212665	4.62978808574041	4.15127892536085
H	0.55007362033487	4.28842557426428	-2.44844180553882
H	0.00520155493915	5.91341511279061	-2.08657835076853
H	-5.09654537580094	-2.37310702255271	-3.54255450398027
H	-5.45456491675357	-1.09411356926986	-2.39589227703607
H	-1.43636316155158	-3.94024641347936	0.74704176088344
H	-2.38931951258401	-4.80827795588417	1.95301833238546
H	-3.37097020499556	-1.82146260188065	3.97882542020790
H	-3.53539012850742	-3.58118691142738	3.81757470626234
H	-4.37280640219037	-2.50013079925848	2.68679640238829
H	-0.07057333207718	-3.00269545629839	2.54841411762725
H	-1.05197074533413	-3.80882890220188	3.79299731974702
H	-0.79735310348366	-2.05799337909310	3.86934599172271
H	-2.88933246728221	0.48817179874980	-4.45821758969847
H	-4.48940200343803	0.67580591614160	-3.70109864021145
H	-4.24672720861514	-0.51651893907622	-4.99661692868494
H	-1.50613993407412	-1.58617892377961	-4.04131478901331
H	-2.87202986649668	-2.56898915498509	-4.59583898958227
H	-2.12283719719339	-2.90533664031759	-3.03120360086294
H	-3.52151642345293	5.17691510067327	-1.41349414291299
H	-2.37269582542747	6.39853224832420	-1.98987388225336

H	-2.28558639009850	5.81904153466173	-0.32327128300029
H	-2.88330276045649	3.84769570729774	-3.45366056336131
H	-1.20361412566886	3.31505394909597	-3.70701933508858
H	-1.64389210099839	5.02282883676142	-3.93084334260063
H	-1.46274256570893	1.47640850329265	5.07086286989195
H	-2.27148271260412	2.96748188603354	4.55873291049086
H	-0.89298705588884	3.04071955366757	5.67821238423245
H	0.91215317310293	0.96945535324242	4.26591614661010
H	1.42151565289326	2.54154506590480	4.89713826916945
H	1.76147028232783	2.11296641069014	3.21029420819173
H	0.87988564583268	-5.26913413609690	1.68846386801959
C	1.72710103624245	-4.93601941627183	1.09609201480369
H	3.08955499922771	-6.33618530434739	2.00872375132263
C	2.97779684943273	-5.53884220176871	1.28021987915136
C	1.56072688074875	-3.92799698503385	0.16015196016850
H	0.59572326805761	-3.44724704926244	0.02506788074084
C	2.65031888597123	-3.51926110985564	-0.61437372671438
C	2.68977673960917	-2.53454298887667	-1.66859502624047
H	0.61930871011675	-1.93009620552401	-1.85766712242917
C	1.63489332756235	-1.79203513698239	-2.21615558690481
C	1.88327899501073	-0.94201351087271	-3.29297274266890
H	1.05596009177903	-0.39834801488730	-3.74105759108790
C	3.17380909845350	-0.79942438311030	-3.80954415927757
H	3.35064573242249	-0.13506254271736	-4.64986926306615
C	3.90580892766322	-4.12628109496177	-0.40417385574597
C	4.07992139187142	-5.13657091580853	0.53731346271560
H	5.04735232699540	-5.60827034874682	0.68086038289611
C	4.23339153165739	-1.52612949292885	-3.27992513921416
H	5.23100965126356	-1.43329572466673	-3.69786941553338
C	3.98016728004455	-2.39353216169108	-2.21997918340066
S	2.41076688725534	1.09962481432532	0.31191622365281
C	1.08783688125267	0.54571967232557	-0.76477627109476
S	5.15181894439396	-3.46422197201636	-1.46078536351247
H	1.28142351881939	1.00493317800563	-1.74354933621364

XYZ Structures (indene -> naphtalene pathway)

17

indene r2SCAN-3c

C	0.74657117467959	-3.84203862943711	-4.83828857057799
C	-0.50823594293283	-4.44705222775399	-4.86882644034251
C	1.41673007645074	-3.75827015465356	-3.62007699343664
C	2.71759885275589	-3.19466908924953	-3.27296836173177
C	-1.08345211497701	-4.95878466759079	-3.70548909899827
C	0.83407516821475	-4.27560660800116	-2.44678182249555
C	-0.41256502944897	-4.87556434487968	-2.48260608823772
C	2.94118785268543	-3.34898363256389	-1.95508062762253
C	1.77926950269422	-4.04517230469423	-1.29841515839066
H	3.82468697485336	-3.02686798565833	-1.41614062564063
H	2.08459417609727	-4.98663662955055	-0.81952752119001
H	1.19091214269713	-3.44478211344368	-5.74707859620541
H	-1.04644155728810	-4.52176293899445	-5.80948767065806
H	-2.06262678545072	-5.42643005684559	-3.75132809172164
H	3.39108965533631	-2.72570465361786	-3.98229128744940
H	-0.86691964273315	-5.27668208289551	-1.57971378938053
H	1.32762549636607	-3.42811188017006	-0.50828925592065

122

B+indene r2SCAN-3c

Rh	-1.78508194723221	-2.09137441959459	-0.70206267413867
Rh	-0.34987601632967	-0.32352038287441	0.06808215905305
S	1.75135292265847	1.78369152642339	-0.93693097274079
O	0.76485362933170	-0.82981676953846	-1.62135232046715
O	0.56394118464011	-1.74771480835084	1.22757227939497
O	-1.68113759963841	0.03695723105903	1.56291771944044
O	-1.37610930891074	0.99050208295998	-1.12549934193527
O	-3.04898745372440	-1.58873722856653	0.80618365278613
O	-0.80364025652790	-3.40466046664846	0.50808200057346
O	-0.52676423261069	-2.58144861815110	-2.21567554392858
O	-2.65563860553857	-0.69496105898587	-1.92194440982024

C	-2.77786131352167	-0.63037286309371	1.58276289082841
C	-2.29903134743179	0.51455136566080	-1.88588621013208
C	0.46599357863875	-1.81497534890307	-2.37716758652430
C	0.18882391037155	-2.96813357789020	1.16089538853340
C	1.01668948925752	-3.95726549538952	1.97870534093138
C	-3.81658750814776	-0.26372450599198	2.63755707575262
C	-2.99956212395427	1.51877294920362	-2.79928182644317
C	1.40423555535765	-2.10241092724932	-3.54830832823156
C	2.29527245800099	-0.89682559705905	-3.85098128652188
C	0.56317846615887	-2.45721574950185	-4.78361853102422
C	0.53549547931941	-3.80421416985767	3.43847051534934
C	0.78009891653119	-5.40540824659218	1.53440717978261
C	-3.54775687924571	1.10343286503630	3.27297753246174
C	-3.70435264795755	-1.35840202793396	3.72219998885513
C	-1.92291081098298	2.28844901412849	-3.58442215622246
C	-3.94115113976178	0.79797950568894	-3.76446370070608
C	-3.79140533322181	2.53111914774588	-1.91364829021105
C	-5.22849365151539	-0.33045611194205	2.02000096812864
C	2.50795477018938	-3.56898330579313	1.90843610319477
C	2.26622045374344	-3.34632426218839	-3.15670223526399
C	3.13371585456671	-3.32085043517703	0.55062398819416
C	2.46409224892767	-3.48749292922468	-0.65964773958063
C	4.45846544484686	-2.87054277341291	0.50864075767875
C	3.06448223825133	-3.19447283947155	-1.88819922428101
C	5.08072737971963	-2.59926363212261	-0.70316745649733
C	4.38653971031067	-2.75204839885002	-1.90184000477668
C	-4.89177538370513	1.91138177897103	-1.09192951291481
C	-4.57443994825019	1.21596058629468	0.07876673566066
C	-6.22232928626734	1.97120559575371	-1.49857427933722
C	-5.54096316257543	0.54347566998572	0.82263245923532
C	-7.20857715289457	1.33737703584267	-0.74721576112413
C	-6.87100147413669	0.62434985293401	0.39476335693024
C	0.04924397043359	3.93968728062903	-0.26059030923106
C	1.33074796895543	3.46543875160598	-0.45684509859346
C	3.49652435210447	2.14053923823994	-0.81550805952509

C	4.50716812186424	1.20913902179216	-0.96661277639849
C	-0.08306846138728	5.29596375572841	0.03002595189961
C	2.48429119452733	4.25616798137470	-0.37833236406443
C	3.71803068930723	3.50393667559084	-0.57854788913398
C	5.81587738718600	1.67604758741050	-0.87273822696347
C	1.04019079268124	6.12088296708658	0.09853085060921
C	2.31988740340226	5.61368720560545	-0.10090522626544
C	5.03781826724980	3.94878946616889	-0.51330675241613
C	6.07358513745527	3.03152885356279	-0.65907675936323
C	0.32556227753655	1.83314634006519	3.76508124608015
C	0.33993521903095	3.30658025226391	3.47241618728947
C	2.35426862260623	0.18286507324532	3.55314322572064
C	1.72954756145369	1.41332534156735	3.43699834978841
C	3.69607149986441	0.07301052213875	3.18195075454064
C	1.54874552410010	3.69335427935497	3.02254199157579
C	2.44158965893150	2.54300181990947	2.97661783850123
C	4.40032049129686	1.18532516943459	2.71306005124458
C	3.78367186716594	2.42938459501629	2.61186426022113
C	1.20907279760092	0.74431738659750	0.42396211278572
H	2.94899255985745	-0.65393317306978	-3.01027495493204
H	2.92374636199907	-1.12212904028777	-4.71888027649978
H	1.69401410936087	-0.01299850190253	-4.08664946291185
H	1.22874380711820	-2.68051313509476	-5.62348523669137
H	-0.07139989012769	-3.32621603572844	-4.59789524134754
H	-0.07867016656134	-1.61839771253326	-5.07317455467985
H	1.12458330109284	-4.45916198180353	4.08889089102936
H	0.65096912056059	-2.77387789153537	3.78763051326705
H	-0.51719919711735	-4.09062553145581	3.52988050721484
H	1.13236723308807	-5.59658194445214	0.51840544151691
H	1.32769934215841	-6.07159333962666	2.20885332167699
H	-0.27999537366101	-5.66424013713253	1.58129926028982
H	-3.56298386617624	1.91445737384697	2.54088349547184
H	-4.32685080714020	1.30503768908995	4.01503027342825
H	-2.58271103022077	1.11688799411032	3.78480354545833
H	-4.43769428635458	-1.16548745984785	4.51201411179976

H	-3.89554179590910	-2.34997641430845	3.30226614312266
H	-2.70702842244740	-1.35322524739124	4.17523960785157
H	-1.30556385159451	1.60323103150748	-4.17557208385819
H	-2.40674597868438	2.98659447323007	-4.27475604477415
H	-1.26812367672292	2.85671686683125	-2.91749281934948
H	-4.70758568758410	0.23205653070178	-3.23123478266639
H	-4.43641910252776	1.53705492821715	-4.40335293750728
H	-3.38889462554633	0.10472610467849	-4.40540389263904
H	-4.21006701437097	3.28232987046136	-2.59415863947699
H	-3.07904881808096	3.04910447671620	-1.25925034504016
H	-5.42664452928958	-1.36972125884229	1.72835421377797
H	-5.93878119110852	-0.09982769339347	2.82516562255995
H	3.07299615904518	-4.35880918980548	2.42293666096937
H	2.65216202108130	-2.65624640432432	2.49881531307414
H	2.93605114838652	-3.54041995455153	-4.00333210851586
H	1.59250752476814	-4.20645151941458	-3.06866204108005
H	1.44355685871223	-3.85762963230833	-0.67902021268492
H	5.01129277061923	-2.74874532012362	1.43771699709906
H	6.11930211894804	-2.27911841917982	-0.71572256001690
H	4.88060304787146	-2.54743542428022	-2.84881749331219
H	-3.53586878494967	1.21637473709983	0.39401587264668
H	-6.48999775028435	2.51350099687575	-2.40209836242132
H	-8.24688149934881	1.39364209160781	-1.06035253375640
H	-7.64775396933856	0.11600070697932	0.96170630774623
H	-0.80759379719933	3.27649760237861	-0.31811955565190
H	4.29783365580754	0.15436990219218	-1.12668557770652
H	-1.07141703081403	5.71082495362101	0.20018249559721
H	6.63985572639562	0.97681237642951	-0.97273783852513
H	0.91455324123960	7.17709972436784	0.31549633124005
H	3.18359071654488	6.26891481047513	-0.03908204739031
H	5.25745707184782	4.99792574560921	-0.33991076582614
H	7.10134494673681	3.37632053900784	-0.60192951416160
H	-0.40850904713363	1.30408605148856	3.14060344227266
H	1.80977444651401	-0.68215807724567	3.92151104528344
H	1.83192370775959	4.70130719863438	2.74012507812419

H	4.20681808502668	-0.88059691793436	3.27587625864941
H	5.44704028122312	1.08087305397934	2.44247156115357
H	4.34594328454947	3.29633645315008	2.27713397676544
H	0.06090954063111	1.62310353589217	4.81130892128570
H	-0.52146443420507	3.94938399618012	3.61399135269206
H	2.07258098544929	0.33853972049234	0.97095126211505

122

TS2 r2SCAN-3c

Rh	-1.91974307465814	-2.21516347174941	-0.83202199594472
Rh	-0.53460855237465	-0.38771534754008	-0.11366448199232
S	1.64597905369262	1.67803942216926	-1.07166142333348
O	0.59385141963611	-0.89193218502337	-1.79176075732989
O	0.41047743315387	-1.76103370946240	1.08491888309987
O	-1.86380805219665	-0.03383456070625	1.37899831402763
O	-1.60128031444060	0.87002331560197	-1.32332251990007
O	-3.18300306168705	-1.72690775976434	0.68193739358335
O	-0.89354758678621	-3.47898302991910	0.39453100746503
O	-0.65008662615351	-2.69141525897836	-2.34244960036293
O	-2.84742777440433	-0.86515809793915	-2.06248446391058
C	-2.92955183532683	-0.74643761074578	1.43686285333476
C	-2.52767480547119	0.35531770226814	-2.05352110102436
C	0.32360969954222	-1.90471054552495	-2.52185774324188
C	0.07839210107069	-2.99521028860231	1.04414222608267
C	0.93813523244161	-3.93153788486094	1.88919198957832
C	-3.94883165755917	-0.40203863453037	2.51660213566868
C	-3.27862820761823	1.32595409870055	-2.96265403255565
C	1.27175547655533	-2.19995379361238	-3.68324672917260
C	2.15781944358768	-0.99319299262436	-3.99542993590449
C	0.43791221419411	-2.57300644572094	-4.91857410525413
C	0.46559965907150	-3.73336683279813	3.34656227121075
C	0.73695192096288	-5.39953122157715	1.49746666541985
C	-3.67989566621168	0.96597217822585	3.14933827388466
C	-3.79196416846327	-1.49770635600963	3.59455630115261
C	-2.24306876740774	2.10376137321088	-3.79370395729865

C	-4.23174528017319	0.56633071814945	-3.88565211356834
C	-4.06444481162999	2.33843805868663	-2.07117798500360
C	-5.37392660185022	-0.48894548841179	1.93299620939745
C	2.41866008723604	-3.50678925874798	1.79333486649398
C	2.13881894505363	-3.43501565271226	-3.27498083536766
C	3.03546977142009	-3.30632543138683	0.42403086464530
C	2.35565708494726	-3.50661830856187	-0.77562364102092
C	4.36333207172934	-2.86733215185930	0.35850373282079
C	2.94858697107107	-3.25589052186036	-2.01715328692142
C	4.97991781539196	-2.64575508523258	-0.86620702119734
C	4.27531079400926	-2.82939183638477	-2.05456781120307
C	-5.13712601989667	1.71392770455150	-1.21666094462177
C	-4.78484108304239	1.04592170843359	-0.03983221470625
C	-6.47574630692043	1.74065332205086	-1.59958515923104
C	-5.72401125549995	0.36429597903319	0.73076385299919
C	-7.43540283309593	1.10018060533311	-0.81998324824460
C	-7.06281428238528	0.41147415115065	0.32594615095085
C	-0.02554372908815	3.87253891498882	-0.45486969696183
C	1.25215434317139	3.36597138299829	-0.58504145213057
C	3.39209915677548	1.97677876910893	-0.83189116065552
C	4.37844784945514	1.01415183079827	-0.93989690690388
C	-0.13585062791170	5.21939534182443	-0.11615976254933
C	2.42232360781593	4.11307023988025	-0.40094224366249
C	3.64202869024702	3.32641833280037	-0.54919869141602
C	5.69338209160892	1.43554359093666	-0.75893444447122
C	1.00614848744075	6.00156457716118	0.06645469199071
C	2.28066014140217	5.46202769493594	-0.07268989605385
C	4.96912293865868	3.72613722762479	-0.40031888835780
C	5.98137411044152	2.77777492327571	-0.50574465406297
C	0.40506095751525	1.02033973056612	3.71683306423090
C	0.03088931450275	2.41130387850558	3.28311693196462
C	2.81209334961422	-0.02917941329477	3.67352057171562
C	1.89023892155246	0.98643092741423	3.48482784723403
C	4.15113861726115	0.20440129719165	3.34737675185477
C	1.11919621689863	3.09478097872689	2.88165056973911

C	2.29810843161037	2.24130664390802	2.98712501586172
C	4.55749019682931	1.44804231140589	2.86170771967657
C	3.63741731359822	2.47966460773010	2.68658187199044
C	0.98795986823586	0.69861283062330	0.27699612152714
H	2.80039131322905	-0.73047100747433	-3.15172831057681
H	2.79819960914347	-1.22927778435940	-4.85166061346357
H	1.55307875224225	-0.11829592344264	-4.25421125024353
H	1.10838476131948	-2.79899310389752	-5.75379007220483
H	-0.19067501740163	-3.44513770625926	-4.72690770398279
H	-0.20893519565748	-1.74171032939092	-5.21847286504390
H	1.08515133246209	-4.33758043962713	4.01753986477882
H	0.54477999411162	-2.68419565225229	3.64881548688394
H	-0.57453937128923	-4.05516276317856	3.46226169297188
H	1.09197780534170	-5.61466149775255	0.48706514848604
H	1.30291719093304	-6.02814118712306	2.19245051018498
H	-0.31617698242772	-5.68329444766845	1.55627999728893
H	-3.76738051398432	1.78179758850712	2.42724134633806
H	-4.41257069530606	1.13741358020936	3.94419076635316
H	-2.68049109939738	0.99990307338870	3.59015772500329
H	-4.51521411359598	-1.32412052067955	4.39797573091998
H	-3.96695211648003	-2.49232848032718	3.17494681087409
H	-2.78712181242408	-1.46999790984334	4.03046605455560
H	-1.62682735078226	1.42128299129886	-4.38911830561289
H	-2.76285372858073	2.77658076983996	-4.48300108102070
H	-1.58296566311668	2.69997026286936	-3.15718211272537
H	-4.96682640264782	-0.00916557151747	-3.31951050370995
H	-4.76515969982796	1.28268034844109	-4.51962405011026
H	-3.68262377833423	-0.12327143651137	-4.53314035197531
H	-4.51049132104016	3.07341297958419	-2.75195225128221
H	-3.34398417180734	2.87440763251454	-1.44048076849913
H	-5.56794647949042	-1.53394021132374	1.65909286301177
H	-6.06745432219484	-0.25533857546933	2.75186706766768
H	3.00466330365054	-4.25852329646978	2.34030876491189
H	2.53904822682524	-2.56569813818985	2.34326693274847
H	2.80235218351358	-3.64309493026046	-4.12314402692488

H	1.46798089182452	-4.29492079852795	-3.16556695165642
H	1.33398242630991	-3.87396861158927	-0.77711615649612
H	4.92089585205983	-2.71181177305630	1.27967041654374
H	6.02127337438165	-2.33572337468772	-0.89683561957468
H	4.76402272594817	-2.65884554180581	-3.01093204771476
H	-3.74192359303354	1.07698256029905	0.25841005149642
H	-6.77066018435289	2.26192414933807	-2.50699794961118
H	-8.48017184192982	1.13118831168646	-1.11457570611238
H	-7.81861524618357	-0.10428207796907	0.91413957115217
H	-0.89702344166687	3.24040545418680	-0.59386194927352
H	4.14601721320002	-0.02968340801611	-1.13510645999205
H	-1.11992351585037	5.66059764063646	0.00505658417199
H	6.49835124269435	0.71044802532180	-0.82268942163968
H	0.89843678859445	7.05014726334015	0.32568237089631
H	3.15720078157103	6.08543429006998	0.07602788607780
H	5.21187461635128	4.76398280147175	-0.19280831600163
H	7.01466060799838	3.08753679613352	-0.38341760827482
H	-0.12331834827087	0.25302914067389	3.13306028346459
H	2.50664406022301	-0.98952523182835	4.08125099174254
H	1.13445890698690	4.12219969022628	2.53317394023372
H	4.88596074609488	-0.58191894938163	3.49331293116476
H	5.60541293886240	1.61719652159067	2.63134698690866
H	3.96190957806438	3.45335300500469	2.33114646705863
H	0.14941921919342	0.84411171061598	4.77251524451312
H	-0.98323900677809	2.79208076358244	3.31045302595137
H	1.78361975357230	0.36854613522002	0.96124869530473

122

C_endo r2SCAN-3c

Rh	2.67558445671945	-1.47769173751504	0.30305137111809
Rh	0.73036295242205	-0.07640872662051	0.01164428461949
S	-1.70813666054130	1.06878026558580	-1.75747025111416
O	1.85200910451825	1.04071811034679	-1.28812106463063
O	1.38312948546748	1.02379887416068	1.60995311324427
O	-0.27653793359029	-1.32062273631909	1.32355920176901

O	0.18122374478892	-1.30207523672299	-1.55410601050747
O	1.53121623161634	-2.66172655655356	1.51191197311068
O	3.17527202059065	-0.32166038718445	1.90991413982917
O	3.69948314300931	-0.20330549561482	-0.91391026970096
O	2.05624055282473	-2.54819350494934	-1.32279231039166
C	0.33411623712288	-2.34843371052844	1.77648149989975
C	0.94073631218219	-2.28632467135435	-1.85870870717976
C	3.08283226653475	0.75894833174922	-1.46435202154610
C	2.47681367017569	0.69523700001359	2.18745864854800
C	2.93534478824269	1.60277855089612	3.32769478312427
C	-0.42534250515340	-3.28804683343363	2.71788334329813
C	0.45757389388866	-3.20208606302779	-2.98584368801288
C	3.89139778138867	1.66026782766969	-2.39898801422586
C	2.97939835671254	2.62839891335773	-3.15398379693583
C	4.64976470783928	0.76627221397046	-3.39384961696079
C	2.04693437206929	1.23886769738162	4.53698632112642
C	4.40190824420344	1.35400752083517	3.69469888807625
C	-1.82712177315503	-2.76307966788877	3.02669378009780
C	0.39023798727110	-3.39643091197775	4.01862604321760
C	0.65666493422391	-2.40425689207466	-4.29183190778466
C	1.27993971281115	-4.49244403228117	-3.04662984412633
C	-1.04581770536356	-3.51231162591469	-2.83321233743347
C	-0.50270458974762	-4.70109170490683	2.06376367280521
C	2.68881798702382	3.08112838897898	2.96722827221973
C	4.93434807939095	2.43980285731780	-1.53819235078488
C	3.25673827975155	3.61986561417179	1.66900217827047
C	3.89767927723754	2.83360115952360	0.71441417142975
C	3.08765142096853	4.98157913010864	1.39241707964150
C	4.32913455577451	3.35770035287330	-0.50808760401992
C	3.53737877060717	5.52575097836021	0.19678041506748
C	4.14910827524863	4.71675090510741	-0.75753141831138
C	-1.51952334226208	-4.21739184392187	-1.57921199125240
C	-0.79457967815871	-4.23128420363059	-0.38820483385472
C	-2.78134599850830	-4.82410807165905	-1.58451523820359
C	-1.31318514887851	-4.76883723310890	0.79382719741811

C	-3.29938035356477	-5.39539543611159	-0.42846357050333
C	-2.57977184773094	-5.35165546235970	0.76412604774526
C	-2.96093277972330	-1.18575516572379	-0.62624278041912
C	-3.07802983118873	0.08231393190567	-1.17013526971728
C	-2.84166027599307	2.39537345258586	-2.20846624830111
C	-2.46267268249253	3.59640030911730	-2.78370275675914
C	-4.13440396597888	-1.81513803327527	-0.21062026665838
C	-4.30862406077121	0.73813875379051	-1.34271219456584
C	-4.17368382466277	2.06433567492454	-1.93074792024234
C	-3.46655618785346	4.52104384297919	-3.06377407983037
C	-5.36820580162740	-1.18382534943113	-0.35916198607998
C	-5.46488385068437	0.08648785051468	-0.92299934802413
C	-5.16420027454440	3.00109096940462	-2.22650801105481
C	-4.80193191851505	4.22265057514566	-2.78451484663573
C	-2.35775377859524	3.19974461912555	0.96536908930570
C	-1.51673079392613	1.89217964482308	1.00888046762387
C	-4.85111999543101	3.53540268768478	1.66211404342225
C	-3.69339594416801	2.79811285239820	1.50409565224023
C	-5.96547029025791	2.91271511021624	2.23018771614054
C	-2.41587178775412	0.88838304804421	1.60037026797002
C	-3.66246349762918	1.42736472433452	1.90104293464879
C	-5.93743214586182	1.56771607498348	2.63987776488054
C	-4.79648804329631	0.81056218825173	2.48091632993519
C	-0.72583771483593	1.44955139678913	-0.24814937974730
H	2.42687841241366	3.27717974848005	-2.47014433268593
H	3.58665868632784	3.25908507812443	-3.81241166875176
H	2.26019158724173	2.08176819758576	-3.77207963037930
H	5.23586355869518	1.39201728661350	-4.07481292571268
H	5.32470444576463	0.08123738995902	-2.87564373693037
H	3.95115224918496	0.17380639336513	-3.99444320878585
H	2.29742172260473	1.88408371686618	5.38569610127043
H	0.98552333935549	1.36960586145458	4.30168404427402
H	2.21470293689176	0.19971642546906	4.83854582325050
H	5.08217291394819	1.62489996186897	2.88381224476061
H	4.65757350513761	1.96460560875838	4.56691936566904

H	4.57265787476236	0.30470684869539	3.94519052611769
H	-2.42203670297566	-2.64234423315753	2.11695082316881
H	-2.34566121406458	-3.47422786255714	3.67907136863449
H	-1.77807384374342	-1.80142299671288	3.54858401528423
H	-0.13196341301998	-4.04761147061954	4.72724834473175
H	1.38318504747177	-3.80894456172009	3.82547425592414
H	0.50989297057314	-2.41242625891123	4.48518971954160
H	1.71414355216562	-2.15914021105893	-4.43466246628216
H	0.33038866804992	-3.00738500900618	-5.14565656860280
H	0.08054120219495	-1.47357669397603	-4.27919639804717
H	1.18159209462287	-5.08255754821731	-2.13226598714000
H	0.92656552768837	-5.10199702379501	-3.88503376736436
H	2.33996790336870	-4.27693202946797	-3.19771751575369
H	-1.34157635749802	-4.10991183503576	-3.70540531214476
H	-1.60259832279539	-2.56743496327912	-2.91506448162116
H	0.52170918713826	-5.04051402025749	1.87188168370558
H	-0.93450837944018	-5.37535234940114	2.81311394969119
H	3.07569549419654	3.68319683835284	3.80094191324655
H	1.60466394345698	3.25621015014455	2.94811185390303
H	5.55254855352715	3.01638972059966	-2.23699666579692
H	5.58665430374455	1.70599950106450	-1.05098090233342
H	4.08474974719784	1.78025789643088	0.89691936056645
H	2.60575166458320	5.62144422240101	2.12919963139901
H	3.41381590699421	6.58820477496301	0.00687243630003
H	4.49440539271485	5.14603530617246	-1.69501043021406
H	0.21404457724684	-3.83615389669519	-0.35486077318168
H	-3.36021114422412	-4.84966954482381	-2.50524120711922
H	-4.27229068627098	-5.87857769461974	-0.45566710570734
H	-2.99374512403817	-5.79385859544030	1.66727321322862
H	-1.99532240699386	-1.66853776833313	-0.52235567883826
H	-1.42186472483036	3.81579354771699	-3.00430370029122
H	-4.07456563070478	-2.81561600128322	0.20593401552081
H	-3.20693240466938	5.47474509982959	-3.51184026345882
H	-6.27205834018211	-1.69379693655445	-0.03929547618093
H	-6.43444658466565	0.56278381094042	-1.03338230137325

H	-6.20892750164116	2.77928522536970	-2.02999645887757
H	-5.57121951870146	4.95222901344917	-3.01748218401422
H	-2.43338442735476	3.62869755130069	-0.03851273004465
H	-4.89926717258284	4.57838612154099	1.36220424293323
H	-2.08939313012070	-0.12529804194170	1.80926264199846
H	-6.87825668030550	3.48497517637623	2.36956511456547
H	-6.82530045709602	1.13002930471386	3.08521724646215
H	-4.75839240866580	-0.23048431274741	2.78668651336008
H	-1.90074480066961	3.97303076301712	1.59688195524934
H	-0.66423329318559	2.01329453071521	1.70742884114797
H	-0.09289291900455	2.28270568193520	-0.58779112459904

122

C_exo r2SCAN-3c

Rh	1.80445329803644	-1.93457895692330	1.08928314972102
Rh	0.36445889901747	-0.34476582514034	-0.00172377874572
S	-1.33365232910664	2.24942363537574	0.50055131604610
O	-0.65676982973565	-0.27835246699157	1.78771836325986
O	-0.73141319880686	-1.95889310667214	-0.62697299332363
O	1.48873844352799	-0.46670103414585	-1.68676026502034
O	1.58982642424395	1.15835511514151	0.68924497363288
O	2.85453110972679	-1.95362732313028	-0.66554645703211
O	0.59537451122612	-3.45052596927163	0.43827216710610
O	0.67330218463901	-1.80396330664471	2.78943068294727
O	2.91195974102153	-0.34651539252334	1.73714709134155
C	2.53004590980825	-1.21383971241094	-1.63886816312719
C	2.59779948109149	0.83291399738606	1.41685512721093
C	-0.29591982269934	-0.98842220715116	2.78429850643603
C	-0.42322622480835	-3.13650553274600	-0.24072865478322
C	-1.37769849850227	-4.23960266741572	-0.69345662251400
C	3.43648965640804	-1.24001495897744	-2.86735781098916
C	3.47298931248810	1.98014494409086	1.92473440838390
C	-1.13432180847815	-0.85767539232068	4.05784201169350
C	-1.77728461577228	0.53089263532682	4.12769204629374
C	-0.25060285302732	-1.08408896403121	5.29056821791119

C	-0.97914701110970	-4.56603253493474	-2.14953853062995
C	-1.22447868916115	-5.51380189925309	0.14568417354801
C	3.13500733394867	-0.08708166426933	-3.82889667536741
C	3.14286709607207	-2.58205816922683	-3.57305482296067
C	2.56305956626526	3.00167916885480	2.62799540986441
C	4.53007628210393	1.45898965013667	2.89926257968372
C	4.15626128364429	2.67718106451787	0.70795310624769
C	4.91570956498876	-1.23474591502920	-2.43124999149492
C	-2.82970684742012	-3.70794150598849	-0.68939438809687
C	-2.23218588107899	-1.97322418487006	4.02643804373643
C	-3.28909732970213	-2.87340333011849	0.48772409127168
C	-2.62162443438582	-2.83195311019943	1.70978111926969
C	-4.43608085363082	-2.08580173664784	0.33957069528603
C	-3.05430271999941	-2.02826665045900	2.76683978146809
C	-4.90531620541602	-1.31000470584042	1.39381884434832
C	-4.21907787383909	-1.27677411344947	2.60595196485466
C	5.10286046809917	1.79784602175032	-0.06757927430294
C	4.59640529830792	0.82614975958946	-0.93613836524793
C	6.48204450997467	1.89460245657567	0.10123051064579
C	5.42533157000162	-0.07774143195394	-1.59662539766238
C	7.32980954374438	1.02696298680713	-0.58234995109179
C	6.80731733568388	0.04632135832966	-1.41428476859641
C	0.60331059644560	3.75427638994110	-0.89583086518460
C	-0.68749440548233	3.64983223629481	-0.40675102895734
C	-2.95757303397643	3.01137946890548	0.55941240394252
C	-4.08769136377902	2.41541020392820	1.09023102168700
C	0.95216398075377	4.94152519457111	-1.53548004673695
C	-1.63899728043077	4.67654692661990	-0.51573764945329
C	-2.93964442795572	4.31185358819558	0.03448550831098
C	-5.26510652129818	3.16003104535890	1.07956993448816
C	0.03100706029924	5.98354578706336	-1.65237632407291
C	-1.25889055418137	5.86075607928280	-1.14838878681886
C	-4.12857455439074	5.04182275786644	0.04647773644324
C	-5.27960121003418	4.45987102182587	0.56871564368662
C	-1.41999169503883	-0.12555709058430	-3.05999566157136

C	-1.36306936247169	1.16877229078718	-2.21306081286953
C	-3.56145141308258	-1.45939938709296	-3.74942275955378
C	-2.88934977811685	-0.37127403341572	-3.22826970290990
C	-4.95682706456067	-1.41046504135212	-3.82439135813468
C	-2.70929579745128	1.73547689201687	-2.33280276651575
C	-3.61802368789398	0.77024259962138	-2.79005037265285
C	-5.68037820772758	-0.28828580931362	-3.39042048383783
C	-5.02505088530991	0.81153967212405	-2.87099734351963
C	-1.28603633608630	0.80541033374250	-0.67149842375428
H	-2.45477108496867	0.70533139772995	3.28916825237223
H	-2.34490173741635	0.62368513829908	5.05976445391748
H	-1.01212841766119	1.31402741634553	4.11445238005502
H	-0.86116822505340	-1.01004925095760	6.19644104557287
H	0.22359410738594	-2.06734891402378	5.26655142351482
H	0.53892209829239	-0.32777516523926	5.34913610379192
H	-1.67196381800834	-5.30654701628521	-2.56393533278592
H	-1.01058738213720	-3.66963201253647	-2.77832529661978
H	0.03179519921373	-4.98406015332688	-2.18881234940174
H	-1.54181641518378	-5.37866212065567	1.18219702198074
H	-1.84518464562819	-6.30293094946172	-0.29126967231209
H	-0.18693883145841	-5.85482178375187	0.15247193421887
H	3.32586477481033	0.88827558201925	-3.37409899709147
H	3.78083893861667	-0.18109058568270	-4.70801682811921
H	2.09457114247080	-0.11405527181240	-4.16417661880162
H	3.77601656251119	-2.67559541845602	-4.46145471168644
H	3.34413843725243	-3.42720704746575	-2.90860428667196
H	2.09639517467448	-2.62910249933283	-3.89425329372900
H	2.02522067860638	2.53548261280740	3.46083376154795
H	3.17290445263883	3.81543052647495	3.03364869324851
H	1.82903687546416	3.42799412743162	1.93756717339687
H	5.18082438125036	0.72088006860729	2.42627072527811
H	5.14701078501627	2.29594640013755	3.24400521622248
H	4.06366750278280	0.99208207913152	3.77124105694673
H	4.69658578041548	3.54468474928203	1.10592747209413
H	3.37160492475280	3.06534319646732	0.04758510149561

H	5.10648481302424	-2.15545339235388	-1.86482460849027
H	5.52094532992085	-1.30825543246459	-3.34471133187134
H	-3.49417345342377	-4.57760455530284	-0.79498953235548
H	-2.98144130694862	-3.10012123175925	-1.58887555469218
H	-2.87608469139942	-1.80257704539036	4.89807945763044
H	-1.73165747547036	-2.93702745710135	4.17452161429238
H	-1.71907365896854	-3.41669621268870	1.86277017180267
H	-4.97466803474381	-2.09581336043137	-0.60644947699597
H	-5.82072980992404	-0.73595869965304	1.27191250774977
H	-4.59682622519406	-0.67943243421444	3.43283321914544
H	3.52132326315488	0.79276712074684	-1.07918589017968
H	6.89540214036733	2.64714449051577	0.76829270650385
H	8.40530741739547	1.11079053791320	-0.45696628860287
H	7.47844839494449	-0.64053328826034	-1.92525691421382
H	1.30639549565998	2.93637973410579	-0.77901139800410
H	-4.06258053148979	1.40145000856420	1.48183380628300
H	1.95349528017428	5.05473531794037	-1.93884662501321
H	-6.17570321354995	2.72666549599587	1.48099671615473
H	0.32513899665018	6.90472035724091	-2.14549886105852
H	-1.96408616513353	6.68029648897456	-1.24761514130114
H	-4.15816027593542	6.05335770295530	-0.34707420708021
H	-6.20497343287712	5.02726752068943	0.57966425010114
H	-0.88588545225513	-0.96765059167113	-2.61806290044977
H	-3.02543500798624	-2.33498935381752	-4.10509989468284
H	-2.98063351154910	2.73763941378071	-2.02189413883368
H	-5.49669731127705	-2.25979284688637	-4.23306609489102
H	-6.76300060097461	-0.29106352935786	-3.46854257251302
H	-5.57208533977897	1.68606726923896	-2.53072275836138
H	-0.96261081148805	0.07988448621060	-4.03768352025988
H	-0.53280823302805	1.83155500623365	-2.46145277141335
H	-2.15279570215547	0.20042922296873	-0.36496773007167

122

TS3_endo r2SCAN-3c

Rh	2.81603048112394	-1.60676799120372	0.33994933280530

Rh	0.86549650752751	-0.19208052181785	0.25007700053765
S	-1.12105533934934	1.29826737353349	-1.69720193019177
O	1.93620084708997	0.84399478806752	-1.17997661056685
O	1.65807728749191	0.86252040604222	1.80903457789934
O	-0.01548008210089	-1.51561175990497	1.61059609717326
O	0.18490198069384	-1.36982026960978	-1.29618602337704
O	1.81173617268137	-2.84249964918817	1.59178389639299
O	3.48569684021147	-0.46834643087158	1.90805571229295
O	3.80962218902620	-0.37744931442084	-0.94140907117278
O	2.06444078820157	-2.63184528437883	-1.25903361710781
C	0.63663936836104	-2.55328269876234	1.96682771276142
C	0.91566548272460	-2.33650677975666	-1.70197946753158
C	3.14534226297001	0.56311996673591	-1.46597161612441
C	2.80781098742071	0.53772777775693	2.26363954554767
C	3.36045381989876	1.45150351931955	3.35511525568738
C	-0.02400251977325	-3.55315741320009	2.92172866655002
C	0.34567205892627	-3.18032605430992	-2.84258265497223
C	3.84157794417019	1.45894011724451	-2.48846682964645
C	2.81035484040323	2.16246317654128	-3.37539560323253
C	4.78726201485367	0.61637932579855	-3.35456460773934
C	2.66711059666757	1.01365441834410	4.66404994574271
C	4.87496739659782	1.28856326654021	3.52890675352422
C	-1.35324034887462	-3.02476490349655	3.45111271966905
C	0.94514654959532	-3.80099817024290	4.09061744628896
C	0.48700688480936	-2.31564124695516	-4.11382420138054
C	1.12819704388351	-4.48317683774135	-3.02901263643064
C	-1.15314066162061	-3.45521091406124	-2.60878748412451
C	-0.23930697816629	-4.89963222533055	2.16283197748562
C	2.96609479334261	2.91656382081236	3.05850172809814
C	4.69395122076458	2.50059883041058	-1.69125918499855
C	3.10684098509662	3.43114058355238	1.63985567898227
C	3.85207094485549	2.78924187682852	0.65213896415291
C	2.44013248367424	4.61187695690324	1.29425451757077
C	3.93366905674494	3.28214800171008	-0.65317910368158
C	2.53234421012748	5.13266467522474	0.00854319965504

C	3.27578354478583	4.47202591101352	-0.96567340501200
C	-1.57510991861441	-4.12500473752220	-1.31778529884946
C	-0.73167102992802	-4.29306838868380	-0.22135768383246
C	-2.90371262846172	-4.54896246066975	-1.19195549014248
C	-1.18588258180630	-4.81481933353606	0.99322800482590
C	-3.36641463540410	-5.09613565205909	-0.00174097664595
C	-2.51650536256836	-5.21712359445709	1.09548322908709
C	-2.83953070077510	-0.76080445355224	-0.77274341573649
C	-2.68427825382781	0.45260137903044	-1.42040717994025
C	-1.98918059504120	2.68282164924833	-2.46612508788411
C	-1.38466397297867	3.83945399876390	-2.92504421638884
C	-4.12634740468745	-1.29031482574418	-0.70706477234573
C	-3.74617153540127	1.13759225911610	-2.03211068873099
C	-3.35205979127320	2.40767947447440	-2.63120868630594
C	-2.19426245320385	4.77823839640075	-3.56078560071734
C	-5.20072361791267	-0.63400022928743	-1.30829453776671
C	-5.01948625138529	0.57154879783450	-1.97495665889264
C	-4.14178961039652	3.35394770894301	-3.28538296850634
C	-3.55672323024686	4.53121992484540	-3.74035203591745
C	-2.49820432612037	2.99824088362748	0.80619207032967
C	-1.10898259685590	2.34272876915324	1.01818934455619
C	-4.83818953665493	2.04059829308527	1.44064972981198
C	-3.45837627922103	2.00116088514380	1.38529391254614
C	-5.51008802525719	1.01296300361357	2.10605750197318
C	-1.37592339818015	1.15747144757795	1.88888461126361
C	-2.75100880691714	0.93436537503862	2.00919026302213
C	-4.81939198256330	-0.03391774056612	2.73779743410264
C	-3.44208996184277	-0.08577451548711	2.69814952228226
C	-0.35715518974246	1.70965885451096	-0.14452975067579
H	2.14814546068947	2.80662818611218	-2.79208140149952
H	3.33001210567760	2.77827720767207	-4.11726748254888
H	2.19382967525015	1.43139058494028	-3.90885884214567
H	5.30363057941918	1.26631517991609	-4.06852271965228
H	5.53347831736830	0.10134968055054	-2.74595147188651
H	4.23004046527417	-0.13636953327665	-3.92192134828026

H	2.96997912596046	1.67863195006196	5.47988892414202
H	1.57730684823873	1.05903025707140	4.56761142578220
H	2.95660028942611	-0.00776918710600	4.93117146122393
H	5.43838867901496	1.64781267561385	2.66475811312654
H	5.19523787942248	1.86980975197565	4.39972336262128
H	5.14019654799362	0.24224516993155	3.69547122355919
H	-2.05809203396149	-2.84396113332347	2.63573203056654
H	-1.79517375581979	-3.76092623198897	4.13141789879009
H	-1.20097942460618	-2.09550613793667	4.01033389580809
H	0.49137881574685	-4.50110453204690	4.79967288118938
H	1.89063331549820	-4.21902015119671	3.73819661971949
H	1.15833949286177	-2.86842737596251	4.62454523748322
H	1.54064559613781	-2.09529790915648	-4.31473815593880
H	0.08369494169473	-2.85835411565917	-4.97526570391058
H	-0.05611562886122	-1.37091411680953	-4.00975988790402
H	1.05833474637019	-5.13569018188447	-2.15510856028903
H	0.71653857371829	-5.02301191850639	-3.88801688169702
H	2.18518811553282	-4.28414089671062	-3.21952773208561
H	-1.50674509339872	-4.06011935402185	-3.45466446957027
H	-1.69325089469520	-2.50045639277019	-2.67501092643295
H	0.73997549791676	-5.26119719528364	1.82906028920821
H	-0.61929007145384	-5.61884570517075	2.89846782017742
H	3.55908335254555	3.55300906644251	3.73087959799816
H	1.92011107580258	3.06164341937558	3.35506916647727
H	5.13480004115683	3.17903590533904	-2.43229414693207
H	5.51833062608221	1.96006411718562	-1.21231656817767
H	4.38281611852406	1.86744103869259	0.87383328259472
H	1.86041538626582	5.13938296517274	2.04953729803072
H	2.03131691186521	6.06631318489219	-0.23243319747574
H	3.35678132263203	4.88925314619161	-1.96649470674338
H	0.31949362245684	-4.03413332480318	-0.28549074055412
H	-3.57859164089316	-4.45015754452893	-2.04002233746063
H	-4.39537137122102	-5.43795245846234	0.07110119591332
H	-2.88456871953843	-5.64263731275998	2.02607482920256
H	-1.99826657084686	-1.28064246200707	-0.32562270313360

H	-0.32111344540742	4.01399017896309	-2.78773215726384
H	-4.27609752514384	-2.23506841265043	-0.19452192462398
H	-1.75932253893807	5.70317906968892	-3.92545901996409
H	-6.19287249442392	-1.07221872454266	-1.25674490127781
H	-5.86211444880371	1.07057526309561	-2.44392756867373
H	-5.20160928707043	3.17629117298547	-3.44132783765137
H	-4.16999224943950	5.26993560652064	-4.24686059060368
H	-2.71227998458991	3.23805211628043	-0.23774823226940
H	-5.39587866061755	2.85055705683036	0.97879508169545
H	-0.59599931125184	0.61366986363403	2.40303150481020
H	-6.59564770909637	1.02831231192640	2.14601976087590
H	-5.37942113152862	-0.80330012661303	3.25998718733915
H	-2.89181724520207	-0.88607249455467	3.17702797004797
H	-2.57007143175381	3.94532932536071	1.35705872638947
H	-0.40047702316038	3.03381475636473	1.49370198127390
H	0.54028818761100	2.26331322497565	-0.43286380544881

122

TS3_exo r2SCAN-3c

Rh	1.93887212972777	-2.02442661761739	1.18894211843095
Rh	0.49502824748442	-0.46235390123707	0.09424052675621
S	-1.25784951947373	2.12118481797951	0.57454938755791
O	-0.55053467617802	-0.38200942615228	1.86319956059891
O	-0.59898204877058	-2.06688813591534	-0.53226354747212
O	1.65150351675479	-0.60597157129224	-1.58324014753087
O	1.72702115052327	1.04556085103204	0.78480772389347
O	3.01749930246864	-2.08165624002318	-0.54183399026980
O	0.73453000770114	-3.54381477989936	0.54168100395779
O	0.79952750630604	-1.87815204551896	2.88097749478409
O	3.05558604503936	-0.45002760432945	1.83812354456841
C	2.69164481256552	-1.34936695519613	-1.52351011443173
C	2.73650106590347	0.72844045417895	1.50991382306025
C	-0.18055274137246	-1.07415953939117	2.86810026013455
C	-0.28640102791749	-3.24123533159620	-0.14253519466924
C	-1.22973387449424	-4.35322291432696	-0.59153669776677

C	3.61089213167735	-1.39243138478961	-2.74344858454191
C	3.61365710695286	1.87648730767119	2.01209246073790
C	-1.01637218799331	-0.94127239994538	4.14181210823184
C	-1.66161379651371	0.44672618175054	4.20086641068034
C	-0.13368219546698	-1.15699052737470	5.37721948783586
C	-0.82114174373942	-4.68634714888562	-2.04348088817311
C	-1.07551542191912	-5.62246430345011	0.25519465011530
C	3.31435380129290	-0.25480903392990	-3.72379126829528
C	3.33040924168402	-2.74604090003693	-3.43230835751631
C	2.70228072833881	2.90167367881168	2.70758705106097
C	4.66986780122004	1.36375319938083	2.99242399383646
C	4.29888134614443	2.56453700626972	0.79135157987435
C	5.08792089035275	-1.37807561029221	-2.29764332371749
C	-2.68331212917467	-3.82642194493774	-0.59692340089479
C	-2.11069967419753	-2.06086674842063	4.11399224427261
C	-3.14648106884901	-2.98441812859308	0.57362362392555
C	-2.48794275894424	-2.93882774053233	1.80104460879402
C	-4.28791274205668	-2.19142425249924	0.41157496728003
C	-2.92537289416376	-2.12692831838885	2.84990850606679
C	-4.76200980610837	-1.40774296377644	1.45789418291049
C	-4.08616973925295	-1.37205186926725	2.67576892025626
C	5.25438841123777	1.68193574155449	0.03023242294043
C	4.75594994325066	0.69755341071269	-0.82835296010580
C	6.63242518478275	1.78760902942536	0.20315217339975
C	5.59141451827964	-0.20987344946749	-1.47540128103226
C	7.48692718312105	0.91602096381361	-0.46714803053648
C	6.97222660146787	-0.07692208449321	-1.28961204994626
C	0.69114472122652	3.63493263068119	-0.79864126377125
C	-0.59722221543816	3.54029492000903	-0.30347886537229
C	-2.85699564337677	2.93829434749356	0.72700018150059
C	-3.97913290717948	2.35739914409006	1.28509294820127
C	1.05357453770169	4.83385230893374	-1.40975406953769
C	-1.52906994504740	4.58888320274272	-0.36130777238766
C	-2.82325658632736	4.24330010641336	0.22006943591220
C	-5.13873037325166	3.12903715502288	1.32661622839532

C	0.14878553949680	5.89336936734586	-1.48890635832450
C	-1.13567183158479	5.78216101075274	-0.96676427043824
C	-3.99267510479430	5.00089874741775	0.28437467979296
C	-5.13867937373374	4.43664721505565	0.83583505767519
C	-1.25046636339931	-0.25223333589220	-3.00181279827559
C	-1.17621284369605	1.04013080195665	-2.17005086938330
C	-3.39511489574090	-1.58939615016859	-3.68090419555325
C	-2.72901625810113	-0.51181409094332	-3.12209774444815
C	-4.79003546806910	-1.55742786418910	-3.73936988957676
C	-2.53678465367771	1.55206248317509	-2.10349773327714
C	-3.46122021215364	0.57588229772970	-2.60115528191942
C	-5.51431339366950	-0.46512150969628	-3.25305337896431
C	-4.85780461985808	0.61154242267584	-2.66819961548598
C	-1.37713045426292	0.80093818854090	-0.65225064794129
H	-2.33469017102941	0.61316489490644	3.35738204395870
H	-2.23405366700429	0.54745530073428	5.12901743293016
H	-0.89628273803192	1.22983198501870	4.18497907135873
H	-0.74569027144710	-1.07641507056359	6.28159705111289
H	0.34179364028580	-2.13991147913299	5.36206967774914
H	0.65534215297476	-0.39959507771328	5.43086214475754
H	-1.51003220673154	-5.42999969400462	-2.45860168147223
H	-0.84995063361776	-3.79355143061145	-2.67734484581851
H	0.19076580730523	-5.10298163499485	-2.07296548481714
H	-1.39836762619866	-5.48407527456890	1.28966116078227
H	-1.69167044080777	-6.41541878382770	-0.18133769356384
H	-0.03690845498135	-5.96049159666578	0.26815552145837
H	3.49940012993275	0.72713079602792	-3.28078570114020
H	3.96697888454077	-0.35970732697607	-4.59665223808274
H	2.27624261894443	-0.28967073564219	-4.06542015942404
H	3.96971738094762	-2.84696722795134	-4.31557672798126
H	3.53447777995966	-3.58064732082036	-2.75534392786437
H	2.28633240294996	-2.80617221688712	-3.75883678439281
H	2.16414659815499	2.43903544609893	3.54232439887470
H	3.30972143238702	3.71916071436589	3.10924440344207
H	1.96832500018115	3.32164651903518	2.01323788705967

H	5.32203455913046	0.62190973784630	2.52724582818158
H	5.28602435475377	2.20409332074540	3.33051397191171
H	4.20280120882587	0.90445321754015	3.86818204641038
H	4.83235174113853	3.43972078242730	1.18181742445355
H	3.51413043742346	2.93911463013364	0.12265743567761
H	5.27687918540132	-2.29086788951575	-1.71788551696115
H	5.69874651222552	-1.46254205396992	-3.20651288058637
H	-3.34591114029918	-4.69792884277678	-0.69822242004352
H	-2.83433357385978	-3.22562293317088	-1.50122756899905
H	-2.75994179386874	-1.88709438021781	4.98109677722673
H	-1.60687856213859	-3.02149246549045	4.27144287202275
H	-1.58720450800318	-3.52405215458325	1.96347429861257
H	-4.81730313874907	-2.20276744186331	-0.53943543653745
H	-5.67390243616911	-0.83053584698093	1.32473426182879
H	-4.46835491821210	-0.77029169151902	3.49748077302060
H	3.68161244934991	0.65631733886154	-0.97425044598387
H	7.04011390018157	2.54971725511879	0.86292485470516
H	8.56156705796786	1.00666758352963	-0.33888282634936
H	7.64905457725335	-0.76603253516191	-1.78999748955545
H	1.38460040393843	2.80516543627957	-0.70297736251979
H	-3.96560001024657	1.33565772488182	1.65755132072433
H	2.05318538851103	4.94199210873857	-1.81798966891495
H	-6.04525546210700	2.70975271649285	1.75026699620524
H	0.45265498103271	6.82162861984602	-1.96274847105493
H	-1.82477167747722	6.61900691988194	-1.02916718456430
H	-4.01233430718140	6.01890723992691	-0.09312895967382
H	-6.05013950125486	5.02390824148909	0.88425711912064
H	-0.69908774375845	-1.08341127790288	-2.55906864400499
H	-2.84714438068471	-2.43601698177901	-4.08373368205247
H	-2.80172907541099	2.56331387469988	-1.81722581678012
H	-5.32317553239888	-2.39553004532417	-4.17987390437762
H	-6.59598682588326	-0.46201618857759	-3.33438447672003
H	-5.41548388905946	1.46536911959028	-2.29288782081412
H	-0.81614460430884	-0.05175249560345	-3.99006412716761
H	-0.34822248610149	1.71367620137099	-2.38024565271203

H	-2.06344909142930	0.02784019905795	-0.30438994853777

17

indene r2scan-3c

C	0.74657117467959	-3.84203862943711	-4.83828857057799
C	-0.50823594293283	-4.44705222775399	-4.86882644034251
C	1.41673007645074	-3.75827015465356	-3.62007699343664
C	2.71759885275589	-3.19466908924953	-3.27296836173177
C	-1.08345211497701	-4.95878466759079	-3.70548909899827
C	0.83407516821475	-4.27560660800116	-2.44678182249555
C	-0.41256502944897	-4.87556434487968	-2.48260608823772
C	2.94118785268543	-3.34898363256389	-1.95508062762253
C	1.77926950269422	-4.04517230469423	-1.29841515839066
H	3.82468697485336	-3.02686798565833	-1.41614062564063
H	2.08459417609727	-4.98663662955055	-0.81952752119001
H	1.19091214269713	-3.44478211344368	-5.74707859620541
H	-1.04644155728810	-4.52176293899445	-5.80948767065806
H	-2.06262678545072	-5.42643005684559	-3.75132809172164
H	3.39108965533631	-2.72570465361786	-3.98229128744940
H	-0.86691964273315	-5.27668208289551	-1.57971378938053
H	1.32762549636607	-3.42811188017006	-0.50828925592065

19

naphtalene protonated r2scan-3c

C	2.43482115121244	0.71950262176302	-0.00011295220926
C	1.22707890841556	1.41011926279221	-0.00015453994561
C	0.02949804304453	0.71031693032932	-0.00008654472040
C	0.06191806246962	-0.71839964314191	0.00006360533180
C	1.30566387401217	-1.40160489642689	0.00010091300510
C	2.47760374847670	-0.68484367275200	0.00001687938207
H	-1.35546724194867	2.09679161040358	0.85898169559216
H	3.36557517685799	1.27948499614581	-0.00018192098907
H	1.22550440893947	2.49688903026410	-0.00025092703771
C	-1.28764165158836	1.40264672880360	0.00002154857403
C	-1.14704246539166	-1.42806875863575	0.00007073744473

H	1.31497436320417	-2.48782170850366	0.00019421224592
H	3.43495637647780	-1.19531619275586	0.00004523996723
C	-2.48619758790345	0.55646204945579	-0.00019322344371
H	-1.10313147113538	-2.51612101480873	0.00019305258873
H	-1.35541935223096	2.09748087793576	-0.85836711317023
H	-3.45389636292742	1.05310340792433	-0.00030394856259
C	-2.40925735320590	-0.80574814256054	-0.00008491562766
H	-3.30764662677865	-1.41413848623219	-0.00015279842552

XYZ Structures (pyrrole -> pyridine pathway)

10

pyrrole r2scan-3c

C	-1.37375187399329	-0.44066518034644	-0.00012788989903
C	0.00252372997984	-0.43326785783618	0.00031786787809
C	0.42232253951822	0.92429104243762	-0.00034306974713
C	-0.70944917358980	1.70734192519564	0.00010372526659
N	-1.79475948438497	0.86623005735828	-0.00011542935385
H	-2.75492698752127	1.16317409536536	-0.00023623086120
H	-2.08019444114155	-1.25762442384931	-0.00021301141005
H	0.63761494065597	-1.30722642767113	0.00058780196762
H	1.43987663576461	1.28719290828960	-0.00057891084273
H	-0.83129548528776	2.78049236105656	0.00020314700168

115

B+pyrrole r2scan-3c

C	1.39944630611447	0.18114786923059	5.30301889238660
H	2.04094193556424	-5.68088004190090	7.93375832851998
H	1.46588381471310	0.05024528231356	6.39227392306047
C	2.01499332960670	-4.82473773993598	7.26682232562746
H	-0.01217814934083	-5.20011077603860	6.64578352097903
C	0.85634518487239	-4.55632756230032	6.54448961874163
C	3.14435273849674	-4.01256965393484	7.15095153265500
H	4.03703493545499	-4.24020023885995	7.72431515222865
H	-1.84292395177604	-4.40949201470933	5.22640043783173
C	0.82132480637854	-3.45423180317936	5.69215179067063
C	-1.54671365330616	-3.51741758065162	4.68279185355762
C	3.13696067001299	-2.91039641450606	6.29870740238264
C	-0.26667546582630	-2.98761537030764	4.84163421080187
C	1.97216827249084	-2.65995525926231	5.59623157482774
C	-2.44486878306360	-2.88828087397835	3.82826808093548
H	-3.44274054509567	-3.29862891888428	3.70910923788599
H	4.01250682335649	-2.27807777909856	6.18759002712218
C	0.05685258370607	-1.84271300210710	4.10577322501048

C	-2.09121083470788	-1.73180327379918	3.13085382490866
S	1.76109279818844	-1.33776289717854	4.40636482186319
C	-0.81820560655505	-1.18461961573246	3.26460297921673
H	-2.81271657156784	-1.25132769855504	2.47794950977853
H	-0.51876445208398	-0.28284291159142	2.74166270157371
C	-1.72601470100787	5.52412901581165	8.01436972515992
C	-0.39252557570727	5.56782349185245	8.40770467464316
C	0.61678498750295	5.75679836559767	7.46121680156298
C	-2.06714475714090	5.64871790832691	6.67154068114154
C	-1.07539621248838	5.83951420830583	5.70702034400076
C	0.25479806090289	5.91446086869508	6.12226396723866
H	-2.50555294986239	5.39968056899760	8.76041337078286
H	-0.13493488415138	5.47241101063520	9.45974610033179
H	1.02854019117157	6.11077700035325	5.38522265250357
H	-3.11233591453611	5.61706407722122	6.37337589489660
C	2.07310423700443	5.75882610159522	7.84762626267614
C	2.74341214649988	4.35302307240746	7.79171731447565
H	2.19523040421766	6.12591917461629	8.87386163238387
H	2.63082079177053	6.43163725070982	7.18771634965639
C	2.13616902294679	3.41316822950849	8.83394474927679
C	4.25368905308592	4.51082804678486	8.04469183097909
H	4.41299459967823	4.92793300703039	9.04451504866020
H	4.71262445813299	5.17579266574708	7.30856954818842
H	4.75784743101021	3.53902807390318	8.00184214593434
H	2.26308982483952	3.85157558355806	9.82948594205093
H	2.63732916446351	2.44096282706573	8.82343762588954
H	1.07015123484985	3.24998998543346	8.65973526930120
C	-1.40530774461157	5.93600355446645	4.23976252114811
C	-1.39652995446311	4.56736411776924	3.49415024959549
H	-0.69100554711272	6.60126806371630	3.74299350967546
H	-2.40557594120659	6.36401921916149	4.10051786631278
C	-0.02627877073223	3.92417842776781	3.68337129415043
C	-1.57873591293700	4.82072532606639	1.98629354290994
C	-2.50787025415980	3.65199668608667	4.00968834500869
H	-0.78974492881067	5.46738567339801	1.59383332553071

H	-2.54683704515029	5.30228641187152	1.81419370744599
H	-1.56771418320341	3.87751093527769	1.42914245285699
H	-2.38691664683114	3.42726911399807	5.07190098663104
H	-2.52267568571890	2.70573478152425	3.46105027954449
H	-3.47468035482243	4.14589643440109	3.86625980532037
O	0.99411406567010	4.57864859392749	3.31745536774236
O	0.00788440576724	2.74500858706849	4.17907654459769
C	2.57397077089128	3.79397439552408	6.37919819072833
O	2.01136562685447	2.65252292944811	6.26586495553708
O	3.02085673958542	4.49354783073215	5.42109479762404
H	6.47848914202813	2.16459107269613	6.28596255623749
H	7.03269282008012	3.08048228930198	4.86938614239319
C	6.94546427717692	2.07976344178643	5.29887488542365
H	7.94999313136428	1.66562302309556	5.43444830133794
O	3.73676687901826	1.06857824131214	4.50836768971960
O	4.70213168613467	2.95915588585747	3.73753960319750
C	4.74859086219990	1.77892764333046	4.18991047142162
C	6.13040112562474	1.15074942173564	4.38283039112938
H	5.54923326107473	-0.17798317729762	6.00015411626989
H	7.02416904133547	2.09828478724634	2.65369738004197
C	6.02055791644784	-0.23825431338836	5.01388146005887
H	7.79917861261847	0.58878045595516	3.15000288653065
C	6.82880879489855	1.07507427806506	2.99180347954567
H	7.02457121923484	-0.65718825679886	5.14032913752543
H	5.43879135972231	-0.91892057919303	4.38590295857941
H	6.90487800069621	-1.59568294570877	2.32005211447504
C	6.03440034326201	0.33963528338921	1.94477759764966
C	6.17016776957040	-1.03632615861766	1.74559128640553
H	5.03424056937546	2.11761336648463	1.27984769563768
C	5.11710889202665	1.04066366801477	1.16125173028386
C	5.39517188382720	-1.68721032239213	0.79067308707617
H	1.76249836420466	-0.82085701941570	0.71285218151702
H	5.52518455111591	-2.75269005230018	0.62194813625443
C	4.31189877074440	0.39890543733038	0.21929845577233
O	1.61123183634100	1.09557994141202	2.49175728983948

C	4.46456772966900	-0.97761972838035	0.03700349256819
C	1.21530669970108	-0.09464740569658	0.10719631902032
C	2.06167406201501	1.81240880051728	1.52970289543546
O	2.62069596870666	2.93901436336552	1.66013356628691
H	0.19365154759479	-0.02724253104355	0.49255989121655
C	1.89332896946207	1.27564021788083	0.10791540003310
C	3.29971949310486	1.19909537476027	-0.55859327568642
H	1.16064270109098	-0.47035115053649	-0.92017394312875
H	3.87096488849038	-1.49365074600032	-0.71411797920439
H	3.66897975661296	2.22099604713157	-0.69400796619977
H	3.15476967089707	0.76658011800498	-1.55603420571860
C	1.03163965651256	2.28538765796964	-0.67128526009783
H	0.03557606854999	2.37533238940444	-0.22427596408576
H	1.49698937859147	3.27401425087428	-0.68517535632209
H	0.90875171997010	1.93912686794882	-1.70282495197223
Rh	2.85227444960927	3.77409362716566	3.51946342215104
Rh	1.79047625080621	1.81635068585669	4.40957913426186
C	2.46803059534186	-4.57565439352834	2.84675748205683
C	2.24426273260653	-3.52293610078306	1.98390658630529
C	0.96185300979094	-3.71941555161816	1.39788097243695
C	0.44238143891969	-4.88055561523595	1.91990122260438
N	1.36494110545683	-5.38876861228690	2.80242939092489
H	1.26740093391749	-6.25462343831775	3.30525734591695
H	3.31922282832371	-4.81684390848117	3.46723877721834
H	2.94513846642545	-2.72751063508904	1.76864144343271
H	0.47727470119563	-3.08566579063420	0.66915486141748
H	-0.49525601131037	-5.38401654654413	1.73554413814313

115

TS2 r2scan-3c

C	-1.23279989018479	4.60742999529018	8.42950975426314
C	0.12147409359173	4.76676552827700	8.70577395247338
C	0.99101785322922	5.24725489466634	7.72242907276258
C	-1.73647314562785	4.91797819183545	7.16940760762889

C	-0.88705841023230	5.39839110162587	6.16905090473409
C	0.46742808225672	5.56476055457035	6.46821586138879
H	-1.90475684779737	4.25358188177626	9.20603183923275
H	0.49855974743735	4.55002057895720	9.70247808665298
H	1.12930165083016	5.96934571319257	5.70655767957108
H	-2.80126824451431	4.81614066295458	6.97427302487697
C	2.46567553954338	5.42641135628539	7.97652204760631
C	3.33348520594482	4.15917333530075	7.69853938204045
H	2.64307120935975	5.70976284866220	9.02125558892381
H	2.84417374792679	6.23894448939206	7.34743450556720
C	3.09075064751296	3.08694211176284	8.75982586689472
C	4.81671068606712	4.56376278944595	7.69890146071168
H	5.07838807031969	4.98106672699201	8.67684366384023
H	5.02484202467826	5.31173142116530	6.93060610741347
H	5.45799472644587	3.69391184987324	7.52022476083161
H	3.39345069498187	3.47346585512532	9.73861953157094
H	3.67650531545530	2.18672775290702	8.55041372380481
H	2.03815511189854	2.80398361793830	8.81275822565927
C	-1.38488695341229	5.73164797691769	4.78584362465476
C	-1.38110154417964	4.53056827329836	3.78769488496133
H	-0.76043216303327	6.52488760185941	4.36173904445130
H	-2.41418875365310	6.10812729267896	4.82791541185949
C	-0.00280647185591	3.87014199278365	3.87243111854349
C	-1.57205066041569	5.07262047352113	2.36113390047406
C	-2.50610997858222	3.54753000988888	4.11210046779806
H	-0.76505684985943	5.75438120687708	2.08440694686671
H	-2.52396475127638	5.61048964781270	2.30289627746060
H	-1.60173616895504	4.25444529291331	1.63359436875711
H	-2.49220912845207	3.23028886459679	5.15702500986063
H	-2.44702382368292	2.65749187727832	3.47850646058084
H	-3.46958162954234	4.03460794492051	3.92980975278204

O	0.97747685163603	4.55982471841958	3.47170939848560
O	0.06790737425664	2.68356834324261	4.35078736348254
C	2.97089185090024	3.65435138841819	6.30184288367605
O	2.39264995688913	2.51941086050419	6.21708302734807
O	3.25821171261514	4.40513737864621	5.32421101688796
H	6.71683615298406	1.81047955404632	5.78348236288498
H	7.09169821877857	2.85271528135520	4.39344460610190
C	7.06021991915052	1.81801186724611	4.74322827848983
H	8.07471682720480	1.40676740813247	4.72071860299180
O	3.77523095890446	0.88984999138708	4.27082578184159
O	4.66507598859595	2.80314998231011	3.46927803970049
C	4.75582954229941	1.60751377911100	3.86193789788898
C	6.13993301617093	0.95923055387275	3.85798217090453
H	5.71610847170687	-0.48804272878889	5.42602640780246
H	6.86091841881255	2.02704485207157	2.11682500412968
C	6.08792314339864	-0.47093009335449	4.39736462025443
H	7.65382170737358	0.47526184462835	2.41069121289554
C	6.68216958683195	0.98364619136528	2.39738458679329
H	7.09783977266180	-0.89429468429019	4.39434823086137
H	5.44666587747701	-1.11470735055959	3.78920896235585
H	6.66713804746942	-1.61695002706411	1.49183879072901
C	5.77059466390767	0.34012366083947	1.38431969310522
C	5.88400518161880	-1.01021821522344	1.04370912055109
H	4.71822149695194	2.16571422660754	0.98568521703124
C	4.78251272330052	1.10475582681577	0.76115535387974
C	5.02577279750032	-1.57056815091427	0.10259905543089
H	1.42376043861274	-0.66559810151639	0.51154437154790
H	5.14693774747759	-2.61208564267480	-0.18288775480168
C	3.89132738492083	0.55065187107736	-0.15989256424719
O	1.53912861302370	1.14509917777647	2.43793829469833
C	4.02952338345142	-0.79983659594740	-0.49024522888113

C	0.79486700057292	0.10761091394823	0.06608693245868
C	1.83882977825445	1.92446680110614	1.47155780971560
O	2.40138763322269	3.05181108040782	1.59570921616946
H	-0.13895594028624	0.14781076353588	0.63398565655720
C	1.49273984911250	1.46671039785061	0.05568607114415
C	2.81500004243112	1.41191118870261	-0.76822541370061
H	0.55912001318486	-0.18447567201340	-0.96280328333511
H	3.37474509631121	-1.24059293971472	-1.23900211660427
H	3.18743105874413	2.43522065096985	-0.88390091037726
H	2.55095647984488	1.04207304284101	-1.76631781907899
C	0.56558783847237	2.52567734837571	-0.56641880657493
H	-0.37909343588448	2.58502532070452	-0.01553394769904
H	1.03311363224563	3.51341744857816	-0.56698619047360
H	0.33571315621708	2.24395073044119	-1.59924185099350
Rh	2.83748601026100	3.73633035083644	3.45629915472343
Rh	1.90323014556107	1.71048363435695	4.37549233092064
C	-1.91636344511748	-0.54969536837564	7.14292890794565
C	1.22288722576729	0.12809419058562	5.24964471532725
H	-0.07389284215848	-5.17738735278392	8.68637468820255
H	0.22284132759311	-0.16038463863676	5.60297805359337
C	0.40528543077322	-4.37887731076229	8.12867702386413
H	0.09956584843261	-5.34224811298611	6.22852247419862
C	0.49541106672095	-4.47265667462674	6.74377329289224
C	0.94087277855366	-3.28877453929779	8.81543555001025
H	0.87831548951887	-3.24722995762340	9.89819684392711
H	0.55219046577155	-5.20746464707053	3.84941628498594
C	1.12106862277196	-3.45199724958755	6.03017374335012
C	1.10543574230183	-4.30494480514506	3.60847485377685
C	1.56179556377817	-2.25300038917570	8.12263022399952
C	1.40732094079506	-3.37808060214163	4.60412501995181
C	1.62018859609956	-2.35391328637300	6.74440168123834

C	1.53036443677486	-4.06870388276011	2.30578502358515
H	1.29972770969473	-4.79296829040752	1.53078450427319
H	1.98010593495498	-1.39922862833800	8.64562540242060
C	2.13548856545445	-2.23447299860239	4.23809988314324
C	2.25678256986327	-2.92374906625313	1.98108088905176
S	2.43396588340321	-1.17551545099792	5.67403649847889
C	2.56777720748184	-1.97208722083736	2.95102005001305
H	2.60043443135872	-2.76341373016741	0.96516635602255
H	3.12647620799942	-1.07563800511834	2.70171431092519
N	-1.09198872666493	1.47283634671967	6.73193675124653
C	-0.45890726360557	1.01548100197656	7.85732679723264
C	-1.96361416342899	0.52906603414370	6.27222752638586
C	-0.97488570799883	-0.23558870844485	8.14854111695145
H	-0.72132087085930	-0.84037740435217	9.00543969220130
H	-2.55067286233556	0.69018066207417	5.37990333320378
H	-2.51558924466700	-1.44586055654120	7.07012651955775
H	0.25475786715927	1.63519325779037	8.37796528748784
H	-0.84167796355838	2.31767044230574	6.23654014538154

115

C_exo r2scan-3c

C	-1.50539073988904	4.43059735508843	8.28890239013121
C	-0.19379862710585	4.80939430454100	8.56905366809178
C	0.62785563177072	5.30989260127412	7.55369454907991
C	-2.01073900871326	4.53710926854261	6.99561969238292
C	-1.20971364026342	5.03913666330839	5.96556092761254
C	0.09887350272203	5.42191511893863	6.26606428885432
H	-2.15264259397451	4.08626658284168	9.09196504275622
H	0.17882907026873	4.76557708410123	9.59043061091482
H	0.71904734669511	5.83878930616092	5.47619193635275
H	-3.04546388905126	4.27043062647474	6.79506566525297

C	2.05138643524918	5.73256031176120	7.80498025803169
C	3.10365013021479	4.59541697479089	7.62341059682932
H	2.16233985253977	6.11907473330033	8.82549335814670
H	2.30881079511411	6.54283177140459	7.11477161017541
C	3.00336213498657	3.57891013917950	8.76129207464305
C	4.50663269192071	5.22115541167688	7.60969123876954
H	4.68081381270972	5.75410837815075	8.55045020134384
H	4.61593662886765	5.92354459866781	6.78055475315576
H	5.27611243877408	4.44826212959059	7.51021886355564
H	3.23477186003099	4.07180932835740	9.71160705119952
H	3.71170072032515	2.75793340088992	8.61698768463256
H	2.00044017000357	3.15001045461637	8.83276523407278
C	-1.71458991222936	5.18826387911368	4.55401502928427
C	-1.51140746320988	3.92988986116290	3.65632578534273
H	-1.20030752343248	6.02968321968516	4.07791779804047
H	-2.78781987477108	5.41509458285772	4.55491028324436
C	-0.04515130987501	3.50539348176017	3.76992001602703
C	-1.80397116185284	4.31127716660451	2.19738147126548
C	-2.44639342223301	2.80136573405492	4.09646541571180
H	-1.13435850186390	5.10375789416430	1.85569544032537
H	-2.83869089083402	4.65822550287840	2.10579169609375
H	-1.67644484872179	3.44660896069331	1.53650429520121
H	-2.28662820016085	2.52250651240619	5.14154814137160
H	-2.29494519896991	1.90739997401509	3.48381815778625
H	-3.48685289109552	3.12355560034894	3.98013337807548
O	0.83012184555226	4.27976608497910	3.29568928178448
O	0.18615383358184	2.39083970068540	4.36095743844696
C	2.84107131934682	3.93683177406997	6.26654959453755
O	2.39533605057505	2.73629215677990	6.27670982216692
O	3.05961845717357	4.63069488016318	5.23580214591352
H	6.67050655354679	2.53701877642422	6.19202857413052

H	7.03485109108439	3.58093122203668	4.79965993216497
C	7.12147357648244	2.56489258471322	5.19361098079106
H	8.18302992169640	2.31567652891171	5.29678875089815
O	4.08838065625911	1.17052905368665	4.57235858779069
O	4.75373357096774	3.09640009702175	3.58278743595466
C	4.97516444004690	1.96880878038385	4.11960137301010
C	6.44051452304003	1.54784859935515	4.25991449926709
H	6.11812592478796	0.10379562955796	5.85241755026758
H	7.12659552909897	2.66648124878724	2.53795472849797
C	6.55772750031846	0.14246045393614	4.85149799648989
H	8.16490026771607	1.31010089022259	2.99470341435612
C	7.12112026024460	1.62035258170134	2.86143769416695
H	7.61604983683285	-0.12940153634626	4.93099368545569
H	6.05546211525168	-0.60011903256996	4.22599957459633
H	7.70120237854256	-0.96118118060688	2.11275734879225
C	6.45385271939058	0.76880769828248	1.81289579648552
C	6.85450562886991	-0.54685551562649	1.57047613298761
H	5.09298131113404	2.32158953429199	1.23330615730164
C	5.38644788273269	1.28766645594125	1.07857383126465
C	6.19081511251523	-1.32002965367673	0.62282481725468
H	2.56621363469040	-1.11394676711437	0.96945105660441
H	6.52584517168740	-2.33372427175543	0.42177745436425
C	4.68749047252553	0.51477800422563	0.15114871391077
O	1.97231938750072	0.87485099994718	2.62745774996906
C	5.10809049926556	-0.79751983893614	-0.07729104781410
C	1.82666176493106	-0.59307926446327	0.35594074829857
C	2.26377161844013	1.57223685506008	1.59394473276809
O	2.61214005762903	2.78712687062404	1.60696184350298
H	0.83895113531229	-0.74929049947984	0.79844639431253
C	2.15016260480713	0.89594211077879	0.22439663405924
C	3.48690787809962	1.09624892126061	-0.54931281101498

H	1.82214736842808	-1.05029272163001	-0.63956661609030
H	4.59736096264057	-1.40760392727748	-0.81867066119916
H	3.63433092228235	2.16928921556309	-0.70892880623848
H	3.35859367883406	0.62981516915895	-1.53403915626039
C	1.02335618088657	1.61132027641170	-0.54282385862281
H	0.06556313277837	1.50353146379776	-0.01976460933449
H	1.23879009604249	2.67710842307395	-0.65554449523887
H	0.91452373226620	1.16580544805763	-1.53741522896064
Rh	2.83153522304302	3.76779085764725	3.39033377711579
Rh	2.12553254626978	1.73616731863824	4.49096666641424
C	-0.05774604201594	-0.92086252401885	7.40540075061065
C	1.58877734911085	0.01966673751705	5.62477447086626
H	0.95463056118094	-6.39219475414318	7.03592077097915
H	2.40293453021542	-0.13985704040958	6.34723218228013
C	1.12625029853843	-5.42845382621570	6.56694936293339
H	-0.77003259960357	-5.43769286998989	5.55003560707890
C	0.15120228266809	-4.89224592357957	5.73148957834156
C	2.32456408454559	-4.75293547095406	6.80555997941151
H	3.07508092790201	-5.19399333751792	7.45347846929364
H	-2.23472952033713	-4.15810596646240	4.09254820832017
C	0.37483117918551	-3.65442558253899	5.12820330635384
C	-1.78605532913549	-3.20892926991123	3.81606892384796
C	2.57202742382920	-3.52012541508567	6.20540301758977
C	-0.50234442747052	-2.88439249125825	4.25460853591910
C	1.58333885936977	-2.99625888869047	5.39026422068297
C	-2.48688832929727	-2.30742044309999	3.02295997449376
H	-3.48482368857970	-2.56115717880706	2.67947863720659
H	3.50759768313831	-2.99382400132782	6.37123826060143
C	0.04458470742283	-1.65157659716517	3.86313176333294
C	-1.92577810632570	-1.08153809588321	2.66631872862702
S	1.71020935940943	-1.43307586020288	4.50434945304279

C	-0.64018716679103	-0.73658271718823	3.08071438021979
H	-2.48904884846123	-0.38804810623717	2.04981373384373
H	-0.18594904859728	0.20940658052012	2.80903456872844
N	0.11895192670714	1.33286247682746	7.11599016864383
C	-0.11971237676632	1.09804480058582	8.37787773353896
C	0.20794213569681	0.09431675175644	6.35364706883917
C	-0.22853505354602	-0.30863676832714	8.60568517132720
H	-0.41444900511709	-0.76690215290202	9.56692498666969
H	-0.57747173018178	0.10187160758113	5.57906715476247
H	-0.09226265518710	-1.98417956392521	7.20774570544001
H	-0.21423266740985	1.91351503130117	9.08698562859777
H	0.30287309030685	2.24478630325492	6.70050924639927

115

C_endo r2scan-3c

C	0.46656714125367	-1.13930001585480	7.58006158597728
C	1.90348953298382	-0.17766672580734	5.55307030528476
H	0.61249048529217	-6.32126491754192	7.32834951092708
H	2.89703729258480	-0.36590397281965	5.98408029135774
C	0.85185565356334	-5.41572181023100	6.77979068335581
H	-1.15079229273566	-5.18166784806449	6.02869133210891
C	-0.14436743572206	-4.77430926064155	6.05043698599030
C	2.15793225477906	-4.92157366406870	6.80821874274713
H	2.92264454597910	-5.44531534627206	7.37271961717817
H	-2.61033686570430	-3.76115804815433	4.68999608675294
C	0.16738404372676	-3.61338162426649	5.34391754742933
C	-2.06573657071029	-2.91294821638401	4.28615961227828
C	2.49237132717352	-3.76887574203983	6.10155809053240
C	-0.70319871332439	-2.76345649977885	4.54184855864572
C	1.48335721472077	-3.13562406795176	5.39462556452327
C	-2.71932802992710	-1.97612067144648	3.49081194105963

H	-3.77730732482170	-2.09974358321934	3.28074647266465
H	3.50894805270654	-3.38595563298092	6.10404327904583
C	-0.04305987122332	-1.65141961345986	3.99610440567338
C	-2.02968565189514	-0.89452582991886	2.94252332599536
S	1.69476904920465	-1.62777276835461	4.43988192338449
C	-0.66880478483954	-0.71812880203495	3.18849207415457
H	-2.55274390363027	-0.18823830849273	2.30528483176695
H	-0.11441141344899	0.11798376664084	2.77682681868895
N	-0.40155141670242	0.51812267334471	6.26441539001811
C	-1.38881899573532	-0.13402735005180	6.81513263814975
C	0.88201608021955	-0.02037793299333	6.69546893347799
C	-0.88907500872748	-1.18297514285153	7.64933362134937
H	-1.50311761849415	-1.86710927694280	8.21838074226697
H	1.37091822175699	0.76338111548724	7.31309353410309
H	1.16858262817190	-1.77575237044342	8.10332531234133
H	-2.42254723979169	0.13271091614183	6.62020308363868
H	-0.44773524226474	1.28447729485481	5.56652698285221
C	-1.73588140570150	4.95680070644766	8.13880860088418
C	-0.39959024429838	5.19947411504981	8.44173751550511
C	0.51732994084006	5.47285696022287	7.42383834013771
C	-2.16904550953442	4.96421030144391	6.81632253960293
C	-1.26869833944347	5.22747854185775	5.78039218543885
C	0.06094855866008	5.50052374335509	6.10478800371556
H	-2.44787213961520	4.77965206073600	8.94012256010059
H	-0.07180688822684	5.20336845709888	9.47859814038879
H	0.76006022498353	5.74817051119081	5.31125382222472
H	-3.21861489381696	4.79098966370312	6.58889217907642
C	1.97498962983967	5.71883831729428	7.71439486605569
C	2.86018657054073	4.43831871476897	7.67282468486493
H	2.09272629832359	6.15988168760656	8.71185130206035
H	2.37695239427215	6.43334555514165	6.98861015496824

C	2.46970269114248	3.47006120504196	8.79049532406474
C	4.33337003675170	4.85443322246488	7.83457098547306
H	4.46914428418033	5.35630195039128	8.79845191448168
H	4.63802859194748	5.53618278577044	7.03653094077372
H	4.99056525192371	3.97834350546352	7.81328108153504
H	2.58288160738629	3.96890829958942	9.75914365723507
H	3.11604721585007	2.58710571567388	8.78711502954515
H	1.43044114050736	3.14194185024973	8.69543231370549
C	-1.69194866805957	5.18655454477033	4.33451673451213
C	-1.51521304341080	3.78748032441630	3.67436056676168
H	-1.11106934833640	5.91189284406838	3.75571547429811
H	-2.75047237017692	5.45820464279052	4.23749146848687
C	-0.04376368869256	3.37856037368539	3.77675378635951
C	-1.85254242008049	3.89250783474173	2.17626463149711
C	-2.43462370818648	2.76263074431493	4.34073487099737
H	-1.19726760558849	4.61148766833126	1.67857067559650
H	-2.89137545781323	4.21635858752598	2.05298350733310
H	-1.73777461891244	2.92161576282955	1.67992569277007
H	-2.25175343705260	2.70587405744714	5.41927522331680
H	-2.30815793637147	1.76842133556444	3.90081102419310
H	-3.47823326850691	3.06442336563391	4.20024323788718
O	0.81135498237056	4.16761971526845	3.30335228480735
O	0.22874060074124	2.23850870954303	4.31030627963345
C	2.71545606815746	3.78635090149943	6.29612945509158
O	2.36476360150767	2.55601736673023	6.25955164668934
O	2.96696624828450	4.50610113287536	5.28793803979107
H	6.75834901298203	2.65877935536138	6.10620000336024
H	7.05861524420653	3.70989595111117	4.70564946339274
C	7.18969172279082	2.69891263225605	5.09988701000673
H	8.26128019436498	2.48855191111781	5.18319767776165
O	4.20090719552328	1.16665468769801	4.50723746992190

O	4.76713817976705	3.14233842188799	3.56073379125669
C	5.04727420850307	2.01534836107759	4.06744172076210
C	6.53048688635089	1.65554761053493	4.18000008890095
H	6.30221906384928	0.19583431905718	5.77529364396694
H	7.13706759473691	2.80644954962150	2.45004686897648
C	6.71736150034605	0.25504557917509	4.76483086224784
H	8.23440224708851	1.48786566351929	2.87837674758100
C	7.17729533498052	1.75905834170579	2.76682312288219
H	7.78744690101241	0.02729824969947	4.82135111475808
H	6.23312711077338	-0.50670439578565	4.14838789969468
H	7.81868900509892	-0.80663484188811	2.01352718039834
C	6.52227395497467	0.88897296626532	1.72604642376309
C	6.95588723263300	-0.41557950910178	1.47949979611828
H	5.11603869089579	2.40544402632709	1.15835972956957
C	5.43552559615586	1.37944365860345	1.00082913470194
C	6.30479468287128	-1.20619382641796	0.53765957877433
H	2.65950566907226	-1.12288275582701	0.82969322903556
H	6.66498870107398	-2.21056446745435	0.33338720585905
C	4.75076541165513	0.58881030005001	0.07737428297887
O	2.08603504571729	0.80140857204959	2.55942530815937
C	5.20331866829728	-0.71219134440475	-0.15404236526845
C	1.89599888544348	-0.59070387894311	0.25689141901071
C	2.32559621165745	1.54344215938763	1.54425376335053
O	2.63100858977384	2.77002436495080	1.58769131451483
H	0.92538151561049	-0.76762175941833	0.73030110544432
C	2.20368905228117	0.90441100365682	0.15838601160420
C	3.53317012239196	1.14046796117090	-0.61795356369793
H	1.85862818938740	-1.01886115922587	-0.75077243906625
H	4.70359204865871	-1.33477549837745	-0.89254336373892
H	3.65260344405530	2.21785217693039	-0.77241317070818
H	3.41487087654612	0.67601125316177	-1.60486063345982

C	1.06396624223332	1.62679724806339	-0.58192093620037
H	0.11032487385426	1.49175013235170	-0.05764478126897
H	1.26486486928614	2.69808175869809	-0.66435179190623
H	0.95541753895646	1.20919180863327	-1.58856057225969
Rh	2.81673849005544	3.71259477422353	3.40035860860583
Rh	2.21300881178332	1.61760599289631	4.44041816203413

115

TS3_endo r2scan-3c

C	-1.66771929323539	5.45919900229964	8.06035367318751
C	-0.31753521245704	5.70177970339188	8.29281776057757
C	0.55683341456624	5.91135493001884	7.22355672072028
C	-2.15845125257564	5.40541136089774	6.75925964531916
C	-1.30225241845112	5.60676336152155	5.67376335539415
C	0.04325708331317	5.87824816983056	5.92594619075615
H	-2.34519157428891	5.32754347102104	8.89927362920012
H	0.05483331775048	5.75446721268349	9.31310368207214
H	0.70789297132339	6.07679133380875	5.08991801180509
H	-3.21855864775871	5.23250652850169	6.58783743161331
C	2.02869996885902	6.15149864111990	7.43756449000806
C	2.89370782546335	4.85601225278542	7.44235970061494
H	2.19534840018266	6.65206112427408	8.39932028731269
H	2.40923109097926	6.81256448679033	6.65191238728400
C	2.55194385295892	3.97636040217758	8.64554949069295
C	4.37913734128155	5.25627616966401	7.49280515931884
H	4.57397235399210	5.82284464763097	8.40940534348046
H	4.64975613879964	5.87455699154715	6.63319422649985
H	5.02263476440354	4.36979520255784	7.50021400592360
H	2.73747652014309	4.53688923053517	9.56798652399833
H	3.17420065975481	3.07668289201879	8.66336413400376
H	1.50217501963492	3.67029942720638	8.63768020194524

C	-1.78806275280910	5.50822640379238	4.25058249772367
C	-1.67469910295371	4.07657752760041	3.64633398846391
H	-1.21411991500792	6.19109677279440	3.61565756434614
H	-2.84242833982898	5.80392659464799	4.18381640299373
C	-0.21846213896116	3.61572988953218	3.75042147737162
C	-2.03775411767853	4.13400882745749	2.15255127767375
C	-2.61849581690608	3.11601607612799	4.37146825666558
H	-1.37119921840878	4.81172151377909	1.61390508656303
H	-3.06855759721905	4.48474850409567	2.03530517277450
H	-1.96263162859389	3.14147276547086	1.69199513412499
H	-2.40164871591276	3.07991494762421	5.44404306895452
H	-2.55534664394348	2.10639285519374	3.94989238583464
H	-3.65152365030692	3.46062472093496	4.25366615598416
O	0.65053286694269	4.33947928428840	3.18577353349170
O	0.02129016213859	2.52506200338646	4.37763904766238
C	2.65919688388334	4.11061114989671	6.12882883032763
O	2.26158920029158	2.89764954305192	6.19302089651787
O	2.88867622673243	4.74370538083294	5.05660175111842
H	6.57281042246704	2.71170271225658	5.97225992827617
H	6.89235199426702	3.68543281928449	4.52030791097654
C	6.98833021481540	2.68873948245241	4.95875929922529
H	8.05154984970249	2.43789464781026	5.03564919000157
O	3.92718150518901	1.28053820578869	4.50754240473383
O	4.56364472574454	3.15399721137879	3.41323119479124
C	4.80482427938921	2.05074839333820	3.99224414871826
C	6.27116309880278	1.63170643375250	4.09915300642029
H	6.00344795788335	0.26553461650069	5.76933282496105
H	6.89865954920615	2.66421900576846	2.30129860072394
C	6.40379250343196	0.25495298928894	4.75134297506356
H	7.94656126856933	1.32585231848200	2.78554803595168
C	6.89988418698018	1.63458772284888	2.67403136786336

H	7.46303477992297	-0.01903073145320	4.80426915232183
H	5.87543703230773	-0.51282207103632	4.18035034751897
H	7.43914265908209	-0.98540030279748	2.03987159146491
C	6.19378426316878	0.73694783598084	1.69099371779755
C	6.57808605435050	-0.59276246609544	1.50419517543472
H	4.82765252282239	2.26962087235810	1.07055925165438
C	5.10795667449860	1.22598408689151	0.96314703164994
C	5.88012913609819	-1.40832939439463	0.61899170445248
H	2.26501336093424	-1.15339099355089	1.03943986542358
H	6.20233833942811	-2.43359524940854	0.46036949749135
C	4.37560428356371	0.41206378694250	0.09826949131612
O	1.76591588347118	0.91808234663010	2.63364156383003
C	4.77945707079873	-0.91390373928015	-0.07363529947879
C	1.49840948945657	-0.64298479474171	0.45056686255918
C	2.01729445941528	1.56485049102425	1.56164714884189
O	2.37042033507909	2.78045270778497	1.50890281202660
H	0.53970306447926	-0.74786803660999	0.96631349665516
C	1.84620594969929	0.82992257632173	0.23013652031040
C	3.15889102098739	0.96714011910945	-0.59619721173600
H	1.41734396488546	-1.14394522848008	-0.52020971446786
H	4.24219117901089	-1.55710520861072	-0.76671891917824
H	3.31282554726393	2.02730468936782	-0.82273144073969
H	2.99205351914459	0.44657379831851	-1.54728529457756
C	0.70475249535587	1.52866906695652	-0.53049430315415
H	-0.23407341691141	1.46339284156766	0.03241326883061
H	0.93308892612456	2.58365064861514	-0.70291298044217
H	0.55148199213959	1.03779298734776	-1.49740120091818
Rh	2.63417764472128	3.80522494055827	3.25799360870974
Rh	1.97104391624330	1.85310342634072	4.45597540513723
C	0.70277068045928	-0.55958548366154	7.48804291295615
C	1.45856817220432	-0.07581975651559	5.84809488390498

H	0.39931414618267	-6.57734305093267	6.32668047903627
H	2.50244695776895	0.05652063565864	6.12059480167334
C	0.59842560035579	-5.55284093208707	6.02842988075803
H	-1.23552700938174	-5.37964934038408	4.91614449302547
C	-0.32527889278977	-4.88068071481509	5.23450055570356
C	1.78076907880760	-4.93604437313955	6.44147572801366
H	2.49130822517095	-5.48210637947507	7.05352616596292
H	-2.60777815761891	-3.88480263098087	3.61786657913846
C	-0.06695514591930	-3.56322560981514	4.85611244130071
C	-2.15065640840746	-2.90533889439186	3.51641832730443
C	2.06371545563876	-3.62614116056739	6.05989837876581
C	-0.89553277360131	-2.65504428420543	4.07163103545303
C	1.11964394010965	-2.97112538154918	5.29238759273758
C	-2.81067595135914	-1.89366535930007	2.82908461932676
H	-3.78811079993696	-2.08868200393413	2.39894850485258
H	2.98814586217391	-3.14150892710394	6.36080522282942
C	-0.33851435526208	-1.37839763998205	3.90513814091447
C	-2.22811993177177	-0.63520236395673	2.67823153592903
S	1.32636806386873	-1.31129495418825	4.60638461784431
C	-0.96652886619494	-0.36483859595678	3.20493166761582
H	-2.75296551546568	0.14208840586844	2.13123421280646
H	-0.49819309264826	0.60380945634156	3.08417466491471
N	-0.98340042597783	0.48479609523848	6.37656165813481
C	-1.41677877324588	-0.75003594754738	6.74862669012707
C	0.36497351741194	0.65182777691418	6.74550899437217
C	-0.43693728619509	-1.42151479748336	7.43168129357507
H	-0.51287195072583	-2.41259151357305	7.85186255851920
H	0.70028855808837	1.65647869567849	6.99758426893422
H	1.53432155417667	-0.63712006314637	8.17325771256828
H	-2.41244843523957	-1.08579050997837	6.48491300001680
H	-1.44997919946450	1.12783696607281	5.75252453682236

115

TS3_exo r2scan-3c

C	0.29882312595543	-0.37762443716592	7.52999392472247
C	1.35823847315296	0.15943943675473	6.11754204867452
H	1.27901226708428	-6.29222598995327	7.27391935377465
H	2.29396607140378	0.44521711268383	6.58693241340852
C	1.35000194731785	-5.29448433398487	6.85241039714915
H	-0.60275325714889	-5.39281593303065	5.95264852941581
C	0.28655365972123	-4.79026407573568	6.11027545956865
C	2.50894937912845	-4.54443866622464	7.05917423578819
H	3.32797087427613	-4.96130784389029	7.63624010897141
H	-2.24276379421240	-4.15711392976714	4.66613922561728
C	0.38153124350798	-3.50739879567998	5.57085411510520
C	-1.87837267361145	-3.16940438245685	4.40128759087071
C	2.62825985280295	-3.26601796671393	6.51753570544182
C	-0.59936816261622	-2.76601506965832	4.78335796150335
C	1.55270459562804	-2.77865016429036	5.79799340406645
C	-2.68602390587952	-2.29559190814461	3.68251042632322
H	-3.68252775781015	-2.60952408672908	3.38817148175738
H	3.53127247019304	-2.67840189496476	6.65648766584885
C	-0.16810933309866	-1.48497889587192	4.40914223229645
C	-2.23441706218338	-1.02249037025012	3.33727244585350
S	1.52336506935590	-1.17173693081006	4.96174176896456
C	-0.95357183670505	-0.60058995438118	3.69026759687457
H	-2.87864575856554	-0.35189587561857	2.77747463830252
H	-0.58147675376675	0.37935310545377	3.41272185050855
N	0.04791251167942	1.87678381092311	7.41619968301400
C	0.49854892989155	1.55646358243047	8.65965890369063
C	-0.04129917835051	0.71442378699942	6.62395295945577
C	0.65856299245297	0.20321856684800	8.78751692959819
H	0.99084150512804	-0.32667295763108	9.66672291046165

H	-0.81074037888484	0.69097112903590	5.85931594732669
H	0.03193778192332	-1.41257049649599	7.36145179593157
H	0.67402386932775	2.33631482214868	9.38924737303610
H	-0.00717443178142	2.81583606628822	7.04174276628850
C	5.29336690316368	-1.90061541887082	0.63270936474433
C	4.20209483780028	-1.33647456716939	-0.02022171671916
C	3.93759336632658	0.02975823306661	0.10022259475269
C	6.12068119591340	-1.11336978616165	1.42740264089896
C	5.87600577736799	0.25510801630982	1.56224285837577
C	4.79673856781041	0.81079588215627	0.87351238020752
H	5.50686857195211	-2.95903692794901	0.51285842965905
H	3.56232782451036	-1.95764331077085	-0.64257710233864
H	4.62267070505406	1.88065610732804	0.93883560732429
H	6.97413645164857	-1.56113951783799	1.93107733592687
C	2.73729456374751	0.66492372747581	-0.55201010430029
C	1.46656228468804	0.67976757742221	0.34863301609216
H	2.47224639788309	0.12451210363722	-1.46915326279583
H	2.97115772865716	1.69691608765158	-0.83330532511750
C	1.00347907707320	-0.74607905044429	0.64945591902145
C	0.35135354173928	1.44845581391843	-0.38295291420039
H	0.10166937177220	0.93322817243408	-1.31648002555578
H	0.66447197654360	2.46930820553163	-0.61647793228705
H	-0.55659701413757	1.49509797472758	0.22986276978247
H	0.84671811151540	-1.28116789587597	-0.29347555902162
H	0.05704911176831	-0.74862090135914	1.19691739947948
H	1.74224801066672	-1.29584804087678	1.23848720244276
C	6.72293110733328	1.12695364957607	2.45243621639673
C	6.18695909906958	1.24889807715366	3.90958770758761
H	6.79025593700823	2.13375676044305	2.02722645365357
H	7.74242063294595	0.72748504606754	2.51830967808784
C	4.75882119601649	1.79395811787814	3.86865250588352

C	7.04869475119848	2.27285130861500	4.67042357862601
C	6.23746531449772	-0.10289277297568	4.62397636513154
H	7.01493852904827	3.25332599102531	4.18807121156220
H	8.08826560008960	1.92936915173866	4.69667991352025
H	6.70245427339323	2.37914213205081	5.70462606801067
H	5.60902501952220	-0.84476918732867	4.12409945875194
H	5.90241342428254	-0.00859044037269	5.66126195994748
H	7.26943408940156	-0.47037359407416	4.63077574450770
O	4.57596598306460	2.88231736402968	3.24119836989946
O	3.85615463887561	1.13477978156216	4.48374532952436
C	1.78133895747096	1.44978775447562	1.63365700783969
O	1.54952946894699	0.87676840054988	2.75134847520450
O	2.23373389316260	2.62512690637063	1.49791687851203
H	-1.99154344755216	3.71752159797860	1.75513659307373
H	-1.25036674981311	5.32224016259848	1.89528467488833
C	-1.95552891845890	4.63000050381575	2.36066218380260
H	-2.95266455053305	5.08260350844946	2.35045717933761
O	0.07579004991037	2.68327012898927	4.47968564520884
O	0.76462842552722	4.40889986796789	3.19241945384917
C	-0.12302287328338	3.75964880554683	3.81674081240688
C	-1.54875380227750	4.31268453614477	3.80749137157751
H	-2.52642505659632	2.36863591639466	3.85189911465385
H	-1.01451947528661	6.39654219801158	4.03744100556413
C	-2.52967078861624	3.30772680271875	4.41500464011164
H	-2.58800581295595	5.96688039099033	4.71851080819113
C	-1.54456565003574	5.64088708774795	4.62679925409918
H	-3.54312884049229	3.72134496405344	4.37889517678798
H	-2.28917312388297	3.07838498718385	5.45641389062877
H	-2.68351486244333	4.94175484612645	7.04786440829919
C	-0.90334463670444	5.54067531300636	5.98663129727731
C	-1.61894663848562	5.14939793222001	7.12252143163960

H	1.01048413510488	6.17566226028803	5.25364289531491
C	0.45094206212856	5.84962528301808	6.12709441597844
C	-0.98520354333482	5.07416257624043	8.36083067341595
H	2.46750424130906	3.61894831026566	8.68567331592724
H	-1.55875530172697	4.80338480987932	9.24332707977288
C	1.10513593813384	5.77060469138574	7.35802366580977
O	2.49403247052098	2.97616157136889	6.15900061547721
C	0.36860071637728	5.37992828969792	8.48027405383163
C	3.48155752340243	3.95858871416294	8.46677940827927
C	3.03589333037436	4.11962509785608	5.99227139915093
O	3.18784473686409	4.70389097073636	4.87882420905739
H	4.10793808084206	3.07407384814805	8.31686364391999
C	3.51412051879119	4.86644984763540	7.23802953893270
C	2.57361610611184	6.09639371939752	7.43454343903444
H	3.86047517195675	4.50701095096987	9.33575987451642
H	0.84496328558670	5.35857430542434	9.45793201140581
H	2.81724338847265	6.83429975606905	6.66296741542039
H	2.81967608054929	6.54109957126843	8.40650640886252
C	4.94549670930790	5.37179233672116	7.00245397488710
H	5.63834995920702	4.53247050292694	6.88110198130695
H	5.00289033021890	5.99633158179474	6.10830598914396
H	5.27360653498900	5.95960523853525	7.86627366342418
Rh	2.70239172274887	3.70474684780365	3.16438912020371
Rh	1.95729572243518	1.87984824331614	4.51039422579811

33

D_exo r2scan-3c

C	0.82917541340360	-0.60516105147700	7.65859955833361
C	1.33513107236558	-0.14115288071362	6.27662779125312
H	0.70308716059095	-6.53923228246420	6.66852762623522
H	2.15699050111426	0.56904184309882	6.23556910949405

C	0.86510979320054	-5.51489586077854	6.34832282242199
H	-1.03525907409032	-5.38904321378034	5.34529880936998
C	-0.11783111152372	-4.86971670143898	5.60403679104290
C	2.05618881681911	-4.87201857025201	6.68648138871139
H	2.81088659968617	-5.39548262549597	7.26411995738007
H	-2.51052194945772	-3.93516178379693	4.04090761699927
C	0.08952811443512	-3.55375438705718	5.19375480833578
C	-2.05568155599003	-2.96030024387342	3.89554267100024
C	2.29229905043409	-3.56064368495774	6.27733776455611
C	-0.79340232250563	-2.68412217403005	4.41877757195919
C	1.29595170789494	-2.93822860746371	5.54860229333212
C	-2.72794021222657	-1.97513937365617	3.17871410349860
H	-3.71095789359565	-2.19080183992190	2.77221952647979
H	3.21992160684668	-3.05393362151276	6.52573360835409
C	-0.25241054762553	-1.41291229899058	4.19043613903256
C	-2.15925407795617	-0.71912144138014	2.96571308469874
S	1.40016952241851	-1.26530663065491	4.89165018379233
C	-0.89317197491211	-0.42313912057304	3.46787047124579
H	-2.69770072927449	0.03131856906538	2.39632052797453
H	-0.43335255861047	0.54511602299495	3.29382864851092
N	-0.06637236670059	1.53989142688111	7.54086341986116
C	0.79584122512556	1.47113738087047	8.62487298025437
C	-0.03502370326934	0.31120138968017	6.83843482883843
C	1.30716417010036	0.23330567360306	8.77971437725776
H	1.92884547157081	-0.10899677638336	9.59299727328415
H	-0.92402238630794	-0.00799927200346	6.30138220543599
H	0.61314094182820	-1.66389110020685	7.77171150888736
H	0.95480956127728	2.34807765789285	9.24024245914557
H	-0.34526326506555	2.40703657877609	7.11212607302259

33

D_endo r2scan-3c

C	0.98636297793160	-0.42687473123470	7.53695282860309
C	1.47294775095030	-0.19978351664663	6.09787203711643
H	0.19983739730656	-6.58198142530044	6.49312272531760
H	2.40222422714885	0.35307566677666	5.99160372770551
C	0.45458427526794	-5.57205973167563	6.18793664066038
H	-1.38157148947951	-5.29625079043151	5.09876817936310
C	-0.43967111387017	-4.85005463259801	5.40278122138651
C	1.67636725803026	-5.02545441655325	6.58088781927465
H	2.36343274599990	-5.60989752226651	7.18396533706525
H	-2.69271207954556	-3.71905656863773	3.77237972914615
C	-0.11301174314729	-3.55299621766692	5.01285362502419
C	-2.16122723852647	-2.77936289306332	3.65744697303568
C	2.03143390148624	-3.73477464676178	6.19223377840380
C	-0.89624481553315	-2.60983292243467	4.22015365821307
C	1.11508722468712	-3.03174196895033	5.43500383564164
C	-2.73570783406212	-1.73644396880980	2.93768193523751
H	-3.72034084914269	-1.86993564234950	2.50084617582056
H	2.98418415645794	-3.30361133683582	6.48376497215632
C	-0.25681071552275	-1.37922052503259	4.02970015748965
C	-2.06159778701899	-0.52827736780402	2.75121994294648
S	1.38509686162967	-1.38427517354660	4.76536518091890
C	-0.79019439563881	-0.33990112415110	3.29040459814587
H	-2.52001574950426	0.26524197925670	2.16999040304220
H	-0.24614287839635	0.58721818840197	3.13485099007836
N	-1.02729782451038	0.25602585340637	6.58051174351878
C	-1.20913899309873	-0.98944307344186	7.15026202049573
C	0.32244390497606	0.65099050213904	6.69741270316586
C	-0.08730520470510	-1.42028888652721	7.75907618489932
H	0.01251018824009	-2.30842579101919	8.36436632121823
H	0.53592925002547	1.71374598217448	6.76162443334027

H	1.72447560347491	-0.21650871180176	8.30525688903441
H	-2.17412146734326	-1.47703964192829	7.08398237821626
H	-1.65470754456730	0.65331405531408	5.90126085431801

33

TS4_endo r2scan-3c

C	2.81099094381445	-0.52866257864736	4.64651187685375
C	4.00911852666202	0.37489591041581	5.65714126834595
C	3.92906112017215	0.31151874214848	4.19600753036759
H	-1.60360375262302	1.70307600423575	1.26836973619724
H	4.76582234105919	-0.22827354833941	3.75470004149034
C	-0.56664759733022	1.69529448118985	1.58882072014968
H	-0.78724270977462	3.46708703378280	2.79078962120447
C	-0.10997384136833	2.69148194342699	2.44683001278665
C	0.28514783725147	0.69174304463895	1.12695808957086
H	-0.08986128609581	-0.07054009387968	0.45203344676305
H	0.37437589894211	5.01960105995558	4.28510937422104
C	1.22021830391488	2.68213065923916	2.85963101561817
C	1.40567190255897	4.71238808485793	4.42906038235850
C	1.62346031318342	0.66629279634617	1.51436783872579
C	1.90519593852316	3.59114950264319	3.76958198598808
C	2.04983547267472	1.65935136347567	2.37738369948594
C	2.24361990518714	5.44502551049340	5.26447151798657
H	1.85362926137398	6.31940429002437	5.77581297502379
H	2.29606180031513	-0.10244663714494	1.14715896208281
C	3.24790315656688	3.24777515797069	3.98642901951125
C	3.58118156622320	5.08827710881595	5.44003924605908
S	3.70923515337276	1.82365533152764	3.00796124283328
C	4.10929707505592	3.97897454021178	4.78345805774851
H	4.22199242210417	5.68450140743323	6.08132349673302
H	5.15212111429488	3.70270198748783	4.90253473039862

N	3.16504208380251	1.18531547265579	6.40285994102423
C	1.82930241100361	1.06015543017833	6.07250046415799
C	1.60718099517873	0.09421859361864	5.15375211710225
H	0.62062669236900	-0.24010158109090	4.86114000764122
H	4.91133160674250	0.01110381276399	6.14046797802861
H	2.81135684296734	-1.57149064909567	4.33607832483139
H	1.09865339024592	1.68987691948382	6.56473478496519
H	3.43152736803172	1.41738939597609	7.34691270714494

33

TS4_exo r2scan-3c

C	0.18636150792727	-0.65701522647495	7.36397107786144
C	0.56550920360502	0.99308625975887	6.32389574299912
C	1.26600489058530	-0.29774995031704	6.41853108936973
H	1.03310616721387	-6.50469811343748	6.68992772478202
H	2.29764415094701	-0.27541611904582	6.80820497451236
C	1.13748027190223	-5.48271051303478	6.33974678204076
H	-0.87884147379631	-5.39482678488112	5.59228467764195
C	0.05741213585302	-4.86079746234205	5.72116896804104
C	2.35305185659516	-4.82111166964004	6.51231404977904
H	3.18479245659268	-5.32820611776448	6.98986301574260
H	-2.49927084755111	-4.00678099258086	4.40690765534940
C	0.19147346190152	-3.54917604864660	5.26924040180338
C	-2.08491765665210	-3.02705238006015	4.19076699138513
C	2.51540914161088	-3.51107591417451	6.06432675262429
C	-0.78750567731327	-2.70828581488821	4.58728582507691
C	1.42536115521339	-2.91485200589659	5.45971242399106
C	-2.84047042558634	-2.08411249811566	3.50113540677924
H	-3.85015403551152	-2.33431495590668	3.19150983807829
H	3.46152326754391	-2.99276204478048	6.18453171203569
C	-0.30063637980537	-1.42986960667875	4.27692517564074

C	-2.31566666707585	-0.83285058246534	3.17839557210561
S	1.39533258930897	-1.23079760263959	4.83334492449885
C	-1.01829088406335	-0.49227817914950	3.55467280081249
H	-2.91241674559744	-0.12173854250253	2.61694179321705
H	-0.59274578739946	0.46590748137585	3.27413675506116
N	0.77946076304697	1.83737935489929	7.38219739880991
C	0.76449843409974	1.25057048546943	8.63323515346866
C	0.33174587968479	-0.04763524300609	8.63758017574184
H	0.02361904906957	-0.53869065489793	9.55289259045600
H	0.02311411214590	1.33943446091471	5.45371169775848
H	-0.52535588751106	-1.44739039736206	7.14581225985304
H	0.97216419407726	1.86821195099916	9.49697755011537
H	0.53728277893867	2.81357642727224	7.29518904256638

12

pyridine protonated r2scan-3c

C	-2.63995378926984	2.32819895075002	0.00000000092765
C	-2.55950736181687	0.95190522650079	-0.00000000043499
C	-1.46445448507279	3.07670408234997	0.00000000140313
C	-0.22726292722935	2.43518380466917	-0.00000000044273
N	-1.34427447346052	0.36456876953100	0.00000000151598
C	-0.18577495551435	1.05710628364161	0.00000000107815
H	0.73149712907486	0.47815931853125	0.00000000037560
H	0.70088309183173	2.99549695211405	-0.00000000275659
H	-3.61397710438054	2.80428950870692	0.00000000118612
H	-1.51262684893709	4.16142788205716	-0.00000000006768
H	-3.42201208381675	0.29415890217446	-0.00000000367964
H	-1.29931795730853	-0.65000829862642	0.00000000089500

REFERENCES

[1] Sheldrick, G. M. A short history of SHELX. *Acta Cryst. A* **2008**, *64*, 112–122.

[2] Dolomanov, O. V.; Bourhis, L. J.; Gildea, R. J.; Howard, J. A. K.; Puschmann, H. OLEX2. *J. Appl. Cryst.* **2009**, *42*, 339–341.

[3] Williams, C. M.; Mander, L. N. Chromatography with silver nitrate. *Tetrahedron* **2001**, *57*, 425–447.

[4] Li, T.-S.; Li, J.-T.; Li, H.-Z. Modified and convenient preparation of silica impregnated with silver nitrate and its application to the separation of steroids and triterpenes. *J. Chromatogr. A* **1995**, *715*, 372–375.

[5] Mander, L. N.; Williams, C. M. Chromatography with silver nitrate: part 2. *Tetrahedron* **2016**, *72* (9), 1133–1150.

[6] Waldecker, B.; Kafuta, K.; Alcarazo, M. Preparation of 5-(Triisopropylalkynyl) dibenzo[b,d]thiophenium triflate. *Org. Synth.* **2019**, *96*, 258–276.

[7] Simkó, D. C.; Elekes, P.; Pázmándi, V.; Novák, Z. Sulfonium Salts as Alkylating Agents for Palladium-Catalyzed Direct Ortho Alkylation of Anilides and Aromatic Ureas. *Org. Lett.* **2018**, *20* (3), 676–679.

[8] Wu, F.-P.; Chintawar, C. C.; Lalisse, R.; Mukherjee, P.; Dutta, S.; Tyler, J.; Daniliuc, C. G.; Gutierrez, O.; Glorius, F. Ring expansion of indene by photoredox-enabled functionalized carbon-atom insertion. *Nat. Catal.* **2024**, *7* (3), 242–251.

[9] Timmann, S.; Wu, T.-H.; Golz, C.; Alcarazo, M. Reactivity of α-diazo sulfonium salts: rhodium-catalysed ring expansion of indenes to naphthalenes. *Chem. Sci.* **2024**, *15* (16), 5938–5943.

[10] Finkelstein, P.; Reisenbauer, J. C.; Botlik, B. B.; Green, O.; Florin, A.; Morandi, B. Nitrogen atom insertion into indenes to access isoquinolines. *Chem. Sci.* **2023**, *14* (11), 2954–2959.

[11] Deng, R.; Sun, L.; Li, Z. Nickel-catalyzed carboannulation reaction of o-bromobenzyl zinc bromide with unsaturated compounds. *Org. Lett.* **2007**, *9* (25), 5207–5210.

[12] Zhou, Q.; Li, S.; Zhang, Y.; Wang, J. Rhodium(II)- or Copper(I)-Catalyzed Formal Intramolecular Carbene Insertion into Vinylic C(sp2)-H Bonds: Access to Substituted 1H-Indenes. *Angew. Chem. Int. Ed.* **2017**, *56* (50), 16013–16017.

[13] Wang, Z.; Xu, H.; Han, X.; Fan, S.; Zhu, J. Manganese-Catalyzed Cycloalkene Ring Expansion Synthesis of Azaheterocycles. *Org. Lett.* **2024**, *26* (40), 8559–8564.

[14] Xie, L.-G.; Wang, Z.-X. Cross-coupling of aryl/alkenyl ethers with aryl Grignard reagents through nickel-catalyzed C-O activation. *Chem. Eur. J.* **2011**, *17* (18), 4972–4975.

[15] Kang, T.; Cao, W.; Hou, L.; Tang, Q.; Zou, S.; Liu, X.; Feng, X. Chiral Zinc(II)-Catalyzed Enantioselective Tandem α-Alkenyl Addition/Proton Shift Reaction of Silyl Enol Ethers with Ketimines. *Angew. Chem. Int. Ed.* **2019**, *58* (8), 2464–2468.

[16] Timmann, S.; Dilchert, M. T. H.; Dietzel, J.; Pöltl, V. S.; Wennekamp, M. R.; Golz, C.; Alcarazo, M. A Photocatalytic Approach to Radical 1-(Trifluoromethyl)cyclopropanation. *ACS Catal.* **2025**, *15* (9), 7232–7240.

[17] Zaikina, L. A.; Mulina, O. M.; Merkulova, V. M.; Ilovaisky, A. I.; Vil', V. A.; Terent'ev, A. O. Electrochemically Induced Synthesis of β-Ketosulfones from Enol Acetates and Sodium Sulfinates. *ChemistrySelect* **2024**, *9* (36), e202403708.

[18] Basdevant, B.; Legault, C. Y. Enantioselective Iodine(III)-Mediated Synthesis of α-Tosyloxy Ketones: Breaking the Selectivity Barrier. *Org. Lett.* **2015**, *17* (19), 4918–4921.

[19] Heilmann, T.; Lopez-Soria, J. M.; Ulbrich, J.; Kircher, J.; Li, Z.; Worbs, B.; Golz, C.; Mata, R. A.; Alcarazo, M. N-(Sulfonio)Sulfilimine Reagents: Non-Oxidizing Sources of Electrophilic Nitrogen Atom for Skeletal Editing. *Angew. Chem. Int. Ed.* **2024**, *63* (25), e202403826.

[20] Detty, M. R.; Murray, B. J.; Smith, D. L.; Zumbulyadis, N. Cyclization of 3-(arylchalcogeno)propenoyl chlorides. 1. 1,2-Oxatellurol-1-ium halides via ipso acylation. *J. Am. Chem. Soc.* **1983**, *105* (4), 875–882.

[21] Friedfeld, M. R.; Shevlin, M.; Margulieux, G. W.; Campeau, L.-C.; Chirik, P. J. Cobalt-Catalyzed Enantioselective Hydrogenation of Minimally Functionalized Alkenes: Isotopic Labeling Provides Insight into the Origin of Stereoselectivity and Alkene Insertion Preferences. *J. Am. Chem. Soc.* **2016**, *138* (10), 3314–3324.

[22] Wu, Q.; Han, S.; Ren, X.; Lu, H.; Li, J.; Zou, D.; Wu, Y.; Wu, Y. Pd-Catalyzed Alkylation of (Iso)quinolines and Arenes: 2-Acylpyridine Compounds as Alkylation Reagents. *Org. Lett.* **2018**, *20* (20), 6345–6348.

[23] Igarashi, T.; Haito, A.; Chatani, N.; Tobisu, M. Nickel-Catalyzed Reductive Cleavage of Carbon–Oxygen Bonds in Anisole Derivatives Using Diisopropylaminoborane. *ACS Catal.* **2018**, *8* (8), 7475–7483.

[24] He, Q.; Wang, L.; Liang, Y.; Zhang, Z.; Wnuk, S. F. Transition-Metal-Free Cross-Coupling of Aryl Halides with Arylstannanes. *J. Org. Chem.* **2016**, *81* (19), 9422–9427.

[25] Xiang, S.; Hu, H.; Ma, J.; Li, Y.; Wang, B.; Feng, C.; Zhao, K.; Hu, P.; Chen, X. Synthesis of naphthalene derivatives through inexpensive BF3·Et2O-catalyzed annulation reaction of arylacetaldehydes with arylalkynes. *Sci. China Chem.* **2013**, *56* (7), 945–951.

[26] Ackermann, L.; Kapdi, A. R.; Fenner, S.; Kornhaass, C.; Schulzke, C. Well-defined air-stable palladium HASPO complexes for efficient Kumada-Corriu cross-couplings of (hetero)aryl or alkenyl tosylates. *Chem. Eur. J.* **2011**, *17* (10), 2965–2971.

[27] Sahoo, B.; Surkus, A.-E.; Pohl, M.-M.; Radnik, J.; Schneider, M.; Bachmann, S.; Scalone, M.; Junge, K.; Beller, M. A Biomass-Derived Non-Noble Cobalt Catalyst for Selective

Hydrodehalogenation of Alkyl and (Hetero)Aryl Halides. *Angew. Chem. Int. Ed.* **2017**, *56* (37), 11242–11247.

[28] Bose, A.; Mal, P. Electrophilic aryl-halogenation using N-halosuccinimides under ball-milling. *Tetrahedron Lett.* **2014**, *55* (13), 2154–2156.

[29] Pan, J.; Wang, X.; Zhang, Y.; Buchwald, S. L. An improved palladium-catalyzed conversion of aryl and vinyl triflates to bromides and chlorides. *Org. Lett.* **2011**, *13* (18), 4974–4976.

[30] Bazyar, Z.; Hosseini-Sarvari, M. On/Off O2 Switchable Photocatalytic Oxidative and Protodecarboxylation of Carboxylic Acids. *J. Org. Chem.* **2019**, *84* (21), 13503–13515.

[31] Dhokale, R. A.; Mhaske, S. B. Nucleophilic Nitration of Arynes by Sodium Nitrite and its Multicomponent Reaction Leading to Double-Functionalized Arenes. *Org. Lett.* **2016**, *18* (12), 3010–3013.

[32] Choi, H. Y.; Srisook, E.; Jang, K. S.; Chi, D. Y. Electrophilic aromatic addition reaction: electrophilic attack at an aromatic H substituent position. *J. Org. Chem.* **2005**, *70* (4), 1222–1226.

[33] Iwai, T.; Konishi, S.; Miyazaki, T.; Kawamorita, S.; Yokokawa, N.; Ohmiya, H.; Sawamura, M. Silica-Supported Triptycene-Type Phosphine. Synthesis, Characterization, and Application to Pd-Catalyzed Suzuki–Miyaura Cross-Coupling of Chloroarenes. *ACS Catal.* **2015**, *5* (12), 7254–7264.

[34] Ikonnikova, V. A.; Solyev, P. N.; Terekhov, S. S.; Alferova, V. A.; Tyurin, A. P.; Korshun, V. A.; Baranov, M. S.; Mikhaylov, A. A. Total Synthesis of Elmenols A and B and Related Rearranged Angucyclinones. *ChemistrySelect* **2021**, *6* (42), 11775–11778.

[35] Kumar, M.; Bagchi, S.; Sharma, A. The first vinyl acetate mediated organocatalytic transesterification of phenols: a step towards sustainability. *New J. Chem.* **2015**, *39* (11), 8329–8336.

[36] Molander, G. A.; Beaumard, F. Nickel-catalyzed C-O activation of phenol derivatives with potassium heteroaryltrifluoroborates. *Org. Lett.* **2010**, *12* (18), 4022–4025.

[37] Mutule, I.; Suna, E. Arylzinc species by microwave assisted Grignard formation–transmetallation sequence: application in the Negishi coupling. *Tetrahedron* **2005**, *61* (47), 11168–11176.

[38] Desmarets, C.; Omar-Amrani, R.; Walcarius, A.; Lambert, J.; Champagne, B.; Fort, Y.; Schneider, R. Naphthidine di(radical cation)s-stabilized palladium nanoparticles for efficient catalytic Suzuki–Miyaura cross-coupling reactions. *Tetrahedron* **2008**, *64* (2), 372–381.

[39] Zhang, B.; Chakma, P.; Shulman, M. P.; Ke, J.; Digby, Z. A.; Konkolewicz, D. Probing the mechanism of thermally driven thiol-Michael dynamic covalent chemistry. *Org. Biomol. Chem.* **2018**, *16* (15), 2725–2734.

[40] Zhang, Y.; Li, X.; Xu, L.; Xie, X.; Lu, Y.; Zhang, Z. Ru-Catalyzed Enantioselective Hydrogenation of Diaryl 1,4-Diketones: Synthesis of Chiral 1,4-Diarylbutane-1,4-Diols. *Org. Lett.* **2022**, *24* (41), 7512–7516.

[41] Zhao, M.; Liu, Y.; Chen, X.; Peng, M.; Wang, Y.; Liu, X.; Jiang, H.; Tan, R.; Li, J. Photocatalyst-free formate-mediated C-O cleavage by the EDA complex and SCS strategy for the synthesis of diaryl 1,4-diketone in air. *Org. Biomol. Chem.* **2025**, *23* (9), 2079–2085.

[42] Liu, Y.; Liu, S.; Li, D.; Zhang, N.; Peng, L.; Ao, J.; Song, C. E.; Lan, Y.; Yan, H. Kinetic Resolution of Allylic Alcohol with Chiral BINOL-Based Alkoxides: A Combination of Experimental and Theoretical Studies. *J. Am. Chem. Soc.* **2019**, *141* (2), 1150–1159.

[43] Hartmann, A. P.; Carvalho, M. R. de; Bernardes, L. S. C.; Moraes, M. H. de; Melo, E. B. de; Lopes, C. D.; Steindel, M.; Da Silva, J. S.; Carvalho, I. Synthesis and 2D-QSAR studies of neolignan-based diaryl-tetrahydrofuran and -furan analogues with remarkable activity against Trypanosoma cruzi and assessment of the trypanothione reductase activity. *Eur. J. Med. Chem.* **2017**, *140*, 187–199.

[44] Reisenbauer, J. C.; Paschke, A.-S. K.; Krizic, J.; Botlik, B. B.; Finkelstein, P.; Morandi, B. Direct Access to Quinazolines and Pyrimidines from Unprotected Indoles and Pyrroles through Nitrogen Atom Insertion. *Org. Lett.* **2023**, *25* (47), 8419–8423.

[45] Zhen, Q.; Li, R.; Qi, L.; Hu, K.; Yao, X.; Shao, Y.; Chen, J. Nickel(II)-catalyzed C–C, N–C cascade coupling of ketonitriles into substituted pyrroles and pyridines. *Org. Chem. Front.* **2020**, *7* (2), 286–291.

[46] van Rossom, W.; Matsushita, Y.; Ariga, K.; Hill, J. P. New synthesis of unsymmetrically-substituted 2,5-diarylpyrroles from homopropargyl sulfonamides. *RSC Adv.* **2014**, *4* (10), 4897.

[47] Wen, J.; Qin, S.; Ma, L.-F.; Dong, L.; Zhang, J.; Liu, S.-S.; Duan, Y.-S.; Chen, S.-Y.; Hu, C.-W.; Yu, X.-Q. Iron-mediated direct Suzuki-Miyaura reaction: a new method for the ortho-arylation of pyrrole and pyridine. *Org. Lett.* **2010**, *12* (12), 2694–2697.

[48] Cen, J.; Wu, Y.; Li, J.; Huang, L.; Wu, W.; Zhu, Z.; Yang, S.; Jiang, H. Switchable Reactivity between Vinyl Azides and Terminal Alkyne by Nano Copper Catalysis. *Org. Lett.* **2019**, *21* (7), 2090–2094.

[49] Ma, Z.; Liu, H.; Zhang, C.; Zheng, X.; Yuan, M.; Fu, H.; Li, R.; Chen, H. One-Pot Synthesis of Symmetrical 2,6-Diarylpyridines via Palladium/Copper-Catalyzed Sequential Decarboxylative and Direct C□H Arylation. *Adv. Synth. Catal.* **2015**, *357* (6), 1143–1148.

[50] Shen, Y.; Chen, J.; Liu, M.; Ding, J.; Gao, W.; Huang, X.; Wu, H. Copper-catalyzed direct C-H arylation of pyridine N-oxides with arylboronic esters: one-pot synthesis of 2-arylpyridines. *Chem. Commun.* **2014**, *50* (33), 4292–4295.

[51] Yin, C.; Zhong, K.; Li, W.; Yang, X.; Sun, R.; Zhang, C.; Zheng, X.; Yuan, M.; Li, R.; Lan, Y.; Fu, H.; Chen, H. C 6 -Selective Direct Arylation of 2-Phenylpyridine via an Activated N -

methylpyridinium Salt: A Combined Experimental and Theoretical Study. *Adv. Synth. Catal.* **2018**, *360* (20), 3990–3998.

[52] Mee, S. P. H.; Lee, V.; Baldwin, J. E. Significant enhancement of the Stille reaction with a new combination of reagents-copper(I) iodide with cesium fluoride. *Chem. Eur. J.* **2005**, *11* (11), 3294–3308.

[53] Paul, S.; Guin, J. Dioxygen-Mediated Decarbonylative C-H Alkylation of Heteroaromatic Bases with Aldehydes. *Chem. Eur. J.* **2015**, *21* (49), 17618–17622.

[54] Wang, Y.-F.; Chiba, S. Mn(III)-mediated reactions of cyclopropanols with vinyl azides: synthesis of pyridine and 2-azabicyclo3.3.1non-2-en-1-ol derivatives. *J. Am. Chem. Soc.* **2009**, *131* (35), 12570–12572.

[55] Gupta, S.; Sureshbabu, P.; Singh, A. K.; Sabiah, S.; Kandasamy, J. Deoxygenation of tertiary amine N-oxides under metal free condition using phenylboronic acid. *Tetrahedron Lett.* **2017**, *58* (10), 909–913.

[56] Son, J.-K.; Zhao, L.-X.; Basnet, A.; Thapa, P.; Karki, R.; Na, Y.; Jahng, Y.; Jeong, T. C.; Jeong, B.-S.; Lee, C.-S.; Lee, E.-S. Synthesis of 2,6-diaryl-substituted pyridines and their antitumor activities. *Eur. J. Med. Chem.* **2008**, *43* (4), 675–682.

[57] Zhang, X.; Feng, X.; Zhou, C.; Yu, X.; Yamamoto, Y.; Bao, M. Transition-Metal-Free Decarboxylative Arylation of 2-Picolinic Acids with Arenes under Air Conditions. *Org. Lett.* **2018**, *20* (22), 7095–7099.

[58] Flitsch, W.; Lubisch, W. Zur Umsetzung von 2-Pyrrolcarbaldehyd mit heterosubstituierten Ethenen. *Chem. Ber.* **1984**, *117* (4), 1424–1435.

[59] Frisch, M. J.; Trucks, G. W.; Schlegel, H. B.; Scuseria, G. E.; Robb, M. A.; Cheeseman, J. R.; Scalmani, G.; Barone, V.; Petersson, G. A.; Nakatsuji, H.; Li, X.; Caricato, M.; Marenich, A. V.; Bloino, J.; Janesko, B. G.; Gomperts, R.; Mennucci, B.; Hratchian, H. P.; Ortiz, J. V.; Izmaylov, A. F.; Sonnenberg, J. L.; Williams-Young, D.; Ding, F.; Lipparini, F.; Egidi, F.; Goings, J.; Peng, B.; Petrone, A.; Henderson, T.; Ranasinghe, D.; Zakrzewski, V. G.; Gao, J.; Rega, N.; Zheng, G.; Liang, W.; Hada, M.; Ehara, M.; Toyota, K.; Fukuda, R.; Hasegawa, J.; Ishida, M.; Nakajima, T.; Honda, Y.; Kitao, O.; Nakai, H.; Vreven, T.; Throssell, K.; Montgomery, Jr., J. A.; Peralta, J. E.; Ogliaro, F.; Bearpark, M. J.; Heyd, J. J.; Brothers, E. N.; Kudin, K. N.; Staroverov, V. N.; Keith, T. A.; Kobayashi, R.; Normand, J.; Raghavachari, K.; Rendell, A. P.; Burant, J. C.; Iyengar, S. S.; Tomasi, J.; Cossi, M.; Millam, J. M.; Klene, M.; Adamo, C.; Cammi, R.; Ochterski, J. W.; Martin, R. L.; Morokuma, K.; Farkas, O.; Foresman, J. B.; Fox, D. J. *Gaussian 16 Revision A.03*, 2016.

[60] Becke, A. D. DFT calculations. *Phys. Rev. A* **1988**, *38*, 3098–3100.

[61] Grimme, S.; Antony, J.; Ehrlich, S.; Krieg, H. A consistent and accurate ab initio parametrization of density functional dispersion correction (DFT-D) for the 94 elements H-Pu. *J. Chem. Phys.* **2010**, *132* (15), 154104.

[62] Grimme, S.; Ehrlich, S.; Goerigk, L. Effect of the Damping Function in Dispersion Corrected Density Functional Theory. *J. Comput. Chem.* **2011**, *32* (7), 1456–1465.

[63] Schäfer, A.; Horn, H.; Ahlrichs, R. DFT calculations. *J. Chem. Phys.* **1992**, *97*, 2571–2577.

[64] Weigend, F. DFT calculations. *Phys. Chem. Chem. Phys.* **2006**, *8*, 1057–1065.

[65] Weigend, F.; Ahlrichs, R. Balanced basis sets of split valence, triple zeta valence and quadruple zeta valence quality for H to Rn: Design and assessment of accuracy. *Phys. Chem. Chem. Phys.* **2005**, *7*, 3297–3305.

[66] Becke, A. D. Density-functional thermochemistry. III. The role of exact exchange. *J. Chem. Phys.* **1993**, *98* (7), 5648–5652.

[67] Knizia, G. Intrinsic Atomic Orbitals: An Unbiased Bridge between Quantum Theory and Chemical Concepts. *J. Chem. Theory Comput.* **2013**, *9* (11), 4834–4843.

[68] Knizia, G.; Klein, J. E. M. N. Electron flow in reaction mechanisms--revealed from first principles. *Angew. Chem. Int. Ed.* **2015**, *54* (18), 5518–5522.

[69] Lu, T.; Chen, F. Multiwfn: a multifunctional wavefunction analyzer. *J. Comput. Chem.* **2012**, *33* (5), 580–592.

[70] Bannwarth, C.; Ehlert, S.; Grimme, S. GFN2-xTB-An Accurate and Broadly Parametrized Self-Consistent Tight-Binding Quantum Chemical Method with Multipole Electrostatics and Density-Dependent Dispersion Contributions. *J. Chem. Theory Comput.* **2019**, *15* (3), 1652–1671.

[71] Grimme, S.; Hansen, A.; Ehlert, S.; Mewes, J.-M. r2SCAN-3c: A "Swiss army knife" composite electronic-structure method. *J. Chem. Phys.* **2021**, *154* (6), 64103.

[72] Neese, F. Software update: The ORCA program system—Version 5.0. *WIREs Comput Mol Sci.* **2022**, *12* (5).

[73] Grimme, S. Supramolecular binding thermodynamics by dispersion-corrected density functional theory. *Chem. Eur. J.* **2012**, *18* (32), 9955–9964.

[74] Marenich, A. V.; Cramer, C. J.; Truhlar, D. G. Universal solvation model based on solute electron density and on a continuum model of the solvent defined by the bulk dielectric constant and atomic surface tensions. *J. Phys. Chem. B* **2009**, *113* (18), 6378–6396.

[75] a) Schleyer, P. v. R.; Maerker, C.; Dransfeld, A.; Jiao, H.; van Eikema Hommes, N. J. R. Nucleus-Independent Chemical Shifts: A Simple and Efficient Aromaticity Probe. *J. Am. Chem. Soc.* **1996**, *118*, 6317-6318; b) Chen, Z.; Wannere, C. S.; Corminboeuf, C.; Puchta, R.; Schleyer, P. v. R. Nucleus-Independent Chemical Shifts (NICS) as an Aromaticity Criterion. *Chem. Rev.* **2005**, *105*, 3842-3888.
